# *Serratia liquefaciens* FG3 isolated from a metallophyte plant sheds light on the evolution and mechanisms of adaptive traits in extreme environments

**DOI:** 10.1038/s41598-019-54601-4

**Published:** 2019-11-29

**Authors:** Washington Luiz Caneschi, Angélica Bianchini Sanchez, Érica Barbosa Felestrino, Camila Gracyelle de Carvalho Lemes, Isabella Ferreira Cordeiro, Natasha Peixoto Fonseca, Morghana Marina Villa, Izadora Tabuso Vieira, Lauro Ângelo Gonçalves Moraes, Renata de Almeida Barbosa Assis, Flávio Fonseca do Carmo, Luciana Hiromi Yoshino Kamino, Robson Soares Silva, Jesus Aparecido Ferro, Maria Inês Tiraboschi Ferro, Rafael Marini Ferreira, Vera Lúcia Santos, Ubiana de Cássia Mourão Silva, Nalvo Franco Almeida, Alessandro de Mello Varani, Camila Carrião Machado Garcia, João Carlos Setubal, Leandro Marcio Moreira

**Affiliations:** 1 Núcleo de Pesquisas em Ciências Biológicas (NUPEB), Universidade Federal de Ouro Preto (UFOP), Ouro Preto, MG Brazil; 2 Instituto Prístino, Belo Horizonte, MG Brazil; 3 Faculdade de Computação (FACOM), Universidade Federal de Mato Grosso do Sul, Campo Grande, MS Brazil; 4 Faculdade de Ciências Agrárias e Veterinárias de Jaboticabal, UNESP – Universidade Estadual Paulista, Departamento de Tecnologia, SP, Brazil; 5 Departamento de Microbiologia, Universidade Federal de Minas Gerais (UFMG), Belo Horizonte, MG Brazil; 6 Departamento de Ciências Biológicas (DECBI), Instituto de Ciências Exatas e Biológicas (ICEB), Universidade Federal de Ouro Preto (UFOP), Ouro Preto, MG Brazil; 7 Departamento de Bioquímica (DB), Instituto de Química (IQ), Universidade de São Paulo (USP), São Paulo, SP Brazil; 8 Biocomplexity Institute, Virginia Tech, Blacksburg, VA USA

**Keywords:** Comparative genomics, Genome evolution, Molecular evolution

## Abstract

*Serratia liquefaciens* strain FG3 (SlFG3), isolated from the flower of *Stachytarpheta glabra* in the Brazilian ferruginous fields, has distinctive genomic, adaptive, and biotechnological potential. Herein, using a combination of genomics and molecular approaches, we unlocked the evolution of the adaptive traits acquired by S1FG3, which exhibits the second largest chromosome containing the largest conjugative plasmids described for *Serratia*. Comparative analysis revealed the presence of 18 genomic islands and 311 unique protein families involved in distinct adaptive features. S1FG3 has a diversified repertoire of genes associated with Nonribosomal peptides (NRPs/PKS), a complete and functional cluster related to cellulose synthesis, and an extensive and functional repertoire of oxidative metabolism genes. In addition, S1FG3 possesses a complete pathway related to protocatecuate and chloroaromatic degradation, and a complete repertoire of genes related to DNA repair and protection that includes mechanisms related to UV light tolerance, redox process resistance, and a laterally acquired capacity to protect DNA using phosphorothioation. These findings summarize that SlFG3 is well-adapted to different biotic and abiotic stress situations imposed by extreme conditions associated with ferruginous fields, unlocking the impact of the lateral gene transfer to adjust the genome for extreme environments, and providing insight into the evolution of prokaryotes.

## Introduction

*Serratia* comprises a genus of gram-negative bacteria belonging to the Enterobacteriaceae family. It has a cosmopolitan distribution and is associated with different environments. Among the several species belonging to this genus, *S. marcencens* is the best-studied, since it is associated with human opportunistic pathologies^1,2^.

More recently, other species from the *Serratia* genus have gained notoriety, either because they present reduced genome size, such as *S. symbiotica*^3^, or because they are necessarily associated with plants and rhizospheres, as is the case for *S. phymuthica*^4^ and *S. fonticola*^5^ strains.

*S. liquefaciens* is a species still poorly characterized. Three strains of this species have undergone genome sequencing: ATCC27592^6^, an environmental isolate capable of growing under low pressure conditions; strain HUMV-21, a human opportunistic pathogen^7^ with high biofilm production potential; and FDAARGOS-125^8^, which is relevant in validation of infectious disease tests.

Recently, we investigated^9^ the biotechnological potential of a bacterial strain provisionally identified as *S. liquefaciens*, isolated from a plant flower (*Stachytarpheta glabra* Cham.), in the ferruginous rupestrian fields of the Iron Quadrangle (IQ) region of Minas Gerais State, in Brazil^10^, at an altitude of 1,400 m. The IQ displays extreme environmental conditions for microbial communities, including a harsh soil rich in metals, especially iron, manganese, and arsenic, poor in nitrogen, phosphate, and organic matter, and suffering high UV ray incidence, with temperatures ranging from 70 °C during the day to 4 °C at night^11^. Despite these adverse conditions, the surviving plants are highly adapted, presenting a high degree of endemism^11^ and biodiversity^12^, characterizing the region as an important conservation hotspot^13^.

The *S. liquefaciens* strain, which we named *S. liquefaciens* SlFG3, was shown to possess some remarkable characteristics, such as the capability of producing siderophores, exhibiting resistance to ampicillin and arsenic, and presenting high bacterial turnover rates. Molecular analysis allowed the identification of two plasmids associated with this bacterium, pFG3A and pFG3B, the latter responsible for conferring resistance to ampicillin, reducing the production of reactive oxygen species, elevating catalase activity, reducing DNA degradation and increasing bacterial turnover in transformed *E. coli*^10^.

Based on the observed phenotypes of the SlFG3 strain, we decided to sequence its genome, aiming to uncover which genes could be related to the observed phenotypes. The results we present here show that strain SlFG3 is unique, and has potential for several biotechnological applications.

## Results

### Structure and general features of SlFG3 genome

**Figure 1:**
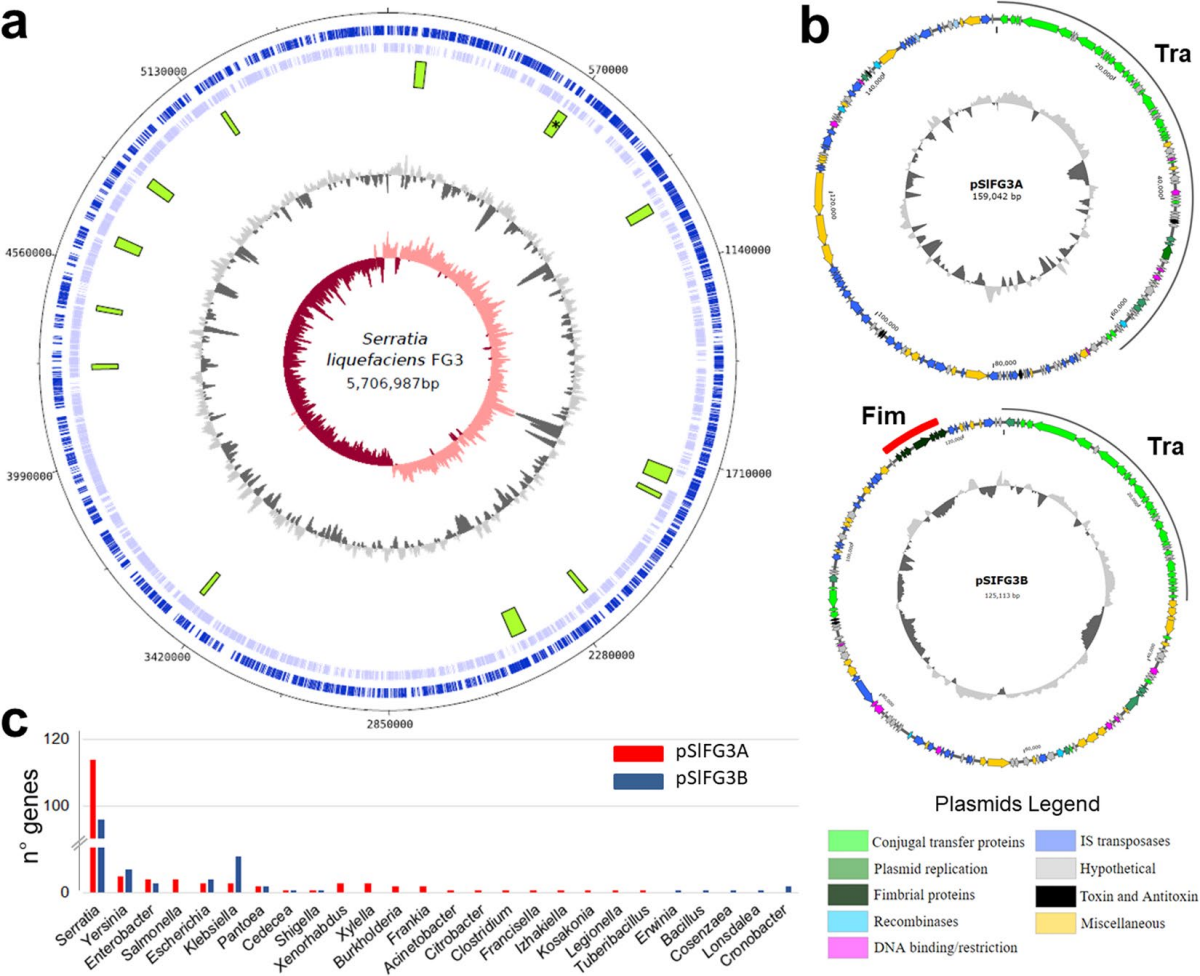
Circular map of chromosome (**a**) and plasmids (**b**) of SlFG3. Chromosome - dark blue and light bars represent, respectively, genes annotated in the + and - DNA strand; Green blocks - anomalous regions; Dark gray circle - GC content; Salmon and wine lines - GC cumulative content. Plasmids - arrows determine the strand encoding the genes with respective functions presented in the legend; Tra - conjugation genes (light green); Fim - fimbriae genes (red bar). (**c**) Histogram that determines the representativeness of the plasmid genes for the genres identified as best comparison hits.

The SlFG3 presents a chromosome of 5,706,987 bp encoding 5,398 putative genes and with 55% GC content (Fig. 1a and Supplementary Table 1). Analysis of the chromosome by the PHAST program revealed the presence of 13 regions that were characterized as phage insertions.

Two complete conjugative plasmids were identified, one spanning 159,042 bp (SlpFG3A) and one spanning 125,113 bp (SlpFG3B) encoding 179 and 146 putative genes, respectively (Fig. 1b and Supplementary Table 1). They are the two largest conjugative plasmids identified in genomes from the *Serratia* genus. Most (96,2%) of the plasmid genes were found in other bacteria from the *Serratia* genus, or belonged to the Enterobacterales order, the remaining genes (3,8%) code for hypothetical proteins and transposases (Fig. 1c and Supplementary Table 2).

### *Serratia* genome comparison and phylogenomics

**Figure 2:**
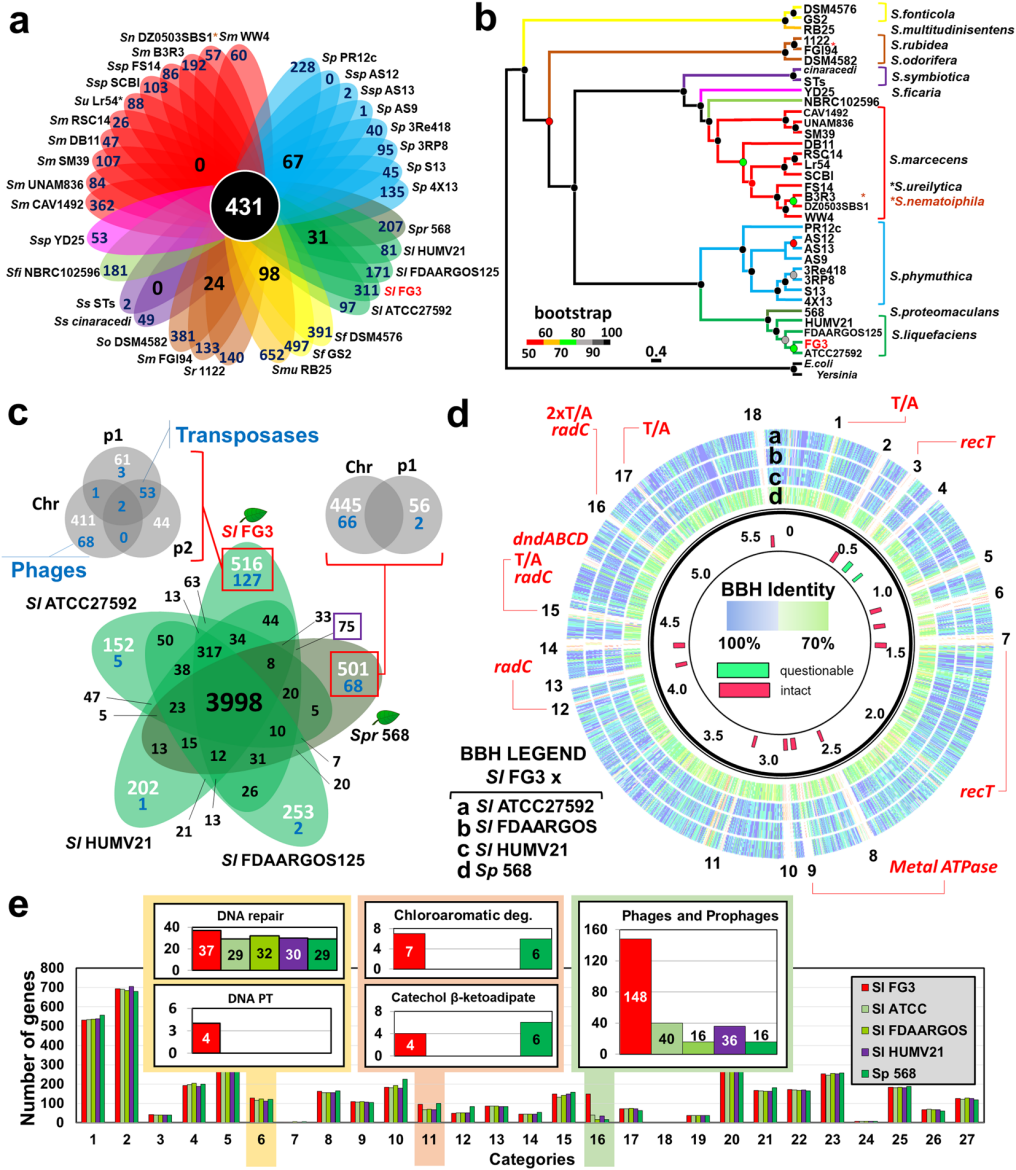
Comparative analysis of SlFG3 genome with other complete genomes of bacteria from the *Serratia* genus. (**a**) Flower plot that highlights the number of unique, core, and flexible genes. (**b**) Phylogenomic analysis determined from the core genome. *Yersinia pestis* strain 8081 and *Escherichia coli* strain K12 were used as an external group. *genomes that have clustered outside the clade of their respective species. (**c**) Venn diagram highlighting the unique, flexible, and core genes for the genomes present in the clade of *S. liquefaciens* (SlATCC27592, SlFDAARGOS, and SlHUMV21), including *S. proteamaculans* 568 (Sp568). In white, the number of unique single copy genes is highlighted, and the unique genes in multiple copies are in blue. For the genomes of SlFG3 and Sp568 a more detailed analysis was established, classifying the unique genes present in the chromosomal or plasmid units. (**d**) Bidirectional best hit analysis (BBH) between the genes that make up the SlFG3 genome in relation to the other genomes of *S. liquefaciens* and Sp568, using RAST. Colors range from dark blue (100%) to light green (70%), representing the degree of conservation of the sequences. The red and green bars identify putative (intact or questionable) phage regions according to analysis established by the PHAST program. (**e**) Comparative analysis of SlFG3 genes relative to the genomes of SlATCC27592, SlFDAARGOS, SlHUMV21, and Sp568 according to the functional classifications of RAST. Categories: 1 - Amino Acids and Derivatives (531); 2 - Carbohydrates (693); 3 - Cell Division and Cell Cycle (42); 4 - Cell Wall and Capsule (193); 5 - Cofactors, Vitamins, Prosthetic Groups, Pigments (292); 6 - DNA Protection and Metabolism (128); 7 - Dormancy and Sporulation (3); 8 - Fatty Acids, Lipids, and Isoprenoids (162); 9 - Iron Acquisition and Metabolism (110); 10 - Membrane Transport (183); 11 - Metabolism of Aromatic Compounds (95); 12 - Miscellaneous (50); 13 - Motility and Chemotaxis (86); 14 - Nitrogen Metabolism (44); 15 - Nucleosides and Nucleotides (149); 16 - Phages, Prophages, Transposable Elements, Plasmids (148); 17 - Phosphorus Metabolism (73); 18 - Photosynthesis (0); 19 - Potassium Metabolism (37); 20 - Protein Metabolism (295); 21 - Regulation and Cell Signaling (168); 22 - Respiration (171); 23 - RNA Metabolism (252); 24 - Secondary Metabolism (8); 25 - Stress Response (183); 26 - Sulfur Metabolism (68); 27 - Virulence, Disease and Defense (126). Six functional subcategories were highlighted by variation in the number of annotated genes and will be discussed throughout the text (DNA repair, DNA phosphorothioation (PT), chloroaromatic degradation, catechol β-ketoadipate, and phages and prophages).

Comparing the genome of SlFG3 with 33 other *Serratia* genomes, SlFG3 showed 311 unique genes (Fig. 2a). The core genome of this dataset contained 431 gene families (Supplementary Table 3). Phylogenetic analysis grouped all strains of *S. liquefaciens* into the same clade, presenting high bootstrap values (Fig. 2b). The *S. liquefaciens* strains are closer to *S. phymuthica*, and these two well-defined clades are clearly separate from all other genomes investigated. Given its position in the phylogenetic hierarchy, the clade comprising *S. fonticola* and *S. multitudinisentens* RB25 may be more closely related to the common ancestor of all *Serratia* species than the other clades.

### *Serratia liquefaciens* genome comparison

All of the following descriptions are based on comparison of the SlFG3 genome with the other four genomes that are part of the *S. liquefaciens* clade identified in Fig. 2b. These five genomes share a core of 3,998 orthologous gene families (Fig. 2c), and 317 orthologous gene families are specific to *S. liquefaciens* strains (Supplementary Table 4).

The two strains isolated from plants (SlFG3 and Sp568) have approximately twice as many specific gene families as any other *S. liquefaciens* strain (Fig. 2c). In Sp568, 445 unique single copy genes are present on the chromosome, while 56 are plasmid-borne (Supplementary Table 5). Regarding the unique multiple copy genes, 66 are present on the chromosome and only two on the plasmid (Supplementary Table 5). Concerning SlFG3, 411 unique single copy genes are present on the chromosome, whereas 61 are on the plasmid pFG3A and 44 on pFG3B (Supplementary Table 6). In addition, in SlFG3, 68 unique genes in multiple copies are inserted into the chromosome and encode mostly phage proteins, while another 53 unique genes in multiple copies are inserted into both plasmids, and these encode mostly for Insertion Sequences transposases (Supplementary Table 6). Only 2 genes (0.8%) of the plasmids that code for hypothetical proteins are unique to SlFG3 (Supplementary Table 2).

### Anomalous regions

Comparison of the SlFG3 genome with the other strains present in the *S. liquefaciens* clade revealed the existence of genomic islands numbered from 1 to 18 (Fig. 2d and Supplementary Table 7). There is a tRNA gene flanking five of the islands, and genes that encode integrases were found in all of them. Four islands have genes that code for toxin and antitoxin systems, presumably involved with the maintenance of each island. Fourteen islands have at least one gene associated with DNA repair mechanisms. Islands 3 and 7 have one copy each of the *recT* gene, and islands 12, 15, and 16 have one copy each of *radC*. These genes are discussed later on. Genes associated with DNA phosphorothioation (*dndBCDE* and *dptFGH*) were identified in island 15 (Fig. 2d).

### Differences in gene repertoire between SlFG3 and the other *S. liquefaciens* genomes

We now present results related to three functional categories: Mobile genetic elements; degradation of organic solvents and aromatic compounds; and DNA protection. These are the categories in which the gene repertoire differences between SlFG3 and the other four *S. liquefaciens* genomes were most striking.

### Mobile genetic elements

The category that presented the greatest difference in the number of genes with respect to the other four genomes was that associated with mobile genetic elements, with emphasis on the massive presence of genes associated with prophages. While SlFG3 has in its genome 148 genes annotated with this function, all four other genomes have between 15 and 38 genes associated with the same function, indicating that SlFG3 was more susceptible to temperate phage infections.

### Degradation of organic solvents and aromatic compounds

**Figure 3:**
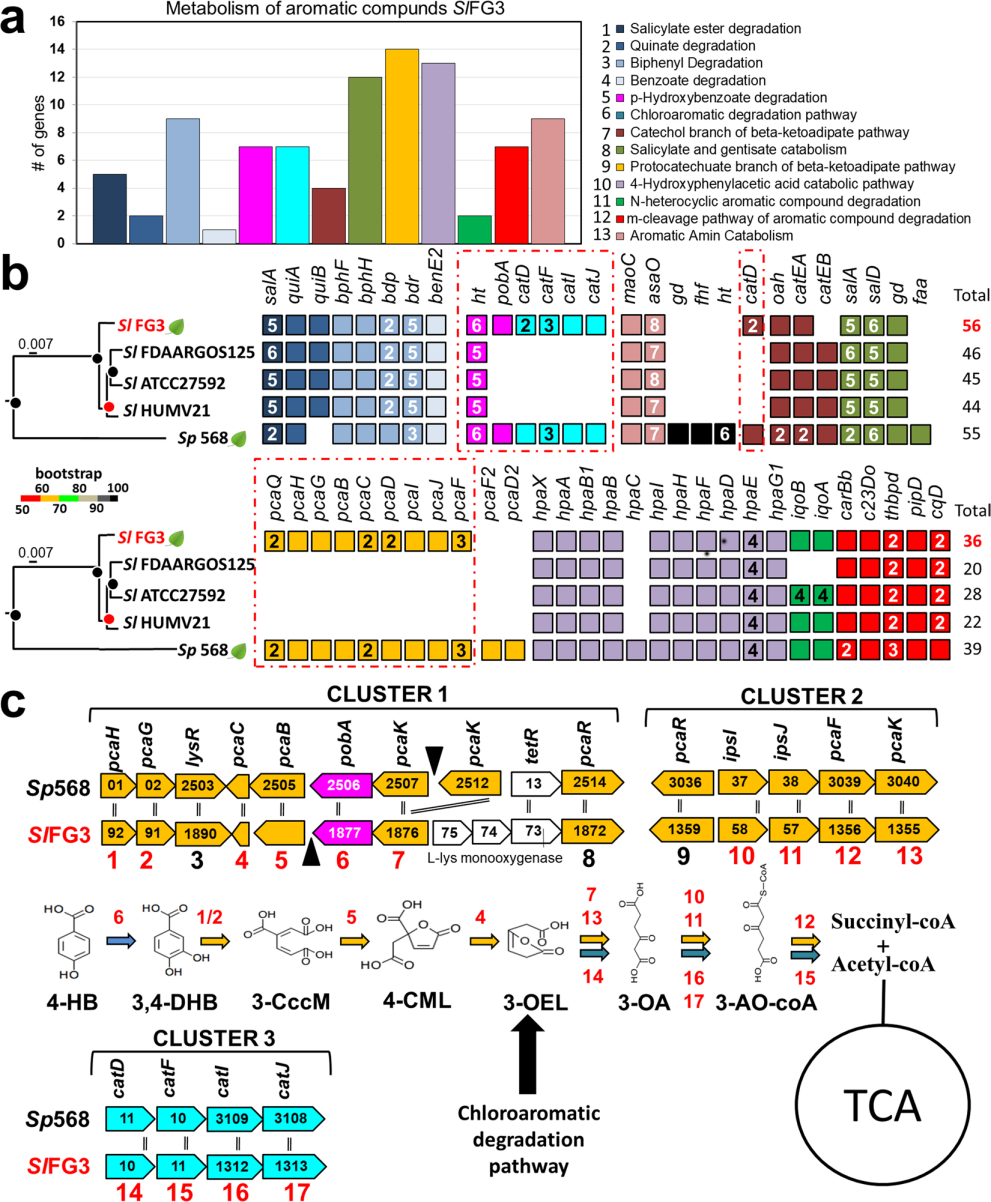
Analysis of the genes and metabolic pathways associated with metabolism of phenolic compounds in the genome of SlFG3. (**a**) Distribution of the SlFG3 genes in the metabolic pathway subcategories associated with degradation of phenolic compounds. (**b**) Comparison of the genes quantitatively presented in A in relation to the genomes of the other species present in the clade of *S. liquefaciens*. The *cat* and *pca* genes involved, respectively, with the chloroaromatic degradation pathway, and catechol B-ketoadipate pathway are highlighted: these are present exclusively in plant-associated genomes (SlFG3 and Spro568). (**c**) Analysis of the syntenia and function of each of these genes in the metabolism of 4-hydroxybenzoate (4-HB) and chloroaromatic compounds. 3,4-DHB-3,4-dihydroxybenzoate (protochatecuate); 3-cycloM-3-carboxy-cis, cis-muconate; 4-CML-4-carboxymuconolactone; 3-OEL-3-oxoadipate enol lactone; 3-AO-3-oxoadipate; 3-AO-coA-3-oxoadipyl-CoA; TCA - tricarboxylic acid cycle.

A total of 95 genes associated with degradation of aromatic compounds were identified in the SlFG3 genome and grouped into 13 metabolic subcategories (Fig. 3a). Although most of the genes are present in all five genomes, for three of these subcategories, genes that participate in the degradation pathway of p-hydroxibenzoate such as *pobA* (pink) via degradation of protochatechuate (orange) and degradation pathway of chloroaromatics (aquamarine) were identified only in the genomes of SlFG3 and Sp568 (Fig. 3b). Analysis of the localization of these genes in both the genomes has shown that they are inserted in three syntenic clusters (Fig. 3c).

### DNA protection

**Figure 4:**
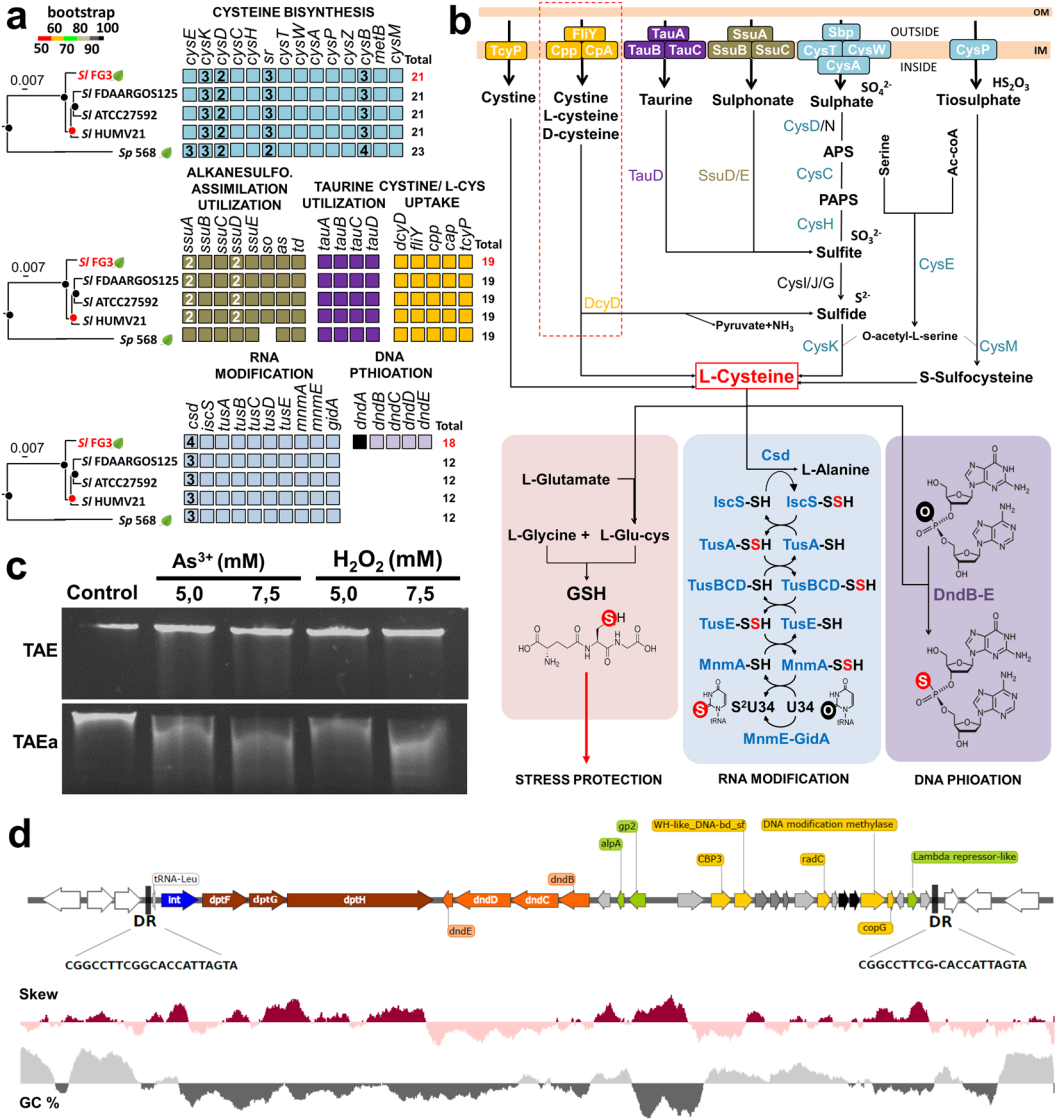
Comparative analysis of sulfur metabolism pathways. (**a**) Presence and absence of genes involved in cysteine biosynthesis and metabolism involving the five genomes present in the clade of *S. liquefaciens*. Squares represent the presence of the respective investigated genes, which may be present in single copy, or as multiple copies according to the number within these squares. (**b**) Integrated metabolism of cysteine biosynthetic and metabolic pathways that culminate in three important routes: biosynthesis of glutathione that, in turn, would be associated with protection against oxidative stress; RNA modification, as an additional protection of the addition of a sulfur molecule at position 34; and DNA protection against phosphorothioation–mediated events. (**c**) Functional analysis of DNA protection of SlFG3 by phosphothioation events when submitted to stress conditions (For details see Supplementary Fig. S1). (**d**) Analysis of the possible region associated with horizontal gene tranfer where the genes associated with DNA PT (*dndBCDE*) in the genome of SlFG3 are inserted. DR - direct repeats; tRNALeu – Leucine tRNA gene; *DptFGH* - genes involved with DNA restriction and modification mediated by PT DNA.

In this functional category, two subcategories stand out in the genome of SlFG3: DNA phosphorothioation (DNA PT), and DNA repair, both involving genes that may justify the adaptation of SlFG3 to extreme conditions. DNA phosphorothioation is related to the protection of DNA against oxidatively induced damage, by changing the oxygen of the phosphate group of DNA to a sulfur moiety, thus conferring greater stability and protection against nucleases. It was evidenced that all species of *S. liquefaciens* have all the genes involved in transport and cysteine synthesis (Fig. 4a). Among these, we found genes involved in direct cysteine uptake of the media, mediated by the TcyP and FliY-Cpp-Cpa transporters (also involved in D-cysteine detoxification in the presence of the *dcyD* gene), or mediated by taurine transport and metabolism (*sbp*, *cysTWADCHEK*), and thiosulfate (*cysPEM*) (Fig. 4b). Once within the bacterium, this cysteine may be a precursor of glutathione synthesis for modifications to carrier RNA, or for DNA phosphorothioation (Fig. 4b).

To verify the role of the genes related to PT DNA, SlFG3 was exposed to arsenic and H_2_O_2_. Our results revealed that the DNA extracted from cells thus treated were fragmented once in contact with activated TAE buffer. This effect was observed in DNA electrophoresis, but not observed when the DNA was pretreated with non-activated TAE buffer (Fig. 4c – for details see Supplementary Fig. S1). Interestingly, it has also been proven that the higher the concentrations of metalloid and oxidizing agents, the greater the degree of DNA fragmentation when treated with activated TAE^14,15^. Therefore, these results strongly support the functionality of the *dnd* gene cluster by the inclusion of sulfur in the DNA structure.

Although all five strains investigated for these processes have the same genes for modification of the carrier RNA (*csd*, *iscS*, *tusABCDE*, *mnmAE*, and *gidA*), only SlFG3 has genes associated with DNA PT, and this may be explained by the fact that DNA PT genes (*dndBCDE*) are part of island number 15. This island presents genomic signatures commonly found in genomic islands, such as insertion sites associated with tRNAs and tyrosine recombinase integrase, and the presence of direct repeats on both edges of the island (CGGCCTCGGCACCATTAGTA) (Fig. 4d). In addition, genes encoding a toxin and antitoxin system, and a copy of *radC*, and restriction and modulation system (*dptFGH*) that is directly associated with DNA PT mechanisms were also identified. According to data available in the literature, these genes are classically transferred together with *dnd* genes^15,16^ and are commonly associated with extremophilic microorganisms^17^.

### DNA repair

**Figure 5:**
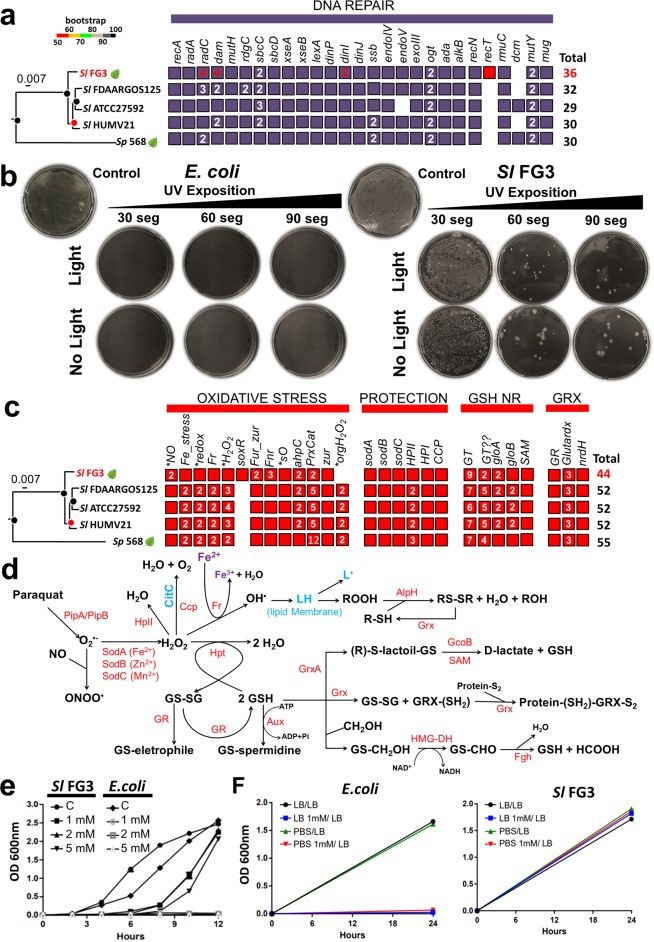
Analysis of oxidative stress metabolism and DNA repair in the genome of SlFG3. (**a**) Comparison involving the presence and absence of genes involved in DNA repair, involving the five genomes present in the clade of *S. liquefaciens*. In red is highlighted the exclusive *recT* gene of SlFG3 and an additional copy of *dam*, *dinI* and *radC* genes, the latter present in the HGT region where the DNA PT genes are inserted (Fig. 3). (**b**) Tolerance of SlFG3 exposed for 30, 60, and 90 seconds of UV and incubated in the presence and absence of light, compared to *E. coli*. (**c**) Comparison involving the presence and absence of genes involved in oxidative stress adaptation (red), enzymes (orange) and GSH biosynthesis and recycling (brown), involving the five genomes present in the *S. liquefaciens* clade. (**d**) Integrative metabolism of pathways associated with oxidative stress in the SlFG3 genome. It is possible to observe a complete repertoire of genes involved in protection against cell damage induced by reactive oxygen species (ROS). (**e**) Functional analysis of the ability of SlFG3 to protect against H_2_O_2_-induced damage in comparison to *E. coli* in the absence and increasing presence of 1, 2 and 5 mM H_2_O_2_ (f) Evaluation of resistance to acute exposure to 1 mM H_2_O_2_ SlFG3 compared to *E. coli*.

With respect to the subcategory of genes associated with DNA repair, 36 genes were identified (Fig. 5a). To verify the potential of these genes and DNA repair mechanisms, SlFG3 was subjected to UV light exposure. It was possible to observe that when compared to *E. coli* submitted to the same conditions and used as an experimental control, SlFG3 appeared to be very tolerant to UV light-induced damage since SlFG3 showed growth colonies even after 90 s (~10.44 mJ/cm^2^) of exposure, in the presence or absence of light during incubation, while 30 s were sufficient to impair *E. coli* growth (Fig. 5b).

### Response and adaptation to redox processes

A total of 44 genes related to oxidative stress pathways (including protection and GSH and GRX systems) were identified in S1FG3 (Fig. 5c, details see Supplementary Results), which relate to each other in an intricate metabolic network (Fig. 5d). Aiming to validate this redox potential, SlFG3 and *E. coli* were challenged in the presence of different concentrations of hydrogen peroxide. It was observed that SlFG3 was able to grow at concentrations of 1, 2, and 5 mM H_2_O_2_ compared to *E. coli* (Fig. 5e). The same effect was observed when both strains were exposed to 1 mM H_2_O_2_ in PBS buffer for 30 min. After being incubated in LB broth, only SlFG3 cells were able to resume growth (Fig. 5f).

### Iron acquisition and metabolism

A total of 110 genes were identified in the genome of SlFG3 for this category. Of these, 49 were associated with siderophore biosynthesis, secretion (enterobactin and aerobactin) and iron internalization, and the other 61 were involved with metal metabolism (Supplementary Fig. S2). The enterobactin biosynthetic genes are inserted in a cluster (*entCEBA*) arranged downstream of *ybdZ* in tandem with the gene encoding the siderophore carrier protein (*entS*), a gene encoding enterobactin esterase (*fes*), the *entF* gene (synthesis component), and the transport system of this compound (*fepAGDCB*), to be secreted into the environment. As for genes involved in aerobactin synthesis, the *iucABCD* gene cluster is located in tandem and upstream of the gene coding for the receptor of this siderophore (*iutA*). Besides the genes related to siderophore biosynthesis, genes encoding the ABC transporter complex associated with aerobactin internalization were also identified (Supplementary Fig. S2) Regarding proteins involved in the metabolism of this metal, the *fur* (ferric uptake regulator) gene, two copies of the *dps* related to iron storage inside the cell, as well as proteins that present Fe-S-based prosthetic groups as the ones coded by *sufEDBCA* were identified

To validate the synthesis of these siderophores, extraction and separation of these compounds by TLC was carried out (Supplementary Fig. S3). It was possible to verify that SlFG3 produces two siderophores, one with chemical characteristics of catecholamates, with a purple color after development, and the other with hydroxamate characteristics, due to the yellowish coloration after development. These are possibly siderophores produced by the gene clusters described above.

### Resistance to other metals

A total of 11 genes associated with copper homeostasis were found in SlFG3, which also has a gene cluster related to resistance to cobalt, zinc and cadmium, including the respective two-component system (*cusSR*). We also found a copy of *arsR* (transcriptional regulator), *arsB* (efflux pump), and two copies of *arsC* (arsenate reductase) related to arsenic resistance, which validate the results previously described by Caneschi, *et al*.^10^ that experimentally shown that SlFG3 is able to high concentrations of this metalloid.

### Cellulose production

**Figure 6:**
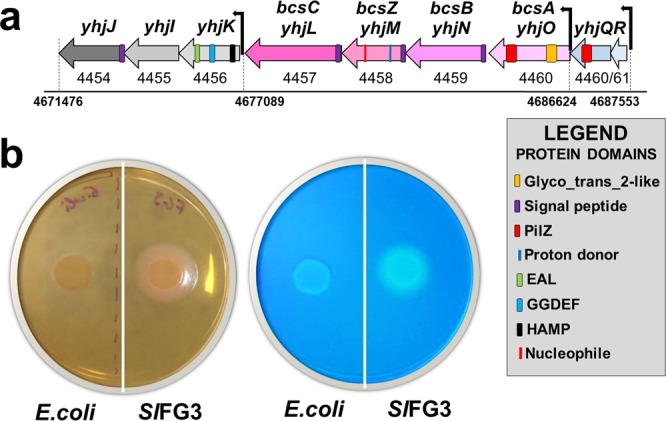
Structural and functional analysis of SlFG3 gene cluster related to cellulose production. (**a**) Analysis of the composition of a gene cluster associated with cellulose synthesis. The clustering involves nine genes: 2 regulatory (blue), 4 associated with export and cellulose synthesis (pink), and 3 associated with quorum sensing and modulation of cellulose synthesis responses (green). In each of the genes that are part of the cluster was verified the presence of functional domains. (**b**) Plates demonstrating the production of cellulose by SlFG3 when compared to *E. coli*, revealed by the bright blue methods of Coomassie (above) and calcofluor (below).

Cellulose synthesis-related genes are arranged in three distinct tandem operons in all *S. liquefaciens* genomes (Fig. 6a). The first consists of the regulatory *yhjQR*, followed by the biosynthetic *yhjONML* and signal transduction *yhjKIJ* operons^18^. In addition, each operon was analyzed for the presence of functional domains allowing us to determine a route of synthesis and secretion. An experimental assay showed that SlFG3 is able to produce cellulose in culture medium (Fig. 6b) in comparison to *E.coli* (a negative control to cellulose production).

### Secondary metabolite biosynthesis gene clusters

**Figure 7:**
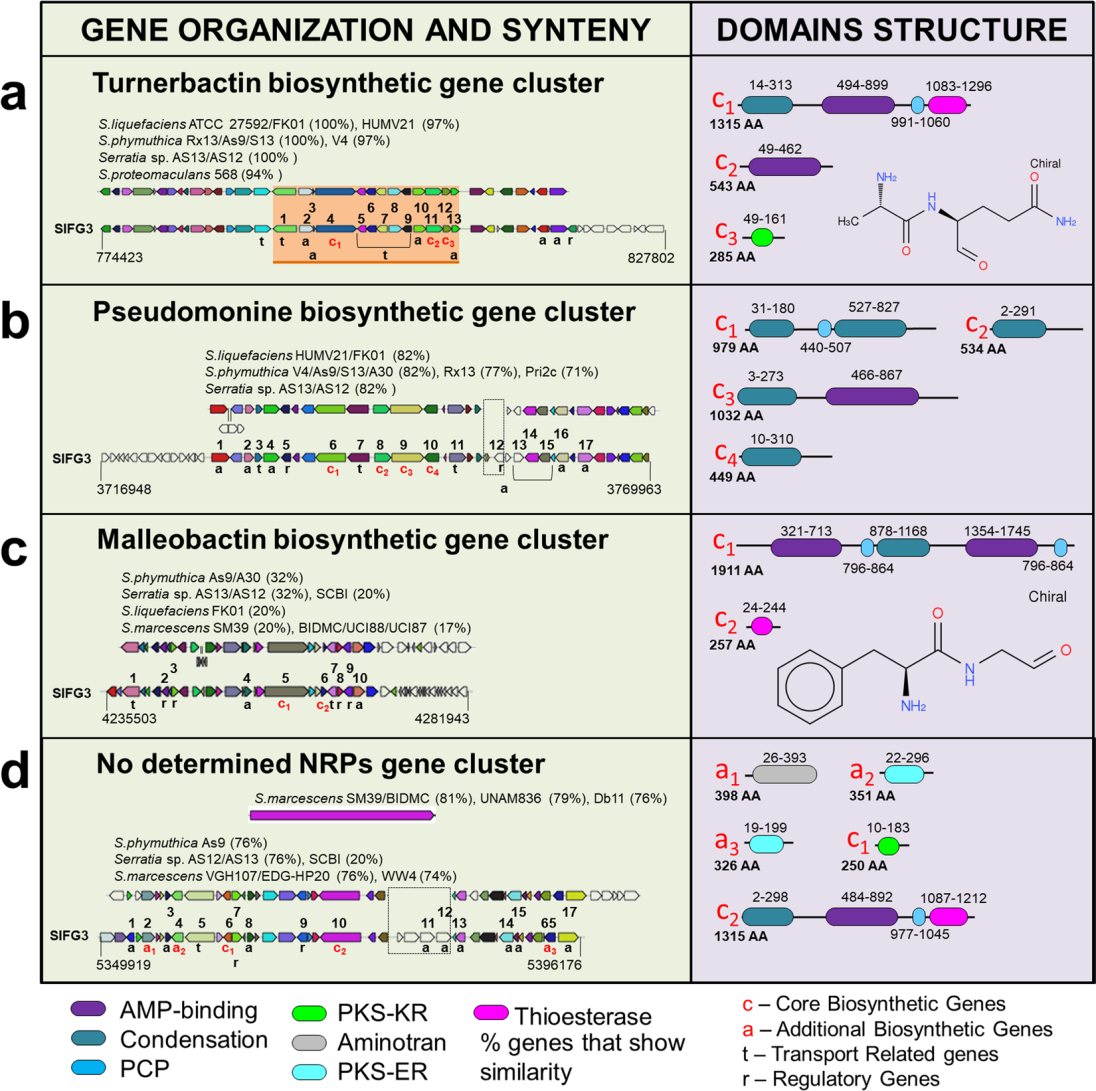
Analysis of the composition of gene clusters associated with synthesis of secondary metabolites in the genome of SlFG3. Of the 35 clusters identified by AntiSmash tool, four were highlighted - among them: biosynthesis of turmebactin (**a**), pseudomonine (**b**), malleobactin (**c**) and a cluster associated with an unidentified NRP (**d**). In green background, the gene clusters that are part of the biosynthetic core (**c**) and accessory genes (**a**) involved in the synthesis of these compounds are highlighted. The legend below the figure highlights the classification of these genes and the characterization of functional domains. It is possible to observe that all four clusters were identified in other species from *Serratia* genus, sometimes maintaining a high degree of conservation, as is the case of the cluster of turnebactin (**a**), sometimes with a low degree of maintenance of the genus composition, as is the case for the malleobactin synthesis cluster (**c**).

SlFG3 has 35 gene clusters potentially associated with biosynthesis of secondary metabolites. For instance, four gene clusters are associated with the synthesis of NRPs: Turnerbactin, Pseudomonine, Malleobactin, and a no determined NRPs gene cluster, all shared with other *Serratia* species (Fig. 7 and Supplementary Table 8).

### Colonization and adaptation to plant tissue

SlFG3 present a cluster of genes associated with the induction of hyperadherence (*yidE-16hspB-16hspA-yidR-yidQ*), described as fundamental for some microorganisms to colonize plants and seeds^19^. Moreover, it was found that genes in these genomes were associated with tolerance to E2 colicin (*creBCD, creA*), and genes related to colicin V and bacteriocin biosynthesis (*R1-dedA-R3-R4-R5-dedD-colV-purF*). In addition, 32 other genes were annotated with multidrug resistance functions. SlFG3 presents genes encoding acetolactate synthase (*als*) and α-acetolactate decarboxylase (*aldC*), involved with the conversion of pyruvate to acetoin, which can be converted to 2,3-butanediol or converted to acetaldehyde. Nevertheless, although, SlFG3 possesses a gene involved in the synthesis of 2,3-butanediol, it lacks the *acoR* involved in synthesis of acetoin.

### Secretion systems, motility, and chemotaxis

In the *SfFG3* genome, genes associated with synthesis and regulation of types I, II (gsp), III, VI and VIII secretion system were not found. However, we identified genes for the type II CBSS-562.2.peg.633 system. We identified 14 genes related to type IV, 10 genes related to type V, 19 genes related to type VII, 2 genes related to bacterial signal recognition particle (SRP), and four genes related to twin-arginine translocation, all representing a complete repertoire of genes related to flagellum structure and chemotactic modulation.

### Analysis of additional metabolic pathways and cellular processes

In addition to the categories described above, other metabolic pathways and cellular processes were identified in the genome of SlFG3 and may be related to its adaptive capacity (Supplementary Results). These pathways include central carbohydrate metabolism, respiration, invasion and intracellular resistance, response and adaptation to redox processes, and other stress adaptations, that may also contribute to their survival against biotic and abiotic factors related to IQ region.

## Discussion

### Genetic repertoire of SlG3 associated with adaptation to abiotic stresses

Although many studies have revealed the adaptive characteristics of plants to adverse conditions imposed by ferruginous fields, there is a lack of knowledge regarding the genetic adaptive potential of bacteria living in such environments^20^. By sequencing the SlFG3 genome, it was possible to identify a series of genes associated with metabolism of lead, cadmium, zinc and arsenic. Some of these corroborate the results empirically shown by Caneschi, *et al*.^10^ in preliminary work regarding the ability of SlFG3 to tolerate high concentrations of arsenic in culture medium. However, it was in terms of iron metabolism that SlFG3 stands out. Two gene clusters associated with the synthesis of siderophores enterobactin and aerobactin were found. Functional analysis showed that two siderophores with similar chemical characteristics (catechol and hydroxamate respectively) can be synthesized by SlFG3. These siderphores are known to be internalized by specific transporters and stored by specialized proteins such as Dps^21,22^.

Another SlFG3 capability determined here is the production of cellulose, which can promote adhesion to rhizospheric^23^ or phyloplanic^24^ tissue, stabilizing bacterial colonization of the plant surface^25^, and reducing the loss of water by dehydration^26^. Additionally, the presence of a functional cellulose biosynthetic cluster could favor the maintenance of floral structures, increasing the chance of visitation by possible pollinators, thus contributing to the reproductive cycle of the plant species^27,28^.

However, the most intriguing genes in the SlFG3 genome are related to DNA repair and protection. Genes with DNA repair function were identified in 9 of 18 genomic islands, with islands 3 and 7 showing additional copies of the *recT* gene, and islands 12, 15 and 16 having additional copies of the *radC* gene. Functionally, *recT* encodes a single-stranded DNA (ssDNA)-annealing and strand invasion protein related to homologous phage recombination^29^, which could justify phage insertions in the genome of SlFG3. RadC is involved with prokaryotic repair of DNA damage after UV and X-ray irradiation^30^. SlFG3 possesses an additional copy of the *dam* gene that codes for a Dam-methylase whose function is to identify the parent DNA strand during DNA repair processes, and *dinI*, which encodes a DNA damage-inducible protein whose function is to inhibit RecA during SOS responses related to homologous recombination^31^. Although 16 of the 18 islands identified had some gene that could confer genetic benefits to the genome of SlFG3, it is important to highlight island 15. It has in its composition a gene cluster associated with DNA phosphorothioation (*dndBCDE*) arranged downstream of a cluster coding for endonucleases (*dptFGH*)^32^ and upstream of other genes with functions associated with DNA binding.

Analysis of the flanks of this genomic island allowed the identification of direct repeats^33^, one of which is positioned upstream of a gene encoding tRNA-Leu, whose 3’ position may have acted as an insertion site of an immediately localized integrase downstream, which are well-known lateral gene transfer signatures^34^. Regions containing genes associated with DNA PT are described with side transfer products in other genomes, including other Enterobacteriaceae^16^. DNA phosphorothioation genes are responsible for a molecular mechanism that exchanges an oxygen for a sulfur atom bound to the phosphate group^35^, protecting DNA against damage by oxidative stress or endonucleases^36^. Indirect analysis of the functionality of these genes revealed the potential of DNA protection against different concentrations of arsenic and H_2_O_2_.

Corroborating this increase in DNA protection by phosphorothioation, genes of *tusABCDE* and *mnm* associated with tRNA protection were identified^37^. The proteins encoded by them promote the replacement of the oxygen atom of uracil at position 34 (U34) of the tRNA by a sulfur (S2-U34), conferring to this biomolecule increased thermal stability that translates into protection against non-specific degradation by bases and nucleases^38^.

SlFG3 also possess a diversified repertoire of metabolic pathways capable of meeting the need for production of a sulfur donor substrate. We showed here that SlFG3 has transport and metabolism systems involving taurine, sulfonate, sulfate, thiosulfate, cysteine, L- and D-cysteine^39^, all culminating in intracellular L-cysteine that could then act as a sulfur donor for the mechanisms of thioation of DNA and tRNA, and substrate for the synthesis of glutathione (GSH).

Considering this potential for generation of GSH, it was possible to verify that SlFG3 also has a broad repertoire of genes related to oxidative metabolism. Our experimental assays showed that S1FG3 was able to multiply even under oxidizing stress conditions This may be associated with the previously described DNA modification events, but also with a high capacity to detoxify this compound, as observed previously^10^.

**Figure 8:**
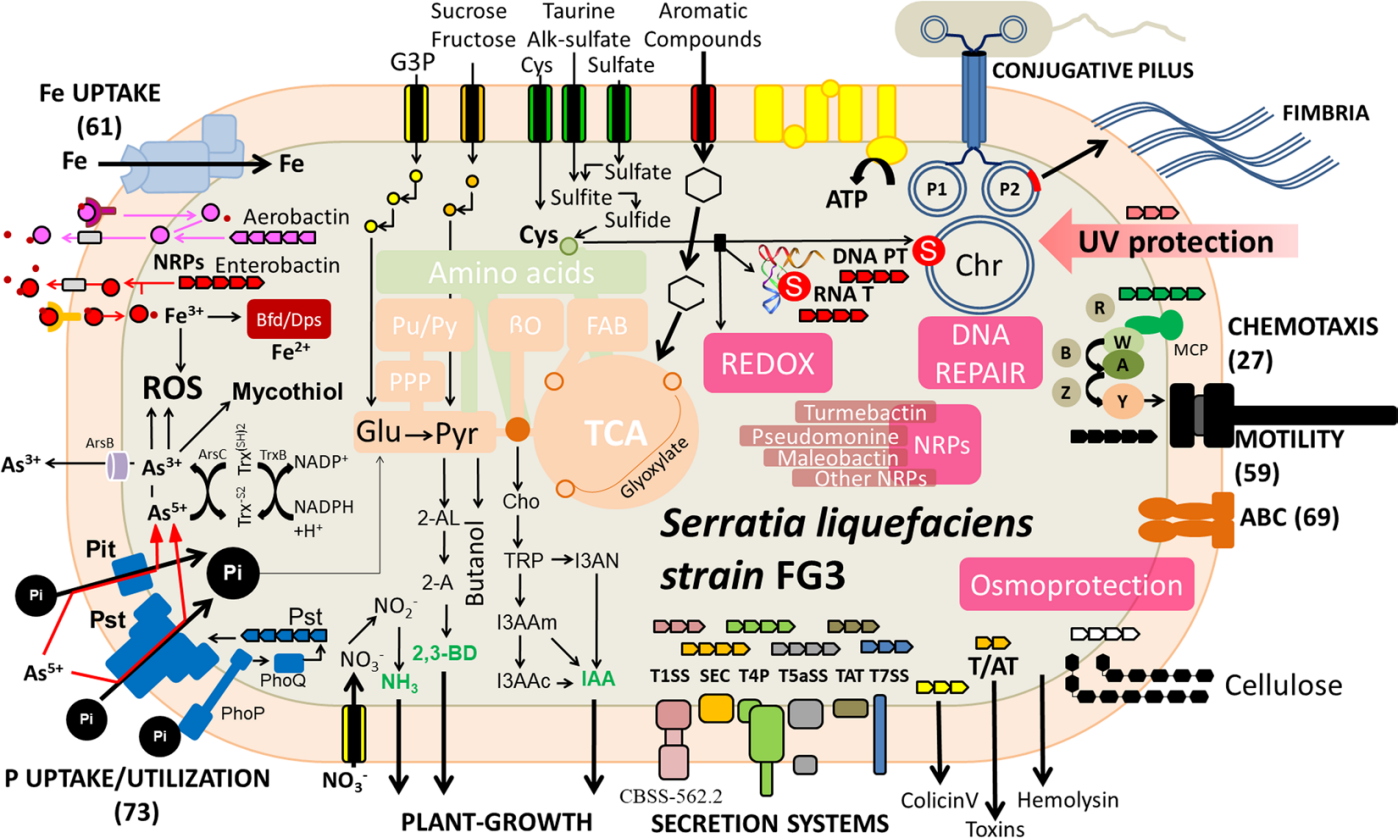
Schematic representation of the integrated metabolism of SlFG3. The arrows determine the flow of metabolic information. The colors of these arrows are differentiated only to allow a better understanding of the processes involved. The tandem arrows identify the presence of gene clusters associated with their respective characterized functions. The numbers in parentheses determine the total number of genes associated with the respective function presented.

Together, the additional copies of genes related to DNA repair, the presence of genes involved with tRNA protection, the acquisition of functional genes associated with DNA PT, a diversified metabolism of sulfur and oxidative damage, and a diverse repertoire of genes associated with metabolism of different metals can explain the successful growth of SlFG3 even under highly damaging environmental conditions in which it was isolated (Fig. 8).

### Genetic repertoire of SlFG3 associated with metalophyte plant colonization

With respect to the presence of genes that could justify the interaction with metallophyte plants, SlFG3 also presents in its genome a diverse repertoire of genes and functions. Regarding energy metabolism, SlFG3 has genes involved in the degradation of a number of carbohydrates (Supplementary Results), many of them produced exclusively by plants^40^. Once converted to pyruvate, it could be metabolized under aerobic and anaerobic conditions, which corroborates the fact that SlFG3 presents a series of electron acceptors and donors, making the generation of energy highly versatile (Supplementary Results).

SlFG3 also has complete metabolic pathways associated with plant growth promotion, as might be expected from its natural habitat. Among these pathways are those involved with Indole-3-acetic acid (IAA), acetoin, and butanediol synthesis, as previously also found in the genome of *Serratia marcescens* RSC-14^41^. These characteristics highlight SlFG3 as an important bacterium that can be used to promote plant growth especially in proposals involving regeneration of areas contaminated by metals. This condition is well evidenced in the IQ since it has suffered intense anthropic action due to mineral extraction activities.

Although the pathways of nitrogen metabolism and phytonutrient synthesis are important to correlate their adaptive profile to the host plant, the most interesting pathways present in SlFG3 are related to the degradation capacity of phenolic compounds. These compounds are produced directly or indirectly by plants, and have been described as molecules against infections^42^. In SlFG3, we also identified the complete degradation pathways of 4-hydroxybenzoate, and the protocatechuate branch of beta-ketoadipates and chloroaromatics, not only found in Sp568, but also isolated from plants. The genes that participate in these pathways are located in three syntenic clusters that could be associated as representing detoxification mechanisms of these compounds that would culminate in acetyl-CoA and succinyl-CoA synthesis and which are then used as alternative sources of carbon^43^.

The absence of some of the secretion systems that are classically found in pathogenic organisms, such as type II, III, VI, and VIII^44^, may be due to the symbiotic nature of interactions between SlFG3 and its plant host. Despite this absence, SlFG3 does have secretion systems involved with adhesion and colonization of host tissues, such as: type II CBSS-562.2.peg.633, type IV, type V, and two-partner secretion pathway (TPS). Additionally, the secretion system type VII, capable of synthesizing type I pili and genes related to induction of hyperadhesion, which allows interaction and colonization of plants and seeds^19^, were also identified (Fig. 8).

### Genetic repertoire of SlFG3 associated with adaptation to the presence of other organisms

In addition to the adaptive resources under abiotic conditions and survival in contact with host tissue, SlFG3 has genes that help it avoid competition with other organisms present in the same niche, including 35 gene clusters associated with synthesis of potential secondary metabolites. Among them, we identified clusters involved in polysaccharide and O-antigen synthesis, and clusters associated with the synthesis of marinacarboline and taxillaid metabolites, which have antimalarial activity^45,46^. However, it was with respect to siderophore biosynthesis that SlFG3 stood out. Six gene clusters were identified related to the synthesis of these compounds, hypothetically capable of synthesizing turnerbactin^47^, pseudomonin^48^, malleobactin^49^, a siderophore derived from an arylpolyene^50^, enterobactin^49^, and aerobactin^51^, as previously described. Siderophores may contribute to plant growth^52,53^, or may be associated with competition among organisms of the same niche since the efficiency of iron uptake by a competent microorganism could induce bacteriostasis or bacterial death of other microorganisms^54^. Considering that this competition may reduce the incidence of phytopathogenic organisms^55^, and that plant immunity can be activated by the presence of microbial siderophores^56,57^, SlFG3 could act to promote the protection and indirect growth of plants controlling populations of pathogenic microbiota.

Furthermore, a series of genes associated with the synthesis and secretion of colicins and of RTX toxins were identified, which are related to the presence of a type I secretory system^58^ (Fig. 8).

The results presented here showed that SlFG3 is a highly versatile bacterium with diverse adaptive mechanisms enabling it to survive in extreme conditions, featuring clusters associated with DNA PT and cellulose biosynthesis for the first time has been described functionally to *S. liquefaciens*. All of these characteristics highlight the importance of exploratory research in environments with lack of studies from a molecular perspective, as is the case for IQ. In an environment that is suffering from high anthropic activity, these results emphasize the importance of preserving this area, not only due to the high degree of plant endemism already described, but also due to the diversity and genetic potential of microorganisms, such as SlFG3.

## Materials and Methods

### Bacterial isolation and culture conditions

*Serratia liquefaciens* strain FG3 (SlFG3) was isolated, identified and grown under conditions which were previously established^10^.

### Genomic DNA isolation, sequencing, and genome assembly

The total genome of SlFG3 was obtained from 24 h cultures in 50 ml LB broth (10 g/l NaCl, 10 g/l Peptone, 5 g/l Yeast Extract, pH 7.0) using the DNeasy PowerLyzer Microbial Kit ™ (Qiagen, Hilden, Germany) following the manufacturer’s instructions. The material was sent to Duke University (USA) and sequenced on a single SMRT cell on a PacBio™ RS II platform (Pacific Bioscences, California, USA). A total of 188,003 reads were generated, with average length of 13,074 bp. The genome was assembled using HGAP2 protocol. Chromosome and plasmids sequences were deposited with NCBI under accession numbers CP033893-CP033895, Bioproject PRJNA505252, and Biosample SAMN10413339.

### Genome annotation and comparison

By joining up our SlFG3 strain with other thirty-three *Serratia* genomes obtained from NCBI (Supplementary Table 1), sequenced by November 2016, and also two outgroup species (*E. coli* K12 and *Yersinia enterocolitica* 8081), we created a comparison framework using Orthologsorter^59^. The data is available at http://jau.facom.ufms.br/serratia. Orthologsorter consists in a customized web search tool for finding specific protein families, phylogenetic species trees and lateral gene transfer inferences. All genomes used in this framework have been re-annotated with Prokka program^60^.

### Phylogenomic analyses

Two phylogenomic analyses were performed. The first one using concatenated protein families containing exactly one protein gene from each in-group genome, admitting zero or one gene from each outgroup genome. A multiple alignment was made using MUSCLE^61^ and refined by using GBlocks^62^. The final whole alignment was passed to RAxML^63^, who built the unrooted phylogenetic tree using PROTCATJTT substitution model, with rapid bootstrapping (100 replicates) and subsequent Maximum Likelihood search. The second one has been made using Mumi^64^ having as input all chromosome sequences.

### Functional and metabolic categorization of genes

Four genomes of the genus *S. liquefaciens* and the genome of *Serratia proteamaculans* 568 were compared to each other using RAST and Ortologsorter^59^ to determine families of orthologous proteins (http://jau.facom.ufms.br/4liquefaciensplusproteomaculans/). Metabolic pathways were investigated from RAST itself or using KEGG^65^.

### Phage insertion analysis

Phage insertion into the genome was verified using the PHAST program (http://phast.wishartlab.com)^66^.

### Biosynthesis and analysis of secondary metabolites

The program AntiSmash (bacterial version) was used to predict possible genes associated with biosynthesis of secondary metabolites^67^.

### Ultraviolet light (UV) tolerance

SlFG3 and *E. coli* were grown in LB broth at 28 ± 2 °C with mechanical agitation of 150 rpm until reaching optical density (OD) approximately equal to 1 (10^8^ cells/ml). One hundred microliters of the bacterial suspension were spread on LB agar plates and exposed to 0, 30, 60, and 90 seconds of UV light, equivalent to 0, 3.48, 6.96, and 10.44 mJ/cm2, respectively. The plates were then incubated at 28 ± 2 °C for 24 hours in the presence and absence of light, and colony growth was evaluated for each exposure time. Experiments were performed in triplicate.

### DNA phosphorothioation

SlFG3 and *E. coli* were grown in 25 ml of LB broth at 28 ± 2 °C under agitation of 150 rpm until the OD approximately equaled 1 (10^8^ cells/ml). Then, the cells were treated with 5 and 7.5 mM of sodium arsenite and H_2_O_2_ for 40 minutes. Cells were centrifuged at 8,000 × *g* and washed twice with LB medium. Genomic DNA was extracted using the Wizard Genomic DNA Purification Kit ™ (Promega, Wisconsin, USA). The presence of phosphorothioate DNA was identified by the methodology described in Wang, *et al*.^14^.

### Hydrogen peroxide sensitivity

SlFG3 and *E. coli* were incubated in 25 ml LB broth for 12 h at 28 ± 2 °C with shaking at 150 rpm. The cell density was standardized for all isolates as OD equal to 1 (10^8^ cells/ml). Cell suspensions were diluted 1:500 in LB broth and incubated at 28 °C with 150 rpm agitation in the presence of H_2_O_2_ at 1, 2, and 5 mM, and OD was then monitored over time at a wavelength of 600 nm. Cell sensitivity was verified by growing as previously described. The cells were centrifuged at 2000 × g for 5 min and washed with PBS buffer pH 7.2, or LB broth, three times. Then, the cells were exposed to 0 or 1 mM of H_2_O_2_ for 30 min in LB broth or PBS buffer 100 µl of cell suspensions was transferred to 10 ml LB broth for 24 h at 28 ± 2 °C with shaking at 150 rpm. After incubation, the ODs of each treatment were verified at a wavelength of 600 nm. The experiments were conducted in triplicate.

### Analysis of the production of siderophores by thin layer chromatography (TLC)

SlFG3 was grown in 10 ml of M9 medium for 12 hours at 28 ± 2 °C. The cells were removed by centrifugation (8000 × *g*, 10 min). Siderophores were extracted from the supernatant with 10 ml of ethyl acetate, dried and resuspended in 10 μl of 50% aqueous ethanol. Siderophores were then separated on TLC plates (Macherey-Nagel, Düren, Germany), coated with a 0.25 mm layer of silica gel, with methanol/acetonitrile (7:3, v/v). Iron-binding compounds were identified by sputtering the TLC plates with 0.05% ferric chloride in ethanol. The commercial siderophores 2,3-dihydroxybenzoic acid and acetohydroxamic acid (Sigma, Darmstadt, Germany) were used as controls for migration during TLC.

### Analysis of cellulose production

SlFG3 and *E. coli* were grown in 25 ml LB broth for 12 hours at 28 ± 2 °C with shaking at 150 rpm. Five microliters of the bacterial suspension was placed in LB agar without salt (10 g/l Peptone, 5 g/l Yeast Extract, 15 g/l Agar, pH 7.0) supplemented with 40 mg/l Congo Red and 20 mg/l Bright Coomassie Blue^68^. Colonies with salmon tones indicate the production of cellulose. Another 5 µl of bacterial suspension was placed in LB agar supplemented with 0.02% calcofluor. After growth, the fluorescence of colonies was verified under UV light at 365 nm^69,70^. Colonies that reflect light at this wavelength indicate cellulose production. All assays were performed in triplicate.

### Ethical approval

This article does not involve any studies with human participants or animals performed by any of the authors.

## Supplementary information

Supplementary information
