# Supplementary material for: Serratia liquefaciens FG3 isolated from a metallophyte plant sheds light on the evolution and mechanisms of adaptive traits in extreme environments
Source: Sci Rep. 2019 Nov 29;9:18006. doi: 10.1038/s41598-019-54601-4 (PMC6884506; doi:10.1038/s41598-019-54601-4)
Supplement: Supplementary file 1 — Supplementary information [file 41598_2019_54601_MOESM1_ESM.docx]

**Supplementary Information for:**

***Serratia liquefaciens* FG3 isolated from a metallophyte plant sheds light on the evolution and mechanisms of adaptive traits in extreme environments**

Washington Luiz Caneschi^1*^, Angélica Bianchini Sanchez^1*^, Érica Barbosa Felestrino^1*^, Camila Gracyelle de Carvalho Lemes^1^, Isabella Ferreira Cordeiro^1^, Natasha Peixoto Fonseca^1^, Morghana Marina Villa^1^, Izadora Tabuso Vieira^1^, Lauro Angelo Gonçalves de Moraes^1^, Renata de Almeida Barbosa Assis^1^, Flávio Fonseca do Carmo^2^, Luciana Hiromi Yoshino Kamino^2^, Robson Soares Silva^3^, Jesus Aparecido Ferro^4^, Maria Inês Tiraboschi Ferro^4^, Rafael Marini Ferreira^4^, Vera Lúcia Santos^5^, Ubiana de Cássia Mourão Silva^5^, Nalvo Franco Almeida^3^, Alessandro de Mello Varani^4^, Camila Carrião Machado Garcia^1,6^, João Carlos Setubal^7,8^, Leandro Marcio Moreira^1,6^.

^1^Núcleo de Pesquisas em Ciências Biológicas (NUPEB), Universidade Federal de Ouro Preto, Ouro Preto, MG, Brazil; ^2^Instituto Prístino, Belo Horizonte, MG, Brazil; ^3^Faculdade de Computação (FACOM), Universidade Federal do Mato Grosso do Sul, Campo Grande, MS, Brazil; ^4^Faculdade de Ciências Agrárias e Veterinárias de Jaboticabal, UNESP – Universidade Estadual Paulista, Departamento de Tecnologia, SP, Brazil; ^5^Departamento de Microbiologia, Universidade Federal de Minas Gerais, (UFMG), Belo Horizonte, MG, Brazil; ^6^Departamento de Ciências Biológicas (DECBI), Instituto de Ciências Exatas e Biológicas (ICEB), Universidade Federal de Ouro Preto (UFOP), Ouro Preto, MG, Brazil; ^7^Departamento de Bioquímica (DB), Instituto de Química (IQ), Universidade de São Paulo (USP), São Paulo, SP, Brazil; ^8^Biocomplexity Institute, Virginia Tech, Blacksburg, VA, USA.

**#Corresponding author**

LMM mailing address: Departamento de Ciências Biológicas (DECBI), Instituto de Ciências Exatas e Biológicas (ICEB), Universidade Federal de Ouro Preto (UFOP), Ouro Preto, MG, Brazil. Zip Code 35400-000. Phone: +55 (31) 3559-1698, FAX: +55 (31) 3559-1672, e-mail lmmorei@gmail.com

**SUPPLEMENTARY RESULTS**

**Carbohydrate central metabolism**

In the genome of SfFG3, we identified genes associated with the metabolism of trehalose, sucrose, maltose, maltodextrin, lactose, galactose, cellobiose, glycerol, mannitol, inositol, arabinose, sorbitol, xylose, galactonate, galacturonate, fucose, fructose, ketogluconate, and mannose. The Entner-Doudoroff pathways, Embden-Meyerhof glyoxylate pathway, and glyoxylate glycolate interconversion components were complete, enabling the TCA and pentose pathways, and glycogen synthesis. In addition, sixteen genes were associated with methylglyoxal metabolism, among them methylglyoxal synthase (*mgsA*), two copies of lactoylgluthatione lyase (*gloA*) and hydroxyacylglutathione hydrolase (*gloB*), a copy of aldehyde dehydrogenase B (*aldB*), and ten copies of aldehyde dehydrogenase A (*aldA*), arranged in five clusters of two tandem genes.

For pyruvate metabolism, genes associated with five pathways besides the anaplerotic reactions related to this metabolic product were identified: i) Decarboxylation of pyruvate to acetyl-CoA mediated by the pyruvate dehydrogenase complex (*pdh*) under aerobic conditions; ii) non-oxidative decarboxylation of pyruvate to acetaldehyde under anaerobic conditions in the presence of the enzyme pyruvate decarboxylase (*pdc*), and in the presence of the pyruvate:ferredoxin oxidoreductase enzyme, formed by the subunits (*pfoR*); iii) acetate dissimilation mediated by the enzyme phosphate acetyltransferase (*pat*) and acetate kinase (*ack*) arranged in tandem in the genome; iv) acetate assimilation mediated by the enzyme acetyl-coenzyme A synthetase that converts acetate into acetyl-coA, arranged in tandem with genes that coding for acetate permease (*actP*), and an accessory membrane protein associated with ActP; v) and a gene encoding pyruvate oxidase (*pox*), an alternative pathway for the formation of other acetyl-P moieties.

In relation to the fermentation pathways, SlFG3 has 24 genes associated with butyric fermentation, lactic acid fermentation, and ten other genes associated with acetoin and butanediol synthesis. In addition, SlFG3 still presents complete routes for use of chitin and N-acetylglycosamine with nine tandemly arranged genes *cbsA-ybfNM-nagTBARD-chiA*, another cluster representing the phosphotransferase system system of N-acetylglicosamine transport (*nagEABC*) and another PTS system for transport of chitobiose and its respective regulator (*chbABCR*).

**Respiration**
In the genome of SlFG3, we identified 171 genes associated with respiratory pathways, of which 39 were associated with biotin biosynthesis, 41 genes were associated with electron accepting reactions, 76 were associated with electron donating reactions, and another 54 not grouped into a specific category. In the electron accepting reaction subcategory, five genes related to tetrathionate respiration were found, arranged in tandem. These encode, respectively, for the A, B, and C subunits of the tetrathionate reductase enzyme (*ttrABC*) arranged upstream of the regulatory genes encoding the sensory and regulatory proteins (*ttrSR*). Similarly, nine genes were associated with trimethylamine N-oxide (*tmaO*) reductase, associated with anaerobic respiratory systems, six of which (*torACDT*) were arranged in tandem downstream of the *torSR* regulatory genes. Six genes were associated with cytochrome d terminal ubiquinol oxidase, two encoding the ATP-binding protein (*cydCD*), two subunits of I and II cytochrome d ubiquinol oxidase itself (*cydAB*), and two genes for cytochrome bd2 subunits I and II (II-II). Four genes were associated with the terminal cytochrome ubiquinol oxidase, which encode the I to IV (*cyoABCD*) subunits, respectively. Four other genes were associated with the fumarate respiration cluster, being the sensory and regulatory proteins (*dcuSR*), in addition to a C4-dicarboxylate transporter (*dcuB*), and a gene coding for fumarate hydratase class I. In the subclass of anaerobic respiratory reductases, five genes were identified: one coding for anaerobic dimethyl sulfoxide reductase (*dmsR*), ferredoxin reductase, flavodoxin reductase; and two copies of arsenate reductase. In the electron donating reaction subcategory, 14 genes were associated with complex I, eight with the succinate dehydrogenase complex, six were related to pyruvate dehydrogenase complex, 13 with biogenesis of c-type cytochromes, and 14 with NADH ubiquinone oxidoreductase, 6 with NiFe hydrogenase maturation and another 13 involved with Na (+)-translocating NADH-quinone oxidoreductase, and an nmr-like group of electron transport complexes.

**Invasion and intracellular resistance**

Despite all of the resistance repertoire found in the SlFG3 genome, no class of these genes draws more attention than the genes associated with ribosomal proteins associated with Mycobacterium virulence. Fourteen genes were found in three gene pools. One of these clusters contained the minor ribosome subunit (rv0682-83-84-85) associated genes that encode S12p, S7p, ef-G, and ef-Tu, respectively. The second cluster contained the rv1641 /42/43 genes encoding a translation initiation factor 3 and the L35p and L20p proteins, respectively. The third cluster contained the rv0067-68 genes encoding the beta-subunits of DNA-directed RNA polymerase, respectively. Finally, what should have been the fourth gene cluster, known as the Mycobacterium virulence operon and possibly involved in quinolinate biosynthesis, in the investigated strains, these genes are distributed throughout the genome without well-defined characteristic clustering: rv1594 - quinolinate synthase, rv1595 - L-aspartate oxidase and rv1596 - quinolinate phosphoribosyltransferase.

**Other stress adaptations**

In addition to a large repertoire of genes associated with oxidative stress protection, SlFG3 presents: five genes associated with cold shock stress, among them *cspA*, two copies of *cspC*, *cspE*, and *cspG*; 14 genes associated with heat shock stress, among them *dnaJ* and *dnaK* arranged in tandem, and *grpE* and *smpB,* also in tandem; five genes associated with selenate and selenite resistance; seven genes associated with periplasmic stress response; 33 genes associated with osmotic stress, among them a cluster of *osmB-yciM-pgpB-yciT* genes encoding the ABC transporter *yehZYXW* of enterobacteriales, and four (*aquZ, guf, ompA* and *osmY*) involved with osmoregulation; five associated with cytoplasmic glucans synthesis *mdoHGC* and arranged in tandem, *mdoB* and *ndvB*; 15 genes associated with choline and betaine uptake, *betA* as five copies, *betB*, *betT* as two copies, *betI*; in addition to the genes coding for an ABC proline, glycine and betaine (*proXVWZ*) transporter.

**Response and adaptation to redox processes**

Regarding the oxidative stress pathways, it was possible to identify 62 genes related to this function in SlFG3. Of 62 genes, 6 were annotated in the subcategory of enzymes associated with reactive species protection, among them copies of superoxide dismutase, *sodA* (Mn), *sodB* (Fe) and *sodC* (Cu-Zn), catalase and peroxidase arranged in tandem, and cytochrome c511 peroxidase (Figure 5C). Regarding the subcategory of oxidative stress, 21 genes were identified. Among these was a cluster composed of the *bhpO-dps-fr-oxyR* genes encoding a bacterio-ferrichrome oxygenase, non-specific DNA binding protein, ferroxidase and a hydrogen peroxide-inducible gene activator (Figure 5C). All other strains had two copies of this cluster, being present in single copy only in SlFG3. In addition, three genes associated with the regulation of oxidative metabolism, two copies of *furP* (ferric uptake regulation), three copies of *fnr* (fumarate and nitrite reduction regulatory), and the *zur* (zinc uptake regulator) gene in the SlFG3 genome were annotated. Regarding glutaredoxins, all strains investigated had a copy of glutaredoxin 1, 2 and 3, and a copy of glutaredoxin-like protein NrdH (Figure 5C). All of these genes collectively denote the metabolic ability of SlFG3 to detoxify reactive oxygen and nitrogen species, thereby protecting lipids, nucleic acids, and proteins from the damage potentially generated by these compounds (Figure 5D).

**SUPPLEMENTARY FIGURES**

**
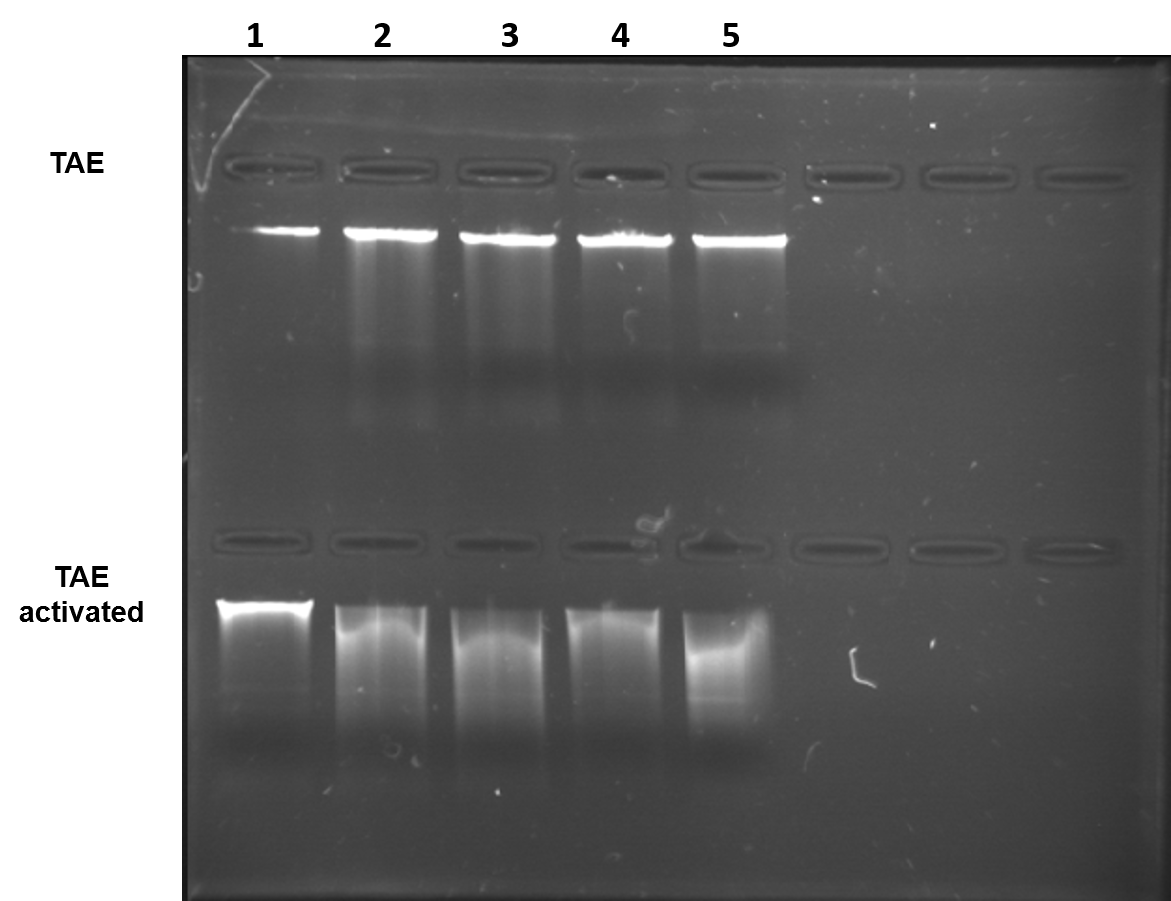
**

**Supplementary Fig. S1. Dnd phenotypes of SFG3:** Electrophoresis gel of DNA extracted from *Serratia liquefaciens* treated with 5 mM (lane 2) or 7.5 mM (lane 3) of arsenite and with 5 mM (lane 4) or 7.5 mM (lane 5) of hydrogen peroxide (lane 1 represents control).When incubated with TAE (top line) the DNA stays whole and when incubated with activated TAE (bottom line) the DNA undergoes degradation. Picture of the full gel without any image treatment.

**
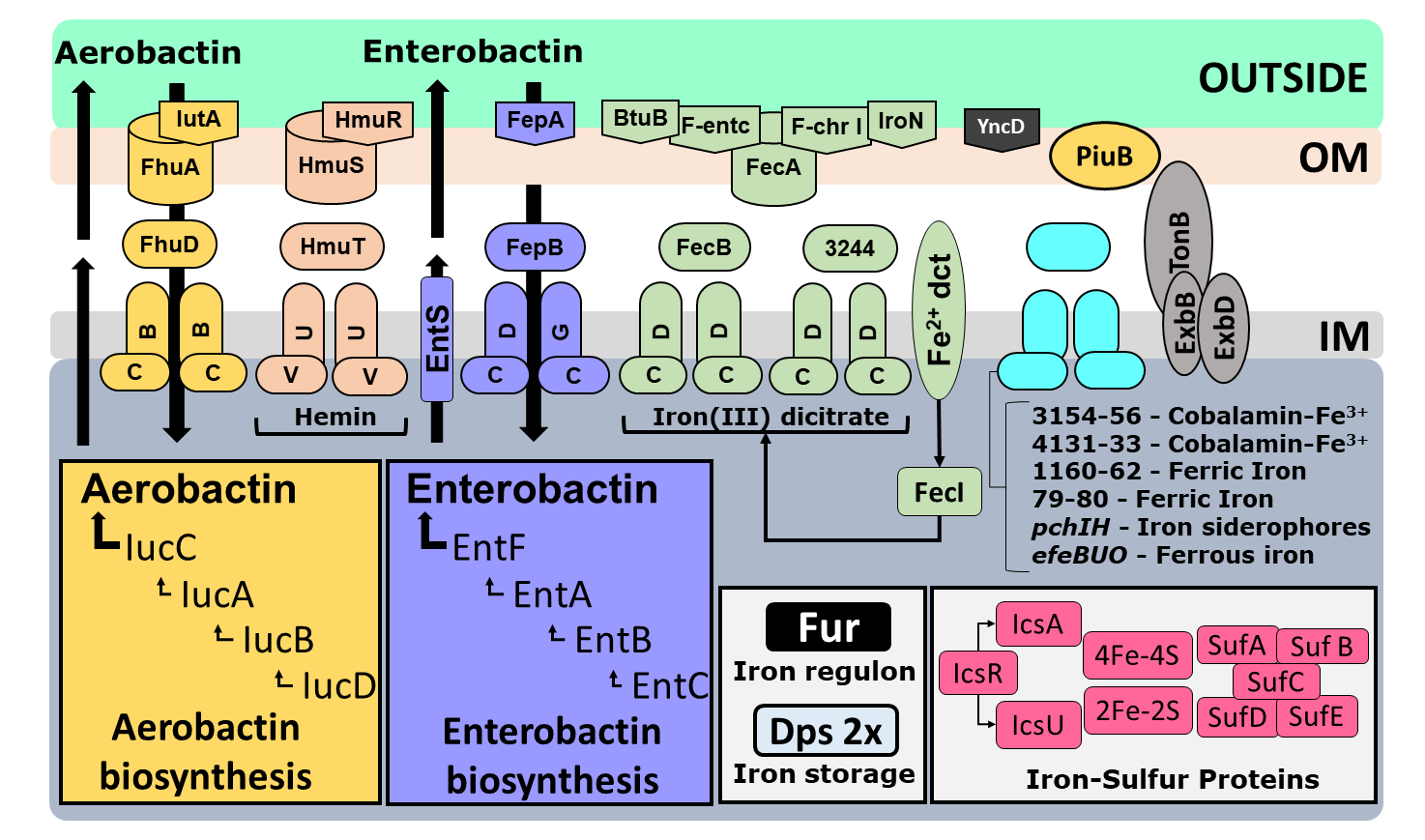
**

**Supplementary Fig. S2.** Analysis of genes and proteins related to SlFg3 iron uptake and metabolism. A total of 110 genes are responsible for the codification of the proteins that make up this intricate network of iron uptake and metabolism.


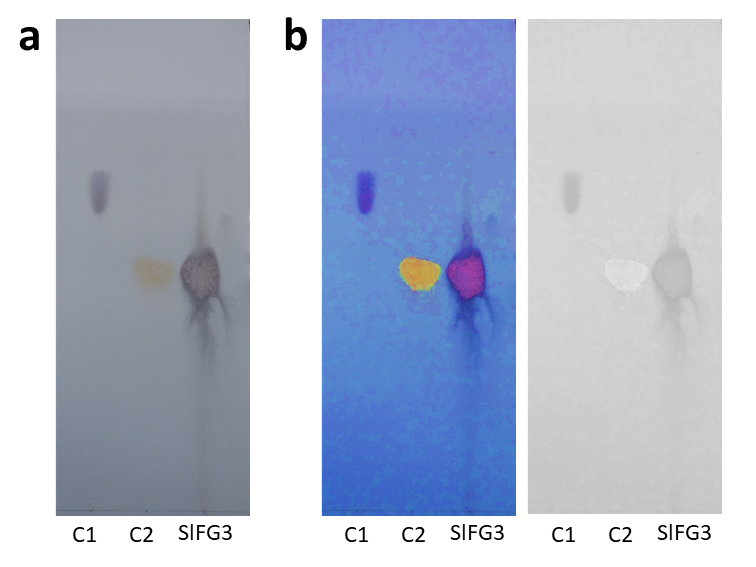


**Supplementary Fig. S3.** Analysis of siderophore production using TLC silica plates. The C1 and C2 spots corresponds to 6 µg of the commercial siderophores 2,3-dihydroxybenzoic acid and acetohydroxamic acid, respectively. It is possible to observe that SlFG3 can produce two siderophores: one hydroxamate-type (stained in yellow) and one catecholate-type (stained in purple), which corroborates the presence of the gene clusters, respectively, associated with aerobactin and enterobactin synthesis. TLC plate developed with ethanolic solution of ferric chloride (0.5%) at visible light. (a) Picture of the TLC without any image treatment. (b and c) Picture of the TLC with two different color filters.

**SUPPLEMENTARY TABLES**

**Supplementary Table 1.** Genome dataset.

| ***Strain*** | **Abbreviation** | **Source** | **Status** | **Replicons** | **Proteins** | **%GC** | **Reference** | **Genbank Assembly** |
| --- | --- | --- | --- | --- | --- | --- | --- | --- |
| *Serratia liquefaciens* FG3 | SlFG3 | Plant | complete | chr | 5.398 | 0.55 | this work | PRJNA505252 (Bioproject) |
|  |  |  |  | p1 | 179 | 0.54 |  |  |
|  |  |  |  | p2 | 146 | 0.52 |  |  |
| *Serratia ficaria* NBRC102596 | NBRC102596 | Human | contig | chr | 4.83 | 0.60 | Brouillard *et al.,* 1984 | GCA_001590885.1 |
| *Serratia fonticola* DSM4576 | DSM4576 | Water | complete | chr | 5.356 | 0.54 | Lyn *et al.,* 2015 | GCA_001006005.1 |
| *Serratia fonticola* GS2 | GS2 | Plant | complete | chr | 5.46 | 0.54 | Jung *et al.*, 2016 | GCA_001514455.1 |
|  |  |  |  | pSF001 | 167 | 0.50 |  |  |
|  |  |  |  | pSF002 | 118 | 0.53 |  |  |
| *Serratia liquefaciens* ATCC27592 | ATCC27592 | Milk | complete | chr | 4.858 | 0.55 | Tan *et al.,* 2001 | GCA_000422085.1 |
|  |  |  |  | p1 | 35 | 0.53 |  |  |
| *Serratia liquefaciens* FDAARGOS125 | FDAARGOS125 | Human | complete | chr | 4.954 | 0.55 | Goldberg *et al*., 2015 | GCA_001559135.1 |
| *Serratia liquefaciens* HUMV21 | HUMV21 | Human | complete | chr | 4.907 | 0.55 | Lazaro-Diez *et al.,* 2015 | GCA_000975245.1 |
| *Serratia marcences* B3R3 | B3R3 | Plant | complete | chr | 5.078 | 0.59 | Xiao-Qiang Wang *et al.,* 2015 | GCA_001417865.2 |
|  |  |  |  | p1 | 122 | 0.52 |  |  |
| *Serratia marcences* CAV1492 | CAV1492 | Clinical (*Homo sapiens)* | complete | chr | 5.019 | 0.59 | Vicente *et al.,* 2016 | GCA_001022215.1 |
|  |  |  |  | p199 | 212 | 0.51 |  |  |
|  |  |  |  | p3223 | 4 | 0.56 |  |  |
|  |  |  |  | p6393 | 6 | 0.53 |  |  |
|  |  |  |  | p73 | 94 | 0.53 |  |  |
|  |  |  |  | pKPC | 79 | 0.49 |  |  |
| *Serratia marcences* DB11 | DB11 | Insect | complete | chr | 4.68 | 0.60 | Iguchi *et al.,* 2014 | GCA_000513215.1 |
| *Serratia marcences* FGI94 | FGI94 | Fungus | complete | chr | 4.365 | 0.59 | Aylward *et al.,* 2013 | GCA_000330865.1 |
| *Serratia marcences* RSC14 | RSC14 | Plant | complete | chr | 4.684 | 0.60 | Kham *et al.,* 2017 | GCA_001280365.1 |
| *Serratia marcences* SM39 | SM39 | Human | complete | chr | 4.779 | 0.60 | Iguchi *et al.,* 2014 | GCA_000828775.1 |
|  |  |  |  | pSMC1 | 54 | 0.61 |  |  |
|  |  |  |  | pSMC2 | 67 | 0.52 |  |  |
| *Serratia marcences* SmUNAM836 | SmUNAM836 | Human | complete | chr | 4.769 | 0.60 | Miranda *et al.,* 2016 | GCA_001294565.1 |
|  |  |  |  | pSmUNAM836 | 36 | 0.44 |  |  |
| *Serratia marcences* WW4 | WW4 | Paper machine aggregates | complete | chr | 4.827 | 0.60 | Chung *et al.,* 2013 | GCA_000336425.1 |
|  |  |  |  | pSmWW4 | 3 | 0.48 |  |  |
| *Serratia multitudinisentens* RB25 | RB25 | Landfill | complete | chr | 4.902 | 0.51 | Lim *et al.,* 2015 | GCA_000520015.2 |
| *Serratia nematodiphila* DZ0503SBS1 | DZ0503SBS1 | Nematode | contig | chr | 4.806 | 0.60 | Zhang *et al.,* 2009 | GCA_000738675.1 |
| *Serratia odorifera* DSM4582 | DSM4582 | Sputum | scaffold | chr | 4.698 | 0.56 | Tan *et al.,* 2001 | GCA_000163595.1 |
| *Serratia plymuthica* 3Re418 | 3Re418 | Plant | complete | chr | 4.941 | 0.56 | Berg *et al.,* 2005 | GCA_001663135.1 |
| *Serratia plymuthica* 3RP8 | 3RP8 | Rizosphere | complete | chr | 5.073 | 0.56 | Adam *et al.,* 2016 | GCA_001663115.1 |
| *Serratia plymuthica* 4Rx13 | 4Rx13 | Plant | complete | chr | 4.917 | 0.56 | Weise *et al.,* 2014 | GCA_000176835.2 |
| *Serratia plymuthica* AS9 | AS9 | Field samples of rapeseed roots | complete | chr | 4.975 | 0.56 | Neupane *et al.,* 2012 | GCA_000214235.1 |
| *Serratia plymuthica* PRI2c | PRI2c | Maize rhizosphere soil | complete | chr | 5.012 | 0.56 | Garbeva *et al.,* 2012 | GCA_000261045.2 |
| *Serratia plymuthica* S13 | S13 | *Styrian pumpkin* anthrosphere | complete | chr | 4.963 | 0.56 | Muller *et al.,* 2013 | GCA_000438825.1 |
| *Serratia proteamaculans* 568 | 568 | Root endophyte from *Populus trichocarpa* | complete | chr | 4.997 | 0.55 | Purushotham *et al.,* 2012 | GCA_000018085.1 |
|  |  |  |  | pSPRO01 | 75 | 0.49 |  |  |
| *Serratia rubidaea* 1122 | 1122 | Sputum | complete | chr | 4.450 | 0.59 | Bonnin *et al.,* 2015 | GCA_001572725.1 |
| *Serratia sp.* AS12 | AS12 | Rape seed roots | complete | chr | 4.972 | 0.56 | Neupane *et al.*, 2012 | GCA_000214195.1 |
| *Serratia sp.* AS13 | AS13 | Rape seed roots | complete | chr | 4.974 | 0.56 | Neupane *et al.,* 2012 | GCA_000214805.1 |
| *Serratia sp.* FS14 | FS14 | *Atractylodes macrocephala Koidz* plant | complete | chr | 4.778 | 0.59 | Li *et al.,* 2015 | GCA_000695995.1 |
| *Serratia sp.* SCBI | SCBI | Nematode *Caenorhabditis briggsae* | complete | chr | 4.647 | 0.60 | Petersen *et al.,* 2014 | GCA_000747565.1 |
|  |  |  |  | p1 | 77 | 0.55 |  |  |
| *Serratia sp.*YD25 | YD25 | Rizosphere tabacco farm | complete | chr | 4.724 | 0.60 | Su *et al.,* 2017 | GCA_001642805.2 |
| *Serratia symbiotica cinaracedri* | cinaracedri | Insect *Cinara cedri* | complete | chr | 739 | 0.29 | Lamelas *et al.,* 2011 | GCA_000238975.1 |
| *Serratia symbiotica* STs | STs | Insect *Tuberolachnus salignus* | complete | chr | 498 | 0.21 | Lamelas *et al.,* 2008 | GCA_900016775.1 |
| *Serratia ureilytica* Lr5/4 | Lr5/4 | Geothermal spring water | contig | chr | 4.982 | 0.59 | - | GCA_000988045.1 |

Chr – Chromosome; p – plasmid

**Supplementary Table 2.** SlFG3 plasmids annotation.

| SlFG3 plasmids | Gi and Gb SlFG3 | #AA | Id.(%) | Cov.(%) | e-Value | Score | Gi and ref seq (reference) | Genus specie (reference) | Product (annotation) | Class | Order |
| --- | --- | --- | --- | --- | --- | --- | --- | --- | --- | --- | --- |
| p1-125 | 1702204641QDL35596.1 | 260 | 98.462 | 100 | 6.38e-178 | 499 | 1741177972\|WP_149571966.1 | Serratia marcescens | type-F conjugative transfer system pilin acetylase TraX | Gammaproteobacteria | Enterobacterales |
| p1-125 | 1702204642\|QDL35597.1 | 72 | 95.833 | 100 | 1.39e-43 | 144 | 1696703001WP_141173965.1 | Serratia sp. SRS-8-S-2018 | hypothetical protein | Gammaproteobacteria | Enterobacterales |
| p1-125 | 1702204643\|QDL35598.1 | 1768 | 96.267 | 100 | 0.0 | 3445 | 1741177970\|WP_149571964.1 | Serratia marcescens | conjugative transfer relaxase/helicase TraI | Gammaproteobacteria | Enterobacterales |
| p1-125 | 1702204644\|QDL35599.1 | 732 | 99.317 | 100 | 0.0 | 1512 | 1696703000\|WP_141173964.1 | Serratia sp. SRS-8-S-2018 | type IV conjugative transfer system coupling protein TraD | Gammaproteobacteria | Enterobacterales |
| p1-125 | 1702204645\|QDL35600.1 | 69 | 98.551 | 100 | 9.29e-42 | 139 | 1728193209\|WP_147882558.1 | Serratia marcescens | hypothetical protein | Gammaproteobacteria | Enterobacterales |
| p1-125 | 1702204646\|QDL35601.1 | 176 | 99.432 | 100 | 1.52e-121 | 349 | 727177243\|WP_033641526.1 | Serratia | hypothetical protein | Gammaproteobacteria | Enterobacterales |
| p1-125 | 1702204647\|QDL35602.1 | 943 | 97.985 | 100 | 0.0 | 1902 | 1048493043\|emb\|SAY46458.1 | Serratia marcescens | Uncharacterised protein (plasmid) | Gammaproteobacteria | Enterobacterales |
| p1-125 | 1702204648\|QDL35603.1 | 455 | 94.725 | 100 | 0.0 | 898 | 1741177963\|WP_149571957.1 | Serratia marcescens | F-type conjugal transfer protein TraH | Gammaproteobacteria | Enterobacterales |
| p1-125 | 1702204649\|QDL35604.1 | 137 | 89.781 | 100 | 6.67e-86 | 256 | 1160408659\|WP_079450615.1 | Serratia marcescens | hypothetical protein | Gammaproteobacteria | Enterobacterales |
| p1-125 | 1702204650\|QDL35605.1 | 179 | 95.531 | 100 | 2.67e-122 | 351 | 1728193214\|WP_147882563.1 | Serratia marcescens | type-F conjugative transfer system pilin assembly thiol-disulfide isomerase TrbB | Gammaproteobacteria | Enterobacterales |
| p1-125 | 1702204651QDL35606.1 | 104 | 97.115 | 100 | 7.95e-69 | 210 | 503838595\|WP_014072589.1 | Serratia | conjugal transfer pilin chaperone TraQ | Gammaproteobacteria | Enterobacterales |
| p1-125 | 1702204652\|QDL35607.1 | 264 | 99.242 | 100 | 0.0 | 534 | 1093660812\|WP_070914264.1 | Serratia marcescens | type-F conjugative transfer system pilin assembly protein TraF | Gammaproteobacteria | Enterobacterales |
| p1-125 | 1702204653\|QDL35608.1 | 634 | 99.527 | 100 | 0.0 | 1317 | 1559989386\|WP_128383113.1 | Serratia marcescens | type-F conjugative transfer system mating-pair stabilization protein TraN | Gammaproteobacteria | Enterobacterales |
| p1-125 | 1702204654\|QDL35609.1 | 224 | 96.875 | 100 | 9.22e-157 | 442 | 757523303\|WP_042785926.1 | Serratia sp. SCBI | type-F conjugative transfer system pilin assembly protein TrbC | Gammaproteobacteria | Enterobacterales |
| p1-125 | 1702204655\|QDL35610.1 | 332 | 99.398 | 100 | 0.0 | 682 | 1741177957\|WP_149571951.1 | Serratia marcescens | conjugal transfer pilus assembly protein TraU | Gammaproteobacteria | Enterobacterales |
| p1-125 | 1702204656\|QDL35611.1 | 125 | 98.361 | 98 | 4.40e-83 | 248 | 1701559841WP_142106073.1 | Serratia marcescens | hypothetical protein | Gammaproteobacteria | Enterobacterales |
| p1-125 | 1702204657\|QDL35612.1 | 211 | 99.526 | 100 | 1.30e-153 | 433 | 676311824\|gb\|AIM24263.1 | Serratia sp. SCBI | conjugal transfer pilus assembly protein TraW (plasmid) | Gammaproteobacteria | Enterobacterales |
| p1-125 | 1702204658\|QDL35613.1 | 278 | 99.281 | 100 | 0.0 | 565 | 757523307\|WP_042785930.1 | Serratia sp. SCBI | DsbA family protein | Gammaproteobacteria | Enterobacterales |
| p1-125 | 1702204659\|QDL35614.1 | 139 | 97.842 | 100 | 3.42e-93 | 275 | 1181282699\|WP_084587994.1 | Serratia sp. SCBI | type-F conjugative transfer system protein TrbI | Gammaproteobacteria | Enterobacterales |
| p1-125 | 1702204660\|QDL35615.1 | 876 | 99.772 | 100 | 0.0 | 1823 | 983383398\|WP_060560512.1 | Serratia marcescens | type IV secretion system protein TraC | Gammaproteobacteria | Enterobacterales |
| p1-125 | 1702204661QDL35616.1 | 128 | 98.438 | 100 | 4.30e-85 | 253 | 1701528615\|WP_142076378.1 | Serratia marcescens | hypothetical protein | Gammaproteobacteria | Enterobacterales |
| p1-125 | 1702204662\|QDL35617.1 | 72 | 93.056 | 100 | 2.59e-41 | 138 | 1741177949\|WP_149571943.1 | Serratia marcescens | hypothetical protein | Gammaproteobacteria | Enterobacterales |
| p1-125 | 1702204663\|QDL35618.1 | 465 | 98.710 | 100 | 0.0 | 935 | 676311832\|gb\|AIM24271.1 | Serratia sp. SCBI | conjugal transfer pilus assembly protein TraB (plasmid) | Gammaproteobacteria | Enterobacterales |
| p1-125 | 1702204664\|QDL35619.1 | 242 | 98.347 | 100 | 6.88e-174 | 487 | 1701528610\|WP_142076373.1 | Serratia marcescens | type-F conjugative transfer system secretin TraK | Gammaproteobacteria | Enterobacterales |
| p1-125 | 1702204665\|QDL35620.1 | 187 | 99.465 | 100 | 2.45e-132 | 377 | 1048493022\|emb\|SAY46437.1 | Serratia marcescens | TraE protein (plasmid) | Gammaproteobacteria | Enterobacterales |
| p1-125 | 1702204666\|QDL35621.1 | 101 | 99.010 | 100 | 1.88e-67 | 206 | 1160408675\|WP_079450631.1 | Serratia marcescens | type IV conjugative transfer system protein TraL | Gammaproteobacteria | Enterobacterales |
| p1-125 | 1702204667\|QDL35622.1 | 120 | 99.167 | 100 | 2.95e-77 | 233 | 1728193230\|WP_147882579.1 | Serratia marcescens | type IV conjugative transfer system pilin TraA | Gammaproteobacteria | Enterobacterales |
| p1-125 | 1702204668\|QDL35623.1 | 233 | 99.571 | 100 | 2.61e-172 | 482 | 1545335404\|WP_126502344.1 | Serratia marcescens | 7-cyano-7-deazaguanine synthase QueC | Gammaproteobacteria | Enterobacterales |
| p1-125 | 1702204669\|QDL35624.1 | 285 | 99.649 | 100 | 0.0 | 575 | 917017282\|WP_051623994.1 | Bacteria | queuosine precursor transporter | Undefined | Undefined |
| p1-125 | 1702204670\|QDL35625.1 | 198 | 96.970 | 100 | 1.78e-134 | 384 | 291420051gb\|EFE93328.1 | Serratia odorifera DSM 4582 | hypothetical protein HMPREF0758_5060 | Gammaproteobacteria | Enterobacterales |
| p1-125 | 1702204671QDL35626.1 | 138 | 98.551 | 100 | 1.27e-95 | 281 | 1160408679\|WP_079450635.1 | Serratia marcescens | helix-turn-helix transcriptional regulator | Gammaproteobacteria | Enterobacterales |
| p1-125 | 1702204672\|QDL35627.1 | 132 | 99.242 | 100 | 9.67e-91 | 268 | 1545335407\|WP_126502347.1 | Serratia marcescens | relaxosome protein TraM | Gammaproteobacteria | Enterobacterales |
| p1-125 | 1702204673\|QDL35628.1 | 156 | 99.359 | 100 | 1.62e-113 | 327 | 757523324\|WP_042785946.1 | Bacteria | transglycosylase SLT domain-containing protein | Undefined | Undefined |
| p1-125 | 1702204674\|QDL35629.1 | 98 | 96.939 | 100 | 1.50e-65 | 201 | 1706435400\|WP_142816607.1 | Serratia liquefaciens | hypothetical protein | Gammaproteobacteria | Enterobacterales |
| p1-125 | 1702204675\|QDL35630.1 | 275 | 99.273 | 100 | 0.0 | 570 | 751569733\|WP_041038628.1 | Serratia marcescens | DUF945 domain-containing protein | Gammaproteobacteria | Enterobacterales |
| p1-125 | 1702204676\|QDL35631.1 | 123 | 98.374 | 100 | 7.50e-86 | 254 | 757523327\|WP_042785948.1 | Serratia sp. SCBI | hypothetical protein | Gammaproteobacteria | Enterobacterales |
| p1-125 | 1702204677\|QDL35632.1 | 265 | 98.113 | 100 | 0.0 | 541 | 1706435396\|WP_142816605.1 | Serratia liquefaciens | N-6 DNA methylase | Gammaproteobacteria | Enterobacterales |
| p1-125 | 1702204678\|QDL35633.1 | 87 | 98.851 | 100 | 6.05e-56 | 176 | 751569727\|WP_041038622.1 | Serratia marcescens | hypothetical protein | Gammaproteobacteria | Enterobacterales |
| p1-125 | 1702204679\|QDL35634.1 | 115 | 99.130 | 100 | 1.81e-79 | 238 | 1048493010\|emb\|SAY46425.1 | Serratia marcescens | Uncharacterised protein (plasmid) | Gammaproteobacteria | Enterobacterales |
| p1-125 | 1702204680\|QDL35635.1 | 138 | 99.275 | 100 | 1.94e-98 | 288 | 751569723\|WP_041038618.1 | Serratia marcescens | hypothetical protein | Gammaproteobacteria | Enterobacterales |
| p1-125 | 1702204681QDL35636.1 | 140 | 95.714 | 100 | 5.85e-98 | 286 | 1254172804\|WP_097103321.1 | Serratia sp. JKS296 | hypothetical protein | Gammaproteobacteria | Enterobacterales |
| p1-125 | 1702204682\|QDL35637.1 | 157 | 95.541 | 100 | 6.67e-107 | 311 | 983153773\|WP_060452160.1 | Serratia marcescens | hypothetical protein | Gammaproteobacteria | Enterobacterales |
| p1-125 | 1702204683\|QDL35638.1 | 95 | 98.947 | 100 | 6.30e-63 | 194 | 1537720925\|WP_125460859.1 | Serratia | type II toxin-antitoxin system RelE/ParE family toxin | Gammaproteobacteria | Enterobacterales |
| p1-125 | 1702204684\|QDL35639.1 | 83 | 98.795 | 100 | 1.07e-52 | 168 | 983153774\|WP_060452161.1 | Serratia marcescens | type II toxin-antitoxin system Phd/YefM family antitoxin | Gammaproteobacteria | Enterobacterales |
| p1-125 | 1702204685\|QDL35640.1 | 148 | 98.649 | 100 | 2.72e-106 | 308 | 983153775\|WP_060452162.1 | Serratia marcescens | hypothetical protein | Gammaproteobacteria | Enterobacterales |
| p1-125 | 1702204686\|QDL35641.1 | 62 | 58.065 | 100 | 3.21e-19 | 82.4 | 1741176714\|WP_149570783.1 | Serratia marcescens | hypothetical protein | Gammaproteobacteria | Enterobacterales |
| p1-125 | 1702204687\|QDL35642.1 | 245 | 99.184 | 100 | 0.0 | 505 | 1545335374\|WP_126502315.1 | Serratia marcescens | plasmid SOS inhibition protein A | Gammaproteobacteria | Enterobacterales |
| p1-125 | 1702204688\|QDL35643.1 | 154 | 98.701 | 100 | 3.68e-110 | 318 | 742404360\|WP_038883481.1 | Serratia marcescens | conjugation system SOS inhibitor PsiB | Gammaproteobacteria | Enterobacterales |
| p1-125 | 1702204689\|QDL35644.1 | 692 | 95.882 | 98 | 0.0 | 1321 | 1537720921WP_125460855.1 | Serratia marcescens | chromosome partitioning protein | Gammaproteobacteria | Enterobacterales |
| p1-125 | 1702204690\|QDL35645.1 | 141 | 95.745 | 100 | 6.53e-97 | 284 | 742404355\|WP_038883476.1 | Serratia marcescens | antirestriction protein | Gammaproteobacteria | Enterobacterales |
| p1-125 | 1702204691QDL35646.1 | 73 | 98.630 | 100 | 8.10e-45 | 147 | 742404353\|WP_038883474.1 | Serratia marcescens | hypothetical protein | Gammaproteobacteria | Enterobacterales |
| p1-125 | 1702204692\|QDL35647.1 | 214 | 97.664 | 100 | 9.06e-153 | 431 | 1397621725\|WP_110147639.1 | Serratia marcescens | DNA methylase | Gammaproteobacteria | Enterobacterales |
| p1-125 | 1702204693\|QDL35648.1 | 67 | 98.507 | 100 | 2.28e-42 | 140 | 1397621724\|WP_110147638.1 | Serratia marcescens | hypothetical protein | Gammaproteobacteria | Enterobacterales |
| p1-125 | 1702204694\|QDL35649.1 | 143 | 96.503 | 100 | 4.80e-96 | 282 | 742404347\|WP_038883468.1 | Serratia marcescens | hypothetical protein | Gammaproteobacteria | Enterobacterales |
| p1-125 | 1702204695\|QDL35650.1 | 442 | 97.511 | 100 | 0.0 | 891 | 742404343\|WP_038883464.1 | Serratia marcescens | hypothetical protein | Gammaproteobacteria | Enterobacterales |
| p1-125 | 1702204696\|QDL35651.1 | 106 | 98.113 | 100 | 1.67e-70 | 214 | 1397621721WP_110147635.1 | Serratia marcescens | hypothetical protein | Gammaproteobacteria | Enterobacterales |
| p1-125 | 1702204697\|QDL35652.1 | 145 | 99.310 | 100 | 3.09e-100 | 293 | 1725078560\|gb\|TXE56925.1 | Serratia nematodiphila | hypothetical protein FOT58_19335 | Gammaproteobacteria | Enterobacterales |
| p1-125 | 1702204698\|QDL35653.1 | 378 | 99.735 | 100 | 0.0 | 774 | 983153805\|WP_060452192.1 | Serratia marcescens | ParB/RepB/Spo0J family partition protein | Gammaproteobacteria | Enterobacterales |
| p1-125 | 1702204699\|QDL35654.1 | 280 | 99.643 | 100 | 0.0 | 577 | 1048493127\|emb\|SAY46542.1 | Serratia marcescens | Chromosome-partitioning ATPase Soj (plasmid) | Gammaproteobacteria | Enterobacterales |
| p1-125 | 1702204700\|QDL35655.1 | 108 | 97.222 | 100 | 1.65e-71 | 218 | 983153787\|WP_060452174.1 | Serratia marcescens | hypothetical protein | Gammaproteobacteria | Enterobacterales |
| p1-125 | 1702204701QDL35656.1 | 259 | 95.753 | 100 | 1.77e-179 | 503 | 1397621716\|WP_110147630.1 | Serratia marcescens | phage integrase family protein | Gammaproteobacteria | Enterobacterales |
| p1-125 | 1702204702\|QDL35657.1 | 102 | 99.020 | 100 | 1.04e-69 | 212 | 983153789\|WP_060452176.1 | Serratia marcescens | hypothetical protein | Gammaproteobacteria | Enterobacterales |
| p1-125 | 1702204703\|QDL35658.1 | 119 | 85.714 | 100 | 1.27e-72 | 221 | 1094036041WP_071031495.1 | Klebsiella pneumoniae | hypothetical protein | Gammaproteobacteria | Enterobacterales |
| p1-125 | 1702204704\|QDL35659.1 | 85 | 75.294 | 100 | 9.89e-38 | 130 | 1537720909\|WP_125460843.1 | Serratia marcescens | hypothetical protein | Gammaproteobacteria | Enterobacterales |
| p1-125 | 1702204705\|QDL35660.1 | 206 | 97.087 | 100 | 2.39e-132 | 379 | 1048493119\|emb\|SAY46534.1 | Serratia marcescens | Uncharacterised protein (plasmid) | Gammaproteobacteria | Enterobacterales |
| p1-125 | 1702204706\|QDL35661.1 | 202 | 78.351 | 96 | 1.57e-105 | 311 | 1539046828\|WP_125875320.1 | Serratia marcescens | hypothetical protein, partial | Gammaproteobacteria | Enterobacterales |
| p1-125 | 1702204707\|QDL35662.1 | 251 | 50.000 | 89 | 9.05e-77 | 241 | 1603594587\|WP_134251410.1 | Acinetobacter haemolyticus | hypothetical protein | Gammaproteobacteria | Pseudomonadales |
| p1-125 | 1702204708\|QDL35663.1 | 308 | 98.701 | 100 | 0.0 | 625 | 1563830212\|WP_128885010.1 | Serratia marcescens | hypothetical protein | Gammaproteobacteria | Enterobacterales |
| p1-125 | 1702204709\|QDL35664.1 | 91 | 98.901 | 100 | 4.93e-59 | 184 | 515953488\|WP_017384071.1 | Enterobacterales | formaldehyde-responsive transcriptional repressor FrmR | Gammaproteobacteria | Enterobacterales |
| p1-125 | 1702204710\|QDL35665.1 | 369 | 99.187 | 100 | 0.0 | 756 | 817691304\|WP_046654883.1 | Klebsiella pneumoniae | S-(hydroxymethyl)glutathione dehydrogenase/class III alcohol dehydrogenase | Gammaproteobacteria | Enterobacterales |
| p1-125 | 1702204711QDL35666.1 | 132 | 99.242 | 100 | 1.09e-87 | 260 | 501556607\|WP_012561111.1 | Enterobacterales | VOC family protein | Gammaproteobacteria | Enterobacterales |
| p1-125 | 1702204712\|QDL35667.1 | 191 | 82.653 | 100 | 1.73e-94 | 282 | 1356493620\|WP_105230944.1 | Serratia sp. MYb239 | hypothetical protein | Gammaproteobacteria | Enterobacterales |
| p1-125 | 1702204713\|QDL35668.1 | 231 | 89.610 | 100 | 1.42e-153 | 435 | 518286275\|WP_019456483.1 | Serratia marcescens | IS6 family transposase | Gammaproteobacteria | Enterobacterales |
| p1-125 | 1702204714\|QDL35669.1 | 97 | 50.450 | 98 | 4.44e-21 | 92.8 | 1517061145\|emb\|VCX08713.1 | Klebsiella pneumoniae | Putative transposase (identified by ISEscan HMM) | Gammaproteobacteria | Enterobacterales |
| p1-125 | 1702204715\|QDL35670.1 | 80 | 96.250 | 100 | 8.80e-47 | 152 | 1356493629\|WP_105230953.1 | Serratia sp. MYb239 | type II toxin-antitoxin system ParD family antitoxin | Gammaproteobacteria | Enterobacterales |
| p1-125 | 1702204716\|QDL35671.1 | 96 | 91.489 | 98 | 1.37e-56 | 179 | 1093660852\|WP_070914292.1 | Serratia marcescens | type II toxin-antitoxin system RelE/ParE family toxin | Gammaproteobacteria | Enterobacterales |
| p1-125 | 1702204717\|QDL35672.1 | 109 | 53.211 | 100 | 7.23e-30 | 112 | 948119968\|WP_056778461.1 | Serratia sp. Leaf51 | hypothetical protein | Gammaproteobacteria | Enterobacterales |
| p1-125 | 1702204718\|QDL35673.1 | 407 | 96.560 | 100 | 0.0 | 825 | 1706435322\|WP_142816551.1 | Serratia liquefaciens | IS91 family transposase | Gammaproteobacteria | Enterobacterales |
| p1-125 | 1702204719\|QDL35674.1 | 1012 | 90.613 | 100 | 0.0 | 1821 | 969849147\|WP_058610010.1 | Enterobacter cancerogenus | autotransporter serine protease | Gammaproteobacteria | Enterobacterales |
| p1-125 | 1702204720\|QDL35675.1 | 232 | 97.414 | 100 | 2.68e-170 | 477 | 1551460162\|WP_127377949.1 | Morganella morganii | IS1 family transposase | Gammaproteobacteria | Enterobacterales |
| p1-125 | 1702204721QDL35676.1 | 246 | 93.089 | 100 | 9.73e-170 | 477 | 1696703153\|WP_141174117.1 | Serratia sp. SRS-8-S-2018 | carbonic anhydrase | Gammaproteobacteria | Enterobacterales |
| p1-125 | 1702204722\|QDL35677.1 | 186 | 100.000 | 100 | 1.73e-134 | 383 | 1702204722\|gb\|QDL35677.1 | Serratia liquefaciens | hypothetical protein EGO53_28160 (plasmid) | Gammaproteobacteria | Enterobacterales |
| p1-125 | 1702204723\|QDL35678.1 | 539 | 99.629 | 100 | 0.0 | 1102 | 1217706749\|WP_089181293.1 | Serratia marcescens | ISL3 family transposase | Gammaproteobacteria | Enterobacterales |
| p1-125 | 1702204724\|QDL35679.1 | 407 | 88.452 | 100 | 0.0 | 773 | 644942982\|WP_025380127.1 | Yersinia enterocolitica | IS91 family transposase | Gammaproteobacteria | Enterobacterales |
| p1-125 | 1702204725\|QDL35680.1 | 443 | 38.213 | 91 | 9.18e-79 | 259 | 1119819513\|WP_072712795.1 | Francisella sp. TX077310 | acyltransferase | Gammaproteobacteria | Thiotrichales |
| p1-125 | 1702204726\|QDL35681.1 | 293 | 61.794 | 99 | 7.60e-123 | 373 | 979098851gb\|KVT84064.1 | Burkholderia territorii | hypothetical protein WT25_13255 | Betabacteria | Burkholderiales |
| p1-125 | 1702204727\|QDL35682.1 | 204 | 98.529 | 100 | 1.10e-145 | 412 | 512720754\|WP_016479974.1 | Klebsiella pneumoniae | DUF2913 family protein | Gammaproteobacteria | Enterobacterales |
| p1-125 | 1702204728\|QDL35683.1 | 321 | 97.231 | 100 | 0.0 | 638 | 754494501WP_041911396.1 | Enterobacter sp. BIDMC 29 | Rpn family recombination-promoting nuclease/putative transposase | Gammaproteobacteria | Enterobacterales |
| p1-125 | 1702204729\|QDL35684.1 | 76 | 94.737 | 100 | 9.73e-44 | 145 | 881086204\|WP_048796378.1 | Serratia | AbrB/MazE/SpoVT family DNA-binding domain-containing protein | Gammaproteobacteria | Enterobacterales |
| p1-125 | 1702204730\|QDL35685.1 | 138 | 91.304 | 100 | 3.82e-89 | 264 | 544754902\|WP_021181796.1 | Serratia | type II toxin-antitoxin system VapC family toxin | Gammaproteobacteria | Enterobacterales |
| p1-125 | 1702204731QDL35686.1 | 177 | 99.435 | 100 | 3.56e-127 | 363 | 1741176737\|WP_149570806.1 | Serratia marcescens | hypothetical protein | Gammaproteobacteria | Enterobacterales |
| p1-125 | 1702204732\|QDL35687.1 | 407 | 96.560 | 100 | 0.0 | 825 | 1706435307\|WP_142816538.1 | Serratia liquefaciens | IS91 family transposase | Gammaproteobacteria | Enterobacterales |
| p1-125 | 1702204733\|QDL35688.1 | 133 | 78.788 | 99 | 7.06e-62 | 203 | 1726115680\|WP_147457654.1 | Serratia plymuthica | hypothetical protein | Gammaproteobacteria | Enterobacterales |
| p1-125 | 1702204734\|QDL35689.1 | 314 | 92.357 | 100 | 0.0 | 576 | 1575565284\|WP_129993723.1 | Serratia | Rpn family recombination-promoting nuclease/putative transposase | Gammaproteobacteria | Enterobacterales |
| p1-125 | 1702204735\|QDL35690.1 | 448 | 95.089 | 100 | 0.0 | 881 | 1511388282\|WP_123060779.1 | Enterobacter | IS3 family transposase | Gammaproteobacteria | Enterobacterales |
| p1-125 | 1702204736\|QDL35691.1 | 209 | 92.823 | 100 | 1.06e-135 | 389 | 1516212871WP_123193171.1 | Serratia nematodiphila | transposase | Gammaproteobacteria | Enterobacterales |
| p1-125 | 1702204737\|QDL35692.1 | 115 | 98.261 | 100 | 3.64e-77 | 232 | 1696702971WP_141173935.1 | Serratia | IS66 family insertion sequence element accessory protein TnpB | Gammaproteobacteria | Enterobacterales |
| p1-125 | 1702204738\|QDL35693.1 | 275 | 90.667 | 99 | 3.80e-39 | 153 | 1093660700\|WP_070914192.1 | Serratia marcescens | IS66 family transposase | Gammaproteobacteria | Enterobacterales |
| p1-125 | 1702204739\|QDL35694.1 | 982 | 65.517 | 99 | 0.0 | 1221 | 1482751761WP_119803024.1 | Serratia sp. S40 | RHS repeat protein | Gammaproteobacteria | Enterobacterales |
| p1-125 | 1702204740\|QDL35695.1 | 1426 | 87.666 | 99 | 0.0 | 2523 | 1341289867\|WP_103780157.1 | Citrobacter amalonaticus | virulence protein | Gammaproteobacteria | Enterobacterales |
| p1-125 | 1702204741QDL35696.1 | 1994 | 67.202 | 99 | 0.0 | 2712 | 1214890268\|WP_088901210.1 | Pantoea sp. AMG 501 | hypothetical protein | Gammaproteobacteria | Enterobacterales |
| p1-125 | 1702204742\|QDL35697.1 | 230 | 83.983 | 100 | 1.07e-140 | 402 | 501633013\|WP_012606398.1 | Yersinia pseudotuberculosis | IS6-like element ISYps1 family transposase | Gammaproteobacteria | Enterobacterales |
| p1-125 | 1702204743\|QDL35698.1 | 141 | 75.177 | 100 | 4.71e-71 | 219 | 1020048486\|WP_063197006.1 | Serratia plymuthica | lysozyme | Gammaproteobacteria | Enterobacterales |
| p1-125 | 1702204744\|QDL35699.1 | 163 | 53.191 | 87 | 3.07e-38 | 137 | 916756730\|WP_051363786.1 | Serratia fonticola | lysis protein | Gammaproteobacteria | Enterobacterales |
| p1-125 | 1702204745\|QDL35700.1 | 111 | 47.917 | 86 | 1.68e-19 | 86.3 | 1719679525\|emb\|VVA49792.1 | Serratia vespertilionis | hypothetical protein SERVES_03551 | Gammaproteobacteria | Enterobacterales |
| p1-125 | 1702204746\|QDL35701.1 | 60 | 66.667 | 100 | 6.00e-17 | 76.3 | 1047798689\|gb\|OCO92370.1 | Serratia marcescens | hypothetical protein AN655_0215755 | Gammaproteobacteria | Enterobacterales |
| p1-125 | 1702204747\|QDL35702.1 | 499 | 98.397 | 100 | 0.0 | 1014 | 1217746180\|WP_089191634.1 | Serratia marcescens | IS21 family transposase | Gammaproteobacteria | Enterobacterales |
| p1-125 | 1702204748\|QDL35703.1 | 253 | 99.209 | 100 | 0.0 | 511 | 1217746179\|WP_089191633.1 | Serratia marcescens | DUF815 domain-containing protein | Gammaproteobacteria | Enterobacterales |
| p1-125 | 1702204749\|QDL35704.1 | 283 | 98.940 | 100 | 0.0 | 581 | 1706435485\|WP_142816658.1 | Serratia liquefaciens | AAA family ATPase | Gammaproteobacteria | Enterobacterales |
| p1-125 | 1702204750\|QDL35705.1 | 97 | 98.969 | 100 | 1.00e-62 | 194 | 1217741249\|WP_089186945.1 | Serratia | hypothetical protein | Gammaproteobacteria | Enterobacterales |
| p1-125 | 1702204751QDL35706.1 | 63 | 98.413 | 100 | 3.77e-37 | 127 | 751569715\|WP_041038610.1 | Serratia marcescens | hypothetical protein | Gammaproteobacteria | Enterobacterales |
| p1-125 | 1702204752\|QDL35707.1 | 70 | 95.714 | 100 | 3.41e-41 | 138 | 1236082332\|WP_094887914.1 | Serratia marcescens | hypothetical protein | Gammaproteobacteria | Enterobacterales |
| p1-125 | 1702204753\|QDL35708.1 | 236 | 99.576 | 100 | 3.13e-170 | 477 | 751569711WP_041038606.1 | Enterobacterales | tyrosine-type recombinase/integrase | Gammaproteobacteria | Enterobacterales |
| p1-125 | 1702204754\|QDL35709.1 | 178 | 99.438 | 100 | 3.14e-129 | 369 | 1028641819\|WP_063988447.1 | Serratia marcescens | stationary phase growth adaptation protein | Gammaproteobacteria | Enterobacterales |
| p1-125 | 1702204755\|QDL35710.1 | 211 | 98.578 | 100 | 2.24e-154 | 435 | 918424283\|WP_052475459.1 | Serratia marcescens | DUF2913 family protein | Gammaproteobacteria | Enterobacterales |
| p1-125 | 1702204756\|QDL35711.1 | 253 | 99.209 | 100 | 0.0 | 511 | 1217746179\|WP_089191633.1 | Serratia marcescens | DUF815 domain-containing protein | Gammaproteobacteria | Enterobacterales |
| p1-125 | 1702204757\|QDL35712.1 | 499 | 98.397 | 100 | 0.0 | 1014 | 1217746180\|WP_089191634.1 | Serratia marcescens | IS21 family transposase | Gammaproteobacteria | Enterobacterales |
| p1-125 | 1702204758\|QDL35713.1 | 64 | 63.333 | 94 | 6.08e-19 | 81.6 | 1569684236\|gb\|RYM68082.1 | Serratia liquefaciens | hypothetical protein BSR00_24610 | Gammaproteobacteria | Enterobacterales |
| p1-125 | 1702204759\|QDL35714.1 | 208 | 99.519 | 100 | 2.24e-148 | 420 | 1563830225\|WP_128885023.1 | Serratia | AAA family ATPase | Gammaproteobacteria | Enterobacterales |
| p1-125 | 1702204760\|QDL35715.1 | 67 | 97.015 | 100 | 1.90e-39 | 133 | 1575565277\|WP_129993716.1 | Serratia | chromosome partitioning protein ParB | Gammaproteobacteria | Enterobacterales |
| p1-125 | 1702204761QDL35716.1 | 84 | 95.062 | 96 | 1.05e-49 | 160 | 1696703067\|WP_141174031.1 | Serratia sp. SRS-8-S-2018 | helix-turn-helix domain-containing protein | Gammaproteobacteria | Enterobacterales |
| p1-125 | 1702204762\|QDL35717.1 | 269 | 99.257 | 100 | 0.0 | 538 | 1696703063\|WP_141174027.1 | Serratia sp. SRS-8-S-2018 | tyrosine-type recombinase/integrase | Gammaproteobacteria | Enterobacterales |
| p1-125 | 1702204763\|QDL35718.1 | 964 | 81.573 | 100 | 0.0 | 1562 | 1214086556\|WP_088733966.1 | Salmonella enterica | hypothetical protein | Gammaproteobacteria | Enterobacterales |
| p1-125 | 1702204764\|QDL35719.1 | 234 | 97.436 | 100 | 1.62e-170 | 478 | 1701419064\|WP_141971449.1 | Serratia marcescens | IS6 family transposase | Gammaproteobacteria | Enterobacterales |
| p1-125 | 1702204765\|QDL35720.1 | 539 | 99.629 | 100 | 0.0 | 1102 | 1217706749\|WP_089181293.1 | Serratia marcescens | ISL3 family transposase | Gammaproteobacteria | Enterobacterales |
| p1-125 | 1702204766\|QDL35721.1 | 96 | 43.548 | 65 | 2.68e-06 | 50.8 | 736894238\|WP_034893232.1 | Erwinia typographi | hypothetical protein | Gammaproteobacteria | Enterobacterales |
| p1-125 | 1702204767\|QDL35722.1 | 147 | 94.521 | 99 | 1.81e-95 | 283 | 1563830155\|WP_128884953.1 | Serratia marcescens | transposase | Gammaproteobacteria | Enterobacterales |
| p1-125 | 1702204768\|QDL35723.1 | - | - | - | - | - | - | - | hypothetical protein |  |  |
| p1-125 | 1702204769\|QDL35724.1 | 334 | 71.651 | 96 | 9.50e-170 | 484 | 1604901943\|WP_134691179.1 | Enterobacter hormaechei | transposase | Gammaproteobacteria | Enterobacterales |
| p1-125 | 1702204770\|QDL35725.1 | 158 | 72.327 | 100 | 5.25e-66 | 207 | 735673496\|WP_034165835.1 | Edwardsiella | hypothetical protein | Gammaproteobacteria | Enterobacterales |
| p1-125 | 1702204771QDL35726.1 | 407 | 96.560 | 100 | 0.0 | 825 | 1706435307\|WP_142816538.1 | Serratia liquefaciens | IS91 family transposase | Gammaproteobacteria | Enterobacterales |
| p1-125 | 1702204772\|QDL35727.1 | 315 | 99.683 | 100 | 0.0 | 650 | 1048493053\|emb\|SAY46468.1 | Serratia marcescens | Uncharacterised protein (plasmid) | Gammaproteobacteria | Enterobacterales |
| p1-125 | 1702204773\|QDL35728.1 | 174 | 97.701 | 100 | 1.57e-120 | 347 | 1228043993\|WP_094141452.1 | Serratia nematodiphila | phospholipase D family protein | Gammaproteobacteria | Enterobacterales |
| p1-125 | 1702204774\|QDL35729.1 | 86 | 98.837 | 100 | 9.09e-57 | 178 | 1728193250\|WP_147882599.1 | Serratia marcescens | hypothetical protein | Gammaproteobacteria | Enterobacterales |
| p1-125 | 1702204775\|QDL35730.1 | 179 | 99.441 | 100 | 9.30e-124 | 355 | 1725078688\|gb\|TXE57053.1 | Serratia nematodiphila | type IV conjugative transfer system lipoprotein TraV | Gammaproteobacteria | Enterobacterales |
| p1-125 | 1702204776\|QDL35731.1 | 97 | 100.000 | 100 | 1.56e-64 | 201 | 1048493138\|emb\|SAY46553.1 | Serratia marcescens | Uncharacterised protein (plasmid) | Gammaproteobacteria | Enterobacterales |
| p1-159 | 1702204780\|QDL35732.1 | 271 | 97.048 | 100 | 0.0 | 537 | 518284144\|WP_019454352.1 | Serratia marcescens | tyrosine-type recombinase/integrase | Gammaproteobacteria | Enterobacterales |
| p1-159 | 1702204781QDL35733.1 | 407 | 95.577 | 100 | 0.0 | 817 | 1705235570\|WP_142591839.1 | Serratia marcescens | IS91 family transposase | Gammaproteobacteria | Enterobacterales |
| p1-159 | 1702204782\|QDL35734.1 | 112 | 76.344 | 83 | 1.37e-48 | 159 | 1124442889\|WP_074729360.1 | Lonsdalea quercina | lipoprotein bor | Gammaproteobacteria | Enterobacterales |
| p1-159 | 1702204783\|QDL35735.1 | 219 | 59.070 | 97 | 4.71e-82 | 253 | 1168694659\|WP_080286911.1 | Serratia marcescens | response regulator transcription factor | Gammaproteobacteria | Enterobacterales |
| p1-159 | 1702204784\|QDL35736.1 | 242 | 85.892 | 99 | 3.34e-149 | 425 | 1175306272\|WP_081854067.1 | Edwardsiella | fimbria/pilus periplasmic chaperone | Gammaproteobacteria | Enterobacterales |
| p1-159 | 1702204785\|QDL35737.1 | 856 | 89.148 | 100 | 0.0 | 1549 | 917298375\|WP_051905087.1 | Edwardsiella | fimbrial biogenesis outer membrane usher protein | Gammaproteobacteria | Enterobacterales |
| p1-159 | 1702204786\|QDL35738.1 | 158 | 72.327 | 100 | 5.25e-66 | 207 | 735673496\|WP_034165835.1 | Edwardsiella | hypothetical protein | Gammaproteobacteria | Enterobacterales |
| p1-159 | 1702204787\|QDL35739.1 | 235 | 26.471 | 97 | 3.17e-10 | 68.9 | 1214890205\|WP_088901147.1 | Pantoea sp. AMG 501 | hypothetical protein | Gammaproteobacteria | Enterobacterales |
| p1-159 | 1702204788\|QDL35740.1 | 310 | 99.355 | 100 | 0.0 | 629 | 503838580\|WP_014072574.1 | Serratia marcescens | Rpn family recombination-promoting nuclease/putative transposase | Gammaproteobacteria | Enterobacterales |
| p1-159 | 1702204789\|QDL35741.1 | 294 | 96.939 | 100 | 0.0 | 593 | 503838579\|WP_014072573.1 | Serratia marcescens | DUF3644 domain-containing protein | Gammaproteobacteria | Enterobacterales |
| p1-159 | 1702204790\|QDL35742.1 | 247 | 100.000 | 100 | 2.96e-177 | 496 | 641641430\|emb\|CDQ52079.1 | Klebsiella pneumoniae | unnamed protein product | Gammaproteobacteria | Enterobacterales |
| p1-159 | 1702204791QDL35743.1 | 207 | 100.000 | 100 | 1.91e-145 | 413 | 641641429\|emb\|CDQ52078.1 | Klebsiella pneumoniae | unnamed protein product | Gammaproteobacteria | Enterobacterales |
| p1-159 | 1702204792\|QDL35744.1 | 80 | 98.750 | 100 | 6.84e-50 | 160 | 1093660712\|WP_070914200.1 | Serratia marcescens | hypothetical protein | Gammaproteobacteria | Enterobacterales |
| p1-159 | 1702204793\|QDL35745.1 | 96 | 98.958 | 100 | 8.37e-62 | 192 | 983104229\|WP_060441998.1 | Serratia | hypothetical protein | Gammaproteobacteria | Enterobacterales |
| p1-159 | 1702204794\|QDL35746.1 | 394 | 98.985 | 100 | 0.0 | 820 | 1217741246\|WP_089186942.1 | Serratia marcescens | pyridoxal phosphate-dependent aminotransferase | Gammaproteobacteria | Enterobacterales |
| p1-159 | 1702204795\|QDL35747.1 | 479 | 99.165 | 100 | 0.0 | 942 | 1701420785\|WP_141973170.1 | Serratia marcescens | Na+/H+ antiporter NhaC | Gammaproteobacteria | Enterobacterales |
| p1-159 | 1702204796\|QDL35748.1 | 336 | 99.405 | 100 | 0.0 | 690 | 1217757499\|WP_089197795.1 | Serratia marcescens | LacI family DNA-binding transcriptional regulator | Gammaproteobacteria | Enterobacterales |
| p1-159 | 1702204797\|QDL35749.1 | 70 | 98.571 | 100 | 1.64e-40 | 136 | 1706435380\|WP_142816595.1 | Serratia liquefaciens | hypothetical protein | Gammaproteobacteria | Enterobacterales |
| p1-159 | 1702204798\|QDL35750.1 | 214 | 99.533 | 100 | 4.08e-157 | 442 | 983104233\|WP_060442002.1 | Serratia marcescens | DNA methylase | Gammaproteobacteria | Enterobacterales |
| p1-159 | 1702204799\|QDL35751.1 | 73 | 95.890 | 100 | 9.38e-44 | 144 | 983093201WP_060432392.1 | Serratia | hypothetical protein | Gammaproteobacteria | Enterobacterales |
| p1-159 | 1702204800\|QDL35752.1 | 141 | 99.291 | 100 | 1.03e-100 | 294 | 1093660734\|WP_070914213.1 | Serratia marcescens | antirestriction protein | Gammaproteobacteria | Enterobacterales |
| p1-159 | 1702204801QDL35753.1 | 132 | 99.242 | 100 | 4.24e-92 | 271 | 1093660737\|WP_070914215.1 | Serratia marcescens | DUF1380 domain-containing protein | Gammaproteobacteria | Enterobacterales |
| p1-159 | 1702204802\|QDL35754.1 | 96 | 97.917 | 100 | 3.77e-64 | 198 | 1093660740\|WP_070914217.1 | Serratia marcescens | ribulokinase | Gammaproteobacteria | Enterobacterales |
| p1-159 | 1702204803\|QDL35755.1 | 685 | 95.766 | 100 | 0.0 | 1340 | 1217757488\|WP_089197790.1 | Serratia marcescens | ParC | Gammaproteobacteria | Enterobacterales |
| p1-159 | 1702204804\|QDL35756.1 | 146 | 98.630 | 100 | 2.41e-102 | 298 | 1741177806\|WP_149571800.1 | Serratia marcescens | conjugation system SOS inhibitor PsiB | Gammaproteobacteria | Enterobacterales |
| p1-159 | 1702204805\|QDL35757.1 | 219 | 98.630 | 100 | 2.66e-154 | 437 | 1169075275\|WP_080437540.1 | Serratia marcescens | hypothetical protein | Gammaproteobacteria | Enterobacterales |
| p1-159 | 1702204806\|QDL35758.1 | 154 | 94.156 | 100 | 2.17e-105 | 306 | 1728193241WP_147882590.1 | Serratia marcescens | protein TraE | Gammaproteobacteria | Enterobacterales |
| p1-159 | 1702204807\|QDL35759.1 | 115 | 99.130 | 100 | 3.21e-79 | 237 | 1728193240\|WP_147882589.1 | Serratia marcescens | hypothetical protein | Gammaproteobacteria | Enterobacterales |
| p1-159 | 1702204808\|QDL35760.1 | 265 | 99.245 | 100 | 0.0 | 548 | 1728193238\|WP_147882587.1 | Serratia marcescens | N-6 DNA methylase | Gammaproteobacteria | Enterobacterales |
| p1-159 | 1702204809\|QDL35761.1 | 123 | 98.374 | 100 | 3.84e-86 | 255 | 757523327\|WP_042785948.1 | Serratia sp. SCBI | hypothetical protein | Gammaproteobacteria | Enterobacterales |
| p1-159 | 1702204810\|QDL35762.1 | 275 | 99.636 | 100 | 0.0 | 572 | 1397621743\|WP_110147657.1 | Serratia marcescens | DUF945 domain-containing protein | Gammaproteobacteria | Enterobacterales |
| p1-159 | 1702204811QDL35763.1 | 98 | 96.939 | 100 | 1.50e-65 | 201 | 1706435269\|WP_142816504.1 | Serratia liquefaciens | hypothetical protein | Gammaproteobacteria | Enterobacterales |
| p1-159 | 1702204812\|QDL35764.1 | 158 | 98.734 | 100 | 8.02e-113 | 326 | 1741178022\|WP_149572016.1 | Serratia marcescens | transglycosylase SLT domain-containing protein | Gammaproteobacteria | Enterobacterales |
| p1-159 | 1702204813\|QDL35765.1 | 131 | 98.473 | 100 | 9.12e-89 | 263 | 1741178023\|WP_149572017.1 | Serratia marcescens | relaxosome protein TraM | Gammaproteobacteria | Enterobacterales |
| p1-159 | 1702204814\|QDL35766.1 | 571 | 98.424 | 100 | 0.0 | 1151 | 1741178024\|WP_149572018.1 | Serratia marcescens | response regulator | Gammaproteobacteria | Enterobacterales |
| p1-159 | 1702204815\|QDL35767.1 | 164 | 98.780 | 100 | 2.22e-116 | 335 | 1741178025\|WP_149572019.1 | Serratia marcescens | oxidoreductase | Gammaproteobacteria | Enterobacterales |
| p1-159 | 1702204816\|QDL35768.1 | 331 | 94.864 | 100 | 0.0 | 650 | 1741178026\|WP_149572020.1 | Serratia marcescens | glycosyltransferase family 9 protein | Gammaproteobacteria | Enterobacterales |
| p1-159 | 1702204817\|QDL35769.1 | 280 | 98.929 | 100 | 0.0 | 566 | 1741178027\|WP_149572021.1 | Serratia marcescens | response regulator | Gammaproteobacteria | Enterobacterales |
| p1-159 | 1702204818\|QDL35770.1 | 128 | 97.656 | 100 | 3.92e-87 | 258 | 1178687821WP_083196351.1 | Serratia sp. 14-2641 | TraY domain-containing protein | Gammaproteobacteria | Enterobacterales |
| p1-159 | 1702204819\|QDL35771.1 | 116 | 97.414 | 100 | 1.03e-70 | 216 | 1046615246\|WP_065684416.1 | Serratia sp. 14-2641 | type IV conjugative transfer system pilin TraA | Gammaproteobacteria | Enterobacterales |
| p1-159 | 1702204820\|QDL35772.1 | 101 | 98.020 | 100 | 7.91e-67 | 205 | 1046615248\|WP_065684418.1 | Serratia | type IV conjugative transfer system protein TraL | Gammaproteobacteria | Enterobacterales |
| p1-159 | 1702204821QDL35773.1 | 188 | 98.936 | 100 | 2.51e-135 | 385 | 1741178030\|WP_149572024.1 | Serratia marcescens | type IV conjugative transfer system protein TraE | Gammaproteobacteria | Enterobacterales |
| p1-159 | 1702204822\|QDL35774.1 | 247 | 98.785 | 100 | 1.06e-173 | 487 | 1741178031WP_149572025.1 | Serratia marcescens | type-F conjugative transfer system secretin TraK | Gammaproteobacteria | Enterobacterales |
| p1-159 | 1702204823\|QDL35775.1 | 464 | 99.138 | 100 | 0.0 | 936 | 1741178032\|WP_149572026.1 | Serratia marcescens | F-type conjugal transfer pilus assembly protein TraB | Gammaproteobacteria | Enterobacterales |
| p1-159 | 1702204824\|QDL35776.1 | 128 | 88.976 | 99 | 6.60e-74 | 225 | 1706435263\|WP_142816499.1 | Serratia liquefaciens | hypothetical protein | Gammaproteobacteria | Enterobacterales |
| p1-159 | 1702204825\|QDL35777.1 | 87 | 100.000 | 82 | 6.75e-44 | 145 | 1706435422\|WP_142816620.1 | Serratia liquefaciens | hypothetical protein | Gammaproteobacteria | Enterobacterales |
| p1-159 | 1702204826\|QDL35778.1 | 162 | 81.818 | 100 | 4.41e-82 | 248 | 1046615254\|WP_065684424.1 | Serratia sp. 14-2641 | hypothetical protein | Gammaproteobacteria | Enterobacterales |
| p1-159 | 1702204827\|QDL35779.1 | 161 | 92.308 | 89 | 5.52e-91 | 271 | 1741178007\|WP_149572001.1 | Serratia marcescens | hypothetical protein | Gammaproteobacteria | Enterobacterales |
| p1-159 | 1702204828\|QDL35780.1 | 115 | 99.130 | 100 | 4.50e-76 | 229 | 1741178008\|WP_149572002.1 | Serratia marcescens | type-F conjugative transfer system protein TrbI | Gammaproteobacteria | Enterobacterales |
| p1-159 | 1702204829\|QDL35781.1 | 218 | 98.165 | 100 | 7.58e-155 | 437 | 1046615258\|WP_065684428.1 | Serratia sp. 14-2641 | type-F conjugative transfer system protein TraW | Gammaproteobacteria | Enterobacterales |
| p1-159 | 1702204830\|QDL35782.1 | 335 | 99.701 | 100 | 0.0 | 690 | 1741178009\|WP_149572003.1 | Serratia marcescens | conjugal transfer pilus assembly protein TraU | Gammaproteobacteria | Enterobacterales |
| p1-159 | 1702204831QDL35783.1 | 209 | 95.215 | 100 | 7.28e-146 | 414 | 1442527611WP_115160177.1 | Serratia fonticola | type-F conjugative transfer system pilin assembly protein TrbC | Gammaproteobacteria | Enterobacterales |
| p1-159 | 1702204832\|QDL35784.1 | 625 | 75.447 | 98 | 0.0 | 987 | 1160408662\|WP_079450618.1 | Serratia marcescens | type-F conjugative transfer system mating-pair stabilization protein TraN | Gammaproteobacteria | Enterobacterales |
| p1-159 | 1702204833\|QDL35785.1 | 247 | 95.142 | 100 | 5.02e-173 | 485 | 1046615262\|WP_065684432.1 | Serratia sp. 14-2641 | type-F conjugative transfer system pilin assembly protein TraF | Gammaproteobacteria | Enterobacterales |
| p1-159 | 1702204834\|QDL35786.1 | 87 | 96.552 | 100 | 1.78e-54 | 172 | 1442527614\|WP_115160180.1 | Serratia fonticola | type-F conjugative transfer system pilin chaperone TraQ | Gammaproteobacteria | Enterobacterales |
| p1-159 | 1702204835\|QDL35787.1 | 188 | 99.468 | 100 | 1.73e-135 | 385 | 1741178010\|WP_149572004.1 | Serratia marcescens | type-F conjugative transfer system pilin assembly thiol-disulfide isomerase TrbB | Gammaproteobacteria | Enterobacterales |
| p1-159 | 1702204836\|QDL35788.1 | 942 | 92.569 | 100 | 0.0 | 1800 | 1046615266\|WP_065684436.1 | Serratia sp. 14-2641 | conjugal transfer mating pair stabilization protein TraG | Gammaproteobacteria | Enterobacterales |
| p1-159 | 1702204837\|QDL35789.1 | 171 | 63.158 | 100 | 1.76e-73 | 227 | 1741178013\|WP_149572007.1 | Serratia marcescens | hypothetical protein | Gammaproteobacteria | Enterobacterales |
| p1-159 | 1702204838\|QDL35790.1 | 728 | 97.817 | 100 | 0.0 | 1471 | 1741178014\|WP_149572008.1 | Serratia marcescens | type IV conjugative transfer system coupling protein TraD | Gammaproteobacteria | Enterobacterales |
| p1-159 | 1702204839\|QDL35791.1 | 1748 | 97.769 | 100 | 0.0 | 3458 | 1741178015\|WP_149572009.1 | Serratia marcescens | conjugative transfer relaxase/helicase TraI | Gammaproteobacteria | Enterobacterales |
| p1-159 | 1702204840\|QDL35792.1 | 245 | 96.327 | 100 | 4.10e-167 | 470 | 1741178016\|WP_149572010.1 | Serratia marcescens | type-F conjugative transfer system pilin acetylase TraX | Gammaproteobacteria | Enterobacterales |
| p1-159 | 1702204841QDL35793.1 | 186 | 89.785 | 100 | 6.70e-119 | 343 | 1046615273\|WP_065684443.1 | Serratia sp. 14-2641 | phospholipase D family protein | Gammaproteobacteria | Enterobacterales |
| p1-159 | 1702204842\|QDL35794.1 | 83 | 96.386 | 100 | 4.09e-51 | 164 | 951151132\|WP_057631561.1 | Yersinia | replication regulatory protein RepA | Gammaproteobacteria | Enterobacterales |
| p1-159 | 1702204843\|QDL35795.1 | 315 | 99.048 | 100 | 0.0 | 645 | 757523290\|WP_042785914.1 | Serratia sp. SCBI | hypothetical protein | Gammaproteobacteria | Enterobacterales |
| p1-159 | 1702204844\|QDL35796.1 | 81 | 100.000 | 88 | 1.18e-44 | 147 | 1706435451WP_142816638.1 | Serratia liquefaciens | hypothetical protein | Gammaproteobacteria | Enterobacterales |
| p1-159 | 1702204845\|QDL35797.1 | 407 | 99.017 | 100 | 0.0 | 845 | 983153798\|WP_060452185.1 | Serratia marcescens | IS91 family transposase | Gammaproteobacteria | Enterobacterales |
| p1-159 | 1702204846\|QDL35798.1 | 173 | 95.376 | 100 | 1.54e-113 | 329 | 742395693\|WP_038874818.1 | Serratia | superoxide dismutase | Gammaproteobacteria | Enterobacterales |
| p1-159 | 1702204847\|QDL35799.1 | 148 | 97.297 | 100 | 3.61e-102 | 298 | 960872278\|WP_058344447.1 | Salmonella enterica | C-lysozyme inhibitor | Gammaproteobacteria | Enterobacterales |
| p1-159 | 1702204848\|QDL35800.1 | 231 | 94.372 | 100 | 2.57e-161 | 454 | 1706435303\|WP_142816534.1 | Serratia liquefaciens | IS6 family transposase | Gammaproteobacteria | Enterobacterales |
| p1-159 | 1702204849\|QDL35801.1 | 63 | 81.250 | 51 | 6.56e-09 | 55.5 | 312171360\|emb\|CBX79619.1 | Erwinia amylovora | hypothetical protein predicted by Glimmer/Critica | Gammaproteobacteria | Enterobacterales |
| p1-159 | 1702204850\|QDL35802.1 | 301 | 60.465 | 100 | 2.83e-130 | 382 | 823310830\|WP_047065314.1 | Klebsiella aerogenes | fimbrial protein | Gammaproteobacteria | Enterobacterales |
| p1-159 | 1702204851QDL35803.1 | 168 | 68.452 | 100 | 2.35e-73 | 227 | 490205152\|WP_004103586.1 | Klebsiella oxytoca | type 1 fimbrial protein | Gammaproteobacteria | Enterobacterales |
| p1-159 | 1702204852\|QDL35804.1 | 875 | 72.000 | 100 | 0.0 | 1311 | 1690790159\|WP_139536434.1 | Klebsiella michiganensis | fimbrial biogenesis usher protein | Gammaproteobacteria | Enterobacterales |
| p1-159 | 1702204853\|QDL35805.1 | 234 | 73.077 | 100 | 3.13e-121 | 353 | 1033027848\|WP_064343785.1 | Klebsiella oxytoca | fimbria/pilus periplasmic chaperone | Gammaproteobacteria | Enterobacterales |
| p1-159 | 1702204854\|QDL35806.1 | 179 | 55.618 | 99 | 4.48e-67 | 212 | 1705325248\|WP_142670546.1 | Klebsiella oxytoca | type 1 fimbrial protein | Gammaproteobacteria | Enterobacterales |
| p1-159 | 1702204855\|QDL35807.1 | 184 | 100.000 | 100 | 4.47e-127 | 364 | 1702204855\|gb\|QDL35807.1 | Serratia liquefaciens | type-1 fimbrial protein subunit A (plasmid) | Gammaproteobacteria | Enterobacterales |
| p1-159 | 1702204856\|QDL35808.1 | 307 | 77.451 | 99 | 3.95e-173 | 491 | 1356493626\|WP_105230950.1 | Serratia sp. MYb239 | LysR family transcriptional regulator | Gammaproteobacteria | Enterobacterales |
| p1-159 | 1702204857\|QDL35809.1 | 231 | 94.372 | 100 | 2.57e-161 | 454 | 1706435303\|WP_142816534.1 | Serratia liquefaciens | IS6 family transposase | Gammaproteobacteria | Enterobacterales |
| p1-159 | 1702204858\|QDL35810.1 | 246 | 98.780 | 100 | 2.11e-179 | 501 | 1166258654\|WP_079964061.1 | Enterobacterales | carbonic anhydrase | Gammaproteobacteria | Enterobacterales |
| p1-159 | 1702204859\|QDL35811.1 | 54 | 97.959 | 91 | 1.14e-26 | 105 | 1217753789\|WP_089196573.1 | Serratia marcescens | transposase | Gammaproteobacteria | Enterobacterales |
| p1-159 | 1702204860\|QDL35812.1 | 160 | 96.855 | 99 | 6.19e-110 | 318 | 1374253384\|WP_107227038.1 | Serratia | type VI secretion system tube protein Hcp | Gammaproteobacteria | Enterobacterales |
| p1-159 | 1702204861QDL35813.1 | 160 | 83.750 | 100 | 1.08e-91 | 273 | 1184549476\|WP_085117366.1 | Serratia proteamaculans | type VI secretion system tube protein Hcp | Gammaproteobacteria | Enterobacterales |
| p1-159 | 1702204862\|QDL35814.1 | 237 | 74.359 | 99 | 6.74e-129 | 373 | 1720118543\|WP_145957281.1 | Serratia marcescens | hypothetical protein | Gammaproteobacteria | Enterobacterales |
| p1-159 | 1702204863\|QDL35815.1 | 232 | 94.397 | 100 | 1.38e-161 | 455 | 1690549966\|WP_139385901.1 | Salmonella enterica | IS1 family transposase | Gammaproteobacteria | Enterobacterales |
| p1-159 | 1702204864\|QDL35816.1 | 86 | 50.000 | 100 | 3.59e-23 | 94.0 | 1046616546\|WP_065685716.1 | Serratia sp. 14-2641 | DUF1471 domain-containing protein | Gammaproteobacteria | Enterobacterales |
| p1-159 | 1702204865\|QDL35817.1 | 231 | 94.372 | 100 | 2.57e-161 | 454 | 1706435303\|WP_142816534.1 | Serratia liquefaciens | IS6 family transposase | Gammaproteobacteria | Enterobacterales |
| p1-159 | 1702204866\|QDL35818.1 | 63 | 96.825 | 100 | 6.93e-36 | 124 | 751569715\|WP_041038610.1 | Serratia marcescens | hypothetical protein | Gammaproteobacteria | Enterobacterales |
| p1-159 | 1702204867\|QDL35819.1 | 97 | 98.969 | 100 | 1.00e-62 | 194 | 1168765437\|WP_080335483.1 | Enterobacterales | hypothetical protein | Gammaproteobacteria | Enterobacterales |
| p1-159 | 1702204868\|QDL35820.1 | 283 | 99.293 | 100 | 0.0 | 585 | 1728193246\|WP_147882595.1 | Serratia marcescens | AAA family ATPase | Gammaproteobacteria | Enterobacterales |
| p1-159 | 1702204869\|QDL35821.1 | 146 | 97.945 | 100 | 2.09e-102 | 298 | 1047619780\|gb\|OCN20785.1 | Serratia marcescens | recombinase | Gammaproteobacteria | Enterobacterales |
| p1-159 | 1702204870\|QDL35822.1 | 258 | 96.124 | 100 | 5.14e-178 | 499 | 1725078569\|gb\|TXE56934.1 | Serratia nematodiphila | hypothetical protein FOT58_19390 | Gammaproteobacteria | Enterobacterales |
| p1-159 | 1702204871QDL35823.1 | 83 | 98.795 | 100 | 1.07e-52 | 168 | 983153774\|WP_060452161.1 | Serratia marcescens | type II toxin-antitoxin system Phd/YefM family antitoxin | Gammaproteobacteria | Enterobacterales |
| p1-159 | 1702204872\|QDL35824.1 | 95 | 98.947 | 100 | 6.30e-63 | 194 | 491068231WP_004929861.1 | Serratia | type II toxin-antitoxin system RelE/ParE family toxin | Gammaproteobacteria | Enterobacterales |
| p1-159 | 1702204873\|QDL35825.1 | 145 | 97.674 | 89 | 1.13e-87 | 261 | 1048493146\|emb\|SAY46561.1 | Serratia marcescens | Uncharacterised protein (plasmid) | Gammaproteobacteria | Enterobacterales |
| p1-159 | 1702204874\|QDL35826.1 | 123 | 96.748 | 100 | 2.32e-85 | 253 | 1741177942\|WP_149571936.1 | Serratia marcescens | dehydrogenase | Gammaproteobacteria | Enterobacterales |
| p1-159 | 1702204875\|QDL35827.1 | 77 | 95.556 | 100 | 8.49e-22 | 90.5 | 1712695291WP_143779255.1 | Serratia marcescens | hypothetical protein | Gammaproteobacteria | Enterobacterales |
| p1-159 | 1702204876\|QDL35828.1 | 274 | 92.701 | 100 | 0.0 | 529 | 1712695292\|WP_143779256.1 | Serratia marcescens | hypothetical protein | Gammaproteobacteria | Enterobacterales |
| p1-159 | 1702204877\|QDL35829.1 | 308 | 95.455 | 100 | 0.0 | 619 | 1712695293\|WP_143779257.1 | Serratia marcescens | hypothetical protein | Gammaproteobacteria | Enterobacterales |
| p1-159 | 1702204878\|QDL35830.1 | 243 | 97.942 | 100 | 2.78e-178 | 498 | 1261234266\|WP_097766947.1 | Escherichia coli | hypothetical protein | Gammaproteobacteria | Enterobacterales |
| p1-159 | 1702204879\|QDL35831.1 | 310 | 100.000 | 100 | 0.0 | 617 | 1702204879\|gb\|QDL35831.1 | Serratia liquefaciens | hypothetical protein EGO53_29140 (plasmid) | Gammaproteobacteria | Enterobacterales |
| p1-159 | 1702204880\|QDL35832.1 | - | - | - | - | - | - | - | hypothetical protein |  |  |
| p1-159 | 1702204881QDL35833.1 | 192 | 99.479 | 100 | 2.38e-135 | 385 | 1119710406\|WP_072628494.1 | Serratia marcescens | recombinase family protein | Gammaproteobacteria | Enterobacterales |
| p1-159 | 1702204882\|QDL35834.1 | 1015 | 96.548 | 99 | 0.0 | 2038 | 491106464\|WP_004966289.1 | Serratia | Tn3 family transposase | Gammaproteobacteria | Enterobacterales |
| p1-159 | 1702204883\|QDL35835.1 | 369 | 98.916 | 100 | 0.0 | 742 | 1701420794\|WP_141973179.1 | Serratia marcescens | recombinase RecA | Gammaproteobacteria | Enterobacterales |
| p1-159 | 1702204884\|QDL35836.1 | 60 | 53.333 | 100 | 8.31e-13 | 66.2 | 752771616\|WP_041418727.1 | Serratia proteamaculans | hypothetical protein | Gammaproteobacteria | Enterobacterales |
| p1-159 | 1702204885\|QDL35837.1 | 193 | 86.010 | 100 | 5.51e-122 | 352 | 37518403\|emb\|CAD58555.1 | Yersinia enterocolitica | hypothetical protein (plasmid) | Gammaproteobacteria | Enterobacterales |
| p1-159 | 1702204886\|QDL35838.1 | 184 | 90.217 | 100 | 1.21e-120 | 348 | 514076109\|WP_016528967.1 | Klebsiella | hypothetical protein | Gammaproteobacteria | Enterobacterales |
| p1-159 | 1702204887\|QDL35839.1 | 167 | 89.222 | 100 | 7.59e-108 | 314 | 657865612\|WP_029571257.1 | Pantoea | hypothetical protein | Gammaproteobacteria | Enterobacterales |
| p1-159 | 1702204888\|QDL35840.1 | 87 | 98.851 | 100 | 8.22e-56 | 176 | 942448004\|WP_055313622.1 | Serratia marcescens | hypothetical protein | Gammaproteobacteria | Enterobacterales |
| p1-159 | 1702204889\|QDL35841.1 | 53 | 96.226 | 100 | 9.99e-28 | 103 | 1741178037\|WP_149572031.1 | Serratia marcescens | TraY domain-containing protein | Gammaproteobacteria | Enterobacterales |
| p1-159 | 1702204890\|QDL35842.1 | 179 | 98.324 | 100 | 4.30e-125 | 358 | 1741178038\|WP_149572032.1 | Serratia marcescens | type IV conjugative transfer system lipoprotein TraV | Gammaproteobacteria | Enterobacterales |
| p1-159 | 1702204891QDL35843.1 | 56 | 89.286 | 100 | 9.60e-28 | 103 | 1741178039\|WP_149572033.1 | Serratia marcescens | Clp protease | Gammaproteobacteria | Enterobacterales |
| p1-159 | 1702204892\|QDL35844.1 | 454 | 98.678 | 100 | 0.0 | 928 | 1741178011WP_149572005.1 | Serratia marcescens | F-type conjugal transfer protein TraH | Gammaproteobacteria | Enterobacterales |
| p1-159 | 1702204893\|QDL35845.1 | 175 | 66.857 | 100 | 1.78e-79 | 243 | 1398359041WP_110275411.1 | Klebsiella oxytoca | type 1 fimbrial protein | Gammaproteobacteria | Enterobacterales |
| p1-159 | 1702204894\|QDL35846.1 | 181 | 64.641 | 100 | 7.70e-75 | 231 | 1575621247\|WP_130017302.1 | Serratia marcescens | hypothetical protein | Gammaproteobacteria | Enterobacterales |
| p1-159 | 1702204895\|QDL35847.1 | 96 | 96.875 | 100 | 1.42e-63 | 196 | 1228044039\|WP_094141498.1 | Serratia nematodiphila | ribulokinase | Gammaproteobacteria | Enterobacterales |
| p1-159 | 1702204896\|QDL35848.1 | 682 | 96.481 | 100 | 0.0 | 1331 | 1741176717\|WP_149570786.1 | Serratia marcescens | ParC | Gammaproteobacteria | Enterobacterales |

**Supplementary Table 3.** *Serratia* core genome of protein families.

| **Family** | **Description** | **Number of genes (paralogous)** | | | | | | | | | | | | | | | | | | | | | | | | | | | | | | | | | | |
| --- | --- | --- | --- | --- | --- | --- | --- | --- | --- | --- | --- | --- | --- | --- | --- | --- | --- | --- | --- | --- | --- | --- | --- | --- | --- | --- | --- | --- | --- | --- | --- | --- | --- | --- | --- | --- |
|  |  | **S_ufop_01_chr** | **S_marcescens_DB11_chr** | **S_odorifera_DSM4582_chr** | **S_plymuthica_3Re418_chr** | **S_marcescens_RSC14_chr** | **S_marcescens_CAV1492_chr** | **S_symbiotica_STs_chr** | **S_marcescens_B3R3_chr** | **S_plymuthica_4Rx13_chr** | **S_marcescens_WW4_chr** | **S_marcences_B3R3_chr** | **S_plymuthica_3RP8_chr** | **S_symbiotica_cinaracedri_chr** | **S_multitudinisentens_RB25_chr** | **S_sp_YD25_chr** | **S_plymuthica_S13_chr** | **S_sp_FS14_chr** | **S_sp_AS13_chr** | **S_ureilytica_Lr54_chr** | **S_marcescens_SmUNAM836_chr** | **S_liquefaciens_HUMV21_chr** | **S_marcescens_FGI94_chr** | **S_fonticola_GS2_chr** | **S_plymuthica_PRI2c_chr** | **S_ficaria_NBRC102596_chr** | **S_sp_SCBI_chr** | **S_proteamaculans_568_chr** | **S_liquefaciens_ATCC27592_chr** | **S_plymuthica_AS9_chr** | **S_liquefaciens_FDAARGOS125_chr** | **S_nematodiphila_DZ0503SBS1_chr** | **S_sp_AS12_chr** | **S_marcescens_SM39_chr** | **S_rubidaea_1122_chr** | **S_fonticola_DSM4576_chr** |
| 14 | Periplasmic serine endoprotease DegP | 2 | 2 | 2 | 2 | 2 | 2 | 1 | 2 | 2 | 2 | 2 | 2 | 1 | 2 | 2 | 2 | 2 | 2 | 2 | 2 | 2 | 2 | 3 | 2 | 2 | 2 | 2 | 2 | 2 | 2 | 2 | 2 | 2 | 2 | 2 |
| 19 | Elongation factor G | 2 | 2 | 2 | 2 | 2 | 2 | 1 | 2 | 2 | 2 | 2 | 2 | 1 | 1 | 2 | 2 | 2 | 2 | 2 | 2 | 2 | 2 | 2 | 2 | 2 | 2 | 2 | 2 | 2 | 2 | 2 | 2 | 2 | 2 | 2 |
| 20 | Peptidoglycan synthase FtsI | 2 | 2 | 2 | 2 | 2 | 2 | 1 | 2 | 2 | 2 | 2 | 2 | 1 | 1 | 2 | 2 | 2 | 2 | 2 | 2 | 2 | 2 | 2 | 2 | 2 | 2 | 2 | 2 | 2 | 2 | 2 | 2 | 2 | 2 | 2 |
| 54 | Pyruvate dehydrogenase E1 component | 1 | 2 | 1 | 2 | 2 | 2 | 1 | 1 | 1 | 2 | 1 | 2 | 1 | 1 | 2 | 2 | 2 | 2 | 2 | 1 | 1 | 1 | 1 | 1 | 1 | 2 | 1 | 1 | 2 | 1 | 1 | 2 | 1 | 1 | 1 |
| 56 | Branched-chain amino acid transport system 2 carrier protein | 1 | 2 | 1 | 1 | 2 | 2 | 1 | 2 | 1 | 2 | 2 | 1 | 1 | 1 | 2 | 1 | 2 | 1 | 2 | 2 | 1 | 1 | 1 | 1 | 2 | 2 | 2 | 1 | 1 | 1 | 2 | 1 | 2 | 1 | 1 |
| 76 | 1-deoxy-D-xylulose-5-phosphate synthase | 1 | 1 | 1 | 2 | 1 | 1 | 1 | 1 | 2 | 1 | 1 | 2 | 1 | 1 | 1 | 2 | 2 | 2 | 1 | 1 | 1 | 1 | 1 | 2 | 1 | 1 | 1 | 1 | 2 | 1 | 1 | 2 | 1 | 1 | 1 |
| 83 | Outer membrane protein assembly factor BamA | 1 | 1 | 1 | 2 | 1 | 1 | 1 | 1 | 2 | 1 | 1 | 2 | 1 | 1 | 1 | 2 | 1 | 2 | 1 | 1 | 1 | 1 | 1 | 1 | 1 | 1 | 1 | 1 | 2 | 1 | 1 | 2 | 1 | 1 | 1 |
| 101 | Single-stranded DNA-binding protein | 1 | 1 | 1 | 1 | 1 | 1 | 1 | 1 | 1 | 1 | 1 | 1 | 1 | 1 | 1 | 1 | 1 | 1 | 1 | 1 | 1 | 1 | 4 | 1 | 1 | 1 | 1 | 1 | 1 | 1 | 1 | 1 | 1 | 1 | 1 |
| 111 | Serine hydroxymethyltransferase | 1 | 1 | 1 | 1 | 1 | 1 | 1 | 1 | 1 | 1 | 1 | 1 | 1 | 1 | 1 | 1 | 1 | 1 | 1 | 1 | 1 | 2 | 1 | 1 | 1 | 1 | 1 | 1 | 1 | 1 | 1 | 1 | 1 | 2 | 2 |
| 119 | Glucose-6-phosphate isomerase | 1 | 1 | 1 | 1 | 1 | 1 | 1 | 1 | 1 | 1 | 1 | 1 | 1 | 1 | 1 | 1 | 1 | 1 | 1 | 1 | 1 | 1 | 2 | 1 | 1 | 1 | 1 | 1 | 1 | 1 | 1 | 1 | 1 | 1 | 1 |
| 120 | Glycerol-3-phosphate acyltransferase | 1 | 1 | 1 | 1 | 1 | 1 | 1 | 1 | 1 | 1 | 1 | 1 | 1 | 1 | 1 | 1 | 1 | 1 | 1 | 1 | 1 | 1 | 2 | 1 | 1 | 1 | 1 | 1 | 1 | 1 | 1 | 1 | 1 | 1 | 1 |
| 121 | tRNA-dihydrouridine(20/20a) synthase | 1 | 1 | 1 | 1 | 1 | 1 | 1 | 1 | 1 | 1 | 1 | 1 | 1 | 1 | 1 | 1 | 1 | 1 | 1 | 1 | 1 | 1 | 2 | 1 | 1 | 1 | 1 | 1 | 1 | 1 | 1 | 1 | 1 | 1 | 1 |
| 122 | Replicative DNA helicase | 1 | 1 | 1 | 1 | 1 | 1 | 1 | 1 | 1 | 1 | 1 | 1 | 1 | 1 | 1 | 1 | 1 | 1 | 1 | 1 | 1 | 1 | 2 | 1 | 1 | 1 | 1 | 1 | 1 | 1 | 1 | 1 | 1 | 1 | 1 |
| 128 | Inner membrane protein YqjA | 1 | 1 | 1 | 1 | 1 | 1 | 1 | 1 | 1 | 1 | 1 | 1 | 1 | 1 | 1 | 1 | 1 | 1 | 1 | 1 | 1 | 1 | 2 | 1 | 1 | 1 | 1 | 1 | 1 | 1 | 1 | 1 | 1 | 1 | 1 |
| 129 | Penicillin-binding protein activator LpoA | 1 | 1 | 1 | 1 | 1 | 1 | 1 | 1 | 1 | 1 | 1 | 1 | 1 | 1 | 1 | 1 | 1 | 1 | 1 | 1 | 1 | 1 | 2 | 1 | 1 | 1 | 1 | 1 | 1 | 1 | 1 | 1 | 1 | 1 | 1 |
| 130 | UDP-N-acetylglucosamine 1-carboxyvinyltransferase | 1 | 1 | 1 | 1 | 1 | 1 | 1 | 1 | 1 | 1 | 1 | 1 | 1 | 1 | 1 | 1 | 1 | 1 | 1 | 1 | 1 | 1 | 2 | 1 | 1 | 1 | 1 | 1 | 1 | 1 | 1 | 1 | 1 | 1 | 1 |
| 131 | Acid stress protein IbaG | 1 | 1 | 1 | 1 | 1 | 1 | 1 | 1 | 1 | 1 | 1 | 1 | 1 | 1 | 1 | 1 | 1 | 1 | 1 | 1 | 1 | 1 | 2 | 1 | 1 | 1 | 1 | 1 | 1 | 1 | 1 | 1 | 1 | 1 | 1 |
| 132 | putative phospholipid ABC transporter-binding protein MlaD | 1 | 1 | 1 | 1 | 1 | 1 | 1 | 1 | 1 | 1 | 1 | 1 | 1 | 1 | 1 | 1 | 1 | 1 | 1 | 1 | 1 | 1 | 2 | 1 | 1 | 1 | 1 | 1 | 1 | 1 | 1 | 1 | 1 | 1 | 1 |
| 133 | Arabinose 5-phosphate isomerase KdsD | 1 | 1 | 1 | 1 | 1 | 1 | 1 | 1 | 1 | 1 | 1 | 1 | 1 | 1 | 1 | 1 | 1 | 1 | 1 | 1 | 1 | 1 | 2 | 1 | 1 | 1 | 1 | 1 | 1 | 1 | 1 | 1 | 1 | 1 | 1 |
| 134 | Lipopolysaccharide export system protein LptC | 1 | 1 | 1 | 1 | 1 | 1 | 1 | 1 | 1 | 1 | 1 | 1 | 1 | 1 | 1 | 1 | 1 | 1 | 1 | 1 | 1 | 1 | 2 | 1 | 1 | 1 | 1 | 1 | 1 | 1 | 1 | 1 | 1 | 1 | 1 |
| 135 | Lipopolysaccharide export system protein LptA | 1 | 1 | 1 | 1 | 1 | 1 | 1 | 1 | 1 | 1 | 1 | 1 | 1 | 1 | 1 | 1 | 1 | 1 | 1 | 1 | 1 | 1 | 2 | 1 | 1 | 1 | 1 | 1 | 1 | 1 | 1 | 1 | 1 | 1 | 1 |
| 136 | Lipopolysaccharide export system ATP-binding protein LptB | 1 | 1 | 1 | 1 | 1 | 1 | 1 | 1 | 1 | 1 | 1 | 1 | 1 | 1 | 1 | 1 | 1 | 1 | 1 | 1 | 1 | 1 | 2 | 1 | 1 | 1 | 1 | 1 | 1 | 1 | 1 | 1 | 1 | 1 | 1 |
| 141 | Glutamate--tRNA ligase | 2 | 1 | 1 | 1 | 1 | 1 | 1 | 1 | 1 | 1 | 1 | 1 | 1 | 1 | 1 | 1 | 1 | 1 | 1 | 1 | 1 | 1 | 1 | 1 | 1 | 1 | 1 | 1 | 1 | 1 | 1 | 1 | 1 | 1 | 1 |
| 143 | Tryptophan synthase beta chain | 1 | 1 | 2 | 1 | 1 | 1 | 1 | 1 | 1 | 1 | 1 | 1 | 1 | 1 | 1 | 1 | 1 | 1 | 1 | 1 | 1 | 1 | 1 | 1 | 1 | 1 | 1 | 1 | 1 | 1 | 1 | 1 | 1 | 1 | 1 |
| 144 | UDP-glucose 6-dehydrogenase TuaD | 1 | 1 | 1 | 1 | 1 | 1 | 1 | 1 | 1 | 1 | 1 | 1 | 1 | 2 | 1 | 1 | 1 | 1 | 1 | 1 | 1 | 1 | 1 | 1 | 1 | 1 | 1 | 1 | 1 | 1 | 1 | 1 | 1 | 1 | 1 |
| 145 | UDP-N-acetylglucosamine 4-epimerase | 1 | 1 | 1 | 1 | 1 | 1 | 1 | 1 | 1 | 1 | 1 | 1 | 1 | 2 | 1 | 1 | 1 | 1 | 1 | 1 | 1 | 1 | 1 | 1 | 1 | 1 | 1 | 1 | 1 | 1 | 1 | 1 | 1 | 1 | 1 |
| 147 | Pantothenate kinase | 1 | 1 | 1 | 1 | 1 | 1 | 1 | 1 | 1 | 1 | 1 | 1 | 1 | 1 | 1 | 1 | 1 | 1 | 1 | 1 | 1 | 1 | 1 | 1 | 1 | 1 | 1 | 1_p1 | 1 | 1 | 1 | 1 | 1 | 1 | 1 |
| 148 | Bifunctional ligase/repressor BirA | 1 | 1 | 1 | 1 | 1 | 1 | 1 | 1 | 1 | 1 | 1 | 1 | 1 | 1 | 1 | 1 | 1 | 1 | 1 | 1 | 1 | 1 | 1 | 1 | 1 | 1 | 1 | 1_p1 | 1 | 1 | 1 | 1 | 1 | 1 | 1 |
| 149 | UDP-N-acetylenolpyruvoylglucosamine reductase | 1 | 1 | 1 | 1 | 1 | 1 | 1 | 1 | 1 | 1 | 1 | 1 | 1 | 1 | 1 | 1 | 1 | 1 | 1 | 1 | 1 | 1 | 1 | 1 | 1 | 1 | 1 | 1_p1 | 1 | 1 | 1 | 1 | 1 | 1 | 1 |
| 152 | Valine--tRNA ligase | 1 | 1 | 1 | 1 | 1 | 1 | 1 | 1 | 1 | 1 | 1 | 1 | 1 | 1 | 1 | 1 | 1 | 1 | 1 | 1 | 1 | 1 | 1 | 1 | 1 | 1 | 1 | 1 | 1 | 1 | 1 | 1 | 1 | 1 | 1 |
| 153 | DNA polymerase III subunit chi | 1 | 1 | 1 | 1 | 1 | 1 | 1 | 1 | 1 | 1 | 1 | 1 | 1 | 1 | 1 | 1 | 1 | 1 | 1 | 1 | 1 | 1 | 1 | 1 | 1 | 1 | 1 | 1 | 1 | 1 | 1 | 1 | 1 | 1 | 1 |
| 154 | Cytosol aminopeptidase | 1 | 1 | 1 | 1 | 1 | 1 | 1 | 1 | 1 | 1 | 1 | 1 | 1 | 1 | 1 | 1 | 1 | 1 | 1 | 1 | 1 | 1 | 1 | 1 | 1 | 1 | 1 | 1 | 1 | 1 | 1 | 1 | 1 | 1 | 1 |
| 155 | Lipopolysaccharide export system permease protein LptF | 1 | 1 | 1 | 1 | 1 | 1 | 1 | 1 | 1 | 1 | 1 | 1 | 1 | 1 | 1 | 1 | 1 | 1 | 1 | 1 | 1 | 1 | 1 | 1 | 1 | 1 | 1 | 1 | 1 | 1 | 1 | 1 | 1 | 1 | 1 |
| 156 | Lipopolysaccharide export system permease protein LptG | 1 | 1 | 1 | 1 | 1 | 1 | 1 | 1 | 1 | 1 | 1 | 1 | 1 | 1 | 1 | 1 | 1 | 1 | 1 | 1 | 1 | 1 | 1 | 1 | 1 | 1 | 1 | 1 | 1 | 1 | 1 | 1 | 1 | 1 | 1 |
| 157 | Transcription-repair-coupling factor | 1 | 1 | 1 | 1 | 1 | 1 | 1 | 1 | 1 | 1 | 1 | 1 | 1 | 1 | 1 | 1 | 1 | 1 | 1 | 1 | 1 | 1 | 1 | 1 | 1 | 1 | 1 | 1 | 1 | 1 | 1 | 1 | 1 | 1 | 1 |
| 158 | Lipoprotein-releasing system transmembrane protein LolC | 1 | 1 | 1 | 1 | 1 | 1 | 1 | 1 | 1 | 1 | 1 | 1 | 1 | 1 | 1 | 1 | 1 | 1 | 1 | 1 | 1 | 1 | 1 | 1 | 1 | 1 | 1 | 1 | 1 | 1 | 1 | 1 | 1 | 1 | 1 |
| 159 | Lipoprotein-releasing system ATP-binding protein LolD | 1 | 1 | 1 | 1 | 1 | 1 | 1 | 1 | 1 | 1 | 1 | 1 | 1 | 1 | 1 | 1 | 1 | 1 | 1 | 1 | 1 | 1 | 1 | 1 | 1 | 1 | 1 | 1 | 1 | 1 | 1 | 1 | 1 | 1 | 1 |
| 160 | Lipoprotein-releasing system transmembrane protein LolE | 1 | 1 | 1 | 1 | 1 | 1 | 1 | 1 | 1 | 1 | 1 | 1 | 1 | 1 | 1 | 1 | 1 | 1 | 1 | 1 | 1 | 1 | 1 | 1 | 1 | 1 | 1 | 1 | 1 | 1 | 1 | 1 | 1 | 1 | 1 |
| 161 | 10 kDa chaperonin | 1 | 1 | 1 | 1 | 1 | 1 | 1 | 1 | 1 | 1 | 1 | 1 | 1 | 1 | 1 | 1 | 1 | 1 | 1 | 1 | 1 | 1 | 1 | 1 | 1 | 1 | 1 | 1 | 1 | 1 | 1 | 1 | 1 | 1 | 1 |
| 162 | 60 kDa chaperonin | 1 | 1 | 1 | 1 | 1 | 1 | 1 | 1 | 1 | 1 | 1 | 1 | 1 | 1 | 1 | 1 | 1 | 1 | 1 | 1 | 1 | 1 | 1 | 1 | 1 | 1 | 1 | 1 | 1 | 1 | 1 | 1 | 1 | 1 | 1 |
| 163 | Elongation factor P | 1 | 1 | 1 | 1 | 1 | 1 | 1 | 1 | 1 | 1 | 1 | 1 | 1 | 1 | 1 | 1 | 1 | 1 | 1 | 1 | 1 | 1 | 1 | 1 | 1 | 1 | 1 | 1 | 1 | 1 | 1 | 1 | 1 | 1 | 1 |
| 164 | Miniconductance mechanosensitive channel MscM | 1 | 1 | 1 | 1 | 1 | 1 | 1 | 1 | 1 | 1 | 1 | 1 | 1 | 1 | 1 | 1 | 1 | 1 | 1 | 1 | 1 | 1 | 1 | 1 | 1 | 1 | 1 | 1 | 1 | 1 | 1 | 1 | 1 | 1 | 1 |
| 165 | Phosphatidylserine decarboxylase proenzyme | 1 | 1 | 1 | 1 | 1 | 1 | 1 | 1 | 1 | 1 | 1 | 1 | 1 | 1 | 1 | 1 | 1 | 1 | 1 | 1 | 1 | 1 | 1 | 1 | 1 | 1 | 1 | 1 | 1 | 1 | 1 | 1 | 1 | 1 | 1 |
| 166 | Oligoribonuclease | 1 | 1 | 1 | 1 | 1 | 1 | 1 | 1 | 1 | 1 | 1 | 1 | 1 | 1 | 1 | 1 | 1 | 1 | 1 | 1 | 1 | 1 | 1 | 1 | 1 | 1 | 1 | 1 | 1 | 1 | 1 | 1 | 1 | 1 | 1 |
| 167 | N-acetylmuramoyl-L-alanine amidase AmiB | 1 | 1 | 1 | 1 | 1 | 1 | 1 | 1 | 1 | 1 | 1 | 1 | 1 | 1 | 1 | 1 | 1 | 1 | 1 | 1 | 1 | 1 | 1 | 1 | 1 | 1 | 1 | 1 | 1 | 1 | 1 | 1 | 1 | 1 | 1 |
| 168 | Threonylcarbamoyl-AMP synthase | 1 | 1 | 1 | 1 | 1 | 1 | 1 | 1 | 1 | 1 | 1 | 1 | 1 | 1 | 1 | 1 | 1 | 1 | 1 | 1 | 1 | 1 | 1 | 1 | 1 | 1 | 1 | 1 | 1 | 1 | 1 | 1 | 1 | 1 | 1 |
| 170 | Peptide deformylase | 1 | 1 | 1 | 1 | 1 | 1 | 1 | 1 | 1 | 1 | 1 | 1 | 1 | 1 | 1 | 1 | 1 | 1 | 1 | 1 | 1 | 1 | 1 | 1 | 1 | 1 | 1 | 1 | 1 | 1 | 1 | 1 | 1 | 1 | 1 |
| 171 | Methionyl-tRNA formyltransferase | 1 | 1 | 1 | 1 | 1 | 1 | 1 | 1 | 1 | 1 | 1 | 1 | 1 | 1 | 1 | 1 | 1 | 1 | 1 | 1 | 1 | 1 | 1 | 1 | 1 | 1 | 1 | 1 | 1 | 1 | 1 | 1 | 1 | 1 | 1 |
| 172 | Trk system potassium uptake protein TrkA | 1 | 1 | 1 | 1 | 1 | 1 | 1 | 1 | 1 | 1 | 1 | 1 | 1 | 1 | 1 | 1 | 1 | 1 | 1 | 1 | 1 | 1 | 1 | 1 | 1 | 1 | 1 | 1 | 1 | 1 | 1 | 1 | 1 | 1 | 1 |
| 173 | 50S ribosomal protein L17 | 1 | 1 | 1 | 1 | 1 | 1 | 1 | 1 | 1 | 1 | 1 | 1 | 1 | 1 | 1 | 1 | 1 | 1 | 1 | 1 | 1 | 1 | 1 | 1 | 1 | 1 | 1 | 1 | 1 | 1 | 1 | 1 | 1 | 1 | 1 |
| 174 | DNA-directed RNA polymerase subunit alpha | 1 | 1 | 1 | 1 | 1 | 1 | 1 | 1 | 1 | 1 | 1 | 1 | 1 | 1 | 1 | 1 | 1 | 1 | 1 | 1 | 1 | 1 | 1 | 1 | 1 | 1 | 1 | 1 | 1 | 1 | 1 | 1 | 1 | 1 | 1 |
| 175 | 30S ribosomal protein S4 | 1 | 1 | 1 | 1 | 1 | 1 | 1 | 1 | 1 | 1 | 1 | 1 | 1 | 1 | 1 | 1 | 1 | 1 | 1 | 1 | 1 | 1 | 1 | 1 | 1 | 1 | 1 | 1 | 1 | 1 | 1 | 1 | 1 | 1 | 1 |
| 176 | 30S ribosomal protein S13 | 1 | 1 | 1 | 1 | 1 | 1 | 1 | 1 | 1 | 1 | 1 | 1 | 1 | 1 | 1 | 1 | 1 | 1 | 1 | 1 | 1 | 1 | 1 | 1 | 1 | 1 | 1 | 1 | 1 | 1 | 1 | 1 | 1 | 1 | 1 |
| 177 | Protein translocase subunit SecY | 1 | 1 | 1 | 1 | 1 | 1 | 1 | 1 | 1 | 1 | 1 | 1 | 1 | 1 | 1 | 1 | 1 | 1 | 1 | 1 | 1 | 1 | 1 | 1 | 1 | 1 | 1 | 1 | 1 | 1 | 1 | 1 | 1 | 1 | 1 |
| 178 | 50S ribosomal protein L15 | 1 | 1 | 1 | 1 | 1 | 1 | 1 | 1 | 1 | 1 | 1 | 1 | 1 | 1 | 1 | 1 | 1 | 1 | 1 | 1 | 1 | 1 | 1 | 1 | 1 | 1 | 1 | 1 | 1 | 1 | 1 | 1 | 1 | 1 | 1 |
| 179 | 30S ribosomal protein S5 | 1 | 1 | 1 | 1 | 1 | 1 | 1 | 1 | 1 | 1 | 1 | 1 | 1 | 1 | 1 | 1 | 1 | 1 | 1 | 1 | 1 | 1 | 1 | 1 | 1 | 1 | 1 | 1 | 1 | 1 | 1 | 1 | 1 | 1 | 1 |
| 180 | 50S ribosomal protein L6 | 1 | 1 | 1 | 1 | 1 | 1 | 1 | 1 | 1 | 1 | 1 | 1 | 1 | 1 | 1 | 1 | 1 | 1 | 1 | 1 | 1 | 1 | 1 | 1 | 1 | 1 | 1 | 1 | 1 | 1 | 1 | 1 | 1 | 1 | 1 |
| 181 | 30S ribosomal protein S8 | 1 | 1 | 1 | 1 | 1 | 1 | 1 | 1 | 1 | 1 | 1 | 1 | 1 | 1 | 1 | 1 | 1 | 1 | 1 | 1 | 1 | 1 | 1 | 1 | 1 | 1 | 1 | 1 | 1 | 1 | 1 | 1 | 1 | 1 | 1 |
| 182 | 50S ribosomal protein L5 | 1 | 1 | 1 | 1 | 1 | 1 | 1 | 1 | 1 | 1 | 1 | 1 | 1 | 1 | 1 | 1 | 1 | 1 | 1 | 1 | 1 | 1 | 1 | 1 | 1 | 1 | 1 | 1 | 1 | 1 | 1 | 1 | 1 | 1 | 1 |
| 183 | 50S ribosomal protein L24 | 1 | 1 | 1 | 1 | 1 | 1 | 1 | 1 | 1 | 1 | 1 | 1 | 1 | 1 | 1 | 1 | 1 | 1 | 1 | 1 | 1 | 1 | 1 | 1 | 1 | 1 | 1 | 1 | 1 | 1 | 1 | 1 | 1 | 1 | 1 |
| 184 | 50S ribosomal protein L14 | 1 | 1 | 1 | 1 | 1 | 1 | 1 | 1 | 1 | 1 | 1 | 1 | 1 | 1 | 1 | 1 | 1 | 1 | 1 | 1 | 1 | 1 | 1 | 1 | 1 | 1 | 1 | 1 | 1 | 1 | 1 | 1 | 1 | 1 | 1 |
| 185 | 30S ribosomal protein S17 | 1 | 1 | 1 | 1 | 1 | 1 | 1 | 1 | 1 | 1 | 1 | 1 | 1 | 1 | 1 | 1 | 1 | 1 | 1 | 1 | 1 | 1 | 1 | 1 | 1 | 1 | 1 | 1 | 1 | 1 | 1 | 1 | 1 | 1 | 1 |
| 186 | 50S ribosomal protein L29 | 1 | 1 | 1 | 1 | 1 | 1 | 1 | 1 | 1 | 1 | 1 | 1 | 1 | 1 | 1 | 1 | 1 | 1 | 1 | 1 | 1 | 1 | 1 | 1 | 1 | 1 | 1 | 1 | 1 | 1 | 1 | 1 | 1 | 1 | 1 |
| 187 | 50S ribosomal protein L16 | 1 | 1 | 1 | 1 | 1 | 1 | 1 | 1 | 1 | 1 | 1 | 1 | 1 | 1 | 1 | 1 | 1 | 1 | 1 | 1 | 1 | 1 | 1 | 1 | 1 | 1 | 1 | 1 | 1 | 1 | 1 | 1 | 1 | 1 | 1 |
| 188 | 30S ribosomal protein S3 | 1 | 1 | 1 | 1 | 1 | 1 | 1 | 1 | 1 | 1 | 1 | 1 | 1 | 1 | 1 | 1 | 1 | 1 | 1 | 1 | 1 | 1 | 1 | 1 | 1 | 1 | 1 | 1 | 1 | 1 | 1 | 1 | 1 | 1 | 1 |
| 189 | 50S ribosomal protein L22 | 1 | 1 | 1 | 1 | 1 | 1 | 1 | 1 | 1 | 1 | 1 | 1 | 1 | 1 | 1 | 1 | 1 | 1 | 1 | 1 | 1 | 1 | 1 | 1 | 1 | 1 | 1 | 1 | 1 | 1 | 1 | 1 | 1 | 1 | 1 |
| 190 | 30S ribosomal protein S19 | 1 | 1 | 1 | 1 | 1 | 1 | 1 | 1 | 1 | 1 | 1 | 1 | 1 | 1 | 1 | 1 | 1 | 1 | 1 | 1 | 1 | 1 | 1 | 1 | 1 | 1 | 1 | 1 | 1 | 1 | 1 | 1 | 1 | 1 | 1 |
| 191 | 50S ribosomal protein L2 | 1 | 1 | 1 | 1 | 1 | 1 | 1 | 1 | 1 | 1 | 1 | 1 | 1 | 1 | 1 | 1 | 1 | 1 | 1 | 1 | 1 | 1 | 1 | 1 | 1 | 1 | 1 | 1 | 1 | 1 | 1 | 1 | 1 | 1 | 1 |
| 192 | 50S ribosomal protein L23 | 1 | 1 | 1 | 1 | 1 | 1 | 1 | 1 | 1 | 1 | 1 | 1 | 1 | 1 | 1 | 1 | 1 | 1 | 1 | 1 | 1 | 1 | 1 | 1 | 1 | 1 | 1 | 1 | 1 | 1 | 1 | 1 | 1 | 1 | 1 |
| 193 | 50S ribosomal protein L4 | 1 | 1 | 1 | 1 | 1 | 1 | 1 | 1 | 1 | 1 | 1 | 1 | 1 | 1 | 1 | 1 | 1 | 1 | 1 | 1 | 1 | 1 | 1 | 1 | 1 | 1 | 1 | 1 | 1 | 1 | 1 | 1 | 1 | 1 | 1 |
| 194 | 50S ribosomal protein L3 | 1 | 1 | 1 | 1 | 1 | 1 | 1 | 1 | 1 | 1 | 1 | 1 | 1 | 1 | 1 | 1 | 1 | 1 | 1 | 1 | 1 | 1 | 1 | 1 | 1 | 1 | 1 | 1 | 1 | 1 | 1 | 1 | 1 | 1 | 1 |
| 195 | 30S ribosomal protein S10 | 1 | 1 | 1 | 1 | 1 | 1 | 1 | 1 | 1 | 1 | 1 | 1 | 1 | 1 | 1 | 1 | 1 | 1 | 1 | 1 | 1 | 1 | 1 | 1 | 1 | 1 | 1 | 1 | 1 | 1 | 1 | 1 | 1 | 1 | 1 |
| 196 | DNA-directed RNA polymerase subunit beta | 1 | 1 | 1 | 1 | 1 | 1 | 1 | 1 | 1 | 1 | 1 | 1 | 1 | 1 | 1 | 1 | 1 | 1 | 1 | 1 | 1 | 1 | 1 | 1 | 1 | 1 | 1 | 1_p1 | 1 | 1 | 1 | 1 | 1 | 1 | 1 |
| 197 | DNA-directed RNA polymerase subunit beta | 1 | 1 | 1 | 1 | 1 | 1 | 1 | 1 | 1 | 1 | 1 | 1 | 1 | 1 | 1 | 1 | 1 | 1 | 1 | 1 | 1 | 1 | 1 | 1 | 1 | 1 | 1 | 1_p1 | 1 | 1 | 1 | 1 | 1 | 1 | 1 |
| 198 | 50S ribosomal protein L10 | 1 | 1 | 1 | 1 | 1 | 1 | 1 | 1 | 1 | 1 | 1 | 1 | 1 | 1 | 1 | 1 | 1 | 1 | 1 | 1 | 1 | 1 | 1 | 1 | 1 | 1 | 1 | 1_p1 | 1 | 1 | 1 | 1 | 1 | 1 | 1 |
| 199 | 50S ribosomal protein L1 | 1 | 1 | 1 | 1 | 1 | 1 | 1 | 1 | 1 | 1 | 1 | 1 | 1 | 1 | 1 | 1 | 1 | 1 | 1 | 1 | 1 | 1 | 1 | 1 | 1 | 1 | 1 | 1_p1 | 1 | 1 | 1 | 1 | 1 | 1 | 1 |
| 200 | 50S ribosomal protein L11 | 1 | 1 | 1 | 1 | 1 | 1 | 1 | 1 | 1 | 1 | 1 | 1 | 1 | 1 | 1 | 1 | 1 | 1 | 1 | 1 | 1 | 1 | 1 | 1 | 1 | 1 | 1 | 1_p1 | 1 | 1 | 1 | 1 | 1 | 1 | 1 |
| 201 | hypothetical protein | 1 | 1 | 1 | 1 | 1 | 1 | 1 | 1 | 1 | 1 | 1 | 1 | 1 | 1 | 1 | 1 | 1 | 1 | 1 | 1 | 1 | 1 | 1 | 1 | 1 | 1 | 1 | 1_p1 | 1 | 1 | 1 | 1 | 1 | 1 | 1 |
| 202 | Protein translocase subunit SecE | 1 | 1 | 1 | 1 | 1 | 1 | 1 | 1 | 1 | 1 | 1 | 1 | 1 | 1 | 1 | 1 | 1 | 1 | 1 | 1 | 1 | 1 | 1 | 1 | 1 | 1 | 1 | 1_p1 | 1 | 1 | 1 | 1 | 1 | 1 | 1 |
| 203 | CDP-diacylglycerol--serine O-phosphatidyltransferase | 1 | 1 | 1 | 1 | 1 | 1 | 1 | 1 | 1 | 1 | 1 | 1 | 1 | 1 | 1 | 1 | 1 | 1 | 1 | 1 | 1 | 1 | 1 | 1 | 1 | 1 | 1 | 1 | 1 | 1 | 1 | 1 | 1 | 1 | 1 |
| 204 | Glutamine--fructose-6-phosphate aminotransferase [isomerizing] | 1 | 1 | 1 | 1 | 1 | 1 | 1 | 1 | 1 | 1 | 1 | 1 | 1 | 1 | 1 | 1 | 1 | 1 | 1 | 1 | 1 | 1 | 1 | 1 | 1 | 1 | 1 | 1 | 1 | 1 | 1 | 1 | 1 | 1 | 1 |
| 205 | Bifunctional protein GlmU | 1 | 1 | 1 | 1 | 1 | 1 | 1 | 1 | 1 | 1 | 1 | 1 | 1 | 1 | 1 | 1 | 1 | 1 | 1 | 1 | 1 | 1 | 1 | 1 | 1 | 1 | 1 | 1 | 1 | 1 | 1 | 1 | 1 | 1 | 1 |
| 206 | ATP synthase epsilon chain | 1 | 1 | 1 | 1 | 1 | 1 | 1 | 1 | 1 | 1 | 1 | 1 | 1 | 1 | 1 | 1 | 1 | 1 | 1 | 1 | 1 | 1 | 1 | 1 | 1 | 1 | 1 | 1 | 1 | 1 | 1 | 1 | 1 | 1 | 1 |
| 207 | ATP synthase subunit beta | 1 | 1 | 1 | 1 | 1 | 1 | 1 | 1 | 1 | 1 | 1 | 1 | 1 | 1 | 1 | 1 | 1 | 1 | 1 | 1 | 1 | 1 | 1 | 1 | 1 | 1 | 1 | 1 | 1 | 1 | 1 | 1 | 1 | 1 | 1 |
| 208 | ATP synthase gamma chain | 1 | 1 | 1 | 1 | 1 | 1 | 1 | 1 | 1 | 1 | 1 | 1 | 1 | 1 | 1 | 1 | 1 | 1 | 1 | 1 | 1 | 1 | 1 | 1 | 1 | 1 | 1 | 1 | 1 | 1 | 1 | 1 | 1 | 1 | 1 |
| 209 | ATP synthase subunit alpha | 1 | 1 | 1 | 1 | 1 | 1 | 1 | 1 | 1 | 1 | 1 | 1 | 1 | 1 | 1 | 1 | 1 | 1 | 1 | 1 | 1 | 1 | 1 | 1 | 1 | 1 | 1 | 1 | 1 | 1 | 1 | 1 | 1 | 1 | 1 |
| 210 | ATP synthase subunit delta | 1 | 1 | 1 | 1 | 1 | 1 | 1 | 1 | 1 | 1 | 1 | 1 | 1 | 1 | 1 | 1 | 1 | 1 | 1 | 1 | 1 | 1 | 1 | 1 | 1 | 1 | 1 | 1 | 1 | 1 | 1 | 1 | 1 | 1 | 1 |
| 211 | ATP synthase subunit b | 1 | 1 | 1 | 1 | 1 | 1 | 1 | 1 | 1 | 1 | 1 | 1 | 1 | 1 | 1 | 1 | 1 | 1 | 1 | 1 | 1 | 1 | 1 | 1 | 1 | 1 | 1 | 1 | 1 | 1 | 1 | 1 | 1 | 1 | 1 |
| 212 | ATP synthase subunit c | 1 | 1 | 1 | 1 | 1 | 1 | 1 | 1 | 1 | 1 | 1 | 1 | 1 | 1 | 1 | 1 | 1 | 1 | 1 | 1 | 1 | 1 | 1 | 1 | 1 | 1 | 1 | 1 | 1 | 1 | 1 | 1 | 1 | 1 | 1 |
| 213 | ATP synthase subunit a | 1 | 1 | 1 | 1 | 1 | 1 | 1 | 1 | 1 | 1 | 1 | 1 | 1 | 1 | 1 | 1 | 1 | 1 | 1 | 1 | 1 | 1 | 1 | 1 | 1 | 1 | 1 | 1 | 1 | 1 | 1 | 1 | 1 | 1 | 1 |
| 214 | tRNA uridine 5-carboxymethylaminomethyl modification enzyme MnmG | 1 | 1 | 1 | 1 | 1 | 1 | 1 | 1 | 1 | 1 | 1 | 1 | 1 | 1 | 1 | 1 | 1 | 1 | 1 | 1 | 1 | 1 | 1 | 1 | 1 | 1 | 1 | 1 | 1 | 1 | 1 | 1 | 1 | 1 | 1 |
| 215 | 2,3-bisphosphoglycerate-dependent phosphoglycerate mutase | 1 | 1 | 1 | 1 | 1 | 1 | 1 | 1 | 1 | 1 | 1 | 1 | 1 | 1 | 1 | 1 | 1 | 1 | 1 | 1 | 1 | 1 | 1 | 1 | 1 | 1 | 1 | 1 | 1 | 1 | 1 | 1 | 1 | 1 | 1 |
| 216 | 6-phosphogluconolactonase | 1 | 1 | 1 | 1 | 1 | 1 | 1 | 1 | 1 | 1 | 1 | 1 | 1 | 1 | 1 | 1 | 1 | 1 | 1 | 1 | 1 | 1 | 1 | 1 | 1 | 1 | 1 | 1 | 1 | 1 | 1 | 1 | 1 | 1 | 1 |
| 217 | Adenosylmethionine-8-amino-7-oxononanoate aminotransferase | 1 | 1 | 1 | 1 | 1 | 1 | 1 | 1 | 1 | 1 | 1 | 1 | 1 | 1 | 1 | 1 | 1 | 1 | 1 | 1 | 1 | 1 | 1 | 1 | 1 | 1 | 1 | 1 | 1 | 1 | 1 | 1 | 1 | 1 | 1 |
| 218 | Biotin synthase | 1 | 1 | 1 | 1 | 1 | 1 | 1 | 1 | 1 | 1 | 1 | 1 | 1 | 1 | 1 | 1 | 1 | 1 | 1 | 1 | 1 | 1 | 1 | 1 | 1 | 1 | 1 | 1 | 1 | 1 | 1 | 1 | 1 | 1 | 1 |
| 219 | ATP-dependent dethiobiotin synthetase BioD 1 | 1 | 1 | 1 | 1 | 1 | 1 | 1 | 1 | 1 | 1 | 1 | 1 | 1 | 1 | 1 | 1 | 1 | 1 | 1 | 1 | 1 | 1 | 1 | 1 | 1 | 1 | 1 | 1 | 1 | 1 | 1 | 1 | 1 | 1 | 1 |
| 220 | Peptidoglycan-associated lipoprotein | 1 | 1 | 1 | 1 | 1 | 1 | 1 | 1 | 1 | 1 | 1 | 1 | 1 | 1 | 1 | 1 | 1 | 1 | 1 | 1 | 1 | 1 | 1 | 1 | 1 | 1 | 1 | 1 | 1 | 1 | 1 | 1 | 1 | 1 | 1 |
| 221 | Flavodoxin | 1 | 1 | 1 | 1 | 1 | 1 | 1 | 1 | 1 | 1 | 1 | 1 | 1 | 1 | 1 | 1 | 1 | 1 | 1 | 1 | 1 | 1 | 1 | 1 | 1 | 1 | 1 | 1 | 1 | 1 | 1 | 1 | 1 | 1 | 1 |
| 222 | Protein-export membrane protein SecG | 1 | 1 | 1 | 1 | 1 | 1 | 1 | 1 | 1 | 1 | 1 | 1 | 1 | 1 | 1 | 1 | 1 | 1 | 1 | 1 | 1 | 1 | 1 | 1 | 1 | 1 | 1 | 1 | 1 | 1 | 1 | 1 | 1 | 1 | 1 |
| 223 | Phosphoglucosamine mutase | 1 | 1 | 1 | 1 | 1 | 1 | 1 | 1 | 1 | 1 | 1 | 1 | 1 | 1 | 1 | 1 | 1 | 1 | 1 | 1 | 1 | 1 | 1 | 1 | 1 | 1 | 1 | 1 | 1 | 1 | 1 | 1 | 1 | 1 | 1 |
| 224 | ATP-dependent zinc metalloprotease FtsH | 1 | 1 | 1 | 1 | 1 | 1 | 1 | 1 | 1 | 1 | 1 | 1 | 1 | 1 | 1 | 1 | 1 | 1 | 1 | 1 | 1 | 1 | 1 | 1 | 1 | 1 | 1 | 1 | 1 | 1 | 1 | 1 | 1 | 1 | 1 |
| 225 | Ribosomal RNA large subunit methyltransferase E | 1 | 1 | 1 | 1 | 1 | 1 | 1 | 1 | 1 | 1 | 1 | 1 | 1 | 1 | 1 | 1 | 1 | 1 | 1 | 1 | 1 | 1 | 1 | 1 | 1 | 1 | 1 | 1 | 1 | 1 | 1 | 1 | 1 | 1 | 1 |
| 226 | Transcription elongation factor GreA | 1 | 1 | 1 | 1 | 1 | 1 | 1 | 1 | 1 | 1 | 1 | 1 | 1 | 1 | 1 | 1 | 1 | 1 | 1 | 1 | 1 | 1 | 1 | 1 | 1 | 1 | 1 | 1 | 1 | 1 | 1 | 1 | 1 | 1 | 1 |
| 227 | GTPase ObgE/CgtA | 1 | 1 | 1 | 1 | 1 | 1 | 1 | 1 | 1 | 1 | 1 | 1 | 1 | 1 | 1 | 1 | 1 | 1 | 1 | 1 | 1 | 1 | 1 | 1 | 1 | 1 | 1 | 1 | 1 | 1 | 1 | 1 | 1 | 1 | 1 |
| 228 | 50S ribosomal protein L27 | 1 | 1 | 1 | 1 | 1 | 1 | 1 | 1 | 1 | 1 | 1 | 1 | 1 | 1 | 1 | 1 | 1 | 1 | 1 | 1 | 1 | 1 | 1 | 1 | 1 | 1 | 1 | 1 | 1 | 1 | 1 | 1 | 1 | 1 | 1 |
| 229 | 50S ribosomal protein L21 | 1 | 1 | 1 | 1 | 1 | 1 | 1 | 1 | 1 | 1 | 1 | 1 | 1 | 1 | 1 | 1 | 1 | 1 | 1 | 1 | 1 | 1 | 1 | 1 | 1 | 1 | 1 | 1 | 1 | 1 | 1 | 1 | 1 | 1 | 1 |
| 231 | Inorganic pyrophosphatase | 1 | 1 | 1 | 1 | 1 | 1 | 1 | 1 | 1 | 1 | 1 | 1 | 1 | 1 | 1 | 1 | 1 | 1 | 1 | 1 | 1 | 1 | 1 | 1 | 1 | 1 | 1 | 1 | 1 | 1 | 1 | 1 | 1 | 1 | 1 |
| 233 | 50S ribosomal protein L9 | 1 | 1 | 1 | 1 | 1 | 1 | 1 | 1 | 1 | 1 | 1 | 1 | 1 | 1 | 1 | 1 | 1 | 1 | 1 | 1 | 1 | 1 | 1 | 1 | 1 | 1 | 1 | 1 | 1 | 1 | 1 | 1 | 1 | 1 | 1 |
| 234 | 30S ribosomal protein S18 | 1 | 1 | 1 | 1 | 1 | 1 | 1 | 1 | 1 | 1 | 1 | 1 | 1 | 1 | 1 | 1 | 1 | 1 | 1 | 1 | 1 | 1 | 1 | 1 | 1 | 1 | 1 | 1 | 1 | 1 | 1 | 1 | 1 | 1 | 1 |
| 235 | 30S ribosomal protein S6 | 1 | 1 | 1 | 1 | 1 | 1 | 1 | 1 | 1 | 1 | 1 | 1 | 1 | 1 | 1 | 1 | 1 | 1 | 1 | 1 | 1 | 1 | 1 | 1 | 1 | 1 | 1 | 1 | 1 | 1 | 1 | 1 | 1 | 1 | 1 |
| 236 | 23S rRNA (guanosine-2-O-)-methyltransferase RlmB | 1 | 1 | 1 | 1 | 1 | 1 | 1 | 1 | 1 | 1 | 1 | 1 | 1 | 1 | 1 | 1 | 1 | 1 | 1 | 1 | 1 | 1 | 1 | 1 | 1 | 1 | 1 | 1 | 1 | 1 | 1 | 1 | 1 | 1 | 1 |
| 237 | Ribonuclease R | 1 | 1 | 1 | 1 | 1 | 1 | 1 | 1 | 1 | 1 | 1 | 1 | 1 | 1 | 1 | 1 | 1 | 1 | 1 | 1 | 1 | 1 | 1 | 1 | 1 | 1 | 1 | 1 | 1 | 1 | 1 | 1 | 1 | 1 | 1 |
| 238 | Adenylosuccinate synthetase | 1 | 1 | 1 | 1 | 1 | 1 | 1 | 1 | 1 | 1 | 1 | 1 | 1 | 1 | 1 | 1 | 1 | 1 | 1 | 1 | 1 | 1 | 1 | 1 | 1 | 1 | 1 | 1 | 1 | 1 | 1 | 1 | 1 | 1 | 1 |
| 239 | Modulator of FtsH protease HflC | 1 | 1 | 1 | 1 | 1 | 1 | 1 | 1 | 1 | 1 | 1 | 1 | 1 | 1 | 1 | 1 | 1 | 1 | 1 | 1 | 1 | 1 | 1 | 1 | 1 | 1 | 1 | 1 | 1 | 1 | 1 | 1 | 1 | 1 | 1 |
| 240 | Modulator of FtsH protease HflK | 1 | 1 | 1 | 1 | 1 | 1 | 1 | 1 | 1 | 1 | 1 | 1 | 1 | 1 | 1 | 1 | 1 | 1 | 1 | 1 | 1 | 1 | 1 | 1 | 1 | 1 | 1 | 1 | 1 | 1 | 1 | 1 | 1 | 1 | 1 |
| 241 | tRNA dimethylallyltransferase | 1 | 1 | 1 | 1 | 1 | 1 | 1 | 1 | 1 | 1 | 1 | 1 | 1 | 1 | 1 | 1 | 1 | 1 | 1 | 1 | 1 | 1 | 1 | 1 | 1 | 1 | 1 | 1 | 1 | 1 | 1 | 1 | 1 | 1 | 1 |
| 242 | hypothetical protein | 1 | 1 | 1 | 1 | 1 | 1 | 1 | 1 | 1 | 1 | 1 | 1 | 1 | 1 | 1 | 1 | 1 | 1 | 1 | 1 | 1 | 1 | 1 | 1 | 1 | 1 | 1 | 1 | 1 | 1 | 1 | 1 | 1 | 1 | 1 |
| 243 | Translation initiation factor IF-2 | 1 | 1 | 1 | 1 | 1 | 1 | 1 | 1 | 1 | 1 | 1 | 1 | 1 | 1 | 1 | 1 | 1 | 1 | 1 | 1 | 1 | 1 | 1 | 1 | 1 | 1 | 1 | 1 | 1 | 1 | 1 | 1 | 1 | 1 | 1 |
| 244 | Ribosome-binding factor A | 1 | 1 | 1 | 1 | 1 | 1 | 1 | 1 | 1 | 1 | 1 | 1 | 1 | 1 | 1 | 1 | 1 | 1 | 1 | 1 | 1 | 1 | 1 | 1 | 1 | 1 | 1 | 1 | 1 | 1 | 1 | 1 | 1 | 1 | 1 |
| 245 | tRNA pseudouridine synthase B | 1 | 1 | 1 | 1 | 1 | 1 | 1 | 1 | 1 | 1 | 1 | 1 | 1 | 1 | 1 | 1 | 1 | 1 | 1 | 1 | 1 | 1 | 1 | 1 | 1 | 1 | 1 | 1 | 1 | 1 | 1 | 1 | 1 | 1 | 1 |
| 246 | 30S ribosomal protein S15 | 1 | 1 | 1 | 1 | 1 | 1 | 1 | 1 | 1 | 1 | 1 | 1 | 1 | 1 | 1 | 1 | 1 | 1 | 1 | 1 | 1 | 1 | 1 | 1 | 1 | 1 | 1 | 1 | 1 | 1 | 1 | 1 | 1 | 1 | 1 |
| 247 | Polyribonucleotide nucleotidyltransferase | 1 | 1 | 1 | 1 | 1 | 1 | 1 | 1 | 1 | 1 | 1 | 1 | 1 | 1 | 1 | 1 | 1 | 1 | 1 | 1 | 1 | 1 | 1 | 1 | 1 | 1 | 1 | 1 | 1 | 1 | 1 | 1 | 1 | 1 | 1 |
| 248 | ATP-dependent RNA helicase DeaD | 1 | 1 | 1 | 1 | 1 | 1 | 1 | 1 | 1 | 1 | 1 | 1 | 1 | 1 | 1 | 1 | 1 | 1 | 1 | 1 | 1 | 1 | 1 | 1 | 1 | 1 | 1 | 1 | 1 | 1 | 1 | 1 | 1 | 1 | 1 |
| 249 | Glycerol-3-phosphate dehydrogenase [NAD(P)+] | 1 | 1 | 1 | 1 | 1 | 1 | 1 | 1 | 1 | 1 | 1 | 1 | 1 | 1 | 1 | 1 | 1 | 1 | 1 | 1 | 1 | 1 | 1 | 1 | 1 | 1 | 1 | 1 | 1 | 1 | 1 | 1 | 1 | 1 | 1 |
| 250 | ATP-dependent 6-phosphofructokinase isozyme 1 | 1 | 1 | 1 | 1 | 1 | 1 | 1 | 1 | 1 | 1 | 1 | 1 | 1 | 1 | 1 | 1 | 1 | 1 | 1 | 1 | 1 | 1 | 1 | 1 | 1 | 1 | 1 | 1 | 1 | 1 | 1 | 1 | 1 | 1 | 1 |
| 251 | Triosephosphate isomerase | 1 | 1 | 1 | 1 | 1 | 1 | 1 | 1 | 1 | 1 | 1 | 1 | 1 | 1 | 1 | 1 | 1 | 1 | 1 | 1 | 1 | 1 | 1 | 1 | 1 | 1 | 1 | 1 | 1 | 1 | 1 | 1 | 1 | 1 | 1 |
| 252 | Cell division protein FtsN | 1 | 1 | 1 | 1 | 1 | 1 | 1 | 1 | 1 | 1 | 1 | 1 | 1 | 1 | 1 | 1 | 1 | 1 | 1 | 1 | 1 | 1 | 1 | 1 | 1 | 1 | 1 | 1 | 1 | 1 | 1 | 1 | 1 | 1 | 1 |
| 253 | 50S ribosomal protein L31 | 1 | 1 | 1 | 1 | 1 | 1 | 1 | 1 | 1 | 1 | 1 | 1 | 1 | 1 | 1 | 1 | 1 | 1 | 1 | 1 | 1 | 1 | 1 | 1 | 1 | 1 | 1 | 1 | 1 | 1 | 1 | 1 | 1 | 1 | 1 |
| 254 | Glutamate racemase | 1 | 1 | 1 | 1 | 1 | 1 | 1 | 1 | 1 | 1 | 1 | 1 | 1 | 1 | 1 | 1 | 1 | 1 | 1 | 1 | 1 | 1 | 1 | 1 | 1 | 1 | 1 | 1 | 1 | 1 | 1 | 1 | 1 | 1 | 1 |
| 255 | Thiol:disulfide interchange protein DsbA | 1 | 1 | 1 | 1 | 1 | 1 | 1 | 1 | 1 | 1 | 1 | 1 | 1 | 1 | 1 | 1 | 1 | 1 | 1 | 1 | 1 | 1 | 1 | 1 | 1 | 1 | 1 | 1 | 1 | 1 | 1 | 1 | 1 | 1 | 1 |
| 256 | DNA polymerase I | 1 | 1 | 1 | 1 | 1 | 1 | 1 | 1 | 1 | 1 | 1 | 1 | 1 | 1 | 1 | 1 | 1 | 1 | 1 | 1 | 1 | 1 | 1 | 1 | 1 | 1 | 1 | 1 | 1 | 1 | 1 | 1 | 1 | 1 | 1 |
| 257 | GTP-binding protein TypA/BipA | 1 | 1 | 1 | 1 | 1 | 1 | 1 | 1 | 1 | 1 | 1 | 1 | 1 | 1 | 1 | 1 | 1 | 1 | 1 | 1 | 1 | 1 | 1 | 1 | 1 | 1 | 1 | 1 | 1 | 1 | 1 | 1 | 1 | 1 | 1 |
| 258 | DNA-directed RNA polymerase subunit omega | 1 | 1 | 1 | 1 | 1 | 1 | 1 | 1 | 1 | 1 | 1 | 1 | 1 | 1 | 1 | 1 | 1 | 1 | 1 | 1 | 1 | 1 | 1 | 1 | 1 | 1 | 1 | 1 | 1 | 1 | 1 | 1 | 1 | 1 | 1 |
| 259 | Guanylate kinase | 1 | 1 | 1 | 1 | 1 | 1 | 1 | 1 | 1 | 1 | 1 | 1 | 1 | 1 | 1 | 1 | 1 | 1 | 1 | 1 | 1 | 1 | 1 | 1 | 1 | 1 | 1 | 1 | 1 | 1 | 1 | 1 | 1 | 1 | 1 |
| 260 | Deoxyuridine 5-triphosphate nucleotidohydrolase | 1 | 1 | 1 | 1 | 1 | 1 | 1 | 1 | 1 | 1 | 1 | 1 | 1 | 1 | 1 | 1 | 1 | 1 | 1 | 1 | 1 | 1 | 1 | 1 | 1 | 1 | 1 | 1 | 1 | 1 | 1 | 1 | 1 | 1 | 1 |
| 261 | 50S ribosomal protein L28 | 1 | 1 | 1 | 1 | 1 | 1 | 1 | 1 | 1 | 1 | 1 | 1 | 1 | 1 | 1 | 1 | 1 | 1 | 1 | 1 | 1 | 1 | 1 | 1 | 1 | 1 | 1 | 1 | 1 | 1 | 1 | 1 | 1 | 1 | 1 |
| 262 | 50S ribosomal protein L33 | 1 | 1 | 1 | 1 | 1 | 1 | 1 | 1 | 1 | 1 | 1 | 1 | 1 | 1 | 1 | 1 | 1 | 1 | 1 | 1 | 1 | 1 | 1 | 1 | 1 | 1 | 1 | 1 | 1 | 1 | 1 | 1 | 1 | 1 | 1 |
| 263 | Phosphopantetheine adenylyltransferase | 1 | 1 | 1 | 1 | 1 | 1 | 1 | 1 | 1 | 1 | 1 | 1 | 1 | 1 | 1 | 1 | 1 | 1 | 1 | 1 | 1 | 1 | 1 | 1 | 1 | 1 | 1 | 1 | 1 | 1 | 1 | 1 | 1 | 1 | 1 |
| 264 | hypothetical protein | 1 | 1 | 1 | 1 | 1 | 1 | 1 | 1 | 1 | 1 | 1 | 1 | 1 | 1 | 1 | 1 | 1 | 1 | 1 | 1 | 1 | 1 | 1 | 1 | 1 | 1 | 1 | 1 | 1 | 1 | 1 | 1 | 1 | 1 | 1 |
| 265 | 3-deoxy-D-manno-octulosonic acid transferase | 1 | 1 | 1 | 1 | 1 | 1 | 1 | 1 | 1 | 1 | 1 | 1 | 1 | 1 | 1 | 1 | 1 | 1 | 1 | 1 | 1 | 1 | 1 | 1 | 1 | 1 | 1 | 1 | 1 | 1 | 1 | 1 | 1 | 1 | 1 |
| 266 | N-acetylgalactosamine-N,N-diacetylbacillosaminyl-diphospho-undecaprenol 4-alpha-N-acetylgalactosaminyltransferase | 1 | 1 | 1 | 1 | 1 | 1 | 1 | 1 | 1 | 1 | 1 | 1 | 1 | 1 | 1 | 1 | 1 | 1 | 1 | 1 | 1 | 1 | 1 | 1 | 1 | 1 | 1 | 1 | 1 | 1 | 1 | 1 | 1 | 1 | 1 |
| 267 | Lipopolysaccharide core biosynthesis protein RfaG | 1 | 1 | 1 | 1 | 1 | 1 | 1 | 1 | 1 | 1 | 1 | 1 | 1 | 1 | 1 | 1 | 1 | 1 | 1 | 1 | 1 | 1 | 1 | 1 | 1 | 1 | 1 | 1 | 1 | 1 | 1 | 1 | 1 | 1 | 1 |
| 268 | Lipopolysaccharide heptosyltransferase 1 | 1 | 1 | 1 | 1 | 1 | 1 | 1 | 1 | 1 | 1 | 1 | 1 | 1 | 1 | 1 | 1 | 1 | 1 | 1 | 1 | 1 | 1 | 1 | 1 | 1 | 1 | 1 | 1 | 1 | 1 | 1 | 1 | 1 | 1 | 1 |
| 269 | ADP-heptose--LPS heptosyltransferase 2 | 1 | 1 | 1 | 1 | 1 | 1 | 1 | 1 | 1 | 1 | 1 | 1 | 1 | 1 | 1 | 1 | 1 | 1 | 1 | 1 | 1 | 1 | 1 | 1 | 1 | 1 | 1 | 1 | 1 | 1 | 1 | 1 | 1 | 1 | 1 |
| 270 | ADP-L-glycero-D-manno-heptose-6-epimerase | 1 | 1 | 1 | 1 | 1 | 1 | 1 | 1 | 1 | 1 | 1 | 1 | 1 | 1 | 1 | 1 | 1 | 1 | 1 | 1 | 1 | 1 | 1 | 1 | 1 | 1 | 1 | 1 | 1 | 1 | 1 | 1 | 1 | 1 | 1 |
| 271 | Oligopeptidase A | 1 | 1 | 1 | 1 | 1 | 1 | 1 | 1 | 1 | 1 | 1 | 1 | 1 | 1 | 1 | 1 | 1 | 1 | 1 | 1 | 1 | 1 | 1 | 1 | 1 | 1 | 1 | 1 | 1 | 1 | 1 | 1 | 1 | 1 | 1 |
| 272 | Ribosomal RNA small subunit methyltransferase J | 1 | 1 | 1 | 1 | 1 | 1 | 1 | 1 | 1 | 1 | 1 | 1 | 1 | 1 | 1 | 1 | 1 | 1 | 1 | 1 | 1 | 1 | 1 | 1 | 1 | 1 | 1 | 1 | 1 | 1 | 1 | 1 | 1 | 1 | 1 |
| 273 | Low-affinity inorganic phosphate transporter 1 | 1 | 1 | 1 | 1 | 1 | 1 | 1 | 1 | 1 | 1 | 1 | 1 | 1 | 1 | 1 | 1 | 1 | 1 | 1 | 1 | 1 | 1 | 1 | 1 | 1 | 1 | 1 | 1 | 1 | 1 | 1 | 1 | 1 | 1 | 1 |
| 274 | Bifunctional protein FolD protein | 1 | 1 | 1 | 1 | 1 | 1 | 1 | 1 | 1 | 1 | 1 | 1 | 1 | 1 | 1 | 1 | 1 | 1 | 1 | 1 | 1 | 1 | 1 | 1 | 1 | 1 | 1 | 1 | 1 | 1 | 1 | 1 | 1 | 1 | 1 |
| 275 | Cysteine--tRNA ligase | 1 | 1 | 1 | 1 | 1 | 1 | 1 | 1 | 1 | 1 | 1 | 1 | 1 | 1 | 1 | 1 | 1 | 1 | 1 | 1 | 1 | 1 | 1 | 1 | 1 | 1 | 1 | 1 | 1 | 1 | 1 | 1 | 1 | 1 | 1 |
| 276 | Phosphoenolpyruvate-protein phosphotransferase | 1 | 1 | 1 | 1 | 1 | 1 | 1 | 1 | 1 | 1 | 1 | 1 | 1 | 1 | 1 | 1 | 1 | 1 | 1 | 1 | 1 | 1 | 1 | 1 | 1 | 1 | 1 | 1 | 1 | 1 | 1 | 1 | 1 | 1 | 1 |
| 277 | Phosphocarrier protein HPr | 1 | 1 | 1 | 1 | 1 | 1 | 1 | 1 | 1 | 1 | 1 | 1 | 1 | 1 | 1 | 1 | 1 | 1 | 1 | 1 | 1 | 1 | 1 | 1 | 1 | 1 | 1 | 1 | 1 | 1 | 1 | 1 | 1 | 1 | 1 |
| 278 | DNA ligase | 1 | 1 | 1 | 1 | 1 | 1 | 1 | 1 | 1 | 1 | 1 | 1 | 1 | 1 | 1 | 1 | 1 | 1 | 1 | 1 | 1 | 1 | 1 | 1 | 1 | 1 | 1 | 1 | 1 | 1 | 1 | 1 | 1 | 1 | 1 |
| 280 | Glutamine--tRNA ligase | 1 | 1 | 1 | 1 | 1 | 1 | 1 | 1 | 1 | 1 | 1 | 1 | 1 | 1 | 1 | 1 | 1 | 1 | 1 | 1 | 1 | 1 | 1 | 1 | 1 | 1 | 1 | 1 | 1 | 1 | 1 | 1 | 1 | 1 | 1 |
| 282 | Endoribonuclease YbeY | 1 | 1 | 1 | 1 | 1 | 1 | 1 | 1 | 1 | 1 | 1 | 1 | 1 | 1 | 1 | 1 | 1 | 1 | 1 | 1 | 1 | 1 | 1 | 1 | 1 | 1 | 1 | 1 | 1 | 1 | 1 | 1 | 1 | 1 | 1 |
| 283 | Magnesium and cobalt efflux protein CorC | 1 | 1 | 1 | 1 | 1 | 1 | 1 | 1 | 1 | 1 | 1 | 1 | 1 | 1 | 1 | 1 | 1 | 1 | 1 | 1 | 1 | 1 | 1 | 1 | 1 | 1 | 1 | 1 | 1 | 1 | 1 | 1 | 1 | 1 | 1 |
| 284 | Apolipoprotein N-acyltransferase | 1 | 1 | 1 | 1 | 1 | 1 | 1 | 1 | 1 | 1 | 1 | 1 | 1 | 1 | 1 | 1 | 1 | 1 | 1 | 1 | 1 | 1 | 1 | 1 | 1 | 1 | 1 | 1 | 1 | 1 | 1 | 1 | 1 | 1 | 1 |
| 285 | Leucine--tRNA ligase | 1 | 1 | 1 | 1 | 1 | 1 | 1 | 1 | 1 | 1 | 1 | 1 | 1 | 1 | 1 | 1 | 1 | 1 | 1 | 1 | 1 | 1 | 1 | 1 | 1 | 1 | 1 | 1 | 1 | 1 | 1 | 1 | 1 | 1 | 1 |
| 286 | LPS-assembly lipoprotein LptE | 1 | 1 | 1 | 1 | 1 | 1 | 1 | 1 | 1 | 1 | 1 | 1 | 1 | 1 | 1 | 1 | 1 | 1 | 1 | 1 | 1 | 1 | 1 | 1 | 1 | 1 | 1 | 1 | 1 | 1 | 1 | 1 | 1 | 1 | 1 |
| 287 | DNA polymerase III subunit delta | 1 | 1 | 1 | 1 | 1 | 1 | 1 | 1 | 1 | 1 | 1 | 1 | 1 | 1 | 1 | 1 | 1 | 1 | 1 | 1 | 1 | 1 | 1 | 1 | 1 | 1 | 1 | 1 | 1 | 1 | 1 | 1 | 1 | 1 | 1 |
| 288 | Octanoyltransferase | 1 | 1 | 1 | 1 | 1 | 1 | 1 | 1 | 1 | 1 | 1 | 1 | 1 | 1 | 1 | 1 | 1 | 1 | 1 | 1 | 1 | 1 | 1 | 1 | 1 | 1 | 1 | 1 | 1 | 1 | 1 | 1 | 1 | 1 | 1 |
| 289 | Lipoyl synthase | 1 | 1 | 1 | 1 | 1 | 1 | 1 | 1 | 1 | 1 | 1 | 1 | 1 | 1 | 1 | 1 | 1 | 1 | 1 | 1 | 1 | 1 | 1 | 1 | 1 | 1 | 1 | 1 | 1 | 1 | 1 | 1 | 1 | 1 | 1 |
| 292 | 2-dehydro-3-deoxyphosphooctonate aldolase | 1 | 1 | 1 | 1 | 1 | 1 | 1 | 1 | 1 | 1 | 1 | 1 | 1 | 1 | 1 | 1 | 1 | 1 | 1 | 1 | 1 | 1 | 1 | 1 | 1 | 1 | 1 | 1 | 1 | 1 | 1 | 1 | 1 | 1 | 1 |
| 293 | Release factor glutamine methyltransferase | 1 | 1 | 1 | 1 | 1 | 1 | 1 | 1 | 1 | 1 | 1 | 1 | 1 | 1 | 1 | 1 | 1 | 1 | 1 | 1 | 1 | 1 | 1 | 1 | 1 | 1 | 1 | 1 | 1 | 1 | 1 | 1 | 1 | 1 | 1 |
| 294 | Peptide chain release factor 1 | 1 | 1 | 1 | 1 | 1 | 1 | 1 | 1 | 1 | 1 | 1 | 1 | 1 | 1 | 1 | 1 | 1 | 1 | 1 | 1 | 1 | 1 | 1 | 1 | 1 | 1 | 1 | 1 | 1 | 1 | 1 | 1 | 1 | 1 | 1 |
| 295 | Outer-membrane lipoprotein LolB | 1 | 1 | 1 | 1 | 1 | 1 | 1 | 1 | 1 | 1 | 1 | 1 | 1 | 1 | 1 | 1 | 1 | 1 | 1 | 1 | 1 | 1 | 1 | 1 | 1 | 1 | 1 | 1 | 1 | 1 | 1 | 1 | 1 | 1 | 1 |
| 296 | 4-diphosphocytidyl-2-C-methyl-D-erythritol kinase | 1 | 1 | 1 | 1 | 1 | 1 | 1 | 1 | 1 | 1 | 1 | 1 | 1 | 1 | 1 | 1 | 1 | 1 | 1 | 1 | 1 | 1 | 1 | 1 | 1 | 1 | 1 | 1 | 1 | 1 | 1 | 1 | 1 | 1 | 1 |
| 297 | Ribose-phosphate pyrophosphokinase | 1 | 1 | 1 | 1 | 1 | 1 | 1 | 1 | 1 | 1 | 1 | 1 | 1 | 1 | 1 | 1 | 1 | 1 | 1 | 1 | 1 | 1 | 1 | 1 | 1 | 1 | 1 | 1 | 1 | 1 | 1 | 1 | 1 | 1 | 1 |
| 298 | Peptidyl-tRNA hydrolase | 1 | 1 | 1 | 1 | 1 | 1 | 1 | 1 | 1 | 1 | 1 | 1 | 1 | 1 | 1 | 1 | 1 | 1 | 1 | 1 | 1 | 1 | 1 | 1 | 1 | 1 | 1 | 1 | 1 | 1 | 1 | 1 | 1 | 1 | 1 |
| 299 | Ribosome-binding ATPase YchF | 1 | 1 | 1 | 1 | 1 | 1 | 1 | 1 | 1 | 1 | 1 | 1 | 1 | 1 | 1 | 1 | 1 | 1 | 1 | 1 | 1 | 1 | 1 | 1 | 1 | 1 | 1 | 1 | 1 | 1 | 1 | 1 | 1 | 1 | 1 |
| 300 | Enoyl-[acyl-carrier-protein] reductase [NADH] 1 | 1 | 1 | 1 | 1 | 1 | 1 | 1 | 1 | 1 | 1 | 1 | 1 | 1 | 1 | 1 | 1 | 1 | 1 | 1 | 1 | 1 | 1 | 1 | 1 | 1 | 1 | 1 | 1 | 1 | 1 | 1 | 1 | 1 | 1 | 1 |
| 301 | Fe/S biogenesis protein NfuA | 1 | 1 | 1 | 1 | 1 | 1 | 1 | 1 | 1 | 1 | 1 | 1 | 1 | 1 | 1 | 1 | 1 | 1 | 1 | 1 | 1 | 1 | 1 | 1 | 1 | 1 | 1 | 1 | 1 | 1 | 1 | 1 | 1 | 1 | 1 |
| 302 | Penicillin-binding protein 1A | 1 | 1 | 1 | 1 | 1 | 1 | 1 | 1 | 1 | 1 | 1 | 1 | 1 | 1 | 1 | 1 | 1 | 1 | 1 | 1 | 1 | 1 | 1 | 1 | 1 | 1 | 1 | 1 | 1 | 1 | 1 | 1 | 1 | 1 | 1 |
| 303 | Shikimate kinase 1 | 1 | 1 | 1 | 1 | 1 | 1 | 1 | 1 | 1 | 1 | 1 | 1 | 1 | 1 | 1 | 1 | 1 | 1 | 1 | 1 | 1 | 1 | 1 | 1 | 1 | 1 | 1 | 1 | 1 | 1 | 1 | 1 | 1 | 1 | 1 |
| 304 | Ribulose-phosphate 3-epimerase | 1 | 1 | 1 | 1 | 1 | 1 | 1 | 1 | 1 | 1 | 1 | 1 | 1 | 1 | 1 | 1 | 1 | 1 | 1 | 1 | 1 | 1 | 1 | 1 | 1 | 1 | 1 | 1 | 1 | 1 | 1 | 1 | 1 | 1 | 1 |
| 305 | Tryptophan--tRNA ligase | 1 | 1 | 1 | 1 | 1 | 1 | 1 | 1 | 1 | 1 | 1 | 1 | 1 | 1 | 1 | 1 | 1 | 1 | 1 | 1 | 1 | 1 | 1 | 1 | 1 | 1 | 1 | 1 | 1 | 1 | 1 | 1 | 1 | 1 | 1 |
| 306 | Sulfurtransferase TusD | 1 | 1 | 1 | 1 | 1 | 1 | 1 | 1 | 1 | 1 | 1 | 1 | 1 | 1 | 1 | 1 | 1 | 1 | 1 | 1 | 1 | 1 | 1 | 1 | 1 | 1 | 1 | 1 | 1 | 1 | 1 | 1 | 1 | 1 | 1 |
| 307 | Intracellular sulfur oxidation protein DsrF | 1 | 1 | 1 | 1 | 1 | 1 | 1 | 1 | 1 | 1 | 1 | 1 | 1 | 1 | 1 | 1 | 1 | 1 | 1 | 1 | 1 | 1 | 1 | 1 | 1 | 1 | 1 | 1 | 1 | 1 | 1 | 1 | 1 | 1 | 1 |
| 308 | Protein TusB | 1 | 1 | 1 | 1 | 1 | 1 | 1 | 1 | 1 | 1 | 1 | 1 | 1 | 1 | 1 | 1 | 1 | 1 | 1 | 1 | 1 | 1 | 1 | 1 | 1 | 1 | 1 | 1 | 1 | 1 | 1 | 1 | 1 | 1 | 1 |
| 309 | 30S ribosomal protein S12 | 1 | 1 | 1 | 1 | 1 | 1 | 1 | 1 | 1 | 1 | 1 | 1 | 1 | 1 | 1 | 1 | 1 | 1 | 1 | 1 | 1 | 1 | 1 | 1 | 1 | 1 | 1 | 1 | 1 | 1 | 1 | 1 | 1 | 1 | 1 |
| 310 | 30S ribosomal protein S7 | 1 | 1 | 1 | 1 | 1 | 1 | 1 | 1 | 1 | 1 | 1 | 1 | 1 | 1 | 1 | 1 | 1 | 1 | 1 | 1 | 1 | 1 | 1 | 1 | 1 | 1 | 1 | 1 | 1 | 1 | 1 | 1 | 1 | 1 | 1 |
| 311 | Diaminopimelate epimerase | 1 | 1 | 1 | 1 | 1 | 1 | 1 | 1 | 1 | 1 | 1 | 1 | 1 | 1 | 1 | 1 | 1 | 1 | 1 | 1 | 1 | 1 | 1 | 1 | 1 | 1 | 1 | 1 | 1 | 1 | 1 | 1 | 1 | 1 | 1 |
| 312 | DNA helicase II | 1 | 1 | 1 | 1 | 1 | 1 | 1 | 1 | 1 | 1 | 1 | 1 | 1 | 1 | 1 | 1 | 1 | 1 | 1 | 1 | 1 | 1 | 1 | 1 | 1 | 1 | 1 | 1 | 1 | 1 | 1 | 1 | 1 | 1 | 1 |
| 313 | Pyridoxal phosphate phosphatase YigL | 1 | 1 | 1 | 1 | 1 | 1 | 1 | 1 | 1 | 1 | 1 | 1 | 1 | 1 | 1 | 1 | 1 | 1 | 1 | 1 | 1 | 1 | 1 | 1 | 1 | 1 | 1 | 1 | 1 | 1 | 1 | 1 | 1 | 1 | 1 |
| 314 | Sulfurtransferase TusA | 1 | 1 | 1 | 1 | 1 | 1 | 1 | 1 | 1 | 1 | 1 | 1 | 1 | 1 | 1 | 1 | 1 | 1 | 1 | 1 | 1 | 1 | 1 | 1 | 1 | 1 | 1 | 1 | 1 | 1 | 1 | 1 | 1 | 1 | 1 |
| 315 | Signal recognition particle receptor FtsY | 1 | 1 | 1 | 1 | 1 | 1 | 1 | 1 | 1 | 1 | 1 | 1 | 1 | 1 | 1 | 1 | 1 | 1 | 1 | 1 | 1 | 1 | 1 | 1 | 1 | 1 | 1 | 1 | 1 | 1 | 1 | 1 | 1 | 1 | 1 |
| 316 | RNA polymerase sigma factor RpoH | 1 | 1 | 1 | 1 | 1 | 1 | 1 | 1 | 1 | 1 | 1 | 1 | 1 | 1 | 1 | 1 | 1 | 1 | 1 | 1 | 1 | 1 | 1 | 1 | 1 | 1 | 1 | 1 | 1 | 1 | 1 | 1 | 1 | 1 | 1 |
| 317 | Trk system potassium uptake protein TrkH | 1 | 1 | 1 | 1 | 1 | 1 | 1 | 1 | 1 | 1 | 1 | 1 | 1 | 1 | 1 | 1 | 1 | 1 | 1 | 1 | 1 | 1 | 1 | 1 | 1 | 1 | 1 | 1 | 1 | 1 | 1 | 1 | 1 | 1 | 1 |
| 318 | RNA polymerase-binding transcription factor DksA | 1 | 1 | 1 | 1 | 1 | 1 | 1 | 1 | 1 | 1 | 1 | 1 | 1 | 1 | 1 | 1 | 1 | 1 | 1 | 1 | 1 | 1 | 1 | 1 | 1 | 1 | 1 | 1 | 1 | 1 | 1 | 1 | 1 | 1 | 1 |
| 319 | Poly(A) polymerase I | 1 | 1 | 1 | 1 | 1 | 1 | 1 | 1 | 1 | 1 | 1 | 1 | 1 | 1 | 1 | 1 | 1 | 1 | 1 | 1 | 1 | 1 | 1 | 1 | 1 | 1 | 1 | 1 | 1 | 1 | 1 | 1 | 1 | 1 | 1 |
| 320 | Hypoxanthine phosphoribosyltransferase | 1 | 1 | 1 | 1 | 1 | 1 | 1 | 1 | 1 | 1 | 1 | 1 | 1 | 1 | 1 | 1 | 1 | 1 | 1 | 1 | 1 | 1 | 1 | 1 | 1 | 1 | 1 | 1 | 1 | 1 | 1 | 1 | 1 | 1 | 1 |
| 321 | Dihydrolipoyl dehydrogenase | 1 | 1 | 1 | 1 | 1 | 1 | 1 | 1 | 1 | 1 | 1 | 1 | 1 | 1 | 1 | 1 | 1 | 1 | 1 | 1 | 1 | 1 | 1 | 1 | 1 | 1 | 1 | 1 | 1 | 1 | 1 | 1 | 1 | 1 | 1 |
| 322 | Dihydrolipoyllysine-residue acetyltransferase component of pyruvate dehydrogenase complex | 1 | 1 | 1 | 1 | 1 | 1 | 1 | 1 | 1 | 1 | 1 | 1 | 1 | 1 | 1 | 1 | 1 | 1 | 1 | 1 | 1 | 1 | 1 | 1 | 1 | 1 | 1 | 1 | 1 | 1 | 1 | 1 | 1 | 1 | 1 |
| 323 | Glutathione synthetase | 1 | 1 | 1 | 1 | 1 | 1 | 1 | 1 | 1 | 1 | 1 | 1 | 1 | 1 | 1 | 1 | 1 | 1 | 1 | 1 | 1 | 1 | 1 | 1 | 1 | 1 | 1 | 1 | 1 | 1 | 1 | 1 | 1 | 1 | 1 |
| 324 | Putative pre-16S rRNA nuclease | 1 | 1 | 1 | 1 | 1 | 1 | 1 | 1 | 1 | 1 | 1 | 1 | 1 | 1 | 1 | 1 | 1 | 1 | 1 | 1 | 1 | 1 | 1 | 1 | 1 | 1 | 1 | 1 | 1 | 1 | 1 | 1 | 1 | 1 | 1 |
| 325 | hypothetical protein | 1 | 1 | 1 | 1 | 1 | 1 | 1 | 1 | 1 | 1 | 1 | 1 | 1 | 1 | 1 | 1 | 1 | 1 | 1 | 1 | 1 | 1 | 1 | 1 | 1 | 1 | 1 | 1 | 1 | 1 | 1 | 1 | 1 | 1 | 1 |
| 326 | tRNA (guanine-N(7)-)-methyltransferase | 1 | 1 | 1 | 1 | 1 | 1 | 1 | 1 | 1 | 1 | 1 | 1 | 1 | 1 | 1 | 1 | 1 | 1 | 1 | 1 | 1 | 1 | 1 | 1 | 1 | 1 | 1 | 1 | 1 | 1 | 1 | 1 | 1 | 1 | 1 |
| 327 | putative Fe(2+)-trafficking protein | 1 | 1 | 1 | 1 | 1 | 1 | 1 | 1 | 1 | 1 | 1 | 1 | 1 | 1 | 1 | 1 | 1 | 1 | 1 | 1 | 1 | 1 | 1 | 1 | 1 | 1 | 1 | 1 | 1 | 1 | 1 | 1 | 1 | 1 | 1 |
| 328 | 6-phosphogluconate dehydrogenase, decarboxylating | 1 | 1 | 1 | 1 | 1 | 1 | 1 | 1 | 1 | 1 | 1 | 1 | 1 | 1 | 1 | 1 | 1 | 1 | 1 | 1 | 1 | 1 | 1 | 1 | 1 | 1 | 1 | 1 | 1 | 1 | 1 | 1 | 1 | 1 | 1 |
| 329 | Magnesium and cobalt efflux protein CorC | 1 | 1 | 1 | 1 | 1 | 1 | 1 | 1 | 1 | 1 | 1 | 1 | 1 | 1 | 1 | 1 | 1 | 1 | 1 | 1 | 1 | 1 | 1 | 1 | 1 | 1 | 1 | 1 | 1 | 1 | 1 | 1 | 1 | 1 | 1 |
| 330 | Deoxycytidine triphosphate deaminase | 1 | 1 | 1 | 1 | 1 | 1 | 1 | 1 | 1 | 1 | 1 | 1 | 1 | 1 | 1 | 1 | 1 | 1 | 1 | 1 | 1 | 1 | 1 | 1 | 1 | 1 | 1 | 1 | 1 | 1 | 1 | 1 | 1 | 1 | 1 |
| 331 | Methionine--tRNA ligase | 1 | 1 | 1 | 1 | 1 | 1 | 1 | 1 | 1 | 1 | 1 | 1 | 1 | 1 | 1 | 1 | 1 | 1 | 1 | 1 | 1 | 1 | 1 | 1 | 1 | 1 | 1 | 1 | 1 | 1 | 1 | 1 | 1 | 1 | 1 |
| 332 | NADH dehydrogenase | 1 | 1 | 1 | 1 | 1 | 1 | 1 | 1 | 1 | 1 | 1 | 1 | 1 | 1 | 1 | 1 | 1 | 1 | 1 | 1 | 1 | 1 | 1 | 1 | 1 | 1 | 1 | 1 | 1 | 1 | 1 | 1 | 1 | 1 | 1 |
| 333 | Thiamine kinase | 1 | 1 | 1 | 1 | 1 | 1 | 1 | 1 | 1 | 1 | 1 | 1 | 1 | 1 | 1 | 1 | 1 | 1 | 1 | 1 | 1 | 1 | 1 | 1 | 1 | 1 | 1 | 1 | 1 | 1 | 1 | 1 | 1 | 1 | 1 |
| 334 | HIT-like protein | 1 | 1 | 1 | 1 | 1 | 1 | 1 | 1 | 1 | 1 | 1 | 1 | 1 | 1 | 1 | 1 | 1 | 1 | 1 | 1 | 1 | 1 | 1 | 1 | 1 | 1 | 1 | 1 | 1 | 1 | 1 | 1 | 1 | 1 | 1 |
| 335 | PTS system glucose-specific EIICB component | 1 | 1 | 1 | 1 | 1 | 1 | 1 | 1 | 1 | 1 | 1 | 1 | 1 | 1 | 1 | 1 | 1 | 1 | 1 | 1 | 1 | 1 | 1 | 1 | 1 | 1 | 1 | 1 | 1 | 1 | 1 | 1 | 1 | 1 | 1 |
| 336 | DNA polymerase III subunit delta | 1 | 1 | 1 | 1 | 1 | 1 | 1 | 1 | 1 | 1 | 1 | 1 | 1 | 1 | 1 | 1 | 1 | 1 | 1 | 1 | 1 | 1 | 1 | 1 | 1 | 1 | 1 | 1 | 1 | 1 | 1 | 1 | 1 | 1 | 1 |
| 337 | Thymidylate kinase | 1 | 1 | 1 | 1 | 1 | 1 | 1 | 1 | 1 | 1 | 1 | 1 | 1 | 1 | 1 | 1 | 1 | 1 | 1 | 1 | 1 | 1 | 1 | 1 | 1 | 1 | 1 | 1 | 1 | 1 | 1 | 1 | 1 | 1 | 1 |
| 338 | hypothetical protein | 1 | 1 | 1 | 1 | 1 | 1 | 1 | 1 | 1 | 1 | 1 | 1 | 1 | 1 | 1 | 1 | 1 | 1 | 1 | 1 | 1 | 1 | 1 | 1 | 1 | 1 | 1 | 1 | 1 | 1 | 1 | 1 | 1 | 1 | 1 |
| 339 | 3-oxoacyl-[acyl-carrier-protein] synthase 2 | 1 | 1 | 1 | 1 | 1 | 1 | 1 | 1 | 1 | 1 | 1 | 1 | 1 | 1 | 1 | 1 | 1 | 1 | 1 | 1 | 1 | 1 | 1 | 1 | 1 | 1 | 1 | 1 | 1 | 1 | 1 | 1 | 1 | 1 | 1 |
| 340 | Acyl carrier protein | 1 | 1 | 1 | 1 | 1 | 1 | 1 | 1 | 1 | 1 | 1 | 1 | 1 | 1 | 1 | 1 | 1 | 1 | 1 | 1 | 1 | 1 | 1 | 1 | 1 | 1 | 1 | 1 | 1 | 1 | 1 | 1 | 1 | 1 | 1 |
| 341 | 3-oxoacyl-[acyl-carrier-protein] reductase FabG | 1 | 1 | 1 | 1 | 1 | 1 | 1 | 1 | 1 | 1 | 1 | 1 | 1 | 1 | 1 | 1 | 1 | 1 | 1 | 1 | 1 | 1 | 1 | 1 | 1 | 1 | 1 | 1 | 1 | 1 | 1 | 1 | 1 | 1 | 1 |
| 342 | Malonyl CoA-acyl carrier protein transacylase | 1 | 1 | 1 | 1 | 1 | 1 | 1 | 1 | 1 | 1 | 1 | 1 | 1 | 1 | 1 | 1 | 1 | 1 | 1 | 1 | 1 | 1 | 1 | 1 | 1 | 1 | 1 | 1 | 1 | 1 | 1 | 1 | 1 | 1 | 1 |
| 343 | 3-oxoacyl-[acyl-carrier-protein] synthase 3 | 1 | 1 | 1 | 1 | 1 | 1 | 1 | 1 | 1 | 1 | 1 | 1 | 1 | 1 | 1 | 1 | 1 | 1 | 1 | 1 | 1 | 1 | 1 | 1 | 1 | 1 | 1 | 1 | 1 | 1 | 1 | 1 | 1 | 1 | 1 |
| 344 | 50S ribosomal protein L32 | 1 | 1 | 1 | 1 | 1 | 1 | 1 | 1 | 1 | 1 | 1 | 1 | 1 | 1 | 1 | 1 | 1 | 1 | 1 | 1 | 1 | 1 | 1 | 1 | 1 | 1 | 1 | 1 | 1 | 1 | 1 | 1 | 1 | 1 | 1 |
| 345 | Ribosomal large subunit pseudouridine synthase C | 1 | 1 | 1 | 1 | 1 | 1 | 1 | 1 | 1 | 1 | 1 | 1 | 1 | 1 | 1 | 1 | 1 | 1 | 1 | 1 | 1 | 1 | 1 | 1 | 1 | 1 | 1 | 1 | 1 | 1 | 1 | 1 | 1 | 1 | 1 |
| 346 | Ribonuclease E | 1 | 1 | 1 | 1 | 1 | 1 | 1 | 1 | 1 | 1 | 1 | 1 | 1 | 1 | 1 | 1 | 1 | 1 | 1 | 1 | 1 | 1 | 1 | 1 | 1 | 1 | 1 | 1 | 1 | 1 | 1 | 1 | 1 | 1 | 1 |
| 347 | Thiosulfate sulfurtransferase GlpE | 1 | 1 | 1 | 1 | 1 | 1 | 1 | 1 | 1 | 1 | 1 | 1 | 1 | 1 | 1 | 1 | 1 | 1 | 1 | 1 | 1 | 1 | 1 | 1 | 1 | 1 | 1 | 1 | 1 | 1 | 1 | 1 | 1 | 1 | 1 |
| 348 | CDP-diacylglycerol--glycerol-3-phosphate 3-phosphatidyltransferase | 1 | 1 | 1 | 1 | 1 | 1 | 1 | 1 | 1 | 1 | 1 | 1 | 1 | 1 | 1 | 1 | 1 | 1 | 1 | 1 | 1 | 1 | 1 | 1 | 1 | 1 | 1 | 1 | 1 | 1 | 1 | 1 | 1 | 1 | 1 |
| 349 | D-serine/D-alanine/glycine transporter | 1 | 1 | 1 | 1 | 1 | 1 | 1 | 1 | 1 | 1 | 1 | 1 | 1 | 1 | 1 | 1 | 1 | 1 | 1 | 1 | 1 | 1 | 1 | 1 | 1 | 1 | 1 | 1 | 1 | 1 | 1 | 1 | 1 | 1 | 1 |
| 350 | Translation initiation factor IF-1 | 1 | 1 | 1 | 1 | 1 | 1 | 1 | 1 | 1 | 1 | 1 | 1 | 1 | 1 | 1 | 1 | 1 | 1 | 1 | 1 | 1 | 1 | 1 | 1 | 1 | 1 | 1 | 1 | 1 | 1 | 1 | 1 | 1 | 1 | 1 |
| 351 | Thioredoxin reductase | 1 | 1 | 1 | 1 | 1 | 1 | 1 | 1 | 1 | 1 | 1 | 1 | 1 | 1 | 1 | 1 | 1 | 1 | 1 | 1 | 1 | 1 | 1 | 1 | 1 | 1 | 1 | 1 | 1 | 1 | 1 | 1 | 1 | 1 | 1 |
| 352 | DNA translocase FtsK | 1 | 1 | 1 | 1 | 1 | 1 | 1 | 1 | 1 | 1 | 1 | 1 | 1 | 1 | 1 | 1 | 1 | 1 | 1 | 1 | 1 | 1 | 1 | 1 | 1 | 1 | 1 | 1 | 1 | 1 | 1 | 1 | 1 | 1 | 1 |
| 353 | Outer-membrane lipoprotein carrier protein | 1 | 1 | 1 | 1 | 1 | 1 | 1 | 1 | 1 | 1 | 1 | 1 | 1 | 1 | 1 | 1 | 1 | 1 | 1 | 1 | 1 | 1 | 1 | 1 | 1 | 1 | 1 | 1 | 1 | 1 | 1 | 1 | 1 | 1 | 1 |
| 354 | Serine--tRNA ligase | 1 | 1 | 1 | 1 | 1 | 1 | 1 | 1 | 1 | 1 | 1 | 1 | 1 | 1 | 1 | 1 | 1 | 1 | 1 | 1 | 1 | 1 | 1 | 1 | 1 | 1 | 1 | 1 | 1 | 1 | 1 | 1 | 1 | 1 | 1 |
| 355 | Phosphoserine aminotransferase | 1 | 1 | 1 | 1 | 1 | 1 | 1 | 1 | 1 | 1 | 1 | 1 | 1 | 1 | 1 | 1 | 1 | 1 | 1 | 1 | 1 | 1 | 1 | 1 | 1 | 1 | 1 | 1 | 1 | 1 | 1 | 1 | 1 | 1 | 1 |
| 356 | Cytidylate kinase | 1 | 1 | 1 | 1 | 1 | 1 | 1 | 1 | 1 | 1 | 1 | 1 | 1 | 1 | 1 | 1 | 1 | 1 | 1 | 1 | 1 | 1 | 1 | 1 | 1 | 1 | 1 | 1 | 1 | 1 | 1 | 1 | 1 | 1 | 1 |
| 357 | 30S ribosomal protein S1 | 1 | 1 | 1 | 1 | 1 | 1 | 1 | 1 | 1 | 1 | 1 | 1 | 1 | 1 | 1 | 1 | 1 | 1 | 1 | 1 | 1 | 1 | 1 | 1 | 1 | 1 | 1 | 1 | 1 | 1 | 1 | 1 | 1 | 1 | 1 |
| 358 | Integration host factor subunit beta | 1 | 1 | 1 | 1 | 1 | 1 | 1 | 1 | 1 | 1 | 1 | 1 | 1 | 1 | 1 | 1 | 1 | 1 | 1 | 1 | 1 | 1 | 1 | 1 | 1 | 1 | 1 | 1 | 1 | 1 | 1 | 1 | 1 | 1 | 1 |
| 359 | Lipid A export ATP-binding/permease protein MsbA | 1 | 1 | 1 | 1 | 1 | 1 | 1 | 1 | 1 | 1 | 1 | 1 | 1 | 1 | 1 | 1 | 1 | 1 | 1 | 1 | 1 | 1 | 1 | 1 | 1 | 1 | 1 | 1 | 1 | 1 | 1 | 1 | 1 | 1 | 1 |
| 360 | Tetraacyldisaccharide 4-kinase | 1 | 1 | 1 | 1 | 1 | 1 | 1 | 1 | 1 | 1 | 1 | 1 | 1 | 1 | 1 | 1 | 1 | 1 | 1 | 1 | 1 | 1 | 1 | 1 | 1 | 1 | 1 | 1 | 1 | 1 | 1 | 1 | 1 | 1 | 1 |
| 361 | 3-deoxy-manno-octulosonate cytidylyltransferase | 1 | 1 | 1 | 1 | 1 | 1 | 1 | 1 | 1 | 1 | 1 | 1 | 1 | 1 | 1 | 1 | 1 | 1 | 1 | 1 | 1 | 1 | 1 | 1 | 1 | 1 | 1 | 1 | 1 | 1 | 1 | 1 | 1 | 1 | 1 |
| 362 | Asparagine--tRNA ligase | 1 | 1 | 1 | 1 | 1 | 1 | 1 | 1 | 1 | 1 | 1 | 1 | 1 | 1 | 1 | 1 | 1 | 1 | 1 | 1 | 1 | 1 | 1 | 1 | 1 | 1 | 1 | 1 | 1 | 1 | 1 | 1 | 1 | 1 | 1 |
| 364 | 3-hydroxydecanoyl-[acyl-carrier-protein] dehydratase | 1 | 1 | 1 | 1 | 1 | 1 | 1 | 1 | 1 | 1 | 1 | 1 | 1 | 1 | 1 | 1 | 1 | 1 | 1 | 1 | 1 | 1 | 1 | 1 | 1 | 1 | 1 | 1 | 1 | 1 | 1 | 1 | 1 | 1 | 1 |
| 365 | Lon protease 1 | 1 | 1 | 1 | 1 | 1 | 1 | 1 | 1 | 1 | 1 | 1 | 1 | 1 | 1 | 1 | 1 | 1 | 1 | 1 | 1 | 1 | 1 | 1 | 1 | 1 | 1 | 1 | 1 | 1 | 1 | 1 | 1 | 1 | 1 | 1 |
| 366 | Sulfurtransferase TusE | 1 | 1 | 1 | 1 | 1 | 1 | 1 | 1 | 1 | 1 | 1 | 1 | 1 | 1 | 1 | 1 | 1 | 1 | 1 | 1 | 1 | 1 | 1 | 1 | 1 | 1 | 1 | 1 | 1 | 1 | 1 | 1 | 1 | 1 | 1 |
| 367 | hypothetical protein | 1 | 1 | 1 | 1 | 1 | 1 | 1 | 1 | 1 | 1 | 1 | 1 | 1 | 1 | 1 | 1 | 1 | 1 | 1 | 1 | 1 | 1 | 1 | 1 | 1 | 1 | 1 | 1 | 1 | 1 | 1 | 1 | 1 | 1 | 1 |
| 368 | Thioredoxin-1 | 1 | 1 | 1 | 1 | 1 | 1 | 1 | 1 | 1 | 1 | 1 | 1 | 1 | 1 | 1 | 1 | 1 | 1 | 1 | 1 | 1 | 1 | 1 | 1 | 1 | 1 | 1 | 1 | 1 | 1 | 1 | 1 | 1 | 1 | 1 |
| 369 | Superoxide dismutase [Mn] | 1 | 1 | 1 | 1 | 1 | 1 | 1 | 1 | 1 | 1 | 1 | 1 | 1 | 1 | 1 | 1 | 1 | 1 | 1 | 1 | 1 | 1 | 1 | 1 | 1 | 1 | 1 | 1 | 1 | 1 | 1 | 1 | 1 | 1 | 1 |
| 370 | Glycine--tRNA ligase beta subunit | 1 | 1 | 1 | 1 | 1 | 1 | 1 | 1 | 1 | 1 | 1 | 1 | 1 | 1 | 1 | 1 | 1 | 1 | 1 | 1 | 1 | 1 | 1 | 1 | 1 | 1 | 1 | 1 | 1 | 1 | 1 | 1 | 1 | 1 | 1 |
| 371 | Glycine--tRNA ligase alpha subunit | 1 | 1 | 1 | 1 | 1 | 1 | 1 | 1 | 1 | 1 | 1 | 1 | 1 | 1 | 1 | 1 | 1 | 1 | 1 | 1 | 1 | 1 | 1 | 1 | 1 | 1 | 1 | 1 | 1 | 1 | 1 | 1 | 1 | 1 | 1 |
| 373 | DNA gyrase subunit B | 1 | 1 | 1 | 1 | 1 | 1 | 1 | 1 | 1 | 1 | 1 | 1 | 1 | 1 | 1 | 1 | 1 | 1 | 1 | 1 | 1 | 1 | 1 | 1 | 1 | 1 | 1 | 1 | 1 | 1 | 1 | 1 | 1 | 1 | 1 |
| 374 | DNA polymerase III subunit beta | 1 | 1 | 1 | 1 | 1 | 1 | 1 | 1 | 1 | 1 | 1 | 1 | 1 | 1 | 1 | 1 | 1 | 1 | 1 | 1 | 1 | 1 | 1 | 1 | 1 | 1 | 1 | 1 | 1 | 1 | 1 | 1 | 1 | 1 | 1 |
| 375 | Chromosomal replication initiator protein DnaA | 1 | 1 | 1 | 1 | 1 | 1 | 1 | 1 | 1 | 1 | 1 | 1 | 1 | 1 | 1 | 1 | 1 | 1 | 1 | 1 | 1 | 1 | 1 | 1 | 1 | 1 | 1 | 1 | 1 | 1 | 1 | 1 | 1 | 1 | 1 |
| 376 | Ribonuclease P protein component | 1 | 1 | 1 | 1 | 1 | 1 | 1 | 1 | 1 | 1 | 1 | 1 | 1 | 1 | 1 | 1 | 1 | 1 | 1 | 1 | 1 | 1 | 1 | 1 | 1 | 1 | 1 | 1 | 1 | 1 | 1 | 1 | 1 | 1 | 1 |
| 377 | Membrane protein insertase YidC | 1 | 1 | 1 | 1 | 1 | 1 | 1 | 1 | 1 | 1 | 1 | 1 | 1 | 1 | 1 | 1 | 1 | 1 | 1 | 1 | 1 | 1 | 1 | 1 | 1 | 1 | 1 | 1 | 1 | 1 | 1 | 1 | 1 | 1 | 1 |
| 378 | tRNA modification GTPase MnmE | 1 | 1 | 1 | 1 | 1 | 1 | 1 | 1 | 1 | 1 | 1 | 1 | 1 | 1 | 1 | 1 | 1 | 1 | 1 | 1 | 1 | 1 | 1 | 1 | 1 | 1 | 1 | 1 | 1 | 1 | 1 | 1 | 1 | 1 | 1 |
| 381 | Cold shock-like protein CspC | 1 | 1 | 1 | 1 | 1 | 1 | 1 | 1 | 1 | 1 | 1 | 1 | 1 | 1 | 1 | 1 | 1 | 1 | 1 | 1 | 1 | 1 | 1 | 1 | 1 | 1 | 1 | 1 | 1 | 1 | 1 | 1 | 1 | 1 | 1 |
| 383 | 1-acyl-sn-glycerol-3-phosphate acyltransferase | 1 | 1 | 1 | 1 | 1 | 1 | 1 | 1 | 1 | 1 | 1 | 1 | 1 | 1 | 1 | 1 | 1 | 1 | 1 | 1 | 1 | 1 | 1 | 1 | 1 | 1 | 1 | 1 | 1 | 1 | 1 | 1 | 1 | 1 | 1 |
| 384 | Outer membrane protein TolC | 1 | 1 | 1 | 1 | 1 | 1 | 1 | 1 | 1 | 1 | 1 | 1 | 1 | 1 | 1 | 1 | 1 | 1 | 1 | 1 | 1 | 1 | 1 | 1 | 1 | 1 | 1 | 1 | 1 | 1 | 1 | 1 | 1 | 1 | 1 |
| 385 | 3,4-dihydroxy-2-butanone 4-phosphate synthase | 1 | 1 | 1 | 1 | 1 | 1 | 1 | 1 | 1 | 1 | 1 | 1 | 1 | 1 | 1 | 1 | 1 | 1 | 1 | 1 | 1 | 1 | 1 | 1 | 1 | 1 | 1 | 1 | 1 | 1 | 1 | 1 | 1 | 1 | 1 |
| 386 | Bifunctional protein HldE | 1 | 1 | 1 | 1 | 1 | 1 | 1 | 1 | 1 | 1 | 1 | 1 | 1 | 1 | 1 | 1 | 1 | 1 | 1 | 1 | 1 | 1 | 1 | 1 | 1 | 1 | 1 | 1 | 1 | 1 | 1 | 1 | 1 | 1 | 1 |
| 387 | Multifunctional CCA protein | 1 | 1 | 1 | 1 | 1 | 1 | 1 | 1 | 1 | 1 | 1 | 1 | 1 | 1 | 1 | 1 | 1 | 1 | 1 | 1 | 1 | 1 | 1 | 1 | 1 | 1 | 1 | 1 | 1 | 1 | 1 | 1 | 1 | 1 | 1 |
| 388 | tRNA N6-adenosine threonylcarbamoyltransferase | 1 | 1 | 1 | 1 | 1 | 1 | 1 | 1 | 1 | 1 | 1 | 1 | 1 | 1 | 1 | 1 | 1 | 1 | 1 | 1 | 1 | 1 | 1 | 1 | 1 | 1 | 1 | 1 | 1 | 1 | 1 | 1 | 1 | 1 | 1 |
| 389 | DNA primase | 1 | 1 | 1 | 1 | 1 | 1 | 1 | 1 | 1 | 1 | 1 | 1 | 1 | 1 | 1 | 1 | 1 | 1 | 1 | 1 | 1 | 1 | 1 | 1 | 1 | 1 | 1 | 1 | 1 | 1 | 1 | 1 | 1 | 1 | 1 |
| 390 | RNA polymerase sigma factor RpoD | 1 | 1 | 1 | 1 | 1 | 1 | 1 | 1 | 1 | 1 | 1 | 1 | 1 | 1 | 1 | 1 | 1 | 1 | 1 | 1 | 1 | 1 | 1 | 1 | 1 | 1 | 1 | 1 | 1 | 1 | 1 | 1 | 1 | 1 | 1 |
| 409 | Phosphoglycerate kinase | 1 | 1 | 1 | 1 | 1 | 1 | 1 | 1 | 1 | 1 | 1 | 1 | 1 | 1 | 1 | 1 | 1 | 1 | 1 | 1 | 1 | 1 | 1 | 1 | 1 | 1 | 1 | 1 | 1 | 1 | 1 | 1 | 1 | 1 | 1 |
| 410 | Fructose-bisphosphate aldolase class 2 | 1 | 1 | 1 | 1 | 1 | 1 | 1 | 1 | 1 | 1 | 1 | 1 | 1 | 1 | 1 | 1 | 1 | 1 | 1 | 1 | 1 | 1 | 1 | 1 | 1 | 1 | 1 | 1 | 1 | 1 | 1 | 1 | 1 | 1 | 1 |
| 411 | Ribose-5-phosphate isomerase A | 1 | 1 | 1 | 1 | 1 | 1 | 1 | 1 | 1 | 1 | 1 | 1 | 1 | 1 | 1 | 1 | 1 | 1 | 1 | 1 | 1 | 1 | 1 | 1 | 1 | 1 | 1 | 1 | 1 | 1 | 1 | 1 | 1 | 1 | 1 |
| 412 | tRNA-modifying protein YgfZ | 1 | 1 | 1 | 1 | 1 | 1 | 1 | 1 | 1 | 1 | 1 | 1 | 1 | 1 | 1 | 1 | 1 | 1 | 1 | 1 | 1 | 1 | 1 | 1 | 1 | 1 | 1 | 1 | 1 | 1 | 1 | 1 | 1 | 1 | 1 |
| 413 | Peptide chain release factor 2 | 1 | 1 | 1 | 1 | 1 | 1 | 1 | 1 | 1 | 1 | 1 | 1 | 1 | 1 | 1 | 1 | 1 | 1 | 1 | 1 | 1 | 1 | 1 | 1 | 1 | 1 | 1 | 1 | 1 | 1 | 1 | 1 | 1 | 1 | 1 |
| 414 | Lysine--tRNA ligase | 1 | 1 | 1 | 1 | 1 | 1 | 1 | 1 | 1 | 1 | 1 | 1 | 1 | 1 | 1 | 1 | 1 | 1 | 1 | 1 | 1 | 1 | 1 | 1 | 1 | 1 | 1 | 1 | 1 | 1 | 1 | 1 | 1 | 1 | 1 |
| 416 | Thymidylate synthase | 1 | 1 | 1 | 1 | 1 | 1 | 1 | 1 | 1 | 1 | 1 | 1 | 1 | 1 | 1 | 1 | 1 | 1 | 1 | 1 | 1 | 1 | 1 | 1 | 1 | 1 | 1 | 1 | 1 | 1 | 1 | 1 | 1 | 1 | 1 |
| 417 | RecBCD enzyme subunit RecC | 1 | 1 | 1 | 1 | 1 | 1 | 1 | 1 | 1 | 1 | 1 | 1 | 1 | 1 | 1 | 1 | 1 | 1 | 1 | 1 | 1 | 1 | 1 | 1 | 1 | 1 | 1 | 1 | 1 | 1 | 1 | 1 | 1 | 1 | 1 |
| 418 | RecBCD enzyme subunit RecB | 1 | 1 | 1 | 1 | 1 | 1 | 1 | 1 | 1 | 1 | 1 | 1 | 1 | 1 | 1 | 1 | 1 | 1 | 1 | 1 | 1 | 1 | 1 | 1 | 1 | 1 | 1 | 1 | 1 | 1 | 1 | 1 | 1 | 1 | 1 |
| 419 | RecBCD enzyme subunit RecD | 1 | 1 | 1 | 1 | 1 | 1 | 1 | 1 | 1 | 1 | 1 | 1 | 1 | 1 | 1 | 1 | 1 | 1 | 1 | 1 | 1 | 1 | 1 | 1 | 1 | 1 | 1 | 1 | 1 | 1 | 1 | 1 | 1 | 1 | 1 |
| 420 | 2,3,4,5-tetrahydropyridine-2,6-dicarboxylate N-succinyltransferase | 1 | 1 | 1 | 1 | 1 | 1 | 1 | 1 | 1 | 1 | 1 | 1 | 1 | 1 | 1 | 1 | 1 | 1 | 1 | 1 | 1 | 1 | 1 | 1 | 1 | 1 | 1 | 1 | 1 | 1 | 1 | 1 | 1 | 1 | 1 |
| 421 | Methionine aminopeptidase | 1 | 1 | 1 | 1 | 1 | 1 | 1 | 1 | 1 | 1 | 1 | 1 | 1 | 1 | 1 | 1 | 1 | 1 | 1 | 1 | 1 | 1 | 1 | 1 | 1 | 1 | 1 | 1 | 1 | 1 | 1 | 1 | 1 | 1 | 1 |
| 422 | 30S ribosomal protein S2 | 1 | 1 | 1 | 1 | 1 | 1 | 1 | 1 | 1 | 1 | 1 | 1 | 1 | 1 | 1 | 1 | 1 | 1 | 1 | 1 | 1 | 1 | 1 | 1 | 1 | 1 | 1 | 1 | 1 | 1 | 1 | 1 | 1 | 1 | 1 |
| 423 | Elongation factor Ts | 1 | 1 | 1 | 1 | 1 | 1 | 1 | 1 | 1 | 1 | 1 | 1 | 1 | 1 | 1 | 1 | 1 | 1 | 1 | 1 | 1 | 1 | 1 | 1 | 1 | 1 | 1 | 1 | 1 | 1 | 1 | 1 | 1 | 1 | 1 |
| 424 | Uridylate kinase | 1 | 1 | 1 | 1 | 1 | 1 | 1 | 1 | 1 | 1 | 1 | 1 | 1 | 1 | 1 | 1 | 1 | 1 | 1 | 1 | 1 | 1 | 1 | 1 | 1 | 1 | 1 | 1 | 1 | 1 | 1 | 1 | 1 | 1 | 1 |
| 425 | Ribosome-recycling factor | 1 | 1 | 1 | 1 | 1 | 1 | 1 | 1 | 1 | 1 | 1 | 1 | 1 | 1 | 1 | 1 | 1 | 1 | 1 | 1 | 1 | 1 | 1 | 1 | 1 | 1 | 1 | 1 | 1 | 1 | 1 | 1 | 1 | 1 | 1 |
| 426 | 1-deoxy-D-xylulose 5-phosphate reductoisomerase | 1 | 1 | 1 | 1 | 1 | 1 | 1 | 1 | 1 | 1 | 1 | 1 | 1 | 1 | 1 | 1 | 1 | 1 | 1 | 1 | 1 | 1 | 1 | 1 | 1 | 1 | 1 | 1 | 1 | 1 | 1 | 1 | 1 | 1 | 1 |
| 427 | Ditrans,polycis-undecaprenyl-diphosphate synthase ((2E,6E)-farnesyl-diphosphate specific) | 1 | 1 | 1 | 1 | 1 | 1 | 1 | 1 | 1 | 1 | 1 | 1 | 1 | 1 | 1 | 1 | 1 | 1 | 1 | 1 | 1 | 1 | 1 | 1 | 1 | 1 | 1 | 1 | 1 | 1 | 1 | 1 | 1 | 1 | 1 |
| 428 | Phosphatidate cytidylyltransferase | 1 | 1 | 1 | 1 | 1 | 1 | 1 | 1 | 1 | 1 | 1 | 1 | 1 | 1 | 1 | 1 | 1 | 1 | 1 | 1 | 1 | 1 | 1 | 1 | 1 | 1 | 1 | 1 | 1 | 1 | 1 | 1 | 1 | 1 | 1 |
| 429 | Chaperone protein Skp | 1 | 1 | 1 | 1 | 1 | 1 | 1 | 1 | 1 | 1 | 1 | 1 | 1 | 1 | 1 | 1 | 1 | 1 | 1 | 1 | 1 | 1 | 1 | 1 | 1 | 1 | 1 | 1 | 1 | 1 | 1 | 1 | 1 | 1 | 1 |
| 430 | UDP-3-O-(3-hydroxymyristoyl)glucosamine N-acyltransferase | 1 | 1 | 1 | 1 | 1 | 1 | 1 | 1 | 1 | 1 | 1 | 1 | 1 | 1 | 1 | 1 | 1 | 1 | 1 | 1 | 1 | 1 | 1 | 1 | 1 | 1 | 1 | 1 | 1 | 1 | 1 | 1 | 1 | 1 | 1 |
| 431 | 3-hydroxyacyl-[acyl-carrier-protein] dehydratase FabZ | 1 | 1 | 1 | 1 | 1 | 1 | 1 | 1 | 1 | 1 | 1 | 1 | 1 | 1 | 1 | 1 | 1 | 1 | 1 | 1 | 1 | 1 | 1 | 1 | 1 | 1 | 1 | 1 | 1 | 1 | 1 | 1 | 1 | 1 | 1 |
| 432 | Acyl-[acyl-carrier-protein]--UDP-N-acetylglucosamine O-acyltransferase | 1 | 1 | 1 | 1 | 1 | 1 | 1 | 1 | 1 | 1 | 1 | 1 | 1 | 1 | 1 | 1 | 1 | 1 | 1 | 1 | 1 | 1 | 1 | 1 | 1 | 1 | 1 | 1 | 1 | 1 | 1 | 1 | 1 | 1 | 1 |
| 433 | Lipid-A-disaccharide synthase | 1 | 1 | 1 | 1 | 1 | 1 | 1 | 1 | 1 | 1 | 1 | 1 | 1 | 1 | 1 | 1 | 1 | 1 | 1 | 1 | 1 | 1 | 1 | 1 | 1 | 1 | 1 | 1 | 1 | 1 | 1 | 1 | 1 | 1 | 1 |
| 434 | DNA polymerase III subunit alpha | 1 | 1 | 1 | 1 | 1 | 1 | 1 | 1 | 1 | 1 | 1 | 1 | 1 | 1 | 1 | 1 | 1 | 1 | 1 | 1 | 1 | 1 | 1 | 1 | 1 | 1 | 1 | 1 | 1 | 1 | 1 | 1 | 1 | 1 | 1 |
| 435 | Acetyl-coenzyme A carboxylase carboxyl transferase subunit alpha | 1 | 1 | 1 | 1 | 1 | 1 | 1 | 1 | 1 | 1 | 1 | 1 | 1 | 1 | 1 | 1 | 1 | 1 | 1 | 1 | 1 | 1 | 1 | 1 | 1 | 1 | 1 | 1 | 1 | 1 | 1 | 1 | 1 | 1 | 1 |
| 436 | tRNA(Ile)-lysidine synthase | 1 | 1 | 1 | 1 | 1 | 1 | 1 | 1 | 1 | 1 | 1 | 1 | 1 | 1 | 1 | 1 | 1 | 1 | 1 | 1 | 1 | 1 | 1 | 1 | 1 | 1 | 1 | 1 | 1 | 1 | 1 | 1 | 1 | 1 | 1 |
| 437 | D-glycero-beta-D-manno-heptose-1,7-bisphosphate 7-phosphatase | 1 | 1 | 1 | 1 | 1 | 1 | 1 | 1 | 1 | 1 | 1 | 1 | 1 | 1 | 1 | 1 | 1 | 1 | 1 | 1 | 1 | 1 | 1 | 1 | 1 | 1 | 1 | 1 | 1 | 1 | 1 | 1 | 1 | 1 | 1 |
| 438 | Succinyl-diaminopimelate desuccinylase | 1 | 1 | 1 | 1 | 1 | 1 | 1 | 1 | 1 | 1 | 1 | 1 | 1 | 1 | 1 | 1 | 1 | 1 | 1 | 1 | 1 | 1 | 1 | 1 | 1 | 1 | 1 | 1 | 1 | 1 | 1 | 1 | 1 | 1 | 1 |
| 439 | 4-hydroxy-tetrahydrodipicolinate synthase | 1 | 1 | 1 | 1 | 1 | 1 | 1 | 1 | 1 | 1 | 1 | 1 | 1 | 1 | 1 | 1 | 1 | 1 | 1 | 1 | 1 | 1 | 1 | 1 | 1 | 1 | 1 | 1 | 1 | 1 | 1 | 1 | 1 | 1 | 1 |
| 440 | GMP synthase [glutamine-hydrolyzing] | 1 | 1 | 1 | 1 | 1 | 1 | 1 | 1 | 1 | 1 | 1 | 1 | 1 | 1 | 1 | 1 | 1 | 1 | 1 | 1 | 1 | 1 | 1 | 1 | 1 | 1 | 1 | 1 | 1 | 1 | 1 | 1 | 1 | 1 | 1 |
| 441 | Inosine-5-monophosphate dehydrogenase | 1 | 1 | 1 | 1 | 1 | 1 | 1 | 1 | 1 | 1 | 1 | 1 | 1 | 1 | 1 | 1 | 1 | 1 | 1 | 1 | 1 | 1 | 1 | 1 | 1 | 1 | 1 | 1 | 1 | 1 | 1 | 1 | 1 | 1 | 1 |
| 442 | GTPase Der | 1 | 1 | 1 | 1 | 1 | 1 | 1 | 1 | 1 | 1 | 1 | 1 | 1 | 1 | 1 | 1 | 1 | 1 | 1 | 1 | 1 | 1 | 1 | 1 | 1 | 1 | 1 | 1 | 1 | 1 | 1 | 1 | 1 | 1 | 1 |
| 443 | hypothetical protein | 1 | 1 | 1 | 1 | 1 | 1 | 1 | 1 | 1 | 1 | 1 | 1 | 1 | 1 | 1 | 1 | 1 | 1 | 1 | 1 | 1 | 1 | 1 | 1 | 1 | 1 | 1 | 1 | 1 | 1 | 1 | 1 | 1 | 1 | 1 |
| 444 | Histidine--tRNA ligase | 1 | 1 | 1 | 1 | 1 | 1 | 1 | 1 | 1 | 1 | 1 | 1 | 1 | 1 | 1 | 1 | 1 | 1 | 1 | 1 | 1 | 1 | 1 | 1 | 1 | 1 | 1 | 1 | 1 | 1 | 1 | 1 | 1 | 1 | 1 |
| 445 | 4-hydroxy-3-methylbut-2-en-1-yl diphosphate synthase (flavodoxin) | 1 | 1 | 1 | 1 | 1 | 1 | 1 | 1 | 1 | 1 | 1 | 1 | 1 | 1 | 1 | 1 | 1 | 1 | 1 | 1 | 1 | 1 | 1 | 1 | 1 | 1 | 1 | 1 | 1 | 1 | 1 | 1 | 1 | 1 | 1 |
| 446 | Dual-specificity RNA methyltransferase RlmN | 1 | 1 | 1 | 1 | 1 | 1 | 1 | 1 | 1 | 1 | 1 | 1 | 1 | 1 | 1 | 1 | 1 | 1 | 1 | 1 | 1 | 1 | 1 | 1 | 1 | 1 | 1 | 1 | 1 | 1 | 1 | 1 | 1 | 1 | 1 |
| 447 | Cysteine desulfurase IscS | 1 | 1 | 1 | 1 | 1 | 1 | 1 | 1 | 1 | 1 | 1 | 1 | 1 | 1 | 1 | 1 | 1 | 1 | 1 | 1 | 1 | 1 | 1 | 1 | 1 | 1 | 1 | 1 | 1 | 1 | 1 | 1 | 1 | 1 | 1 |
| 448 | Inositol-1-monophosphatase | 1 | 1 | 1 | 1 | 1 | 1 | 1 | 1 | 1 | 1 | 1 | 1 | 1 | 1 | 1 | 1 | 1 | 1 | 1 | 1 | 1 | 1 | 1 | 1 | 1 | 1 | 1 | 1 | 1 | 1 | 1 | 1 | 1 | 1 | 1 |
| 449 | tRNA-specific adenosine deaminase | 1 | 1 | 1 | 1 | 1 | 1 | 1 | 1 | 1 | 1 | 1 | 1 | 1 | 1 | 1 | 1 | 1 | 1 | 1 | 1 | 1 | 1 | 1 | 1 | 1 | 1 | 1 | 1 | 1 | 1 | 1 | 1 | 1 | 1 | 1 |
| 450 | Holo-[acyl-carrier-protein] synthase | 1 | 1 | 1 | 1 | 1 | 1 | 1 | 1 | 1 | 1 | 1 | 1 | 1 | 1 | 1 | 1 | 1 | 1 | 1 | 1 | 1 | 1 | 1 | 1 | 1 | 1 | 1 | 1 | 1 | 1 | 1 | 1 | 1 | 1 | 1 |
| 451 | GTPase Era | 1 | 1 | 1 | 1 | 1 | 1 | 1 | 1 | 1 | 1 | 1 | 1 | 1 | 1 | 1 | 1 | 1 | 1 | 1 | 1 | 1 | 1 | 1 | 1 | 1 | 1 | 1 | 1 | 1 | 1 | 1 | 1 | 1 | 1 | 1 |
| 452 | Ribonuclease 3 | 1 | 1 | 1 | 1 | 1 | 1 | 1 | 1 | 1 | 1 | 1 | 1 | 1 | 1 | 1 | 1 | 1 | 1 | 1 | 1 | 1 | 1 | 1 | 1 | 1 | 1 | 1 | 1 | 1 | 1 | 1 | 1 | 1 | 1 | 1 |
| 453 | Signal peptidase I | 1 | 1 | 1 | 1 | 1 | 1 | 1 | 1 | 1 | 1 | 1 | 1 | 1 | 1 | 1 | 1 | 1 | 1 | 1 | 1 | 1 | 1 | 1 | 1 | 1 | 1 | 1 | 1 | 1 | 1 | 1 | 1 | 1 | 1 | 1 |
| 454 | Elongation factor 4 | 1 | 1 | 1 | 1 | 1 | 1 | 1 | 1 | 1 | 1 | 1 | 1 | 1 | 1 | 1 | 1 | 1 | 1 | 1 | 1 | 1 | 1 | 1 | 1 | 1 | 1 | 1 | 1 | 1 | 1 | 1 | 1 | 1 | 1 | 1 |
| 455 | Protein GrpE | 1 | 1 | 1 | 1 | 1 | 1 | 1 | 1 | 1 | 1 | 1 | 1 | 1 | 1 | 1 | 1 | 1 | 1 | 1 | 1 | 1 | 1 | 1 | 1 | 1 | 1 | 1 | 1 | 1 | 1 | 1 | 1 | 1 | 1 | 1 |
| 456 | NAD kinase | 1 | 1 | 1 | 1 | 1 | 1 | 1 | 1 | 1 | 1 | 1 | 1 | 1 | 1 | 1 | 1 | 1 | 1 | 1 | 1 | 1 | 1 | 1 | 1 | 1 | 1 | 1 | 1 | 1 | 1 | 1 | 1 | 1 | 1 | 1 |
| 457 | Persistence and stress-resistance antitoxin PasI | 1 | 1 | 1 | 1 | 1 | 1 | 1 | 1 | 1 | 1 | 1 | 1 | 1 | 1 | 1 | 1 | 1 | 1 | 1 | 1 | 1 | 1 | 1 | 1 | 1 | 1 | 1 | 1 | 1 | 1 | 1 | 1 | 1 | 1 | 1 |
| 458 | Adenylate kinase | 1 | 1 | 1 | 1 | 1 | 1 | 1 | 1 | 1 | 1 | 1 | 1 | 1 | 1 | 1 | 1 | 1 | 1 | 1 | 1 | 1 | 1 | 1 | 1 | 1 | 1 | 1 | 1 | 1 | 1 | 1 | 1 | 1 | 1 | 1 |
| 459 | DNA polymerase III subunit tau | 1 | 1 | 1 | 1 | 1 | 1 | 1 | 1 | 1 | 1 | 1 | 1 | 1 | 1 | 1 | 1 | 1 | 1 | 1 | 1 | 1 | 1 | 1 | 1 | 1 | 1 | 1 | 1 | 1 | 1 | 1 | 1 | 1 | 1 | 1 |
| 460 | Peptidyl-prolyl cis-trans isomerase D | 1 | 1 | 1 | 1 | 1 | 1 | 1 | 1 | 1 | 1 | 1 | 1 | 1 | 1 | 1 | 1 | 1 | 1 | 1 | 1 | 1 | 1 | 1 | 1 | 1 | 1 | 1 | 1 | 1 | 1 | 1 | 1 | 1 | 1 | 1 |
| 461 | Lon protease | 1 | 1 | 1 | 1 | 1 | 1 | 1 | 1 | 1 | 1 | 1 | 1 | 1 | 1 | 1 | 1 | 1 | 1 | 1 | 1 | 1 | 1 | 1 | 1 | 1 | 1 | 1 | 1 | 1 | 1 | 1 | 1 | 1 | 1 | 1 |
| 462 | ATP-dependent Clp protease ATP-binding subunit ClpX | 1 | 1 | 1 | 1 | 1 | 1 | 1 | 1 | 1 | 1 | 1 | 1 | 1 | 1 | 1 | 1 | 1 | 1 | 1 | 1 | 1 | 1 | 1 | 1 | 1 | 1 | 1 | 1 | 1 | 1 | 1 | 1 | 1 | 1 | 1 |
| 463 | ATP-dependent Clp protease proteolytic subunit | 1 | 1 | 1 | 1 | 1 | 1 | 1 | 1 | 1 | 1 | 1 | 1 | 1 | 1 | 1 | 1 | 1 | 1 | 1 | 1 | 1 | 1 | 1 | 1 | 1 | 1 | 1 | 1 | 1 | 1 | 1 | 1 | 1 | 1 | 1 |
| 464 | Cytochrome bo(3) ubiquinol oxidase subunit 2 | 1 | 1 | 1 | 1 | 1 | 1 | 1 | 1 | 1 | 1 | 1 | 1 | 1 | 1 | 1 | 1 | 1 | 1 | 1 | 1 | 1 | 1 | 1 | 1 | 1 | 1 | 1 | 1 | 1 | 1 | 1 | 1 | 1 | 1 | 1 |
| 465 | Cytochrome bo(3) ubiquinol oxidase subunit 1 | 1 | 1 | 1 | 1 | 1 | 1 | 1 | 1 | 1 | 1 | 1 | 1 | 1 | 1 | 1 | 1 | 1 | 1 | 1 | 1 | 1 | 1 | 1 | 1 | 1 | 1 | 1 | 1 | 1 | 1 | 1 | 1 | 1 | 1 | 1 |
| 466 | Cytochrome bo(3) ubiquinol oxidase subunit 3 | 1 | 1 | 1 | 1 | 1 | 1 | 1 | 1 | 1 | 1 | 1 | 1 | 1 | 1 | 1 | 1 | 1 | 1 | 1 | 1 | 1 | 1 | 1 | 1 | 1 | 1 | 1 | 1 | 1 | 1 | 1 | 1 | 1 | 1 | 1 |
| 467 | Cytochrome bo(3) ubiquinol oxidase subunit 4 | 1 | 1 | 1 | 1 | 1 | 1 | 1 | 1 | 1 | 1 | 1 | 1 | 1 | 1 | 1 | 1 | 1 | 1 | 1 | 1 | 1 | 1 | 1 | 1 | 1 | 1 | 1 | 1 | 1 | 1 | 1 | 1 | 1 | 1 | 1 |
| 468 | Protoheme IX farnesyltransferase | 1 | 1 | 1 | 1 | 1 | 1 | 1 | 1 | 1 | 1 | 1 | 1 | 1 | 1 | 1 | 1 | 1 | 1 | 1 | 1 | 1 | 1 | 1 | 1 | 1 | 1 | 1 | 1 | 1 | 1 | 1 | 1 | 1 | 1 | 1 |
| 469 | Farnesyl diphosphate synthase | 1 | 1 | 1 | 1 | 1 | 1 | 1 | 1 | 1 | 1 | 1 | 1 | 1 | 1 | 1 | 1 | 1 | 1 | 1 | 1 | 1 | 1 | 1 | 1 | 1 | 1 | 1 | 1 | 1 | 1 | 1 | 1 | 1 | 1 | 1 |
| 470 | Thiamine-monophosphate kinase | 1 | 1 | 1 | 1 | 1 | 1 | 1 | 1 | 1 | 1 | 1 | 1 | 1 | 1 | 1 | 1 | 1 | 1 | 1 | 1 | 1 | 1 | 1 | 1 | 1 | 1 | 1 | 1 | 1 | 1 | 1 | 1 | 1 | 1 | 1 |
| 471 | N utilization substance protein B | 1 | 1 | 1 | 1 | 1 | 1 | 1 | 1 | 1 | 1 | 1 | 1 | 1 | 1 | 1 | 1 | 1 | 1 | 1 | 1 | 1 | 1 | 1 | 1 | 1 | 1 | 1 | 1 | 1 | 1 | 1 | 1 | 1 | 1 | 1 |
| 472 | 6,7-dimethyl-8-ribityllumazine synthase | 1 | 1 | 1 | 1 | 1 | 1 | 1 | 1 | 1 | 1 | 1 | 1 | 1 | 1 | 1 | 1 | 1 | 1 | 1 | 1 | 1 | 1 | 1 | 1 | 1 | 1 | 1 | 1 | 1 | 1 | 1 | 1 | 1 | 1 | 1 |
| 473 | Riboflavin biosynthesis protein RibD | 1 | 1 | 1 | 1 | 1 | 1 | 1 | 1 | 1 | 1 | 1 | 1 | 1 | 1 | 1 | 1 | 1 | 1 | 1 | 1 | 1 | 1 | 1 | 1 | 1 | 1 | 1 | 1 | 1 | 1 | 1 | 1 | 1 | 1 | 1 |
| 474 | hypothetical protein | 1 | 1 | 1 | 1 | 1 | 1 | 1 | 1 | 1 | 1 | 1 | 1 | 1 | 1 | 1 | 1 | 1 | 1 | 1 | 1 | 1 | 1 | 1 | 1 | 1 | 1 | 1 | 1 | 1 | 1 | 1 | 1 | 1 | 1 | 1 |
| 475 | hypothetical protein | 1 | 1 | 1 | 1 | 1 | 1 | 1 | 1 | 1 | 1 | 1 | 1 | 1 | 1 | 1 | 1 | 1 | 1 | 1 | 1 | 1 | 1 | 1 | 1 | 1 | 1 | 1 | 1 | 1 | 1 | 1 | 1 | 1 | 1 | 1 |
| 476 | hypothetical protein | 1 | 1 | 1 | 1 | 1 | 1 | 1 | 1 | 1 | 1 | 1 | 1 | 1 | 1 | 1 | 1 | 1 | 1 | 1 | 1 | 1 | 1 | 1 | 1 | 1 | 1 | 1 | 1 | 1 | 1 | 1 | 1 | 1 | 1 | 1 |
| 477 | Phosphoheptose isomerase | 1 | 1 | 1 | 1 | 1 | 1 | 1 | 1 | 1 | 1 | 1 | 1 | 1 | 1 | 1 | 1 | 1 | 1 | 1 | 1 | 1 | 1 | 1 | 1 | 1 | 1 | 1 | 1 | 1 | 1 | 1 | 1 | 1 | 1 | 1 |
| 478 | DNA polymerase III subunit epsilon | 1 | 1 | 1 | 1 | 1 | 1 | 1 | 1 | 1 | 1 | 1 | 1 | 1 | 1 | 1 | 1 | 1 | 1 | 1 | 1 | 1 | 1 | 1 | 1 | 1 | 1 | 1 | 1 | 1 | 1 | 1 | 1 | 1 | 1 | 1 |
| 479 | Ribonuclease HI | 1 | 1 | 1 | 1 | 1 | 1 | 1 | 1 | 1 | 1 | 1 | 1 | 1 | 1 | 1 | 1 | 1 | 1 | 1 | 1 | 1 | 1 | 1 | 1 | 1 | 1 | 1 | 1 | 1 | 1 | 1 | 1 | 1 | 1 | 1 |
| 480 | Hydroxyacylglutathione hydrolase | 1 | 1 | 1 | 1 | 1 | 1 | 1 | 1 | 1 | 1 | 1 | 1 | 1 | 1 | 1 | 1 | 1 | 1 | 1 | 1 | 1 | 1 | 1 | 1 | 1 | 1 | 1 | 1 | 1 | 1 | 1 | 1 | 1 | 1 | 1 |
| 481 | Exodeoxyribonuclease I | 1 | 1 | 1 | 1 | 1 | 1 | 1 | 1 | 1 | 1 | 1 | 1 | 1 | 1 | 1 | 1 | 1 | 1 | 1 | 1 | 1 | 1 | 1 | 1 | 1 | 1 | 1 | 1 | 1 | 1 | 1 | 1 | 1 | 1 | 1 |
| 482 | Lysine-specific permease | 1 | 1 | 1 | 1 | 1 | 1 | 1 | 1 | 1 | 1 | 1 | 1 | 1 | 1 | 1 | 1 | 1 | 1 | 1 | 1 | 1 | 1 | 1 | 1 | 1 | 1 | 1 | 1 | 1 | 1 | 1 | 1 | 1 | 1 | 1 |
| 483 | 50S ribosomal protein L25 | 1 | 1 | 1 | 1 | 1 | 1 | 1 | 1 | 1 | 1 | 1 | 1 | 1 | 1 | 1 | 1 | 1 | 1 | 1 | 1 | 1 | 1 | 1 | 1 | 1 | 1 | 1 | 1 | 1 | 1 | 1 | 1 | 1 | 1 | 1 |
| 485 | Ribonucleoside-diphosphate reductase 1 subunit alpha | 1 | 1 | 1 | 1 | 1 | 1 | 1 | 1 | 1 | 1 | 1 | 1 | 1 | 1 | 1 | 1 | 1 | 1 | 1 | 1 | 1 | 1 | 1 | 1 | 1 | 1 | 1 | 1 | 1 | 1 | 1 | 1 | 1 | 1 | 1 |
| 486 | Ribonucleoside-diphosphate reductase 1 subunit beta | 1 | 1 | 1 | 1 | 1 | 1 | 1 | 1 | 1 | 1 | 1 | 1 | 1 | 1 | 1 | 1 | 1 | 1 | 1 | 1 | 1 | 1 | 1 | 1 | 1 | 1 | 1 | 1 | 1 | 1 | 1 | 1 | 1 | 1 | 1 |
| 487 | Acetate kinase | 1 | 1 | 1 | 1 | 1 | 1 | 1 | 1 | 1 | 1 | 1 | 1 | 1 | 1 | 1 | 1 | 1 | 1 | 1 | 1 | 1 | 1 | 1 | 1 | 1 | 1 | 1 | 1 | 1 | 1 | 1 | 1 | 1 | 1 | 1 |
| 488 | Phosphate acetyltransferase | 1 | 1 | 1 | 1 | 1 | 1 | 1 | 1 | 1 | 1 | 1 | 1 | 1 | 1 | 1 | 1 | 1 | 1 | 1 | 1 | 1 | 1 | 1 | 1 | 1 | 1 | 1 | 1 | 1 | 1 | 1 | 1 | 1 | 1 | 1 |
| 489 | Colicin V production protein | 1 | 1 | 1 | 1 | 1 | 1 | 1 | 1 | 1 | 1 | 1 | 1 | 1 | 1 | 1 | 1 | 1 | 1 | 1 | 1 | 1 | 1 | 1 | 1 | 1 | 1 | 1 | 1 | 1 | 1 | 1 | 1 | 1 | 1 | 1 |
| 490 | Bifunctional protein FolC | 1 | 1 | 1 | 1 | 1 | 1 | 1 | 1 | 1 | 1 | 1 | 1 | 1 | 1 | 1 | 1 | 1 | 1 | 1 | 1 | 1 | 1 | 1 | 1 | 1 | 1 | 1 | 1 | 1 | 1 | 1 | 1 | 1 | 1 | 1 |
| 491 | Acetyl-coenzyme A carboxylase carboxyl transferase subunit beta | 1 | 1 | 1 | 1 | 1 | 1 | 1 | 1 | 1 | 1 | 1 | 1 | 1 | 1 | 1 | 1 | 1 | 1 | 1 | 1 | 1 | 1 | 1 | 1 | 1 | 1 | 1 | 1 | 1 | 1 | 1 | 1 | 1 | 1 | 1 |
| 492 | 3-oxoacyl-[acyl-carrier-protein] synthase 1 | 1 | 1 | 1 | 1 | 1 | 1 | 1 | 1 | 1 | 1 | 1 | 1 | 1 | 1 | 1 | 1 | 1 | 1 | 1 | 1 | 1 | 1 | 1 | 1 | 1 | 1 | 1 | 1 | 1 | 1 | 1 | 1 | 1 | 1 | 1 |
| 496 | Electron transport complex subunit RsxA | 1 | 1 | 1 | 1 | 1 | 1 | 1 | 1 | 1 | 1 | 1 | 1 | 1 | 1 | 1 | 1 | 1 | 1 | 1 | 1 | 1 | 1 | 1 | 1 | 1 | 1 | 1 | 1 | 1 | 1 | 1 | 1 | 1 | 1 | 1 |
| 497 | Electron transport complex subunit RsxB | 1 | 1 | 1 | 1 | 1 | 1 | 1 | 1 | 1 | 1 | 1 | 1 | 1 | 1 | 1 | 1 | 1 | 1 | 1 | 1 | 1 | 1 | 1 | 1 | 1 | 1 | 1 | 1 | 1 | 1 | 1 | 1 | 1 | 1 | 1 |
| 498 | Electron transport complex subunit RsxD | 1 | 1 | 1 | 1 | 1 | 1 | 1 | 1 | 1 | 1 | 1 | 1 | 1 | 1 | 1 | 1 | 1 | 1 | 1 | 1 | 1 | 1 | 1 | 1 | 1 | 1 | 1 | 1 | 1 | 1 | 1 | 1 | 1 | 1 | 1 |
| 499 | Electron transport complex subunit RsxG | 1 | 1 | 1 | 1 | 1 | 1 | 1 | 1 | 1 | 1 | 1 | 1 | 1 | 1 | 1 | 1 | 1 | 1 | 1 | 1 | 1 | 1 | 1 | 1 | 1 | 1 | 1 | 1 | 1 | 1 | 1 | 1 | 1 | 1 | 1 |
| 500 | Electron transport complex subunit RsxE | 1 | 1 | 1 | 1 | 1 | 1 | 1 | 1 | 1 | 1 | 1 | 1 | 1 | 1 | 1 | 1 | 1 | 1 | 1 | 1 | 1 | 1 | 1 | 1 | 1 | 1 | 1 | 1 | 1 | 1 | 1 | 1 | 1 | 1 | 1 |
| 501 | Endonuclease III | 1 | 1 | 1 | 1 | 1 | 1 | 1 | 1 | 1 | 1 | 1 | 1 | 1 | 1 | 1 | 1 | 1 | 1 | 1 | 1 | 1 | 1 | 1 | 1 | 1 | 1 | 1 | 1 | 1 | 1 | 1 | 1 | 1 | 1 | 1 |
| 502 | Tyrosine--tRNA ligase | 1 | 1 | 1 | 1 | 1 | 1 | 1 | 1 | 1 | 1 | 1 | 1 | 1 | 1 | 1 | 1 | 1 | 1 | 1 | 1 | 1 | 1 | 1 | 1 | 1 | 1 | 1 | 1 | 1 | 1 | 1 | 1 | 1 | 1 | 1 |
| 503 | Ribonuclease T | 1 | 1 | 1 | 1 | 1 | 1 | 1 | 1 | 1 | 1 | 1 | 1 | 1 | 1 | 1 | 1 | 1 | 1 | 1 | 1 | 1 | 1 | 1 | 1 | 1 | 1 | 1 | 1 | 1 | 1 | 1 | 1 | 1 | 1 | 1 |
| 504 | Glutaredoxin-4 | 1 | 1 | 1 | 1 | 1 | 1 | 1 | 1 | 1 | 1 | 1 | 1 | 1 | 1 | 1 | 1 | 1 | 1 | 1 | 1 | 1 | 1 | 1 | 1 | 1 | 1 | 1 | 1 | 1 | 1 | 1 | 1 | 1 | 1 | 1 |
| 505 | Riboflavin synthase | 1 | 1 | 1 | 1 | 1 | 1 | 1 | 1 | 1 | 1 | 1 | 1 | 1 | 1 | 1 | 1 | 1 | 1 | 1 | 1 | 1 | 1 | 1 | 1 | 1 | 1 | 1 | 1 | 1 | 1 | 1 | 1 | 1 | 1 | 1 |
| 506 | Pyruvate kinase I | 1 | 1 | 1 | 1 | 1 | 1 | 1 | 1 | 1 | 1 | 1 | 1 | 1 | 1 | 1 | 1 | 1 | 1 | 1 | 1 | 1 | 1 | 1 | 1 | 1 | 1 | 1 | 1 | 1 | 1 | 1 | 1 | 1 | 1 | 1 |
| 507 | hypothetical protein | 1 | 1 | 1 | 1 | 1 | 1 | 1 | 1 | 1 | 1 | 1 | 1 | 1 | 1 | 1 | 1 | 1 | 1 | 1 | 1 | 1 | 1 | 1 | 1 | 1 | 1 | 1 | 1 | 1 | 1 | 1 | 1 | 1 | 1 | 1 |
| 508 | Integration host factor subunit alpha | 1 | 1 | 1 | 1 | 1 | 1 | 1 | 1 | 1 | 1 | 1 | 1 | 1 | 1 | 1 | 1 | 1 | 1 | 1 | 1 | 1 | 1 | 1 | 1 | 1 | 1 | 1 | 1 | 1 | 1 | 1 | 1 | 1 | 1 | 1 |
| 509 | Phenylalanine--tRNA ligase beta subunit | 1 | 1 | 1 | 1 | 1 | 1 | 1 | 1 | 1 | 1 | 1 | 1 | 1 | 1 | 1 | 1 | 1 | 1 | 1 | 1 | 1 | 1 | 1 | 1 | 1 | 1 | 1 | 1 | 1 | 1 | 1 | 1 | 1 | 1 | 1 |
| 510 | Phenylalanine--tRNA ligase alpha subunit | 1 | 1 | 1 | 1 | 1 | 1 | 1 | 1 | 1 | 1 | 1 | 1 | 1 | 1 | 1 | 1 | 1 | 1 | 1 | 1 | 1 | 1 | 1 | 1 | 1 | 1 | 1 | 1 | 1 | 1 | 1 | 1 | 1 | 1 | 1 |
| 511 | 50S ribosomal protein L20 | 1 | 1 | 1 | 1 | 1 | 1 | 1 | 1 | 1 | 1 | 1 | 1 | 1 | 1 | 1 | 1 | 1 | 1 | 1 | 1 | 1 | 1 | 1 | 1 | 1 | 1 | 1 | 1 | 1 | 1 | 1 | 1 | 1 | 1 | 1 |
| 512 | 50S ribosomal protein L35 | 1 | 1 | 1 | 1 | 1 | 1 | 1 | 1 | 1 | 1 | 1 | 1 | 1 | 1 | 1 | 1 | 1 | 1 | 1 | 1 | 1 | 1 | 1 | 1 | 1 | 1 | 1 | 1 | 1 | 1 | 1 | 1 | 1 | 1 | 1 |
| 513 | Translation initiation factor IF-3 | 1 | 1 | 1 | 1 | 1 | 1 | 1 | 1 | 1 | 1 | 1 | 1 | 1 | 1 | 1 | 1 | 1 | 1 | 1 | 1 | 1 | 1 | 1 | 1 | 1 | 1 | 1 | 1 | 1 | 1 | 1 | 1 | 1 | 1 | 1 |
| 514 | Threonine--tRNA ligase | 1 | 1 | 1 | 1 | 1 | 1 | 1 | 1 | 1 | 1 | 1 | 1 | 1 | 1 | 1 | 1 | 1 | 1 | 1 | 1 | 1 | 1 | 1 | 1 | 1 | 1 | 1 | 1 | 1 | 1 | 1 | 1 | 1 | 1 | 1 |
| 515 | Protease HtpX | 1 | 1 | 1 | 1 | 1 | 1 | 1 | 1 | 1 | 1 | 1 | 1 | 1 | 1 | 1 | 1 | 1 | 1 | 1 | 1 | 1 | 1 | 1 | 1 | 1 | 1 | 1 | 1 | 1 | 1 | 1 | 1 | 1 | 1 | 1 |
| 516 | tRNA-specific 2-thiouridylase MnmA | 1 | 1 | 1 | 1 | 1 | 1 | 1 | 1 | 1 | 1 | 1 | 1 | 1 | 1 | 1 | 1 | 1 | 1 | 1 | 1 | 1 | 1 | 1 | 1 | 1 | 1 | 1 | 1 | 1 | 1 | 1 | 1 | 1 | 1 | 1 |
| 517 | High frequency lysogenization protein HflD | 1 | 1 | 1 | 1 | 1 | 1 | 1 | 1 | 1 | 1 | 1 | 1 | 1 | 1 | 1 | 1 | 1 | 1 | 1 | 1 | 1 | 1 | 1 | 1 | 1 | 1 | 1 | 1 | 1 | 1 | 1 | 1 | 1 | 1 | 1 |
| 518 | Adenylosuccinate lyase | 1 | 1 | 1 | 1 | 1 | 1 | 1 | 1 | 1 | 1 | 1 | 1 | 1 | 1 | 1 | 1 | 1 | 1 | 1 | 1 | 1 | 1 | 1 | 1 | 1 | 1 | 1 | 1 | 1 | 1 | 1 | 1 | 1 | 1 | 1 |
| 520 | Exoribonuclease 2 | 1 | 1 | 1 | 1 | 1 | 1 | 1 | 1 | 1 | 1 | 1 | 1 | 1 | 1 | 1 | 1 | 1 | 1 | 1 | 1 | 1 | 1 | 1 | 1 | 1 | 1 | 1 | 1 | 1 | 1 | 1 | 1 | 1 | 1 | 1 |
| 521 | Lipopolysaccharide assembly protein A | 1 | 1 | 1 | 1 | 1 | 1 | 1 | 1 | 1 | 1 | 1 | 1 | 1 | 1 | 1 | 1 | 1 | 1 | 1 | 1 | 1 | 1 | 1 | 1 | 1 | 1 | 1 | 1 | 1 | 1 | 1 | 1 | 1 | 1 | 1 |
| 522 | GTP cyclohydrolase-2 | 1 | 1 | 1 | 1 | 1 | 1 | 1 | 1 | 1 | 1 | 1 | 1 | 1 | 1 | 1 | 1 | 1 | 1 | 1 | 1 | 1 | 1 | 1 | 1 | 1 | 1 | 1 | 1 | 1 | 1 | 1 | 1 | 1 | 1 | 1 |
| 523 | DNA topoisomerase 1 | 1 | 1 | 1 | 1 | 1 | 1 | 1 | 1 | 1 | 1 | 1 | 1 | 1 | 1 | 1 | 1 | 1 | 1 | 1 | 1 | 1 | 1 | 1 | 1 | 1 | 1 | 1 | 1 | 1 | 1 | 1 | 1 | 1 | 1 | 1 |
| 524 | Bifunctional protein TrpGD | 1 | 1 | 1 | 1 | 1 | 1 | 1 | 1 | 1 | 1 | 1 | 1 | 1 | 1 | 1 | 1 | 1 | 1 | 1 | 1 | 1 | 1 | 1 | 1 | 1 | 1 | 1 | 1 | 1 | 1 | 1 | 1 | 1 | 1 | 1 |
| 525 | Tryptophan biosynthesis protein TrpCF | 1 | 1 | 1 | 1 | 1 | 1 | 1 | 1 | 1 | 1 | 1 | 1 | 1 | 1 | 1 | 1 | 1 | 1 | 1 | 1 | 1 | 1 | 1 | 1 | 1 | 1 | 1 | 1 | 1 | 1 | 1 | 1 | 1 | 1 | 1 |
| 526 | Tryptophan synthase alpha chain | 1 | 1 | 1 | 1 | 1 | 1 | 1 | 1 | 1 | 1 | 1 | 1 | 1 | 1 | 1 | 1 | 1 | 1 | 1 | 1 | 1 | 1 | 1 | 1 | 1 | 1 | 1 | 1 | 1 | 1 | 1 | 1 | 1 | 1 | 1 |
| 527 | hypothetical protein | 1 | 1 | 1 | 1 | 1 | 1 | 1 | 1 | 1 | 1 | 1 | 1 | 1 | 1 | 1 | 1 | 1 | 1 | 1 | 1 | 1 | 1 | 1 | 1 | 1 | 1 | 1 | 1 | 1 | 1 | 1 | 1 | 1 | 1 | 1 |
| 528 | putative intracellular septation protein A | 1 | 1 | 1 | 1 | 1 | 1 | 1 | 1 | 1 | 1 | 1 | 1 | 1 | 1 | 1 | 1 | 1 | 1 | 1 | 1 | 1 | 1 | 1 | 1 | 1 | 1 | 1 | 1 | 1 | 1 | 1 | 1 | 1 | 1 | 1 |
| 529 | Ribosomal large subunit pseudouridine synthase D | 1 | 1 | 1 | 1 | 1 | 1 | 1 | 1 | 1 | 1 | 1 | 1 | 1 | 1 | 1 | 1 | 1 | 1 | 1 | 1 | 1 | 1 | 1 | 1 | 1 | 1 | 1 | 1 | 1 | 1 | 1 | 1 | 1 | 1 | 1 |
| 530 | 50S ribosomal protein L19 | 1 | 1 | 1 | 1 | 1 | 1 | 1 | 1 | 1 | 1 | 1 | 1 | 1 | 1 | 1 | 1 | 1 | 1 | 1 | 1 | 1 | 1 | 1 | 1 | 1 | 1 | 1 | 1 | 1 | 1 | 1 | 1 | 1 | 1 | 1 |
| 531 | tRNA (guanine-N(1)-)-methyltransferase | 1 | 1 | 1 | 1 | 1 | 1 | 1 | 1 | 1 | 1 | 1 | 1 | 1 | 1 | 1 | 1 | 1 | 1 | 1 | 1 | 1 | 1 | 1 | 1 | 1 | 1 | 1 | 1 | 1 | 1 | 1 | 1 | 1 | 1 | 1 |
| 532 | Ribosome maturation factor RimM | 1 | 1 | 1 | 1 | 1 | 1 | 1 | 1 | 1 | 1 | 1 | 1 | 1 | 1 | 1 | 1 | 1 | 1 | 1 | 1 | 1 | 1 | 1 | 1 | 1 | 1 | 1 | 1 | 1 | 1 | 1 | 1 | 1 | 1 | 1 |
| 533 | 30S ribosomal protein S16 | 1 | 1 | 1 | 1 | 1 | 1 | 1 | 1 | 1 | 1 | 1 | 1 | 1 | 1 | 1 | 1 | 1 | 1 | 1 | 1 | 1 | 1 | 1 | 1 | 1 | 1 | 1 | 1 | 1 | 1 | 1 | 1 | 1 | 1 | 1 |
| 534 | Signal recognition particle protein | 1 | 1 | 1 | 1 | 1 | 1 | 1 | 1 | 1 | 1 | 1 | 1 | 1 | 1 | 1 | 1 | 1 | 1 | 1 | 1 | 1 | 1 | 1 | 1 | 1 | 1 | 1 | 1 | 1 | 1 | 1 | 1 | 1 | 1 | 1 |
| 535 | Glutamate--cysteine ligase | 1 | 1 | 1 | 1 | 1 | 1 | 1 | 1 | 1 | 1 | 1 | 1 | 1 | 1 | 1 | 1 | 1 | 1 | 1 | 1 | 1 | 1 | 1 | 1 | 1 | 1 | 1 | 1 | 1 | 1 | 1 | 1 | 1 | 1 | 1 |
| 536 | Carbon storage regulator | 1 | 1 | 1 | 1 | 1 | 1 | 1 | 1 | 1 | 1 | 1 | 1 | 1 | 1 | 1 | 1 | 1 | 1 | 1 | 1 | 1 | 1 | 1 | 1 | 1 | 1 | 1 | 1 | 1 | 1 | 1 | 1 | 1 | 1 | 1 |
| 537 | Alanine--tRNA ligase | 1 | 1 | 1 | 1 | 1 | 1 | 1 | 1 | 1 | 1 | 1 | 1 | 1 | 1 | 1 | 1 | 1 | 1 | 1 | 1 | 1 | 1 | 1 | 1 | 1 | 1 | 1 | 1 | 1 | 1 | 1 | 1 | 1 | 1 | 1 |
| 538 | Murein hydrolase activator NlpD | 1 | 1 | 1 | 1 | 1 | 1 | 1 | 1 | 1 | 1 | 1 | 1 | 1 | 1 | 1 | 1 | 1 | 1 | 1 | 1 | 1 | 1 | 1 | 1 | 1 | 1 | 1 | 1 | 1 | 1 | 1 | 1 | 1 | 1 | 1 |
| 539 | 2-C-methyl-D-erythritol 2,4-cyclodiphosphate synthase | 1 | 1 | 1 | 1 | 1 | 1 | 1 | 1 | 1 | 1 | 1 | 1 | 1 | 1 | 1 | 1 | 1 | 1 | 1 | 1 | 1 | 1 | 1 | 1 | 1 | 1 | 1 | 1 | 1 | 1 | 1 | 1 | 1 | 1 | 1 |
| 540 | 2-C-methyl-D-erythritol 4-phosphate cytidylyltransferase | 1 | 1 | 1 | 1 | 1 | 1 | 1 | 1 | 1 | 1 | 1 | 1 | 1 | 1 | 1 | 1 | 1 | 1 | 1 | 1 | 1 | 1 | 1 | 1 | 1 | 1 | 1 | 1 | 1 | 1 | 1 | 1 | 1 | 1 | 1 |
| 541 | Cell division protein FtsB | 1 | 1 | 1 | 1 | 1 | 1 | 1 | 1 | 1 | 1 | 1 | 1 | 1 | 1 | 1 | 1 | 1 | 1 | 1 | 1 | 1 | 1 | 1 | 1 | 1 | 1 | 1 | 1 | 1 | 1 | 1 | 1 | 1 | 1 | 1 |
| 542 | Sulfate adenylyltransferase subunit 1 | 1 | 1 | 1 | 1 | 1 | 1 | 1 | 1 | 1 | 1 | 1 | 1 | 1 | 1 | 1 | 1 | 1 | 1 | 1 | 1 | 1 | 1 | 1 | 1 | 1 | 1 | 1 | 1 | 1 | 1 | 1 | 1 | 1 | 1 | 1 |
| 543 | Sulfite reductase [NADPH] flavoprotein alpha-component | 1 | 1 | 1 | 1 | 1 | 1 | 1 | 1 | 1 | 1 | 1 | 1 | 1 | 1 | 1 | 1 | 1 | 1 | 1 | 1 | 1 | 1 | 1 | 1 | 1 | 1 | 1 | 1 | 1 | 1 | 1 | 1 | 1 | 1 | 1 |
| 544 | Enolase | 1 | 1 | 1 | 1 | 1 | 1 | 1 | 1 | 1 | 1 | 1 | 1 | 1 | 1 | 1 | 1 | 1 | 1 | 1 | 1 | 1 | 1 | 1 | 1 | 1 | 1 | 1 | 1 | 1 | 1 | 1 | 1 | 1 | 1 | 1 |
| 545 | Iron-sulfur cluster insertion protein ErpA | 1 | 1 | 1 | 1 | 1 | 1 | 1 | 1 | 1 | 1 | 1 | 1 | 1 | 1 | 1 | 1 | 1 | 1 | 1 | 1 | 1 | 1 | 1 | 1 | 1 | 1 | 1 | 1 | 1 | 1 | 1 | 1 | 1 | 1 | 1 |
| 546 | Dephospho-CoA kinase | 1 | 1 | 1 | 1 | 1 | 1 | 1 | 1 | 1 | 1 | 1 | 1 | 1 | 1 | 1 | 1 | 1 | 1 | 1 | 1 | 1 | 1 | 1 | 1 | 1 | 1 | 1 | 1 | 1 | 1 | 1 | 1 | 1 | 1 | 1 |
| 547 | Protein translocase subunit SecA | 1 | 1 | 1 | 1 | 1 | 1 | 1 | 1 | 1 | 1 | 1 | 1 | 1 | 1 | 1 | 1 | 1 | 1 | 1 | 1 | 1 | 1 | 1 | 1 | 1 | 1 | 1 | 1 | 1 | 1 | 1 | 1 | 1 | 1 | 1 |
| 548 | hypothetical protein | 1 | 1 | 1 | 1 | 1 | 1 | 1 | 1 | 1 | 1 | 1 | 1 | 1 | 1 | 1 | 1 | 1 | 1 | 1 | 1 | 1 | 1 | 1 | 1 | 1 | 1 | 1 | 1 | 1 | 1 | 1 | 1 | 1 | 1 | 1 |
| 549 | UDP-3-O-[3-hydroxymyristoyl] N-acetylglucosamine deacetylase | 1 | 1 | 1 | 1 | 1 | 1 | 1 | 1 | 1 | 1 | 1 | 1 | 1 | 1 | 1 | 1 | 1 | 1 | 1 | 1 | 1 | 1 | 1 | 1 | 1 | 1 | 1 | 1 | 1 | 1 | 1 | 1 | 1 | 1 | 1 |
| 550 | Cell division protein FtsZ | 1 | 1 | 1 | 1 | 1 | 1 | 1 | 1 | 1 | 1 | 1 | 1 | 1 | 1 | 1 | 1 | 1 | 1 | 1 | 1 | 1 | 1 | 1 | 1 | 1 | 1 | 1 | 1 | 1 | 1 | 1 | 1 | 1 | 1 | 1 |
| 551 | Cell division protein FtsA | 1 | 1 | 1 | 1 | 1 | 1 | 1 | 1 | 1 | 1 | 1 | 1 | 1 | 1 | 1 | 1 | 1 | 1 | 1 | 1 | 1 | 1 | 1 | 1 | 1 | 1 | 1 | 1 | 1 | 1 | 1 | 1 | 1 | 1 | 1 |
| 552 | Cell division protein FtsQ | 1 | 1 | 1 | 1 | 1 | 1 | 1 | 1 | 1 | 1 | 1 | 1 | 1 | 1 | 1 | 1 | 1 | 1 | 1 | 1 | 1 | 1 | 1 | 1 | 1 | 1 | 1 | 1 | 1 | 1 | 1 | 1 | 1 | 1 | 1 |
| 553 | UDP-N-acetylmuramate--L-alanine ligase | 1 | 1 | 1 | 1 | 1 | 1 | 1 | 1 | 1 | 1 | 1 | 1 | 1 | 1 | 1 | 1 | 1 | 1 | 1 | 1 | 1 | 1 | 1 | 1 | 1 | 1 | 1 | 1 | 1 | 1 | 1 | 1 | 1 | 1 | 1 |
| 554 | UDP-N-acetylglucosamine--N-acetylmuramyl-(pentapeptide) pyrophosphoryl-undecaprenol N-acetylglucosamine transferase | 1 | 1 | 1 | 1 | 1 | 1 | 1 | 1 | 1 | 1 | 1 | 1 | 1 | 1 | 1 | 1 | 1 | 1 | 1 | 1 | 1 | 1 | 1 | 1 | 1 | 1 | 1 | 1 | 1 | 1 | 1 | 1 | 1 | 1 | 1 |
| 555 | Lipid II flippase FtsW | 1 | 1 | 1 | 1 | 1 | 1 | 1 | 1 | 1 | 1 | 1 | 1 | 1 | 1 | 1 | 1 | 1 | 1 | 1 | 1 | 1 | 1 | 1 | 1 | 1 | 1 | 1 | 1 | 1 | 1 | 1 | 1 | 1 | 1 | 1 |
| 556 | UDP-N-acetylmuramoylalanine--D-glutamate ligase | 1 | 1 | 1 | 1 | 1 | 1 | 1 | 1 | 1 | 1 | 1 | 1 | 1 | 1 | 1 | 1 | 1 | 1 | 1 | 1 | 1 | 1 | 1 | 1 | 1 | 1 | 1 | 1 | 1 | 1 | 1 | 1 | 1 | 1 | 1 |
| 557 | Phospho-N-acetylmuramoyl-pentapeptide-transferase | 1 | 1 | 1 | 1 | 1 | 1 | 1 | 1 | 1 | 1 | 1 | 1 | 1 | 1 | 1 | 1 | 1 | 1 | 1 | 1 | 1 | 1 | 1 | 1 | 1 | 1 | 1 | 1 | 1 | 1 | 1 | 1 | 1 | 1 | 1 |
| 558 | UDP-N-acetylmuramoyl-tripeptide--D-alanyl-D-alanine ligase | 1 | 1 | 1 | 1 | 1 | 1 | 1 | 1 | 1 | 1 | 1 | 1 | 1 | 1 | 1 | 1 | 1 | 1 | 1 | 1 | 1 | 1 | 1 | 1 | 1 | 1 | 1 | 1 | 1 | 1 | 1 | 1 | 1 | 1 | 1 |
| 559 | UDP-N-acetylmuramoyl-L-alanyl-D-glutamate--2,6-diaminopimelate ligase | 1 | 1 | 1 | 1 | 1 | 1 | 1 | 1 | 1 | 1 | 1 | 1 | 1 | 1 | 1 | 1 | 1 | 1 | 1 | 1 | 1 | 1 | 1 | 1 | 1 | 1 | 1 | 1 | 1 | 1 | 1 | 1 | 1 | 1 | 1 |
| 560 | Cell division protein FtsL | 1 | 1 | 1 | 1 | 1 | 1 | 1 | 1 | 1 | 1 | 1 | 1 | 1 | 1 | 1 | 1 | 1 | 1 | 1 | 1 | 1 | 1 | 1 | 1 | 1 | 1 | 1 | 1 | 1 | 1 | 1 | 1 | 1 | 1 | 1 |
| 561 | Ribosomal RNA small subunit methyltransferase H | 1 | 1 | 1 | 1 | 1 | 1 | 1 | 1 | 1 | 1 | 1 | 1 | 1 | 1 | 1 | 1 | 1 | 1 | 1 | 1 | 1 | 1 | 1 | 1 | 1 | 1 | 1 | 1 | 1 | 1 | 1 | 1 | 1 | 1 | 1 |
| 562 | LPS-assembly protein LptD | 1 | 1 | 1 | 1 | 1 | 1 | 1 | 1 | 1 | 1 | 1 | 1 | 1 | 1 | 1 | 1 | 1 | 1 | 1 | 1 | 1 | 1 | 1 | 1 | 1 | 1 | 1 | 1 | 1 | 1 | 1 | 1 | 1 | 1 | 1 |
| 563 | Chaperone SurA | 1 | 1 | 1 | 1 | 1 | 1 | 1 | 1 | 1 | 1 | 1 | 1 | 1 | 1 | 1 | 1 | 1 | 1 | 1 | 1 | 1 | 1 | 1 | 1 | 1 | 1 | 1 | 1 | 1 | 1 | 1 | 1 | 1 | 1 | 1 |
| 564 | Ribosomal RNA small subunit methyltransferase A | 1 | 1 | 1 | 1 | 1 | 1 | 1 | 1 | 1 | 1 | 1 | 1 | 1 | 1 | 1 | 1 | 1 | 1 | 1 | 1 | 1 | 1 | 1 | 1 | 1 | 1 | 1 | 1 | 1 | 1 | 1 | 1 | 1 | 1 | 1 |
| 565 | Protein ApaG | 1 | 1 | 1 | 1 | 1 | 1 | 1 | 1 | 1 | 1 | 1 | 1 | 1 | 1 | 1 | 1 | 1 | 1 | 1 | 1 | 1 | 1 | 1 | 1 | 1 | 1 | 1 | 1 | 1 | 1 | 1 | 1 | 1 | 1 | 1 |
| 566 | Bis(5-nucleosyl)-tetraphosphatase, symmetrical | 1 | 1 | 1 | 1 | 1 | 1 | 1 | 1 | 1 | 1 | 1 | 1 | 1 | 1 | 1 | 1 | 1 | 1 | 1 | 1 | 1 | 1 | 1 | 1 | 1 | 1 | 1 | 1 | 1 | 1 | 1 | 1 | 1 | 1 | 1 |
| 567 | Dihydrofolate reductase | 1 | 1 | 1 | 1 | 1 | 1 | 1 | 1 | 1 | 1 | 1 | 1 | 1 | 1 | 1 | 1 | 1 | 1 | 1 | 1 | 1 | 1 | 1 | 1 | 1 | 1 | 1 | 1 | 1 | 1 | 1 | 1 | 1 | 1 | 1 |
| 568 | 4-hydroxy-tetrahydrodipicolinate reductase | 1 | 1 | 1 | 1 | 1 | 1 | 1 | 1 | 1 | 1 | 1 | 1 | 1 | 1 | 1 | 1 | 1 | 1 | 1 | 1 | 1 | 1 | 1 | 1 | 1 | 1 | 1 | 1 | 1 | 1 | 1 | 1 | 1 | 1 | 1 |
| 569 | 4-hydroxy-3-methylbut-2-enyl diphosphate reductase | 1 | 1 | 1 | 1 | 1 | 1 | 1 | 1 | 1 | 1 | 1 | 1 | 1 | 1 | 1 | 1 | 1 | 1 | 1 | 1 | 1 | 1 | 1 | 1 | 1 | 1 | 1 | 1 | 1 | 1 | 1 | 1 | 1 | 1 | 1 |
| 570 | Lipoprotein signal peptidase | 1 | 1 | 1 | 1 | 1 | 1 | 1 | 1 | 1 | 1 | 1 | 1 | 1 | 1 | 1 | 1 | 1 | 1 | 1 | 1 | 1 | 1 | 1 | 1 | 1 | 1 | 1 | 1 | 1 | 1 | 1 | 1 | 1 | 1 | 1 |
| 571 | Isoleucine--tRNA ligase | 1 | 1 | 1 | 1 | 1 | 1 | 1 | 1 | 1 | 1 | 1 | 1 | 1 | 1 | 1 | 1 | 1 | 1 | 1 | 1 | 1 | 1 | 1 | 1 | 1 | 1 | 1 | 1 | 1 | 1 | 1 | 1 | 1 | 1 | 1 |
| 572 | Riboflavin biosynthesis protein RibF | 1 | 1 | 1 | 1 | 1 | 1 | 1 | 1 | 1 | 1 | 1 | 1 | 1 | 1 | 1 | 1 | 1 | 1 | 1 | 1 | 1 | 1 | 1 | 1 | 1 | 1 | 1 | 1 | 1 | 1 | 1 | 1 | 1 | 1 | 1 |
| 573 | Chaperone protein DnaJ | 1 | 1 | 1 | 1 | 1 | 1 | 1 | 1 | 1 | 1 | 1 | 1 | 1 | 1 | 1 | 1 | 1 | 1 | 1 | 1 | 1 | 1 | 1 | 1 | 1 | 1 | 1 | 1 | 1 | 1 | 1 | 1 | 1 | 1 | 1 |
| 574 | Chaperone protein DnaK | 1 | 1 | 1 | 1 | 1 | 1 | 1 | 1 | 1 | 1 | 1 | 1 | 1 | 1 | 1 | 1 | 1 | 1 | 1 | 1 | 1 | 1 | 1 | 1 | 1 | 1 | 1 | 1 | 1 | 1 | 1 | 1 | 1 | 1 | 1 |
| 576 | Purine nucleoside phosphorylase DeoD-type | 1 | 1 | 1 | 1 | 1 | 1 | 1 | 1 | 1 | 1 | 1 | 1 | 1 | 1 | 1 | 1 | 1 | 1 | 1 | 1 | 1 | 1 | 1 | 1 | 1 | 1 | 1 | 1 | 1 | 1 | 1 | 1 | 1 | 1 | 1 |
| 577 | Phosphopentomutase | 1 | 1 | 1 | 1 | 1 | 1 | 1 | 1 | 1 | 1 | 1 | 1 | 1 | 1 | 1 | 1 | 1 | 1 | 1 | 1 | 1 | 1 | 1 | 1 | 1 | 1 | 1 | 1 | 1 | 1 | 1 | 1 | 1 | 1 | 1 |
| 578 | Peptide chain release factor 3 | 1 | 1 | 1 | 1 | 1 | 1 | 1 | 1 | 1 | 1 | 1 | 1 | 1 | 1 | 1 | 1 | 1 | 1 | 1 | 1 | 1 | 1 | 1 | 1 | 1 | 1 | 1 | 1 | 1 | 1 | 1 | 1 | 1 | 1 | 1 |
| 579 | DNA polymerase III subunit psi | 1 | 1 | 1 | 1 | 1 | 1 | 1 | 1 | 1 | 1 | 1 | 1 | 1 | 1 | 1 | 1 | 1 | 1 | 1 | 1 | 1 | 1 | 1 | 1 | 1 | 1 | 1 | 1 | 1 | 1 | 1 | 1 | 1 | 1 | 1 |
| 584 | Cold shock-like protein CspC | 1 | 1 | 1 | 1 | 1 | 1 | 1 | 1 | 1 | 1 | 1 | 1 | 1 | 1 | 1 | 1 | 1 | 1 | 1 | 1 | 1 | 1 | 1 | 1 | 1 | 1 | 1 | 1 | 1 | 1 | 1 | 1 | 1 | 1 | 1 |
| 585 | Putative lipid II flippase MurJ | 1 | 1 | 1 | 1 | 1 | 1 | 1 | 1 | 1 | 1 | 1 | 1 | 1 | 1 | 1 | 1 | 1 | 1 | 1 | 1 | 1 | 1 | 1 | 1 | 1 | 1 | 1 | 1 | 1 | 1 | 1 | 1 | 1 | 1 | 1 |
| 586 | Arginine--tRNA ligase | 1 | 1 | 1 | 1 | 1 | 1 | 1 | 1 | 1 | 1 | 1 | 1 | 1 | 1 | 1 | 1 | 1 | 1 | 1 | 1 | 1 | 1 | 1 | 1 | 1 | 1 | 1 | 1 | 1 | 1 | 1 | 1 | 1 | 1 | 1 |
| 587 | Aspartate--tRNA ligase | 1 | 1 | 1 | 1 | 1 | 1 | 1 | 1 | 1 | 1 | 1 | 1 | 1 | 1 | 1 | 1 | 1 | 1 | 1 | 1 | 1 | 1 | 1 | 1 | 1 | 1 | 1 | 1 | 1 | 1 | 1 | 1 | 1 | 1 | 1 |
| 588 | Murein DD-endopeptidase MepM | 1 | 1 | 1 | 1 | 1 | 1 | 1 | 1 | 1 | 1 | 1 | 1 | 1 | 1 | 1 | 1 | 1 | 1 | 1 | 1 | 1 | 1 | 1 | 1 | 1 | 1 | 1 | 1 | 1 | 1 | 1 | 1 | 1 | 1 | 1 |
| 589 | Glucose-6-phosphate 1-dehydrogenase | 1 | 1 | 1 | 1 | 1 | 1 | 1 | 1 | 1 | 1 | 1 | 1 | 1 | 1 | 1 | 1 | 1 | 1 | 1 | 1 | 1 | 1 | 1 | 1 | 1 | 1 | 1 | 1 | 1 | 1 | 1 | 1 | 1 | 1 | 1 |
| 591 | tRNA threonylcarbamoyladenosine biosynthesis protein TsaB | 1 | 1 | 1 | 1 | 1 | 1 | 1 | 1 | 1 | 1 | 1 | 1 | 1 | 1 | 1 | 1 | 1 | 1 | 1 | 1 | 1 | 1 | 1 | 1 | 1 | 1 | 1 | 1 | 1 | 1 | 1 | 1 | 1 | 1 | 1 |
| 592 | Septum site-determining protein MinD | 1 | 1 | 1 | 1 | 1 | 1 | 1 | 1 | 1 | 1 | 1 | 1 | 1 | 1 | 1 | 1 | 1 | 1 | 1 | 1 | 1 | 1 | 1 | 1 | 1 | 1 | 1 | 1 | 1 | 1 | 1 | 1 | 1 | 1 | 1 |
| 593 | Septum site-determining protein MinC | 1 | 1 | 1 | 1 | 1 | 1 | 1 | 1 | 1 | 1 | 1 | 1 | 1 | 1 | 1 | 1 | 1 | 1 | 1 | 1 | 1 | 1 | 1 | 1 | 1 | 1 | 1 | 1 | 1 | 1 | 1 | 1 | 1 | 1 | 1 |
| 594 | Glyceraldehyde-3-phosphate dehydrogenase A | 1 | 1 | 1 | 1 | 1 | 1 | 1 | 1 | 1 | 1 | 1 | 1 | 1 | 1 | 1 | 1 | 1 | 1 | 1 | 1 | 1 | 1 | 1 | 1 | 1 | 1 | 1 | 1 | 1 | 1 | 1 | 1 | 1 | 1 | 1 |
| 595 | DNA-binding protein H-NS | 1 | 1 | 1 | 1 | 1 | 1 | 1 | 1 | 1 | 1 | 1 | 1 | 1 | 1 | 1 | 1 | 1 | 1 | 1 | 1 | 1 | 1 | 1 | 1 | 1 | 1 | 1 | 1 | 1 | 1 | 1 | 1 | 1 | 1 | 1 |

p = plasmid

**Supplementary Table 4.** *Serratia* liquefaciens and *Serratia* proteamaculnas core genome family proteins.

| **Family** | **Description** | **Number o genes** | | | | |
| --- | --- | --- | --- | --- | --- | --- |
|  |  | **S_ufop_01_chr** | **S_liquefaciens_HUMV21_chr** | **S_liquefaciens_ATCC27592_chr** | **S_liquefaciens_FDAARGOS125_chr** | **S_proteamaculans_568_chr** |
| 4096 | hypothetical protein | 1 | 1 | 1 | 1 | 1 |
| 4097 | HTH-type transcriptional regulator NimR | 1 | 1 | 1 | 1 | 1 |
| 4098 | hypothetical protein | 1 | 1 | 1 | 1 | 1 |
| 4099 | hypothetical protein | 1 | 1 | 1 | 1 | 1 |
| 4100 | hypothetical protein | 1 | 1 | 1 | 1 | 1 |
| 4103 | hypothetical protein | 1 | 1 | 1 | 1 | 1 |
| 4104 | Iron import ATP-binding/permease protein IrtB | 1 | 1 | 1 | 1 | 1 |
| 4105 | Iron import ATP-binding/permease protein IrtA | 1 | 1 | 1 | 1 | 1 |
| 4106 | Mannonate dehydratase | 1 | 1 | 1 | 1 | 1 |
| 4108 | hypothetical protein | 1 | 1 | 1 | 1 | 1 |
| 4109 | 1H-3-hydroxy-4-oxoquinoline 2,4-dioxygenase | 1 | 1 | 1 | 1 | 1 |
| 4110 | Melamine deaminase | 1 | 1 | 1 | 1 | 1 |
| 4111 | Phthiocerol synthesis polyketide synthase type I PpsC | 1 | 1 | 1 | 1 | 1 |
| 4113 | Shikimate 5-dehydrogenase-like protein | 1 | 1 | 1 | 1 | 1 |
| 4114 | RTX-I toxin-activating lysine-acyltransferase ApxIC | 1 | 1 | 1 | 1 | 1 |
| 4116 | Fe(3+) dicitrate-binding periplasmic protein | 1 | 1 | 1 | 1 | 1 |
| 4118 | hypothetical protein | 1 | 1 | 1 | 1 | 1 |
| 4119 | Toxin HokB | 1 | 1 | 1 | 1 | 1 |
| 4120 | D-malate dehydrogenase [decarboxylating] | 1 | 1 | 1 | 1 | 1 |
| 4121 | HTH-type transcriptional regulator DmlR | 1 | 1 | 1 | 1 | 1 |
| 4122 | hypothetical protein | 1 | 1 | 1 | 1 | 1 |
| 4123 | hypothetical protein | 1 | 1 | 1 | 1 | 1 |
| 4124 | hypothetical protein | 1 | 1 | 1 | 1 | 1 |
| 4125 | hypothetical protein | 1 | 1 | 1 | 1 | 1 |
| 4131 | Positive transcription regulator EvgA | 1 | 1 | 1 | 1 | 1 |
| 4132 | hypothetical protein | 1 | 1 | 1 | 1 | 1 |
| 4133 | Antitoxin DinJ | 1 | 1 | 1 | 1 | 1 |
| 4134 | HTH-type transcriptional regulator PgrR | 1 | 1 | 1 | 1 | 1 |
| 4135 | hypothetical protein | 1 | 1 | 1 | 1 | 1 |
| 4137 | 4-hydroxybenzoate transporter PcaK | 1 | 1 | 1 | 1 | 1 |
| 4138 | hypothetical protein | 1 | 1 | 1 | 1 | 1 |
| 4139 | hypothetical protein | 1 | 1 | 1 | 1 | 1 |
| 4140 | MltA-interacting protein | 1 | 1 | 1 | 1 | 1 |
| 4141 | 4-hydroxybenzoate transporter PcaK | 1 | 1 | 1 | 1 | 1 |
| 4142 | 6-hydroxynicotinate 3-monooxygenase | 1 | 1 | 1 | 1 | 1 |
| 4143 | Carboxylesterase NlhH | 1 | 1 | 1 | 1 | 1 |
| 4144 | hypothetical protein | 1 | 1 | 1 | 1 | 1 |
| 4145 | Toxin ParE1 | 1 | 1 | 1 | 1 | 1 |
| 4146 | Multifunctional cyclase-dehydratase-3-O-methyl transferase TcmN | 1 | 1 | 1 | 1 | 1 |
| 4147 | putative diguanylate cyclase YegE | 1 | 1 | 1 | 1 | 1 |
| 4148 | hypothetical protein | 1 | 1 | 1 | 1 | 1 |
| 4149 | Molybdenum-pterin-binding protein MopA | 1 | 1 | 1 | 1 | 1 |
| 4150 | hypothetical protein | 1 | 1 | 1 | 1 | 1 |
| 4151 | Ferrichrome receptor FcuA | 1 | 1 | 1 | 1 | 1 |
| 4152 | Macrolide export protein MacA | 1 | 1 | 1 | 1 | 1 |
| 4153 | hypothetical protein | 1 | 1 | 1 | 1 | 1 |
| 4154 | putative formate transporter 1 | 1 | 1 | 1 | 1 | 1 |
| 4155 | hypothetical protein | 1 | 1 | 1 | 1 | 1 |
| 4156 | hypothetical protein | 1 | 1 | 1 | 1 | 1 |
| 4157 | hypothetical protein | 1 | 1 | 1 | 1 | 1 |
| 4158 | hypothetical protein | 1 | 1 | 1 | 1 | 1 |
| 63 | Fe(3+) dicitrate transport protein FecA | 2 | 2 | 2 | 2 | 2 |
| 4160 | HTH-type transcriptional regulatory protein GabR | 1 | 1 | 1 | 1 | 1 |
| 4161 | hypothetical protein | 1 | 1 | 1 | 1 | 1 |
| 4163 | hypothetical protein | 1 | 1 | 1 | 1 | 1 |
| 4164 | Altronate dehydratase | 1 | 1 | 1 | 1 | 1 |
| 4165 | Altronate dehydratase | 1 | 1 | 1 | 1 | 1 |
| 4166 | 2-dehydro-3-deoxy-D-gluconate 5-dehydrogenase | 1 | 1 | 1 | 1 | 1 |
| 4167 | putative zinc-type alcohol dehydrogenase-like protein YjmD | 1 | 1 | 1 | 1 | 1 |
| 4168 | Hexuronate transporter | 1 | 1 | 1 | 1 | 1 |
| 4169 | HTH-type transcriptional regulator LutR | 1 | 1 | 1 | 1 | 1 |
| 4170 | hypothetical protein | 1 | 1 | 1 | 1 | 1 |
| 4171 | hypothetical protein | 1 | 1 | 1 | 1 | 1 |
| 4172 | hypothetical protein | 1 | 1 | 1 | 1 | 1 |
| 4173 | putative ABC transporter solute-binding protein YclQ | 1 | 1 | 1 | 1 | 1 |
| 4174 | putative isomerase YddE | 1 | 1 | 1 | 1 | 1 |
| 4175 | HTH-type transcriptional regulator SutR | 1 | 1 | 1 | 1 | 1 |
| 4176 | L-methionine sulfoximine/L-methionine sulfone acetyltransferase | 1 | 1 | 1 | 1 | 1 |
| 4177 | Inner membrane protein YdcZ | 1 | 1 | 1 | 1 | 1 |
| 4178 | HTH-type transcriptional regulator DmlR | 1 | 1 | 1 | 1 | 1 |
| 4179 | Sensor protein CzcS | 1 | 1 | 1 | 1 | 1 |
| 4180 | Transcriptional activator protein CopR | 1 | 1 | 1 | 1 | 1 |
| 4181 | Multidrug resistance protein MexA | 1 | 1 | 1 | 1 | 1 |
| 4182 | Outer membrane protein OprM | 1 | 1 | 1 | 1 | 1 |
| 4183 | Membrane-bound lytic murein transglycosylase F | 1 | 1 | 1 | 1 | 1 |
| 3673 | Prophage CP4-57 integrase | 1 | 2 | 1 | 1 | 1 |
| 3162 | HTH-type transcriptional regulator DmlR | 1 | 1 | 1 | 2 | 2 |
| 4187 | hypothetical protein | 1 | 1 | 1 | 1 | 1 |
| 4188 | Major phosphate-irrepressible acid phosphatase | 1 | 1 | 1 | 1 | 1 |
| 4189 | hypothetical protein | 1 | 1 | 1 | 1 | 1 |
| 4190 | hypothetical protein | 1 | 1 | 1 | 1 | 1 |
| 2143 | Alcohol dehydrogenase [acceptor] | 2 | 1 | 1 | 1 | 1 |
| 4192 | Lipopolysaccharide export system permease protein LptF | 1 | 1 | 1 | 1 | 1 |
| 97 | Putative prophage CPS-53 integrase | 1 | 2 | 1 | 2 | 2 |
| 4194 | hypothetical protein | 1 | 1 | 1 | 1 | 1 |
| 4195 | Inner membrane protein YghQ | 1 | 1 | 1 | 1 | 1 |
| 4196 | Thymidylate kinase | 1 | 1 | 1 | 1 | 1 |
| 4197 | Thymidylate kinase | 1 | 1 | 1 | 1 | 1 |
| 102 | hypothetical protein | 2 | 1 | 2 | 1 | 1 |
| 4199 | 8-amino-7-oxononanoate synthase | 1 | 1 | 1 | 1 | 1 |
| 4200 | Acyl carrier protein | 1 | 1 | 1 | 1 | 1 |
| 4201 | 3 beta-hydroxysteroid dehydrogenase/Delta 5-->4-isomerase | 1 | 1 | 1 | 1 | 1 |
| 4202 | Long-chain-fatty-acid--AMP ligase FadD29 | 1 | 1 | 1 | 1 | 1 |
| 4203 | hypothetical protein | 1 | 1 | 1 | 1 | 1 |
| 4204 | hypothetical protein | 1 | 1 | 1 | 1 | 1 |
| 4205 | Diacylglycerol kinase | 1 | 1 | 1 | 1 | 1 |
| 4206 | General stress protein 69 | 1 | 1 | 1 | 1 | 1 |
| 4207 | hypothetical protein | 1 | 1 | 1 | 1 | 1 |
| 4208 | HTH-type transcriptional regulator DmlR | 1 | 1 | 1 | 1 | 1 |
| 4209 | hypothetical protein | 1 | 1 | 1 | 1 | 1 |
| 4212 | hypothetical protein | 1 | 1 | 1 | 1 | 1 |
| 4213 | Cation transport regulator ChaB | 1 | 1 | 1 | 1 | 1 |
| 4214 | hypothetical protein | 1 | 1 | 1 | 1 | 1 |
| 4216 | hypothetical protein | 1 | 1 | 1 | 1 | 1 |
| 4217 | hypothetical protein | 1 | 1 | 1 | 1 | 1 |
| 4218 | hypothetical protein | 1 | 1 | 1 | 1 | 1 |
| 4219 | hypothetical protein | 1 | 1 | 1 | 1 | 1 |
| 4220 | E3 ubiquitin-protein ligase sspH2 | 1 | 1 | 1 | 1 | 1 |
| 4198 | Thymidylate kinase | 1 | 1 | 1 | 1 | 1 |
| 4222 | hypothetical protein | 1 | 1 | 1 | 1 | 1 |
| 4223 | hypothetical protein | 1 | 1 | 1 | 1 | 1 |
| 4224 | hypothetical protein | 1 | 1 | 1 | 1 | 1 |
| 4225 | hypothetical protein | 1 | 1 | 1 | 1 | 1 |
| 4226 | Lactoylglutathione lyase | 1 | 1 | 1 | 1 | 1 |
| 4227 | HTH-type transcriptional regulator GltR | 1 | 1 | 1 | 1 | 1 |
| 4228 | hypothetical protein | 1 | 1 | 1 | 1 | 1 |
| 4229 | Multiple stress resistance protein BhsA | 1 | 1 | 1 | 1 | 1 |
| 4230 | hypothetical protein | 1 | 1 | 1 | 1 | 1 |
| 4231 | HTH-type transcriptional regulator HmrR | 1 | 1 | 1 | 1 | 1 |
| 4232 | hypothetical protein | 1 | 1 | 1 | 1 | 1 |
| 4233 | Toluene efflux pump outer membrane protein TtgI | 1 | 1 | 1 | 1 | 1 |
| 4234 | Efflux pump periplasmic linker BepF | 1 | 1 | 1 | 1 | 1 |
| 4235 | hypothetical protein | 1 | 1 | 1 | 1 | 1 |
| 4236 | Periplasmic oligopeptide-binding protein | 1 | 1 | 1 | 1 | 1 |
| 4237 | hypothetical protein | 1 | 1 | 1 | 1 | 1 |
| 4238 | hypothetical protein | 1 | 1 | 1 | 1 | 1 |
| 4239 | PTS system glucose-specific EIICB component | 1 | 1 | 1 | 1 | 1 |
| 4240 | L-asparaginase | 1 | 1 | 1 | 1 | 1 |
| 4241 | NADH oxidase | 1 | 1 | 1 | 1 | 1 |
| 4242 | hypothetical protein | 1 | 1 | 1 | 1 | 1 |
| 4243 | Non-heme chloroperoxidase | 1 | 1 | 1 | 1 | 1 |
| 4245 | hypothetical protein | 1 | 1 | 1 | 1 | 1 |
| 4246 | Molecular chaperone Hsp31 and glyoxalase 3 | 1 | 1 | 1 | 1 | 1 |
| 4247 | Manganese-dependent 2,3-dihydroxybiphenyl 1,2-dioxygenase | 1 | 1 | 1 | 1 | 1 |
| 4248 | Phenylacetaldehyde dehydrogenase | 1 | 1 | 1 | 1 | 1 |
| 4249 | Ureidoglycolate lyase | 1 | 1 | 1 | 1 | 1 |
| 4250 | HTH-type transcriptional repressor RspR | 1 | 1 | 1 | 1 | 1 |
| 4251 | Bacilysin biosynthesis oxidoreductase YwfH | 1 | 1 | 1 | 1 | 1 |
| 4252 | Peptidoglycan-N-acetylglucosamine deacetylase | 1 | 1 | 1 | 1 | 1 |
| 4253 | 3-(3-hydroxy-phenyl)propionate/3-hydroxycinnamic acid hydroxylase | 1 | 1 | 1 | 1 | 1 |
| 4254 | hypothetical protein | 1 | 1 | 1 | 1 | 1 |
| 4255 | HTH-type transcriptional regulator DmlR | 1 | 1 | 1 | 1 | 1 |
| 4256 | Aldehyde dehydrogenase PuuC | 1 | 1 | 1 | 1 | 1 |
| 4257 | 3-oxoacyl-[acyl-carrier-protein] reductase FabG | 1 | 1 | 1 | 1 | 1 |
| 4258 | hypothetical protein | 1 | 1 | 1 | 1 | 1 |
| 4259 | 2-haloacrylate reductase | 1 | 1 | 1 | 1 | 1 |
| 4260 | HTH-type transcriptional regulator DmlR | 1 | 1 | 1 | 1 | 1 |
| 4261 | HTH-type transcriptional regulator DmlR | 1 | 1 | 1 | 1 | 1 |
| 4262 | putative transporter | 1 | 1 | 1 | 1 | 1 |
| 4263 | Hemoglobin and hemoglobin-haptoglobin-binding protein | 1 | 1 | 1 | 1 | 1 |
| 4265 | hypothetical protein | 1 | 1 | 1 | 1 | 1 |
| 4266 | hypothetical protein | 1 | 1 | 1 | 1 | 1 |
| 4267 | hypothetical protein | 1 | 1 | 1 | 1 | 1 |
| 4268 | 4-formylbenzenesulfonate dehydrogenase TsaC1/TsaC2 | 1 | 1 | 1 | 1 | 1 |
| 4269 | hypothetical protein | 1 | 1 | 1 | 1 | 1 |
| 4270 | Serralysin C | 1 | 1 | 1 | 1 | 1 |
| 4271 | hypothetical protein | 1 | 1 | 1 | 1 | 1 |
| 4272 | HTH-type transcriptional regulator CysL | 1 | 1 | 1 | 1 | 1 |
| 4273 | putative RNA polymerase sigma factor FecI | 1 | 1 | 1 | 1 | 1 |
| 4274 | hypothetical protein | 1 | 1 | 1 | 1 | 1 |
| 4275 | Phenazine antibiotic resistance protein EhpR | 1 | 1 | 1 | 1 | 1 |
| 4276 | Bifunctional ligase/repressor BirA | 1 | 1 | 1 | 1 | 1 |
| 4277 | HTH-type transcriptional repressor BdcR | 1 | 1 | 1 | 1 | 1 |
| 4278 | Inner membrane transport protein YdhP | 1 | 1 | 1 | 1 | 1 |
| 4279 | hypothetical protein | 1 | 1 | 1 | 1 | 1 |
| 4280 | hypothetical protein | 1 | 1 | 1 | 1 | 1 |
| 4281 | hypothetical protein | 1 | 1 | 1 | 1 | 1 |
| 4282 | hypothetical protein | 1 | 1 | 1 | 1 | 1 |
| 4286 | hypothetical protein | 1 | 1 | 1 | 1 | 1 |
| 4287 | Phosphonoacetaldehyde hydrolase | 1 | 1 | 1 | 1 | 1 |
| 4288 | 2-aminoethylphosphonate--pyruvate transaminase | 1 | 1 | 1 | 1 | 1 |
| 4289 | Putative transcriptional regulator of 2-aminoethylphosphonate degradation operons | 1 | 1 | 1 | 1 | 1 |
| 4290 | hypothetical protein | 1 | 1 | 1 | 1 | 1 |
| 4291 | Phloretin hydrolase | 1 | 1 | 1 | 1 | 1 |
| 4292 | hypothetical protein | 1 | 1 | 1 | 1 | 1 |
| 4293 | Universal stress protein A | 1 | 1 | 1 | 1 | 1 |
| 4294 | hypothetical protein | 1 | 1 | 1 | 1 | 1 |
| 4295 | hypothetical protein | 1 | 1 | 1 | 1 | 1 |
| 4296 | hypothetical protein | 1 | 1 | 1 | 1 | 1 |
| 4297 | hypothetical protein | 1 | 1 | 1 | 1 | 1 |
| 4298 | hypothetical protein | 1 | 1 | 1 | 1 | 1 |
| 4299 | Dihydrofolate reductase | 1 | 1 | 1 | 1 | 1 |
| 4300 | Tetracycline repressor protein class H | 1 | 1 | 1 | 1 | 1 |
| 4301 | Nucleoside permease NupX | 1 | 1 | 1 | 1 | 1 |
| 4302 | Pseudouridine kinase | 1 | 1 | 1 | 1 | 1 |
| 4303 | hypothetical protein | 1 | 1 | 1 | 1 | 1 |
| 4304 | hypothetical protein | 1 | 1 | 1 | 1 | 1 |
| 4305 | (R)-stereoselective amidase | 1 | 1 | 1 | 1 | 1 |
| 4306 | Transcriptional regulatory protein TdiR | 1 | 1 | 1 | 1 | 1 |
| 4307 | hypothetical protein | 1 | 1 | 1 | 1 | 1 |
| 4308 | Sialic acid TRAP transporter permease protein SiaT | 1 | 1 | 1 | 1 | 1 |
| 4309 | HTH-type transcriptional regulator LutR | 1 | 1 | 1 | 1 | 1 |
| 4310 | L-2-hydroxyglutarate oxidase LhgO | 1 | 1 | 1 | 1 | 1 |
| 4311 | hypothetical protein | 1 | 1 | 1 | 1 | 1 |
| 4312 | hypothetical protein | 1 | 1 | 1 | 1 | 1 |
| 4313 | HTH-type transcriptional regulator DmlR | 1 | 1 | 1 | 1 | 1 |
| 4314 | hypothetical protein | 1 | 1 | 1 | 1 | 1 |
| 4315 | hypothetical protein | 1 | 1 | 1 | 1 | 1 |
| 4316 | hypothetical protein | 1 | 1 | 1 | 1 | 1 |
| 4317 | NADPH dehydrogenase | 1 | 1 | 1 | 1 | 1 |
| 4318 | hypothetical protein | 1 | 1 | 1 | 1 | 1 |
| 4321 | hypothetical protein | 1 | 1 | 1 | 1 | 1 |
| 4322 | hypothetical protein | 1 | 1 | 1 | 1 | 1 |
| 4323 | hypothetical protein | 1 | 1 | 1 | 1 | 1 |
| 4324 | Inner membrane protein YdgC | 1 | 1 | 1 | 1 | 1 |
| 4325 | hypothetical protein | 1 | 1 | 1 | 1 | 1 |
| 4326 | hypothetical protein | 1 | 1 | 1 | 1 | 1 |
| 4327 | Inner membrane transport protein RhmT | 1 | 1 | 1 | 1 | 1 |
| 4329 | hypothetical protein | 1 | 1 | 1 | 1 | 1 |
| 4330 | Excinuclease cho | 1 | 1 | 1 | 1 | 1 |
| 4331 | hypothetical protein | 1 | 1 | 1 | 1 | 1 |
| 4332 | Soluble aldose sugar dehydrogenase YliI | 1 | 1 | 1 | 1 | 1 |
| 4221 | putative protease YdeA | 1 | 1 | 1 | 1 | 1 |
| 4336 | hypothetical protein | 1 | 1 | 1 | 1 | 1 |
| 4337 | Inner membrane protein RclC | 1 | 1 | 1 | 1 | 1 |
| 4338 | Shikimate kinase | 1 | 1 | 1 | 1 | 1 |
| 4342 | Multiple stress resistance protein BhsA | 1 | 1 | 1 | 1 | 1 |
| 4343 | Leucine efflux protein | 1 | 1 | 1 | 1 | 1 |
| 4344 | HTH-type transcriptional regulator DmlR | 1 | 1 | 1 | 1 | 1 |
| 4345 | Multidrug resistance protein Stp | 1 | 1 | 1 | 1 | 1 |
| 4346 | hypothetical protein | 1 | 1 | 1 | 1 | 1 |
| 4347 | putative HTH-type transcriptional regulator YahB | 1 | 1 | 1 | 1 | 1 |
| 4348 | hypothetical protein | 1 | 1 | 1 | 1 | 1 |
| 4349 | hypothetical protein | 1 | 1 | 1 | 1 | 1 |
| 4350 | N-substituted formamide deformylase | 1 | 1 | 1 | 1 | 1 |
| 767 | Outer membrane protein X | 2 | 1 | 1 | 1 | 1 |
| 4352 | Serine 3-dehydrogenase | 1 | 1 | 1 | 1 | 1 |
| 4353 | Non-hemolytic phospholipase C | 1 | 1 | 1 | 1 | 1 |
| 4354 | Antitoxin ParD1 | 1 | 1 | 1 | 1 | 1 |
| 4355 | hypothetical protein | 1 | 1 | 1 | 1 | 1 |
| 4356 | Transcriptional regulatory protein ZraR | 1 | 1 | 1 | 1 | 1 |
| 4357 | Methanesulfonate monooxygenase | 1 | 1 | 1 | 1 | 1 |
| 4358 | Alkanesulfonate monooxygenase | 1 | 1 | 1 | 1 | 1 |
| 4359 | tRNA 5-methylaminomethyl-2-thiouridine biosynthesis bifunctional protein MnmC | 1 | 1 | 1 | 1 | 1 |
| 4360 | hypothetical protein | 1 | 1 | 1 | 1 | 1 |
| 4361 | 2-(hydroxymethyl)glutarate dehydrogenase | 1 | 1 | 1 | 1 | 1 |
| 4362 | hypothetical protein | 1 | 1 | 1 | 1 | 1 |
| 4364 | Aerotaxis receptor | 1 | 1 | 1 | 1 | 1 |
| 4365 | Sulfite reductase [NADPH] flavoprotein alpha-component | 1 | 1 | 1 | 1 | 1 |
| 4366 | putative membrane protein YohP | 1 | 1 | 1 | 1 | 1 |
| 4367 | Response regulator protein TmoT | 1 | 1 | 1 | 1 | 1 |
| 4368 | Sensor histidine kinase TodS | 1 | 1 | 1 | 1 | 1 |
| 4369 | hypothetical protein | 1 | 1 | 1 | 1 | 1 |
| 4370 | HTH-type transcriptional regulator CdhR | 1 | 1 | 1 | 1 | 1 |
| 4371 | Threonine efflux protein | 1 | 1 | 1 | 1 | 1 |
| 4186 | HTH-type transcriptional regulator VirS | 1 | 1 | 1 | 1 | 1 |
| 4373 | L-cystine-binding protein FliY | 1 | 1 | 1 | 1 | 1 |
| 4374 | 4-hydroxybenzoate transporter PcaK | 1 | 1 | 1 | 1 | 1 |
| 4375 | 2-aminomuconic 6-semialdehyde dehydrogenase | 1 | 1 | 1 | 1 | 1 |
| 4376 | 2-oxopent-4-enoate hydratase | 1 | 1 | 1 | 1 | 1 |
| 4377 | Acetaldehyde dehydrogenase | 1 | 1 | 1 | 1 | 1 |
| 4378 | 4-hydroxy-2-oxovalerate aldolase | 1 | 1 | 1 | 1 | 1 |
| 4379 | 4-oxalocrotonate decarboxylase | 1 | 1 | 1 | 1 | 1 |
| 4380 | 2-aminomuconate deaminase | 1 | 1 | 1 | 1 | 1 |
| 4381 | 3-hydroxyanthranilate 3,4-dioxygenase | 1 | 1 | 1 | 1 | 1 |
| 4382 | hypothetical protein | 1 | 1 | 1 | 1 | 1 |
| 4383 | hypothetical protein | 1 | 1 | 1 | 1 | 1 |
| 4384 | Quinone oxidoreductase 1 | 1 | 1 | 1 | 1 | 1 |
| 4385 | 3-oxoacyl-[acyl-carrier-protein] reductase FabG | 1 | 1 | 1 | 1 | 1 |
| 4386 | Inner membrane protein YqjF | 1 | 1 | 1 | 1 | 1 |
| 4387 | hypothetical protein | 1 | 1 | 1 | 1 | 1 |
| 4388 | Putative peroxiredoxin bcp | 1 | 1 | 1 | 1 | 1 |
| 4389 | Phosphatidylserine decarboxylase proenzyme | 1 | 1 | 1 | 1 | 1 |
| 4390 | hypothetical protein | 1 | 1 | 1 | 1 | 1 |
| 4391 | Transcription antiterminator LicT | 1 | 1 | 1 | 1 | 1 |
| 4392 | General stress protein 69 | 1 | 1 | 1 | 1 | 1 |
| 4393 | hypothetical protein | 1 | 1 | 1 | 1 | 1 |
| 4394 | hypothetical protein | 1 | 1 | 1 | 1 | 1 |
| 4395 | Multiple stress resistance protein BhsA | 1 | 1 | 1 | 1 | 1 |
| 4397 | Uric acid permease PucK | 1 | 1 | 1 | 1 | 1 |
| 4399 | 23S rRNA (uracil(1939)-C(5))-methyltransferase RlmD | 1 | 1 | 1 | 1 | 1 |
| 4400 | 1,6-anhydro-N-acetylmuramyl-L-alanine amidase AmpD | 1 | 1 | 1 | 1 | 1 |
| 4401 | putative 2-dehydro-3-deoxygalactonokinase DgoK1 | 1 | 1 | 1 | 1 | 1 |
| 4402 | 2-dehydro-3-deoxy-6-phosphogalactonate aldolase | 1 | 1 | 1 | 1 | 1 |
| 4404 | Manganese-dependent 2,3-dihydroxybiphenyl 1,2-dioxygenase | 1 | 1 | 1 | 1 | 1 |
| 4405 | hypothetical protein | 1 | 1 | 1 | 1 | 1 |
| 4406 | putative amino acid permease YhdG | 1 | 1 | 1 | 1 | 1 |
| 4407 | hypothetical protein | 1 | 1 | 1 | 1 | 1 |
| 4408 | hypothetical protein | 1 | 1 | 1 | 1 | 1 |
| 4409 | hypothetical protein | 1 | 1 | 1 | 1 | 1 |
| 4411 | hypothetical protein | 1 | 1 | 1 | 1 | 1 |
| 4412 | Iron-sulfur cluster repair protein YtfE | 1 | 1 | 1 | 1 | 1 |
| 4413 | Outer membrane lipoprotein Blc | 1 | 1 | 1 | 1 | 1 |
| 4414 | Sensor protein TorS | 1 | 1 | 1 | 1 | 1 |
| 4415 | Periplasmic protein TorT | 1 | 1 | 1 | 1 | 1 |
| 4416 | TorCAD operon transcriptional regulatory protein TorR | 1 | 1 | 1 | 1 | 1 |
| 4417 | S-fimbrial protein subunit SfaH | 1_p2 | 1 | 1 | 1 | 1 |
| 4418 | Inner membrane protein YjdF | 1 | 1 | 1 | 1 | 1 |
| 4419 | hypothetical protein | 1 | 1 | 1 | 1 | 1 |
| 4420 | hypothetical protein | 1 | 1 | 1 | 1 | 1 |
| 4421 | hypothetical protein | 1 | 1 | 1 | 1 | 1 |
| 4422 | hypothetical protein | 1 | 1 | 1 | 1 | 1 |
| 4423 | hypothetical protein | 1 | 1 | 1 | 1 | 1 |
| 4424 | hypothetical protein | 1 | 1 | 1 | 1 | 1 |
| 4425 | Acetyltransferase YpeA | 1 | 1 | 1 | 1 | 1 |
| 4426 | hypothetical protein | 1 | 1 | 1 | 1 | 1 |
| 4427 | Porin B | 1 | 1 | 1 | 1 | 1 |
| 4428 | PTS system beta-glucoside-specific EIIBCA component | 1 | 1 | 1 | 1 | 1 |
| 4429 | hypothetical protein | 1 | 1 | 1 | 1 | 1 |
| 4438 | Delta(1)-pyrroline-2-carboxylate reductase | 1 | 1 | 1 | 1 | 1 |
| 4372 | hypothetical protein | 1 | 1 | 1 | 1 | 1 |
| 4351 | Glutathione S-transferase GstB | 1 | 1 | 1 | 1 | 1 |
| 920 | Sensor histidine kinase RcsC | 1 | 1 | 2 | 1 | 1 |
| 4335 | hypothetical protein | 1 | 1 | 1 | 1 | 1 |
| 4191 | Lipopolysaccharide export system permease protein LptG | 1 | 1 | 1 | 1 | 1 |
| 4193 | hypothetical protein | 1 | 1 | 1 | 1 | 1 |
| 4085 | Acetyltransferase | 1 | 1 | 1_p1 | 1 | 1 |
| 4086 | hypothetical protein | 1 | 1 | 1_p1 | 1 | 1 |
| 4087 | hypothetical protein | 1 | 1 | 1_p1 | 1 | 1 |
| 4088 | Autoinducer 2 sensor kinase/phosphatase LuxQ | 1 | 1 | 1_p1 | 1 | 1 |
| 4089 | hypothetical protein | 1 | 1 | 1_p1 | 1 | 1 |
| 4090 | hypothetical protein | 1 | 1 | 1_p1 | 1 | 1 |
| 4091 | hypothetical protein | 1 | 1 | 1 | 1 | 1 |
| 4092 | hypothetical protein | 1 | 1 | 1 | 1 | 1 |
| 4093 | Virulence protein | 1 | 1 | 1 | 1 | 1 |
| 4094 | Arginase | 1 | 1 | 1 | 1 | 1 |
| 4095 | hypothetical protein | 1 | 1 | 1 | 1 | 1 |

p = plasmid (1 and 2)

**Supplementary Table 5**. Unique genes from the *Serratia proteamaculans* 568 genome.

| **Family** | **Description (locus tag - product - location)** | **[S_proteamaculans_568_chr]** | **[S_proteamaculans_568_pSPRO01]** | **total** |
| --- | --- | --- | --- | --- |
| No family | SPROTEAMACULANS568_CHR_00051 tRNA(fMet)-specific endonuclease VapC | 1 | 0 | 1 |
| No family | SPROTEAMACULANS568_CHR_00052 hypothetical protein | 1 | 0 | 1 |
| No family | SPROTEAMACULANS568_CHR_00109 hypothetical protein | 1 | 0 | 1 |
| No family | SPROTEAMACULANS568_CHR_00110 hypothetical protein | 1 | 0 | 1 |
| No family | SPROTEAMACULANS568_CHR_00111 hypothetical protein | 1 | 0 | 1 |
| No family | SPROTEAMACULANS568_CHR_00112 HTH-type transcriptional regulator NorG | 1 | 0 | 1 |
| No family | SPROTEAMACULANS568_CHR_00113 hypothetical protein | 1 | 0 | 1 |
| No family | SPROTEAMACULANS568_CHR_00114 Inner membrane protein YiaW | 1 | 0 | 1 |
| No family | SPROTEAMACULANS568_CHR_00116 Transcriptional activator protein EsaR | 1 | 0 | 1 |
| No family | SPROTEAMACULANS568_CHR_00293 Tetanus toxin | 1 | 0 | 1 |
| No family | SPROTEAMACULANS568_CHR_00297 hypothetical protein | 1 | 0 | 1 |
| No family | SPROTEAMACULANS568_CHR_00313 hypothetical protein | 1 | 0 | 1 |
| No family | SPROTEAMACULANS568_CHR_00314 hypothetical protein | 1 | 0 | 1 |
| No family | SPROTEAMACULANS568_CHR_00315 hypothetical protein | 1 | 0 | 1 |
| No family | SPROTEAMACULANS568_CHR_00316 hypothetical protein | 1 | 0 | 1 |
| No family | SPROTEAMACULANS568_CHR_00317 hypothetical protein | 1 | 0 | 1 |
| No family | SPROTEAMACULANS568_CHR_00318 Thymidylate synthase 1 | 1 | 0 | 1 |
| No family | SPROTEAMACULANS568_CHR_00327 hypothetical protein | 1 | 0 | 1 |
| No family | SPROTEAMACULANS568_CHR_00339 putative siderophore biosynthesis protein SbnA | 1 | 0 | 1 |
| No family | SPROTEAMACULANS568_CHR_00340 Delta(1)-pyrroline-2-carboxylate reductase | 1 | 0 | 1 |
| No family | SPROTEAMACULANS568_CHR_00341 hypothetical protein | 1 | 0 | 1 |
| No family | SPROTEAMACULANS568_CHR_00342 Linear gramicidin synthase subunit D | 1 | 0 | 1 |
| No family | SPROTEAMACULANS568_CHR_00345 hypothetical protein | 1 | 0 | 1 |
| No family | SPROTEAMACULANS568_CHR_00346 Asparagine synthetase [glutamine-hydrolyzing] 1 | 1 | 0 | 1 |
| No family | SPROTEAMACULANS568_CHR_00347 hypothetical protein | 1 | 0 | 1 |
| No family | SPROTEAMACULANS568_CHR_00353 hypothetical protein | 1 | 0 | 1 |
| No family | SPROTEAMACULANS568_CHR_00357 hypothetical protein | 1 | 0 | 1 |
| No family | SPROTEAMACULANS568_CHR_00359 hypothetical protein | 1 | 0 | 1 |
| No family | SPROTEAMACULANS568_CHR_00362 hypothetical protein | 1 | 0 | 1 |
| No family | SPROTEAMACULANS568_CHR_00368 hypothetical protein | 1 | 0 | 1 |
| No family | SPROTEAMACULANS568_CHR_00369 hypothetical protein | 1 | 0 | 1 |
| No family | SPROTEAMACULANS568_CHR_00370 hypothetical protein | 1 | 0 | 1 |
| No family | SPROTEAMACULANS568_CHR_00371 Carboxylesterase NlhH | 1 | 0 | 1 |
| No family | SPROTEAMACULANS568_CHR_00372 Organic hydroperoxide resistance transcriptional regulator | 1 | 0 | 1 |
| No family | SPROTEAMACULANS568_CHR_00375 Catalase | 1 | 0 | 1 |
| No family | SPROTEAMACULANS568_CHR_00377 hypothetical protein | 1 | 0 | 1 |
| No family | SPROTEAMACULANS568_CHR_00378 hypothetical protein | 1 | 0 | 1 |
| No family | SPROTEAMACULANS568_CHR_00381 Monoterpene epsilon-lactone hydrolase | 1 | 0 | 1 |
| No family | SPROTEAMACULANS568_CHR_00382 hypothetical protein | 1 | 0 | 1 |
| No family | SPROTEAMACULANS568_CHR_00383 hypothetical protein | 1 | 0 | 1 |
| No family | SPROTEAMACULANS568_CHR_00386 hypothetical protein | 1 | 0 | 1 |
| No family | SPROTEAMACULANS568_CHR_00388 hypothetical protein | 1 | 0 | 1 |
| No family | SPROTEAMACULANS568_CHR_00389 hypothetical protein | 1 | 0 | 1 |
| No family | SPROTEAMACULANS568_CHR_00444 Primosomal replication protein n | 1 | 0 | 1 |
| No family | SPROTEAMACULANS568_CHR_00466 hypothetical protein | 1 | 0 | 1 |
| No family | SPROTEAMACULANS568_CHR_00566 Serine/threonine-protein kinase HipA | 1 | 0 | 1 |
| No family | SPROTEAMACULANS568_CHR_00567 hypothetical protein | 1 | 0 | 1 |
| No family | SPROTEAMACULANS568_CHR_00568 Sensor protein ZraS | 1 | 0 | 1 |
| No family | SPROTEAMACULANS568_CHR_00569 Type-2 restriction enzyme EcoRII | 1 | 0 | 1 |
| No family | SPROTEAMACULANS568_CHR_00570 Very short patch repair protein | 1 | 0 | 1 |
| No family | SPROTEAMACULANS568_CHR_00585 hypothetical protein | 1 | 0 | 1 |
| No family | SPROTEAMACULANS568_CHR_00586 hypothetical protein | 1 | 0 | 1 |
| No family | SPROTEAMACULANS568_CHR_00587 HTH-type transcriptional regulator CynR | 1 | 0 | 1 |
| No family | SPROTEAMACULANS568_CHR_00588 putative succinyl-CoA:3-ketoacid coenzyme A transferase subunit A | 1 | 0 | 1 |
| No family | SPROTEAMACULANS568_CHR_00589 putative succinyl-CoA:3-ketoacid coenzyme A transferase subunit B | 1 | 0 | 1 |
| No family | SPROTEAMACULANS568_CHR_00590 Acetyl-CoA acetyltransferase | 1 | 0 | 1 |
| No family | SPROTEAMACULANS568_CHR_00591 3-hydroxybutyryl-CoA dehydrogenase | 1 | 0 | 1 |
| No family | SPROTEAMACULANS568_CHR_00592 hypothetical protein | 1 | 0 | 1 |
| No family | SPROTEAMACULANS568_CHR_00593 D-beta-hydroxybutyrate dehydrogenase | 1 | 0 | 1 |
| No family | SPROTEAMACULANS568_CHR_00604 hypothetical protein | 1 | 0 | 1 |
| No family | SPROTEAMACULANS568_CHR_00627 3,4-dihydroxyphenylacetate 2,3-dioxygenase | 1 | 0 | 1 |
| No family | SPROTEAMACULANS568_CHR_00696 hypothetical protein | 1 | 0 | 1 |
| No family | SPROTEAMACULANS568_CHR_00713 hypothetical protein | 1 | 0 | 1 |
| No family | SPROTEAMACULANS568_CHR_00806 hypothetical protein | 1 | 0 | 1 |
| No family | SPROTEAMACULANS568_CHR_00813 hypothetical protein | 1 | 0 | 1 |
| No family | SPROTEAMACULANS568_CHR_00814 hypothetical protein | 1 | 0 | 1 |
| No family | SPROTEAMACULANS568_CHR_00816 hypothetical protein | 1 | 0 | 1 |
| No family | SPROTEAMACULANS568_CHR_00871 hypothetical protein | 1 | 0 | 1 |
| No family | SPROTEAMACULANS568_CHR_00876 hypothetical protein | 1 | 0 | 1 |
| No family | SPROTEAMACULANS568_CHR_00913 hypothetical protein | 1 | 0 | 1 |
| No family | SPROTEAMACULANS568_CHR_00918 hypothetical protein | 1 | 0 | 1 |
| No family | SPROTEAMACULANS568_CHR_00978 hypothetical protein | 1 | 0 | 1 |
| No family | SPROTEAMACULANS568_CHR_00982 hypothetical protein | 1 | 0 | 1 |
| No family | SPROTEAMACULANS568_CHR_00983 hypothetical protein | 1 | 0 | 1 |
| No family | SPROTEAMACULANS568_CHR_01028 hypothetical protein | 1 | 0 | 1 |
| No family | SPROTEAMACULANS568_CHR_01039 hypothetical protein | 1 | 0 | 1 |
| No family | SPROTEAMACULANS568_CHR_01040 hypothetical protein | 1 | 0 | 1 |
| No family | SPROTEAMACULANS568_CHR_01152 Copper-exporting P-type ATPase A | 1 | 0 | 1 |
| No family | SPROTEAMACULANS568_CHR_01174 hypothetical protein | 1 | 0 | 1 |
| No family | SPROTEAMACULANS568_CHR_01175 hypothetical protein | 1 | 0 | 1 |
| No family | SPROTEAMACULANS568_CHR_01176 N-ethylmaleimide reductase | 1 | 0 | 1 |
| No family | SPROTEAMACULANS568_CHR_01179 hypothetical protein | 1 | 0 | 1 |
| No family | SPROTEAMACULANS568_CHR_01180 HTH-type transcriptional regulator VirS | 1 | 0 | 1 |
| No family | SPROTEAMACULANS568_CHR_01181 hypothetical protein | 1 | 0 | 1 |
| No family | SPROTEAMACULANS568_CHR_01325 hypothetical protein | 1 | 0 | 1 |
| No family | SPROTEAMACULANS568_CHR_01347 Glycine cleavage system transcriptional activator | 1 | 0 | 1 |
| No family | SPROTEAMACULANS568_CHR_01348 hypothetical protein | 1 | 0 | 1 |
| No family | SPROTEAMACULANS568_CHR_01360 hypothetical protein | 1 | 0 | 1 |
| No family | SPROTEAMACULANS568_CHR_01361 hypothetical protein | 1 | 0 | 1 |
| No family | SPROTEAMACULANS568_CHR_01365 hypothetical protein | 1 | 0 | 1 |
| No family | SPROTEAMACULANS568_CHR_01423 C4-dicarboxylic acid transporter DauA | 1 | 0 | 1 |
| No family | SPROTEAMACULANS568_CHR_01424 Carbonic anhydrase 2 | 1 | 0 | 1 |
| No family | SPROTEAMACULANS568_CHR_01436 hypothetical protein | 1 | 0 | 1 |
| No family | SPROTEAMACULANS568_CHR_01449 Major myo-inositol transporter IolT | 1 | 0 | 1 |
| No family | SPROTEAMACULANS568_CHR_01465 hypothetical protein | 1 | 0 | 1 |
| No family | SPROTEAMACULANS568_CHR_01502 hypothetical protein | 1 | 0 | 1 |
| No family | SPROTEAMACULANS568_CHR_01503 hypothetical protein | 1 | 0 | 1 |
| No family | SPROTEAMACULANS568_CHR_01535 HTH-type transcriptional repressor Bm3R1 | 1 | 0 | 1 |
| No family | SPROTEAMACULANS568_CHR_01541 hypothetical protein | 1 | 0 | 1 |
| No family | SPROTEAMACULANS568_CHR_01546 hypothetical protein | 1 | 0 | 1 |
| No family | SPROTEAMACULANS568_CHR_01547 hypothetical protein | 1 | 0 | 1 |
| No family | SPROTEAMACULANS568_CHR_01548 hypothetical protein | 1 | 0 | 1 |
| No family | SPROTEAMACULANS568_CHR_01549 hypothetical protein | 1 | 0 | 1 |
| No family | SPROTEAMACULANS568_CHR_01550 hypothetical protein | 1 | 0 | 1 |
| No family | SPROTEAMACULANS568_CHR_01551 hypothetical protein | 1 | 0 | 1 |
| No family | SPROTEAMACULANS568_CHR_01552 hypothetical protein | 1 | 0 | 1 |
| No family | SPROTEAMACULANS568_CHR_01564 hypothetical protein | 1 | 0 | 1 |
| No family | SPROTEAMACULANS568_CHR_01565 Toxin CcdB | 1 | 0 | 1 |
| No family | SPROTEAMACULANS568_CHR_01582 hypothetical protein | 1 | 0 | 1 |
| No family | SPROTEAMACULANS568_CHR_01635 hypothetical protein | 1 | 0 | 1 |
| No family | SPROTEAMACULANS568_CHR_01704 hypothetical protein | 1 | 0 | 1 |
| No family | SPROTEAMACULANS568_CHR_01705 hypothetical protein | 1 | 0 | 1 |
| No family | SPROTEAMACULANS568_CHR_01706 Uracil phosphoribosyltransferase | 1 | 0 | 1 |
| No family | SPROTEAMACULANS568_CHR_01707 Tautomerase PptA | 1 | 0 | 1 |
| No family | SPROTEAMACULANS568_CHR_01708 Pyrophosphatase PpaX | 1 | 0 | 1 |
| No family | SPROTEAMACULANS568_CHR_01709 Tyrocidine synthase 3 | 1 | 0 | 1 |
| No family | SPROTEAMACULANS568_CHR_01779 hypothetical protein | 1 | 0 | 1 |
| No family | SPROTEAMACULANS568_CHR_01789 hypothetical protein | 1 | 0 | 1 |
| No family | SPROTEAMACULANS568_CHR_01791 hypothetical protein | 1 | 0 | 1 |
| No family | SPROTEAMACULANS568_CHR_01797 hypothetical protein | 1 | 0 | 1 |
| No family | SPROTEAMACULANS568_CHR_01798 Outer membrane porin F | 1 | 0 | 1 |
| No family | SPROTEAMACULANS568_CHR_01799 Major exported protein | 1 | 0 | 1 |
| No family | SPROTEAMACULANS568_CHR_01802 hypothetical protein | 1 | 0 | 1 |
| No family | SPROTEAMACULANS568_CHR_01806 hypothetical protein | 1 | 0 | 1 |
| No family | SPROTEAMACULANS568_CHR_01807 hypothetical protein | 1 | 0 | 1 |
| No family | SPROTEAMACULANS568_CHR_01808 hypothetical protein | 1 | 0 | 1 |
| No family | SPROTEAMACULANS568_CHR_01809 hypothetical protein | 1 | 0 | 1 |
| No family | SPROTEAMACULANS568_CHR_01810 hypothetical protein | 1 | 0 | 1 |
| No family | SPROTEAMACULANS568_CHR_01813 hypothetical protein | 1 | 0 | 1 |
| No family | SPROTEAMACULANS568_CHR_01814 hypothetical protein | 1 | 0 | 1 |
| No family | SPROTEAMACULANS568_CHR_01815 hypothetical protein | 1 | 0 | 1 |
| No family | SPROTEAMACULANS568_CHR_01816 hypothetical protein | 1 | 0 | 1 |
| No family | SPROTEAMACULANS568_CHR_01857 hypothetical protein | 1 | 0 | 1 |
| No family | SPROTEAMACULANS568_CHR_01898 NADPH dehydrogenase | 1 | 0 | 1 |
| No family | SPROTEAMACULANS568_CHR_01926 hypothetical protein | 1 | 0 | 1 |
| No family | SPROTEAMACULANS568_CHR_01960 hypothetical protein | 1 | 0 | 1 |
| No family | SPROTEAMACULANS568_CHR_01970 HTH-type transcriptional regulator DmlR | 1 | 0 | 1 |
| No family | SPROTEAMACULANS568_CHR_01994 hypothetical protein | 1 | 0 | 1 |
| No family | SPROTEAMACULANS568_CHR_01995 hypothetical protein | 1 | 0 | 1 |
| No family | SPROTEAMACULANS568_CHR_01996 hypothetical protein | 1 | 0 | 1 |
| No family | SPROTEAMACULANS568_CHR_02041 hypothetical protein | 1 | 0 | 1 |
| No family | SPROTEAMACULANS568_CHR_02042 hypothetical protein | 1 | 0 | 1 |
| No family | SPROTEAMACULANS568_CHR_02043 hypothetical protein | 1 | 0 | 1 |
| No family | SPROTEAMACULANS568_CHR_02047 hypothetical protein | 1 | 0 | 1 |
| No family | SPROTEAMACULANS568_CHR_02053 hypothetical protein | 1 | 0 | 1 |
| No family | SPROTEAMACULANS568_CHR_02054 hypothetical protein | 1 | 0 | 1 |
| No family | SPROTEAMACULANS568_CHR_02055 hypothetical protein | 1 | 0 | 1 |
| No family | SPROTEAMACULANS568_CHR_02056 hypothetical protein | 1 | 0 | 1 |
| No family | SPROTEAMACULANS568_CHR_02060 hypothetical protein | 1 | 0 | 1 |
| No family | SPROTEAMACULANS568_CHR_02061 hypothetical protein | 1 | 0 | 1 |
| No family | SPROTEAMACULANS568_CHR_02062 hypothetical protein | 1 | 0 | 1 |
| No family | SPROTEAMACULANS568_CHR_02064 hypothetical protein | 1 | 0 | 1 |
| No family | SPROTEAMACULANS568_CHR_02066 Putative prophage major tail sheath protein | 1 | 0 | 1 |
| No family | SPROTEAMACULANS568_CHR_02067 hypothetical protein | 1 | 0 | 1 |
| No family | SPROTEAMACULANS568_CHR_02068 hypothetical protein | 1 | 0 | 1 |
| No family | SPROTEAMACULANS568_CHR_02069 hypothetical protein | 1 | 0 | 1 |
| No family | SPROTEAMACULANS568_CHR_02070 Leucine-responsive regulatory protein | 1 | 0 | 1 |
| No family | SPROTEAMACULANS568_CHR_02071 putative inner membrane transporter YedA | 1 | 0 | 1 |
| No family | SPROTEAMACULANS568_CHR_02072 Isocitrate dehydrogenase [NADP] | 1 | 0 | 1 |
| No family | SPROTEAMACULANS568_CHR_02075 putative tautomerase | 1 | 0 | 1 |
| No family | SPROTEAMACULANS568_CHR_02080 hypothetical protein | 1 | 0 | 1 |
| No family | SPROTEAMACULANS568_CHR_02084 hypothetical protein | 1 | 0 | 1 |
| No family | SPROTEAMACULANS568_CHR_02110 Fructose dehydrogenase cytochrome subunit | 1 | 0 | 1 |
| No family | SPROTEAMACULANS568_CHR_02111 Fructose dehydrogenase large subunit | 1 | 0 | 1 |
| No family | SPROTEAMACULANS568_CHR_02112 Fructose dehydrogenase small subunit | 1 | 0 | 1 |
| No family | SPROTEAMACULANS568_CHR_02113 hypothetical protein | 1 | 0 | 1 |
| No family | SPROTEAMACULANS568_CHR_02114 hypothetical protein | 1 | 0 | 1 |
| No family | SPROTEAMACULANS568_CHR_02115 4-oxalomesaconate tautomerase | 1 | 0 | 1 |
| No family | SPROTEAMACULANS568_CHR_02117 4-oxalmesaconate hydratase | 1 | 0 | 1 |
| No family | SPROTEAMACULANS568_CHR_02119 3-dehydroquinate dehydratase | 1 | 0 | 1 |
| No family | SPROTEAMACULANS568_CHR_02120 4-sulfomuconolactone hydrolase | 1 | 0 | 1 |
| No family | SPROTEAMACULANS568_CHR_02121 Gallate dioxygenase | 1 | 0 | 1 |
| No family | SPROTEAMACULANS568_CHR_02122 4-hydroxybenzoate transporter PcaK | 1 | 0 | 1 |
| No family | SPROTEAMACULANS568_CHR_02125 Serralysin | 1 | 0 | 1 |
| No family | SPROTEAMACULANS568_CHR_02141 Paraquat-inducible protein B | 1 | 0 | 1 |
| No family | SPROTEAMACULANS568_CHR_02148 hypothetical protein | 1 | 0 | 1 |
| No family | SPROTEAMACULANS568_CHR_02191 hypothetical protein | 1 | 0 | 1 |
| No family | SPROTEAMACULANS568_CHR_02197 hypothetical protein | 1 | 0 | 1 |
| No family | SPROTEAMACULANS568_CHR_02260 hypothetical protein | 1 | 0 | 1 |
| No family | SPROTEAMACULANS568_CHR_02283 hypothetical protein | 1 | 0 | 1 |
| No family | SPROTEAMACULANS568_CHR_02300 hypothetical protein | 1 | 0 | 1 |
| No family | SPROTEAMACULANS568_CHR_02305 hypothetical protein | 1 | 0 | 1 |
| No family | SPROTEAMACULANS568_CHR_02306 PTS system maltose- and glucose-specific EIICB component | 1 | 0 | 1 |
| No family | SPROTEAMACULANS568_CHR_02339 hypothetical protein | 1 | 0 | 1 |
| No family | SPROTEAMACULANS568_CHR_02340 Metallothiol transferase FosB | 1 | 0 | 1 |
| No family | SPROTEAMACULANS568_CHR_02341 2-hydroxymuconate semialdehyde hydrolase | 1 | 0 | 1 |
| No family | SPROTEAMACULANS568_CHR_02352 hypothetical protein | 1 | 0 | 1 |
| No family | SPROTEAMACULANS568_CHR_02367 2-phosphosulfolactate phosphatase | 1 | 0 | 1 |
| No family | SPROTEAMACULANS568_CHR_02368 Acetyltransferase YpeA | 1 | 0 | 1 |
| No family | SPROTEAMACULANS568_CHR_02377 Glucosyl-3-phosphoglycerate synthase | 1 | 0 | 1 |
| No family | SPROTEAMACULANS568_CHR_02378 Putative mannosyl-3-phosphoglycerate phosphatase | 1 | 0 | 1 |
| No family | SPROTEAMACULANS568_CHR_02379 hypothetical protein | 1 | 0 | 1 |
| No family | SPROTEAMACULANS568_CHR_02380 hypothetical protein | 1 | 0 | 1 |
| No family | SPROTEAMACULANS568_CHR_02390 Multidrug resistance protein Stp | 1 | 0 | 1 |
| No family | SPROTEAMACULANS568_CHR_02391 HTH-type transcriptional regulator YofA | 1 | 0 | 1 |
| No family | SPROTEAMACULANS568_CHR_02395 hypothetical protein | 1 | 0 | 1 |
| No family | SPROTEAMACULANS568_CHR_02397 hypothetical protein | 1 | 0 | 1 |
| No family | SPROTEAMACULANS568_CHR_02410 3 beta-hydroxysteroid dehydrogenase/Delta 5-->4-isomerase | 1 | 0 | 1 |
| No family | SPROTEAMACULANS568_CHR_02411 HTH-type transcriptional repressor NemR | 1 | 0 | 1 |
| No family | SPROTEAMACULANS568_CHR_02412 hypothetical protein | 1 | 0 | 1 |
| No family | SPROTEAMACULANS568_CHR_02413 hypothetical protein | 1 | 0 | 1 |
| No family | SPROTEAMACULANS568_CHR_02414 hypothetical protein | 1 | 0 | 1 |
| No family | SPROTEAMACULANS568_CHR_02415 hypothetical protein | 1 | 0 | 1 |
| No family | SPROTEAMACULANS568_CHR_02416 hypothetical protein | 1 | 0 | 1 |
| No family | SPROTEAMACULANS568_CHR_02417 hypothetical protein | 1 | 0 | 1 |
| No family | SPROTEAMACULANS568_CHR_02418 hypothetical protein | 1 | 0 | 1 |
| No family | SPROTEAMACULANS568_CHR_02419 hypothetical protein | 1 | 0 | 1 |
| No family | SPROTEAMACULANS568_CHR_02420 hypothetical protein | 1 | 0 | 1 |
| No family | SPROTEAMACULANS568_CHR_02426 hypothetical protein | 1 | 0 | 1 |
| No family | SPROTEAMACULANS568_CHR_02455 hypothetical protein | 1 | 0 | 1 |
| No family | SPROTEAMACULANS568_CHR_02456 Carboxylesterase A | 1 | 0 | 1 |
| No family | SPROTEAMACULANS568_CHR_02460 putative diguanylate cyclase YegE | 1 | 0 | 1 |
| No family | SPROTEAMACULANS568_CHR_02461 hypothetical protein | 1 | 0 | 1 |
| No family | SPROTEAMACULANS568_CHR_02488 RNA pyrophosphohydrolase | 1 | 0 | 1 |
| No family | SPROTEAMACULANS568_CHR_02492 2-ketogluconate reductase | 1 | 0 | 1 |
| No family | SPROTEAMACULANS568_CHR_02495 HTH-type transcriptional regulator PgrR | 1 | 0 | 1 |
| No family | SPROTEAMACULANS568_CHR_02498 Tellurite resistance protein TehA | 1 | 0 | 1 |
| No family | SPROTEAMACULANS568_CHR_02534 Cysteine desulfurase | 1 | 0 | 1 |
| No family | SPROTEAMACULANS568_CHR_02535 Major membrane protein I | 1 | 0 | 1 |
| No family | SPROTEAMACULANS568_CHR_02536 Serine acetyltransferase | 1 | 0 | 1 |
| No family | SPROTEAMACULANS568_CHR_02537 hypothetical protein | 1 | 0 | 1 |
| No family | SPROTEAMACULANS568_CHR_02538 Thiosulfate sulfurtransferase GlpE | 1 | 0 | 1 |
| No family | SPROTEAMACULANS568_CHR_02550 hypothetical protein | 1 | 0 | 1 |
| No family | SPROTEAMACULANS568_CHR_02551 hypothetical protein | 1 | 0 | 1 |
| No family | SPROTEAMACULANS568_CHR_02552 Outer membrane protein assembly factor BamA | 1 | 0 | 1 |
| No family | SPROTEAMACULANS568_CHR_02553 hypothetical protein | 1 | 0 | 1 |
| No family | SPROTEAMACULANS568_CHR_02554 4-hydroxybenzoate transporter PcaK | 1 | 0 | 1 |
| No family | SPROTEAMACULANS568_CHR_02555 hypothetical protein | 1 | 0 | 1 |
| No family | SPROTEAMACULANS568_CHR_02578 hypothetical protein | 1 | 0 | 1 |
| No family | SPROTEAMACULANS568_CHR_02584 Vitamin B12-binding protein | 1 | 0 | 1 |
| No family | SPROTEAMACULANS568_CHR_02679 hypothetical protein | 1 | 0 | 1 |
| No family | SPROTEAMACULANS568_CHR_02735 hypothetical protein | 1 | 0 | 1 |
| No family | SPROTEAMACULANS568_CHR_02864 Multidrug resistance protein Stp | 1 | 0 | 1 |
| No family | SPROTEAMACULANS568_CHR_02951 Siroheme synthase | 1 | 0 | 1 |
| No family | SPROTEAMACULANS568_CHR_02952 Nitrate reductase | 1 | 0 | 1 |
| No family | SPROTEAMACULANS568_CHR_02954 Bicarbonate transport ATP-binding protein CmpD | 1 | 0 | 1 |
| No family | SPROTEAMACULANS568_CHR_02955 Bicarbonate transport system permease protein CmpB | 1 | 0 | 1 |
| No family | SPROTEAMACULANS568_CHR_02956 Nitrate transport protein NrtA | 1 | 0 | 1 |
| No family | SPROTEAMACULANS568_CHR_02957 Nitrate regulatory protein | 1 | 0 | 1 |
| No family | SPROTEAMACULANS568_CHR_02991 hypothetical protein | 1 | 0 | 1 |
| No family | SPROTEAMACULANS568_CHR_02992 hypothetical protein | 1 | 0 | 1 |
| No family | SPROTEAMACULANS568_CHR_02993 hypothetical protein | 1 | 0 | 1 |
| No family | SPROTEAMACULANS568_CHR_03023 hypothetical protein | 1 | 0 | 1 |
| No family | SPROTEAMACULANS568_CHR_03024 Antitoxin igA-2 | 1 | 0 | 1 |
| No family | SPROTEAMACULANS568_CHR_03046 hypothetical protein | 1 | 0 | 1 |
| No family | SPROTEAMACULANS568_CHR_03047 hypothetical protein | 1 | 0 | 1 |
| No family | SPROTEAMACULANS568_CHR_03049 Motility protein B | 1 | 0 | 1 |
| No family | SPROTEAMACULANS568_CHR_03050 hypothetical protein | 1 | 0 | 1 |
| No family | SPROTEAMACULANS568_CHR_03051 hypothetical protein | 1 | 0 | 1 |
| No family | SPROTEAMACULANS568_CHR_03052 hypothetical protein | 1 | 0 | 1 |
| No family | SPROTEAMACULANS568_CHR_03056 hypothetical protein | 1 | 0 | 1 |
| No family | SPROTEAMACULANS568_CHR_03057 Serine/threonine phosphatase stp | 1 | 0 | 1 |
| No family | SPROTEAMACULANS568_CHR_03058 hypothetical protein | 1 | 0 | 1 |
| No family | SPROTEAMACULANS568_CHR_03059 hypothetical protein | 1 | 0 | 1 |
| No family | SPROTEAMACULANS568_CHR_03060 hypothetical protein | 1 | 0 | 1 |
| No family | SPROTEAMACULANS568_CHR_03064 Serine/threonine-protein kinase PK-1 | 1 | 0 | 1 |
| No family | SPROTEAMACULANS568_CHR_03066 hypothetical protein | 1 | 0 | 1 |
| No family | SPROTEAMACULANS568_CHR_03067 putative deoxyribonuclease RhsA | 1 | 0 | 1 |
| No family | SPROTEAMACULANS568_CHR_03068 ADP-ribosyl-[dinitrogen reductase] glycohydrolase | 1 | 0 | 1 |
| No family | SPROTEAMACULANS568_CHR_03069 Major exported protein | 1 | 0 | 1 |
| No family | SPROTEAMACULANS568_CHR_03070 hypothetical protein | 1 | 0 | 1 |
| No family | SPROTEAMACULANS568_CHR_03071 DNA gyrase inhibitor | 1 | 0 | 1 |
| No family | SPROTEAMACULANS568_CHR_03082 Anaerobic nitric oxide reductase transcription regulator NorR | 1 | 0 | 1 |
| No family | SPROTEAMACULANS568_CHR_03083 Anaerobic nitric oxide reductase flavorubredoxin | 1 | 0 | 1 |
| No family | SPROTEAMACULANS568_CHR_03084 Nitric oxide reductase FlRd-NAD(+) reductase | 1 | 0 | 1 |
| No family | SPROTEAMACULANS568_CHR_03113 hypothetical protein | 1 | 0 | 1 |
| No family | SPROTEAMACULANS568_CHR_03156 Chitin-binding protein CbpD | 1 | 0 | 1 |
| No family | SPROTEAMACULANS568_CHR_03164 hypothetical protein | 1 | 0 | 1 |
| No family | SPROTEAMACULANS568_CHR_03181 HTH-type transcriptional regulator DmlR | 1 | 0 | 1 |
| No family | SPROTEAMACULANS568_CHR_03182 Inner membrane transport protein YdhP | 1 | 0 | 1 |
| No family | SPROTEAMACULANS568_CHR_03188 Purine ribonucleoside efflux pump NepI | 1 | 0 | 1 |
| No family | SPROTEAMACULANS568_CHR_03192 Penicillin G acylase | 1 | 0 | 1 |
| No family | SPROTEAMACULANS568_CHR_03193 hypothetical protein | 1 | 0 | 1 |
| No family | SPROTEAMACULANS568_CHR_03225 Putative 2-dehydropantoate 2-reductase | 1 | 0 | 1 |
| No family | SPROTEAMACULANS568_CHR_03228 Glutathione-regulated potassium-efflux system ancillary protein KefF | 1 | 0 | 1 |
| No family | SPROTEAMACULANS568_CHR_03229 HTH-type transcriptional regulator PgrR | 1 | 0 | 1 |
| No family | SPROTEAMACULANS568_CHR_03231 Aconitate isomerase | 1 | 0 | 1 |
| No family | SPROTEAMACULANS568_CHR_03232 HTH-type transcriptional regulator CynR | 1 | 0 | 1 |
| No family | SPROTEAMACULANS568_CHR_03234 Sulfoacetaldehyde reductase | 1 | 0 | 1 |
| No family | SPROTEAMACULANS568_CHR_03236 Sodium/proline symporter | 1 | 0 | 1 |
| No family | SPROTEAMACULANS568_CHR_03237 Sodium/proline symporter | 1 | 0 | 1 |
| No family | SPROTEAMACULANS568_CHR_03238 Carbamate kinase 1 | 1 | 0 | 1 |
| No family | SPROTEAMACULANS568_CHR_03239 N(G),N(G)-dimethylarginine dimethylaminohydrolase | 1 | 0 | 1 |
| No family | SPROTEAMACULANS568_CHR_03240 putative amino acid permease YhdG | 1 | 0 | 1 |
| No family | SPROTEAMACULANS568_CHR_03241 Ornithine carbamoyltransferase | 1 | 0 | 1 |
| No family | SPROTEAMACULANS568_CHR_03242 HTH-type transcriptional regulator CynR | 1 | 0 | 1 |
| No family | SPROTEAMACULANS568_CHR_03255 hypothetical protein | 1 | 0 | 1 |
| No family | SPROTEAMACULANS568_CHR_03327 hypothetical protein | 1 | 0 | 1 |
| No family | SPROTEAMACULANS568_CHR_03392 hypothetical protein | 1 | 0 | 1 |
| No family | SPROTEAMACULANS568_CHR_03393 hypothetical protein | 1 | 0 | 1 |
| No family | SPROTEAMACULANS568_CHR_03408 hypothetical protein | 1 | 0 | 1 |
| No family | SPROTEAMACULANS568_CHR_03409 hypothetical protein | 1 | 0 | 1 |
| No family | SPROTEAMACULANS568_CHR_03410 Decarbamoylnovobiocin carbamoyltransferase | 1 | 0 | 1 |
| No family | SPROTEAMACULANS568_CHR_03413 hypothetical protein | 1 | 0 | 1 |
| No family | SPROTEAMACULANS568_CHR_03497 hypothetical protein | 1 | 0 | 1 |
| No family | SPROTEAMACULANS568_CHR_03498 hypothetical protein | 1 | 0 | 1 |
| No family | SPROTEAMACULANS568_CHR_03585 hypothetical protein | 1 | 0 | 1 |
| No family | SPROTEAMACULANS568_CHR_03587 hypothetical protein | 1 | 0 | 1 |
| No family | SPROTEAMACULANS568_CHR_03590 hypothetical protein | 1 | 0 | 1 |
| No family | SPROTEAMACULANS568_CHR_03605 Putative ribosomal N-acetyltransferase YdaF | 1 | 0 | 1 |
| No family | SPROTEAMACULANS568_CHR_03644 hypothetical protein | 1 | 0 | 1 |
| No family | SPROTEAMACULANS568_CHR_03650 Spermidine N(1)-acetyltransferase | 1 | 0 | 1 |
| No family | SPROTEAMACULANS568_CHR_03651 Sugar efflux transporter B | 1 | 0 | 1 |
| No family | SPROTEAMACULANS568_CHR_03692 Cytidine deaminase | 1 | 0 | 1 |
| No family | SPROTEAMACULANS568_CHR_03693 hypothetical protein | 1 | 0 | 1 |
| No family | SPROTEAMACULANS568_CHR_03748 hypothetical protein | 1 | 0 | 1 |
| No family | SPROTEAMACULANS568_CHR_03749 hypothetical protein | 1 | 0 | 1 |
| No family | SPROTEAMACULANS568_CHR_03754 hypothetical protein | 1 | 0 | 1 |
| No family | SPROTEAMACULANS568_CHR_03756 Multidrug export protein AcrE | 1 | 0 | 1 |
| No family | SPROTEAMACULANS568_CHR_03758 MltA-interacting protein | 1 | 0 | 1 |
| No family | SPROTEAMACULANS568_CHR_03761 hypothetical protein | 1 | 0 | 1 |
| No family | SPROTEAMACULANS568_CHR_03769 Multidrug resistance protein MdtG | 1 | 0 | 1 |
| No family | SPROTEAMACULANS568_CHR_03784 hypothetical protein | 1 | 0 | 1 |
| No family | SPROTEAMACULANS568_CHR_03800 hypothetical protein | 1 | 0 | 1 |
| No family | SPROTEAMACULANS568_CHR_03860 NADPH-dependent 7-cyano-7-deazaguanine reductase | 1 | 0 | 1 |
| No family | SPROTEAMACULANS568_CHR_03883 hypothetical protein | 1 | 0 | 1 |
| No family | SPROTEAMACULANS568_CHR_03923 Cold shock-like protein CspG | 1 | 0 | 1 |
| No family | SPROTEAMACULANS568_CHR_03927 3-oxoacyl-[acyl-carrier-protein] reductase FabG | 1 | 0 | 1 |
| No family | SPROTEAMACULANS568_CHR_03928 Putative aldehyde dehydrogenase AldA | 1 | 0 | 1 |
| No family | SPROTEAMACULANS568_CHR_03934 hypothetical protein | 1 | 0 | 1 |
| No family | SPROTEAMACULANS568_CHR_03935 hypothetical protein | 1 | 0 | 1 |
| No family | SPROTEAMACULANS568_CHR_03939 hypothetical protein | 1 | 0 | 1 |
| No family | SPROTEAMACULANS568_CHR_03942 hypothetical protein | 1 | 0 | 1 |
| No family | SPROTEAMACULANS568_CHR_03943 hypothetical protein | 1 | 0 | 1 |
| No family | SPROTEAMACULANS568_CHR_03944 hypothetical protein | 1 | 0 | 1 |
| No family | SPROTEAMACULANS568_CHR_03948 hypothetical protein | 1 | 0 | 1 |
| No family | SPROTEAMACULANS568_CHR_03954 hypothetical protein | 1 | 0 | 1 |
| No family | SPROTEAMACULANS568_CHR_03975 hypothetical protein | 1 | 0 | 1 |
| No family | SPROTEAMACULANS568_CHR_03976 hypothetical protein | 1 | 0 | 1 |
| No family | SPROTEAMACULANS568_CHR_04020 tRNA-binding protein YgjH | 1 | 0 | 1 |
| No family | SPROTEAMACULANS568_CHR_04021 hypothetical protein | 1 | 0 | 1 |
| No family | SPROTEAMACULANS568_CHR_04025 hypothetical protein | 1 | 0 | 1 |
| No family | SPROTEAMACULANS568_CHR_04046 Toxin HokB | 1 | 0 | 1 |
| No family | SPROTEAMACULANS568_CHR_04085 Inner membrane symporter YicJ | 1 | 0 | 1 |
| No family | SPROTEAMACULANS568_CHR_04086 Extracellular exo-alpha-(1->5)-L-arabinofuranosidase | 1 | 0 | 1 |
| No family | SPROTEAMACULANS568_CHR_04117 Phytochrome-like protein cph2 | 1 | 0 | 1 |
| No family | SPROTEAMACULANS568_CHR_04121 ATP-dependent helicase/nuclease subunit A | 1 | 0 | 1 |
| No family | SPROTEAMACULANS568_CHR_04131 hypothetical protein | 1 | 0 | 1 |
| No family | SPROTEAMACULANS568_CHR_04132 hypothetical protein | 1 | 0 | 1 |
| No family | SPROTEAMACULANS568_CHR_04135 hypothetical protein | 1 | 0 | 1 |
| No family | SPROTEAMACULANS568_CHR_04136 putative hydrolase YutF | 1 | 0 | 1 |
| No family | SPROTEAMACULANS568_CHR_04137 hypothetical protein | 1 | 0 | 1 |
| No family | SPROTEAMACULANS568_CHR_04138 Aspartate aminotransferase | 1 | 0 | 1 |
| No family | SPROTEAMACULANS568_CHR_04141 hypothetical protein | 1 | 0 | 1 |
| No family | SPROTEAMACULANS568_CHR_04142 hypothetical protein | 1 | 0 | 1 |
| No family | SPROTEAMACULANS568_CHR_04148 hypothetical protein | 1 | 0 | 1 |
| No family | SPROTEAMACULANS568_CHR_04149 hypothetical protein | 1 | 0 | 1 |
| No family | SPROTEAMACULANS568_CHR_04150 hypothetical protein | 1 | 0 | 1 |
| No family | SPROTEAMACULANS568_CHR_04154 hypothetical protein | 1 | 0 | 1 |
| No family | SPROTEAMACULANS568_CHR_04156 hypothetical protein | 1 | 0 | 1 |
| No family | SPROTEAMACULANS568_CHR_04157 Type-1 restriction enzyme R protein | 1 | 0 | 1 |
| No family | SPROTEAMACULANS568_CHR_04171 hypothetical protein | 1 | 0 | 1 |
| No family | SPROTEAMACULANS568_CHR_04178 hypothetical protein | 1 | 0 | 1 |
| No family | SPROTEAMACULANS568_CHR_04179 hypothetical protein | 1 | 0 | 1 |
| No family | SPROTEAMACULANS568_CHR_04182 hypothetical protein | 1 | 0 | 1 |
| No family | SPROTEAMACULANS568_CHR_04206 hypothetical protein | 1 | 0 | 1 |
| No family | SPROTEAMACULANS568_CHR_04208 hypothetical protein | 1 | 0 | 1 |
| No family | SPROTEAMACULANS568_CHR_04210 hypothetical protein | 1 | 0 | 1 |
| No family | SPROTEAMACULANS568_CHR_04211 hypothetical protein | 1 | 0 | 1 |
| No family | SPROTEAMACULANS568_CHR_04212 hypothetical protein | 1 | 0 | 1 |
| No family | SPROTEAMACULANS568_CHR_04215 hypothetical protein | 1 | 0 | 1 |
| No family | SPROTEAMACULANS568_CHR_04220 putative fimbrial-like protein YfcQ | 1 | 0 | 1 |
| No family | SPROTEAMACULANS568_CHR_04223 putative fimbrial-like protein YfcR | 1 | 0 | 1 |
| No family | SPROTEAMACULANS568_CHR_04224 PAP fimbrial minor pilin protein | 1 | 0 | 1 |
| No family | SPROTEAMACULANS568_CHR_04231 hypothetical protein | 1 | 0 | 1 |
| No family | SPROTEAMACULANS568_CHR_04232 FKBP-type peptidyl-prolyl cis-trans isomerase FkpA | 1 | 0 | 1 |
| No family | SPROTEAMACULANS568_CHR_04233 hypothetical protein | 1 | 0 | 1 |
| No family | SPROTEAMACULANS568_CHR_04234 hypothetical protein | 1 | 0 | 1 |
| No family | SPROTEAMACULANS568_CHR_04235 putative fimbrial chaperone YehC | 1 | 0 | 1 |
| No family | SPROTEAMACULANS568_CHR_04236 Outer membrane usher protein YehB | 1 | 0 | 1 |
| No family | SPROTEAMACULANS568_CHR_04237 hypothetical protein | 1 | 0 | 1 |
| No family | SPROTEAMACULANS568_CHR_04238 hypothetical protein | 1 | 0 | 1 |
| No family | SPROTEAMACULANS568_CHR_04239 hypothetical protein | 1 | 0 | 1 |
| No family | SPROTEAMACULANS568_CHR_04240 hypothetical protein | 1 | 0 | 1 |
| No family | SPROTEAMACULANS568_CHR_04249 FMN-dependent NADH-azoreductase | 1 | 0 | 1 |
| No family | SPROTEAMACULANS568_CHR_04269 hypothetical protein | 1 | 0 | 1 |
| No family | SPROTEAMACULANS568_CHR_04271 hypothetical protein | 1 | 0 | 1 |
| No family | SPROTEAMACULANS568_CHR_04272 hypothetical protein | 1 | 0 | 1 |
| No family | SPROTEAMACULANS568_CHR_04303 HTH-type transcriptional regulatory protein GabR | 1 | 0 | 1 |
| No family | SPROTEAMACULANS568_CHR_04321 hypothetical protein | 1 | 0 | 1 |
| No family | SPROTEAMACULANS568_CHR_04322 GlcNAc-binding protein A | 1 | 0 | 1 |
| No family | SPROTEAMACULANS568_CHR_04324 Type 4 prepilin-like proteins leader peptide-processing enzyme | 1 | 0 | 1 |
| No family | SPROTEAMACULANS568_CHR_04325 hypothetical protein | 1 | 0 | 1 |
| No family | SPROTEAMACULANS568_CHR_04326 Type II secretion system protein L | 1 | 0 | 1 |
| No family | SPROTEAMACULANS568_CHR_04327 Putative type II secretion system protein K | 1 | 0 | 1 |
| No family | SPROTEAMACULANS568_CHR_04328 Type II secretion system protein J | 1 | 0 | 1 |
| No family | SPROTEAMACULANS568_CHR_04329 Putative type II secretion system protein I | 1 | 0 | 1 |
| No family | SPROTEAMACULANS568_CHR_04330 Putative type II secretion system protein H | 1 | 0 | 1 |
| No family | SPROTEAMACULANS568_CHR_04331 Type II secretion system protein G | 1 | 0 | 1 |
| No family | SPROTEAMACULANS568_CHR_04332 Putative type II secretion system protein F | 1 | 0 | 1 |
| No family | SPROTEAMACULANS568_CHR_04333 Type II secretion system protein E | 1 | 0 | 1 |
| No family | SPROTEAMACULANS568_CHR_04334 Type II secretion system protein D | 1 | 0 | 1 |
| No family | SPROTEAMACULANS568_CHR_04335 hypothetical protein | 1 | 0 | 1 |
| No family | SPROTEAMACULANS568_CHR_04336 hypothetical protein | 1 | 0 | 1 |
| No family | SPROTEAMACULANS568_CHR_04384 hypothetical protein | 1 | 0 | 1 |
| No family | SPROTEAMACULANS568_CHR_04394 hypothetical protein | 1 | 0 | 1 |
| No family | SPROTEAMACULANS568_CHR_04395 Serine/threonine-protein kinase HipA | 1 | 0 | 1 |
| No family | SPROTEAMACULANS568_CHR_04469 hypothetical protein | 1 | 0 | 1 |
| No family | SPROTEAMACULANS568_CHR_04470 hypothetical protein | 1 | 0 | 1 |
| No family | SPROTEAMACULANS568_CHR_04481 hypothetical protein | 1 | 0 | 1 |
| No family | SPROTEAMACULANS568_CHR_04482 tRNA nuclease CdiA | 1 | 0 | 1 |
| No family | SPROTEAMACULANS568_CHR_04483 hypothetical protein | 1 | 0 | 1 |
| No family | SPROTEAMACULANS568_CHR_04484 hypothetical protein | 1 | 0 | 1 |
| No family | SPROTEAMACULANS568_CHR_04505 Carbonic anhydrase | 1 | 0 | 1 |
| No family | SPROTEAMACULANS568_CHR_04517 hypothetical protein | 1 | 0 | 1 |
| No family | SPROTEAMACULANS568_CHR_04521 Membrane-bound lytic murein transglycosylase F | 1 | 0 | 1 |
| No family | SPROTEAMACULANS568_CHR_04650 Glucose--fructose oxidoreductase | 1 | 0 | 1 |
| No family | SPROTEAMACULANS568_CHR_04651 Inosose dehydratase | 1 | 0 | 1 |
| No family | SPROTEAMACULANS568_CHR_04652 Inositol 2-dehydrogenase/D-chiro-inositol 3-dehydrogenase | 1 | 0 | 1 |
| No family | SPROTEAMACULANS568_CHR_04653 Putative nucleoside transporter YegT | 1 | 0 | 1 |
| No family | SPROTEAMACULANS568_CHR_04654 hypothetical protein | 1 | 0 | 1 |
| No family | SPROTEAMACULANS568_CHR_04655 hypothetical protein | 1 | 0 | 1 |
| No family | SPROTEAMACULANS568_CHR_04656 HTH-type transcriptional repressor CytR | 1 | 0 | 1 |
| No family | SPROTEAMACULANS568_CHR_04704 PCP degradation transcriptional activation protein | 1 | 0 | 1 |
| No family | SPROTEAMACULANS568_CHR_04705 Betaine aldehyde dehydrogenase | 1 | 0 | 1 |
| No family | SPROTEAMACULANS568_CHR_04706 Gentisate 1,2-dioxygenase | 1 | 0 | 1 |
| No family | SPROTEAMACULANS568_CHR_04707 Ureidoglycolate lyase | 1 | 0 | 1 |
| No family | SPROTEAMACULANS568_CHR_04724 hypothetical protein | 1 | 0 | 1 |
| No family | SPROTEAMACULANS568_CHR_04766 hypothetical protein | 1 | 0 | 1 |
| No family | SPROTEAMACULANS568_CHR_04767 HTH-type transcriptional regulator PgrR | 1 | 0 | 1 |
| No family | SPROTEAMACULANS568_CHR_04768 NADH oxidase | 1 | 0 | 1 |
| No family | SPROTEAMACULANS568_CHR_04785 HTH-type transcriptional repressor RspR | 1 | 0 | 1 |
| No family | SPROTEAMACULANS568_CHR_04786 Sialic acid TRAP transporter permease protein SiaT | 1 | 0 | 1 |
| No family | SPROTEAMACULANS568_CHR_04787 Sialic acid TRAP transporter permease protein SiaT | 1 | 0 | 1 |
| No family | SPROTEAMACULANS568_CHR_04788 2,3-diketo-L-gulonate-binding periplasmic protein YiaO | 1 | 0 | 1 |
| No family | SPROTEAMACULANS568_CHR_04789 L-galactonate-5-dehydrogenase | 1 | 0 | 1 |
| No family | SPROTEAMACULANS568_CHR_04790 2-dehydro-3-deoxy-D-gluconate 5-dehydrogenase | 1 | 0 | 1 |
| No family | SPROTEAMACULANS568_CHR_04791 Mandelate racemase | 1 | 0 | 1 |
| No family | SPROTEAMACULANS568_CHR_04798 hypothetical protein | 1 | 0 | 1 |
| No family | SPROTEAMACULANS568_CHR_04800 hypothetical protein | 1 | 0 | 1 |
| No family | SPROTEAMACULANS568_CHR_04804 hypothetical protein | 1 | 0 | 1 |
| No family | SPROTEAMACULANS568_CHR_04811 hypothetical protein | 1 | 0 | 1 |
| No family | SPROTEAMACULANS568_CHR_04813 hypothetical protein | 1 | 0 | 1 |
| No family | SPROTEAMACULANS568_CHR_04814 hypothetical protein | 1 | 0 | 1 |
| No family | SPROTEAMACULANS568_CHR_04818 RCS-specific HTH-type transcriptional activator RclR | 1 | 0 | 1 |
| No family | SPROTEAMACULANS568_CHR_04819 hypothetical protein | 1 | 0 | 1 |
| No family | SPROTEAMACULANS568_CHR_04825 hypothetical protein | 1 | 0 | 1 |
| No family | SPROTEAMACULANS568_CHR_04826 hypothetical protein | 1 | 0 | 1 |
| No family | SPROTEAMACULANS568_CHR_04840 hypothetical protein | 1 | 0 | 1 |
| No family | SPROTEAMACULANS568_CHR_04841 hypothetical protein | 1 | 0 | 1 |
| No family | SPROTEAMACULANS568_CHR_04855 hypothetical protein | 1 | 0 | 1 |
| No family | SPROTEAMACULANS568_CHR_04909 Biodegradative arginine decarboxylase | 1 | 0 | 1 |
| No family | SPROTEAMACULANS568_CHR_04947 hypothetical protein | 1 | 0 | 1 |
| No family | SPROTEAMACULANS568_CHR_04951 HTH-type transcriptional regulator PgrR | 1 | 0 | 1 |
| No family | SPROTEAMACULANS568_CHR_04962 GlcNAc-binding protein A | 1 | 0 | 1 |
| No family | SPROTEAMACULANS568_PSPRO01_00001 hypothetical protein | 0 | 1 | 1 |
| No family | SPROTEAMACULANS568_PSPRO01_00002 hypothetical protein | 0 | 1 | 1 |
| No family | SPROTEAMACULANS568_PSPRO01_00003 hypothetical protein | 0 | 1 | 1 |
| No family | SPROTEAMACULANS568_PSPRO01_00004 hypothetical protein | 0 | 1 | 1 |
| No family | SPROTEAMACULANS568_PSPRO01_00005 hypothetical protein | 0 | 1 | 1 |
| No family | SPROTEAMACULANS568_PSPRO01_00006 hypothetical protein | 0 | 1 | 1 |
| No family | SPROTEAMACULANS568_PSPRO01_00007 hypothetical protein | 0 | 1 | 1 |
| No family | SPROTEAMACULANS568_PSPRO01_00008 hypothetical protein | 0 | 1 | 1 |
| No family | SPROTEAMACULANS568_PSPRO01_00009 hypothetical protein | 0 | 1 | 1 |
| No family | SPROTEAMACULANS568_PSPRO01_00010 hypothetical protein | 0 | 1 | 1 |
| No family | SPROTEAMACULANS568_PSPRO01_00011 hypothetical protein | 0 | 1 | 1 |
| No family | SPROTEAMACULANS568_PSPRO01_00012 hypothetical protein | 0 | 1 | 1 |
| No family | SPROTEAMACULANS568_PSPRO01_00013 hypothetical protein | 0 | 1 | 1 |
| No family | SPROTEAMACULANS568_PSPRO01_00016 hypothetical protein | 0 | 1 | 1 |
| No family | SPROTEAMACULANS568_PSPRO01_00018 hypothetical protein | 0 | 1 | 1 |
| No family | SPROTEAMACULANS568_PSPRO01_00020 hypothetical protein | 0 | 1 | 1 |
| No family | SPROTEAMACULANS568_PSPRO01_00021 hypothetical protein | 0 | 1 | 1 |
| No family | SPROTEAMACULANS568_PSPRO01_00023 hypothetical protein | 0 | 1 | 1 |
| No family | SPROTEAMACULANS568_PSPRO01_00025 hypothetical protein | 0 | 1 | 1 |
| No family | SPROTEAMACULANS568_PSPRO01_00027 hypothetical protein | 0 | 1 | 1 |
| No family | SPROTEAMACULANS568_PSPRO01_00030 hypothetical protein | 0 | 1 | 1 |
| No family | SPROTEAMACULANS568_PSPRO01_00031 hypothetical protein | 0 | 1 | 1 |
| No family | SPROTEAMACULANS568_PSPRO01_00033 hypothetical protein | 0 | 1 | 1 |
| No family | SPROTEAMACULANS568_PSPRO01_00034 hypothetical protein | 0 | 1 | 1 |
| No family | SPROTEAMACULANS568_PSPRO01_00035 hypothetical protein | 0 | 1 | 1 |
| No family | SPROTEAMACULANS568_PSPRO01_00038 hypothetical protein | 0 | 1 | 1 |
| No family | SPROTEAMACULANS568_PSPRO01_00042 hypothetical protein | 0 | 1 | 1 |
| No family | SPROTEAMACULANS568_PSPRO01_00043 hypothetical protein | 0 | 1 | 1 |
| No family | SPROTEAMACULANS568_PSPRO01_00044 hypothetical protein | 0 | 1 | 1 |
| No family | SPROTEAMACULANS568_PSPRO01_00045 hypothetical protein | 0 | 1 | 1 |
| No family | SPROTEAMACULANS568_PSPRO01_00046 hypothetical protein | 0 | 1 | 1 |
| No family | SPROTEAMACULANS568_PSPRO01_00048 hypothetical protein | 0 | 1 | 1 |
| No family | SPROTEAMACULANS568_PSPRO01_00049 hypothetical protein | 0 | 1 | 1 |
| No family | SPROTEAMACULANS568_PSPRO01_00050 hypothetical protein | 0 | 1 | 1 |
| No family | SPROTEAMACULANS568_PSPRO01_00051 hypothetical protein | 0 | 1 | 1 |
| No family | SPROTEAMACULANS568_PSPRO01_00052 hypothetical protein | 0 | 1 | 1 |
| No family | SPROTEAMACULANS568_PSPRO01_00053 hypothetical protein | 0 | 1 | 1 |
| No family | SPROTEAMACULANS568_PSPRO01_00054 hypothetical protein | 0 | 1 | 1 |
| No family | SPROTEAMACULANS568_PSPRO01_00055 hypothetical protein | 0 | 1 | 1 |
| No family | SPROTEAMACULANS568_PSPRO01_00056 hypothetical protein | 0 | 1 | 1 |
| No family | SPROTEAMACULANS568_PSPRO01_00057 hypothetical protein | 0 | 1 | 1 |
| No family | SPROTEAMACULANS568_PSPRO01_00058 hypothetical protein | 0 | 1 | 1 |
| No family | SPROTEAMACULANS568_PSPRO01_00059 hypothetical protein | 0 | 1 | 1 |
| No family | SPROTEAMACULANS568_PSPRO01_00060 hypothetical protein | 0 | 1 | 1 |
| No family | SPROTEAMACULANS568_PSPRO01_00061 hypothetical protein | 0 | 1 | 1 |
| No family | SPROTEAMACULANS568_PSPRO01_00062 hypothetical protein | 0 | 1 | 1 |
| No family | SPROTEAMACULANS568_PSPRO01_00063 hypothetical protein | 0 | 1 | 1 |
| No family | SPROTEAMACULANS568_PSPRO01_00065 mRNA interferase HigB | 0 | 1 | 1 |
| No family | SPROTEAMACULANS568_PSPRO01_00066 hypothetical protein | 0 | 1 | 1 |
| No family | SPROTEAMACULANS568_PSPRO01_00067 hypothetical protein | 0 | 1 | 1 |
| No family | SPROTEAMACULANS568_PSPRO01_00068 Replication initiation protein | 0 | 1 | 1 |
| No family | SPROTEAMACULANS568_PSPRO01_00071 hypothetical protein | 0 | 1 | 1 |
| No family | SPROTEAMACULANS568_PSPRO01_00072 hypothetical protein | 0 | 1 | 1 |
| No family | SPROTEAMACULANS568_PSPRO01_00073 hypothetical protein | 0 | 1 | 1 |
| No family | SPROTEAMACULANS568_PSPRO01_00074 hypothetical protein | 0 | 1 | 1 |
| No family | SPROTEAMACULANS568_PSPRO01_00075 hypothetical protein | 0 | 1 | 1 |
|  | TOTAL | 445 | 56 | ## |
| **Family** | **Description (product)** | **[S_proteamaculans_568_chr]** | **[S_proteamaculans_568_pSPRO01]** | **total** |
| 123 | hypothetical protein | 5 | 0 | 5 |
| 4059 | putative fimbrial subunit LpfE | 4 | 0 | 4 |
| 4450 | hypothetical protein | 3 | 0 | 3 |
| 4454 | hypothetical protein | 3 | 0 | 3 |
| 4456 | hypothetical protein | 3 | 0 | 3 |
| 4457 | Actin cross-linking toxin VgrG1 | 3 | 0 | 3 |
| 4459 | Transcriptional regulatory protein RcsB | 3 | 0 | 3 |
| 4462 | Tyrosine recombinase XerC | 3 | 0 | 3 |
| 4463 | hypothetical protein | 3 | 0 | 3 |
| 4464 | hypothetical protein | 3 | 0 | 3 |
| 4465 | Toxin YkfI | 3 | 0 | 3 |
| 4466 | hypothetical protein | 3 | 0 | 3 |
| 4467 | hypothetical protein | 3 | 0 | 3 |
| 4472 | hypothetical protein | 3 | 0 | 3 |
| 4473 | hypothetical protein | 3 | 0 | 3 |
| 4474 | hypothetical protein | 3 | 0 | 3 |
| 4475 | hypothetical protein | 3 | 0 | 3 |
| 4476 | hypothetical protein | 3 | 0 | 3 |
| 4477 | Phosphoribosylamine--glycine ligase | 3 | 0 | 3 |
| 4478 | hypothetical protein | 3 | 0 | 3 |
| 4479 | hypothetical protein | 3 | 0 | 3 |
| 4480 | hypothetical protein | 3 | 0 | 3 |
| 4481 | hypothetical protein | 3 | 0 | 3 |
| 4482 | hypothetical protein | 3 | 0 | 3 |
| 4483 | hypothetical protein | 3 | 0 | 3 |
| 4484 | hypothetical protein | 3 | 0 | 3 |
| 4485 | hypothetical protein | 3 | 0 | 3 |
| 4486 | hypothetical protein | 3 | 0 | 3 |
| 4487 | hypothetical protein | 3 | 0 | 3 |
| 4488 | hypothetical protein | 3 | 0 | 3 |
| 4489 | hypothetical protein | 3 | 0 | 3 |
| 4490 | hypothetical protein | 3 | 0 | 3 |
| 4491 | hypothetical protein | 3 | 0 | 3 |
| 4492 | hypothetical protein | 3 | 0 | 3 |
| 4493 | hypothetical protein | 3 | 0 | 3 |
| 4494 | hypothetical protein | 3 | 0 | 3 |
| 4779 | hypothetical protein | 0 | 2 | 2 |
| 4784 | putative chromosome-partitioning protein ParB | 0 | 2 | 2 |
| 4791 | HTH-type transcriptional activator RhaS | 2 | 0 | 2 |
| 4792 | hypothetical protein | 2 | 0 | 2 |
| 4798 | hypothetical protein | 2 | 0 | 2 |
| 4808 | hypothetical protein | 2 | 0 | 2 |
| 4810 | Lipoprotein OutS | 2 | 0 | 2 |
| 4827 | hypothetical protein | 2 | 0 | 2 |
| 4832 | putative PhzA/B-like protein | 2 | 0 | 2 |
| 4833 | hypothetical protein | 2 | 0 | 2 |
| 4834 | hypothetical protein | 2 | 0 | 2 |
| 4835 | hypothetical protein | 2 | 0 | 2 |
| 4836 | hypothetical protein | 2 | 0 | 2 |
| 4837 | hypothetical protein | 2 | 0 | 2 |
| 4844 | Aryl-alcohol dehydrogenase | 2 | 0 | 2 |
| 4845 | Putative alkyl/aryl-sulfatase YjcS | 2 | 0 | 2 |
| 4846 | Type-1 fimbrial protein, A chain | 2 | 0 | 2 |
| 4849 | hypothetical protein | 2 | 0 | 2 |
| 4850 | hypothetical protein | 2 | 0 | 2 |
| 4852 | hypothetical protein | 2 | 0 | 2 |
| 4854 | hypothetical protein | 2 | 0 | 2 |
| 4855 | Single-stranded DNA-binding protein | 2 | 0 | 2 |
| 4856 | hypothetical protein | 2 | 0 | 2 |
| 4857 | DNA topoisomerase 3 | 2 | 0 | 2 |
| 4858 | hypothetical protein | 2 | 0 | 2 |
| 4859 | hypothetical protein | 2 | 0 | 2 |
| 4860 | hypothetical protein | 2 | 0 | 2 |
| 4861 | hypothetical protein | 2 | 0 | 2 |
| 4862 | hypothetical protein | 2 | 0 | 2 |
| 4863 | hypothetical protein | 2 | 0 | 2 |
| 4864 | Replicative DNA helicase | 2 | 0 | 2 |
| 4865 | Tyrosine-protein kinase YwqD | 2 | 0 | 2 |
|  | TOTAL | 171 | 4 | 175 |

**Supplementary Table 6.** Unique genes from the *Serratia liquefaciens* FG3 genome.

| **Family** | **Description (locus tag - product - location)** | **[S_ufop_01_chr]** | **[S_ufop_01_p1]** | **[S_ufop_01_p2]** | **total** |
| --- | --- | --- | --- | --- | --- |
| No family | SUFOP01_CHR_00001 hypothetical protein | 1 | 0 | 0 | 1 |
| No family | SUFOP01_CHR_00144 Putative prophage CPS-53 integrase | 1 | 0 | 0 | 1 |
| No family | SUFOP01_CHR_00145 Prophage CP4-57 integrase | 1 | 0 | 0 | 1 |
| No family | SUFOP01_CHR_00146 hypothetical protein | 1 | 0 | 0 | 1 |
| No family | SUFOP01_CHR_00154 hypothetical protein | 1 | 0 | 0 | 1 |
| No family | SUFOP01_CHR_00155 hypothetical protein | 1 | 0 | 0 | 1 |
| No family | SUFOP01_CHR_00158 hypothetical protein | 1 | 0 | 0 | 1 |
| No family | SUFOP01_CHR_00161 hypothetical protein | 1 | 0 | 0 | 1 |
| No family | SUFOP01_CHR_00323 Acid shock protein | 1 | 0 | 0 | 1 |
| No family | SUFOP01_CHR_00346 hypothetical protein | 1 | 0 | 0 | 1 |
| No family | SUFOP01_CHR_00347 hypothetical protein | 1 | 0 | 0 | 1 |
| No family | SUFOP01_CHR_00348 hypothetical protein | 1 | 0 | 0 | 1 |
| No family | SUFOP01_CHR_00349 hypothetical protein | 1 | 0 | 0 | 1 |
| No family | SUFOP01_CHR_00350 hypothetical protein | 1 | 0 | 0 | 1 |
| No family | SUFOP01_CHR_00351 hypothetical protein | 1 | 0 | 0 | 1 |
| No family | SUFOP01_CHR_00353 Tyrosine recombinase XerD | 1 | 0 | 0 | 1 |
| No family | SUFOP01_CHR_00355 hypothetical protein | 1 | 0 | 0 | 1 |
| No family | SUFOP01_CHR_00488 Putative prophage CPS-53 integrase | 1 | 0 | 0 | 1 |
| No family | SUFOP01_CHR_00489 hypothetical protein | 1 | 0 | 0 | 1 |
| No family | SUFOP01_CHR_00493 hypothetical protein | 1 | 0 | 0 | 1 |
| No family | SUFOP01_CHR_00494 hypothetical protein | 1 | 0 | 0 | 1 |
| No family | SUFOP01_CHR_00495 Exodeoxyribonuclease 8 | 1 | 0 | 0 | 1 |
| No family | SUFOP01_CHR_00496 hypothetical protein | 1 | 0 | 0 | 1 |
| No family | SUFOP01_CHR_00497 hypothetical protein | 1 | 0 | 0 | 1 |
| No family | SUFOP01_CHR_00499 hypothetical protein | 1 | 0 | 0 | 1 |
| No family | SUFOP01_CHR_00500 hypothetical protein | 1 | 0 | 0 | 1 |
| No family | SUFOP01_CHR_00501 hypothetical protein | 1 | 0 | 0 | 1 |
| No family | SUFOP01_CHR_00504 hypothetical protein | 1 | 0 | 0 | 1 |
| No family | SUFOP01_CHR_00505 RNA polymerase-binding transcription factor DksA | 1 | 0 | 0 | 1 |
| No family | SUFOP01_CHR_00506 hypothetical protein | 1 | 0 | 0 | 1 |
| No family | SUFOP01_CHR_00507 hypothetical protein | 1 | 0 | 0 | 1 |
| No family | SUFOP01_CHR_00509 hypothetical protein | 1 | 0 | 0 | 1 |
| No family | SUFOP01_CHR_00514 hypothetical protein | 1 | 0 | 0 | 1 |
| No family | SUFOP01_CHR_00517 hypothetical protein | 1 | 0 | 0 | 1 |
| No family | SUFOP01_CHR_00518 hypothetical protein | 1 | 0 | 0 | 1 |
| No family | SUFOP01_CHR_00519 hypothetical protein | 1 | 0 | 0 | 1 |
| No family | SUFOP01_CHR_00520 hypothetical protein | 1 | 0 | 0 | 1 |
| No family | SUFOP01_CHR_00521 hypothetical protein | 1 | 0 | 0 | 1 |
| No family | SUFOP01_CHR_00522 hypothetical protein | 1 | 0 | 0 | 1 |
| No family | SUFOP01_CHR_00523 hypothetical protein | 1 | 0 | 0 | 1 |
| No family | SUFOP01_CHR_00524 hypothetical protein | 1 | 0 | 0 | 1 |
| No family | SUFOP01_CHR_00525 hypothetical protein | 1 | 0 | 0 | 1 |
| No family | SUFOP01_CHR_00526 hypothetical protein | 1 | 0 | 0 | 1 |
| No family | SUFOP01_CHR_00527 hypothetical protein | 1 | 0 | 0 | 1 |
| No family | SUFOP01_CHR_00528 hypothetical protein | 1 | 0 | 0 | 1 |
| No family | SUFOP01_CHR_00529 hypothetical protein | 1 | 0 | 0 | 1 |
| No family | SUFOP01_CHR_00530 hypothetical protein | 1 | 0 | 0 | 1 |
| No family | SUFOP01_CHR_00531 hypothetical protein | 1 | 0 | 0 | 1 |
| No family | SUFOP01_CHR_00532 hypothetical protein | 1 | 0 | 0 | 1 |
| No family | SUFOP01_CHR_00534 hypothetical protein | 1 | 0 | 0 | 1 |
| No family | SUFOP01_CHR_00535 hypothetical protein | 1 | 0 | 0 | 1 |
| No family | SUFOP01_CHR_00536 hypothetical protein | 1 | 0 | 0 | 1 |
| No family | SUFOP01_CHR_00538 hypothetical protein | 1 | 0 | 0 | 1 |
| No family | SUFOP01_CHR_00539 hypothetical protein | 1 | 0 | 0 | 1 |
| No family | SUFOP01_CHR_00540 hypothetical protein | 1 | 0 | 0 | 1 |
| No family | SUFOP01_CHR_00541 hypothetical protein | 1 | 0 | 0 | 1 |
| No family | SUFOP01_CHR_00542 hypothetical protein | 1 | 0 | 0 | 1 |
| No family | SUFOP01_CHR_00543 hypothetical protein | 1 | 0 | 0 | 1 |
| No family | SUFOP01_CHR_00544 hypothetical protein | 1 | 0 | 0 | 1 |
| No family | SUFOP01_CHR_00546 hypothetical protein | 1 | 0 | 0 | 1 |
| No family | SUFOP01_CHR_00547 hypothetical protein | 1 | 0 | 0 | 1 |
| No family | SUFOP01_CHR_00632 hypothetical protein | 1 | 0 | 0 | 1 |
| No family | SUFOP01_CHR_00634 hypothetical protein | 1 | 0 | 0 | 1 |
| No family | SUFOP01_CHR_00642 hypothetical protein | 1 | 0 | 0 | 1 |
| No family | SUFOP01_CHR_00667 Toxin HokB | 1 | 0 | 0 | 1 |
| No family | SUFOP01_CHR_00679 Isochorismate pyruvate lyase | 1 | 0 | 0 | 1 |
| No family | SUFOP01_CHR_00783 CDP-diacylglycerol pyrophosphatase | 1 | 0 | 0 | 1 |
| No family | SUFOP01_CHR_00896 hypothetical protein | 1 | 0 | 0 | 1 |
| No family | SUFOP01_CHR_00941 hypothetical protein | 1 | 0 | 0 | 1 |
| No family | SUFOP01_CHR_00945 hypothetical protein | 1 | 0 | 0 | 1 |
| No family | SUFOP01_CHR_00946 hypothetical protein | 1 | 0 | 0 | 1 |
| No family | SUFOP01_CHR_00952 hypothetical protein | 1 | 0 | 0 | 1 |
| No family | SUFOP01_CHR_00953 hypothetical protein | 1 | 0 | 0 | 1 |
| No family | SUFOP01_CHR_00954 hypothetical protein | 1 | 0 | 0 | 1 |
| No family | SUFOP01_CHR_00967 hypothetical protein | 1 | 0 | 0 | 1 |
| No family | SUFOP01_CHR_00969 hypothetical protein | 1 | 0 | 0 | 1 |
| No family | SUFOP01_CHR_00972 hypothetical protein | 1 | 0 | 0 | 1 |
| No family | SUFOP01_CHR_00973 hypothetical protein | 1 | 0 | 0 | 1 |
| No family | SUFOP01_CHR_00974 hypothetical protein | 1 | 0 | 0 | 1 |
| No family | SUFOP01_CHR_00975 hypothetical protein | 1 | 0 | 0 | 1 |
| No family | SUFOP01_CHR_00977 hypothetical protein | 1 | 0 | 0 | 1 |
| No family | SUFOP01_CHR_00978 hypothetical protein | 1 | 0 | 0 | 1 |
| No family | SUFOP01_CHR_00983 hypothetical protein | 1 | 0 | 0 | 1 |
| No family | SUFOP01_CHR_00984 hypothetical protein | 1 | 0 | 0 | 1 |
| No family | SUFOP01_CHR_01070 hypothetical protein | 1 | 0 | 0 | 1 |
| No family | SUFOP01_CHR_01071 hypothetical protein | 1 | 0 | 0 | 1 |
| No family | SUFOP01_CHR_01078 hypothetical protein | 1 | 0 | 0 | 1 |
| No family | SUFOP01_CHR_01079 ATP-dependent Clp protease ATP-binding subunit ClpX | 1 | 0 | 0 | 1 |
| No family | SUFOP01_CHR_01081 hypothetical protein | 1 | 0 | 0 | 1 |
| No family | SUFOP01_CHR_01082 hypothetical protein | 1 | 0 | 0 | 1 |
| No family | SUFOP01_CHR_01083 hypothetical protein | 1 | 0 | 0 | 1 |
| No family | SUFOP01_CHR_01084 hypothetical protein | 1 | 0 | 0 | 1 |
| No family | SUFOP01_CHR_01086 hypothetical protein | 1 | 0 | 0 | 1 |
| No family | SUFOP01_CHR_01087 hypothetical protein | 1 | 0 | 0 | 1 |
| No family | SUFOP01_CHR_01088 hypothetical protein | 1 | 0 | 0 | 1 |
| No family | SUFOP01_CHR_01089 hypothetical protein | 1 | 0 | 0 | 1 |
| No family | SUFOP01_CHR_01090 hypothetical protein | 1 | 0 | 0 | 1 |
| No family | SUFOP01_CHR_01091 hypothetical protein | 1 | 0 | 0 | 1 |
| No family | SUFOP01_CHR_01092 hypothetical protein | 1 | 0 | 0 | 1 |
| No family | SUFOP01_CHR_01093 hypothetical protein | 1 | 0 | 0 | 1 |
| No family | SUFOP01_CHR_01094 hypothetical protein | 1 | 0 | 0 | 1 |
| No family | SUFOP01_CHR_01095 hypothetical protein | 1 | 0 | 0 | 1 |
| No family | SUFOP01_CHR_01098 hypothetical protein | 1 | 0 | 0 | 1 |
| No family | SUFOP01_CHR_01099 hypothetical protein | 1 | 0 | 0 | 1 |
| No family | SUFOP01_CHR_01100 hypothetical protein | 1 | 0 | 0 | 1 |
| No family | SUFOP01_CHR_01101 hypothetical protein | 1 | 0 | 0 | 1 |
| No family | SUFOP01_CHR_01102 hypothetical protein | 1 | 0 | 0 | 1 |
| No family | SUFOP01_CHR_01103 hypothetical protein | 1 | 0 | 0 | 1 |
| No family | SUFOP01_CHR_01105 hypothetical protein | 1 | 0 | 0 | 1 |
| No family | SUFOP01_CHR_01106 hypothetical protein | 1 | 0 | 0 | 1 |
| No family | SUFOP01_CHR_01107 hypothetical protein | 1 | 0 | 0 | 1 |
| No family | SUFOP01_CHR_01108 hypothetical protein | 1 | 0 | 0 | 1 |
| No family | SUFOP01_CHR_01110 hypothetical protein | 1 | 0 | 0 | 1 |
| No family | SUFOP01_CHR_01112 hypothetical protein | 1 | 0 | 0 | 1 |
| No family | SUFOP01_CHR_01113 hypothetical protein | 1 | 0 | 0 | 1 |
| No family | SUFOP01_CHR_01114 hypothetical protein | 1 | 0 | 0 | 1 |
| No family | SUFOP01_CHR_01115 hypothetical protein | 1 | 0 | 0 | 1 |
| No family | SUFOP01_CHR_01119 hypothetical protein | 1 | 0 | 0 | 1 |
| No family | SUFOP01_CHR_01120 hypothetical protein | 1 | 0 | 0 | 1 |
| No family | SUFOP01_CHR_01121 hypothetical protein | 1 | 0 | 0 | 1 |
| No family | SUFOP01_CHR_01122 hypothetical protein | 1 | 0 | 0 | 1 |
| No family | SUFOP01_CHR_01123 hypothetical protein | 1 | 0 | 0 | 1 |
| No family | SUFOP01_CHR_01124 hypothetical protein | 1 | 0 | 0 | 1 |
| No family | SUFOP01_CHR_01125 hypothetical protein | 1 | 0 | 0 | 1 |
| No family | SUFOP01_CHR_01127 hypothetical protein | 1 | 0 | 0 | 1 |
| No family | SUFOP01_CHR_01128 hypothetical protein | 1 | 0 | 0 | 1 |
| No family | SUFOP01_CHR_01130 hypothetical protein | 1 | 0 | 0 | 1 |
| No family | SUFOP01_CHR_01131 hypothetical protein | 1 | 0 | 0 | 1 |
| No family | SUFOP01_CHR_01132 hypothetical protein | 1 | 0 | 0 | 1 |
| No family | SUFOP01_CHR_01133 hypothetical protein | 1 | 0 | 0 | 1 |
| No family | SUFOP01_CHR_01134 hypothetical protein | 1 | 0 | 0 | 1 |
| No family | SUFOP01_CHR_01135 hypothetical protein | 1 | 0 | 0 | 1 |
| No family | SUFOP01_CHR_01137 hypothetical protein | 1 | 0 | 0 | 1 |
| No family | SUFOP01_CHR_01139 hypothetical protein | 1 | 0 | 0 | 1 |
| No family | SUFOP01_CHR_01142 hypothetical protein | 1 | 0 | 0 | 1 |
| No family | SUFOP01_CHR_01143 Tyrosine recombinase XerC | 1 | 0 | 0 | 1 |
| No family | SUFOP01_CHR_01144 HTH-type transcriptional regulator DmlR | 1 | 0 | 0 | 1 |
| No family | SUFOP01_CHR_01165 hypothetical protein | 1 | 0 | 0 | 1 |
| No family | SUFOP01_CHR_01173 hypothetical protein | 1 | 0 | 0 | 1 |
| No family | SUFOP01_CHR_01196 hypothetical protein | 1 | 0 | 0 | 1 |
| No family | SUFOP01_CHR_01267 hypothetical protein | 1 | 0 | 0 | 1 |
| No family | SUFOP01_CHR_01268 Tyrosine recombinase XerD | 1 | 0 | 0 | 1 |
| No family | SUFOP01_CHR_01269 hypothetical protein | 1 | 0 | 0 | 1 |
| No family | SUFOP01_CHR_01270 hypothetical protein | 1 | 0 | 0 | 1 |
| No family | SUFOP01_CHR_01271 hypothetical protein | 1 | 0 | 0 | 1 |
| No family | SUFOP01_CHR_01272 Exodeoxyribonuclease 8 | 1 | 0 | 0 | 1 |
| No family | SUFOP01_CHR_01273 hypothetical protein | 1 | 0 | 0 | 1 |
| No family | SUFOP01_CHR_01274 hypothetical protein | 1 | 0 | 0 | 1 |
| No family | SUFOP01_CHR_01275 hypothetical protein | 1 | 0 | 0 | 1 |
| No family | SUFOP01_CHR_01276 hypothetical protein | 1 | 0 | 0 | 1 |
| No family | SUFOP01_CHR_01277 hypothetical protein | 1 | 0 | 0 | 1 |
| No family | SUFOP01_CHR_01278 hypothetical protein | 1 | 0 | 0 | 1 |
| No family | SUFOP01_CHR_01279 hypothetical protein | 1 | 0 | 0 | 1 |
| No family | SUFOP01_CHR_01280 hypothetical protein | 1 | 0 | 0 | 1 |
| No family | SUFOP01_CHR_01281 hypothetical protein | 1 | 0 | 0 | 1 |
| No family | SUFOP01_CHR_01282 hypothetical protein | 1 | 0 | 0 | 1 |
| No family | SUFOP01_CHR_01283 hypothetical protein | 1 | 0 | 0 | 1 |
| No family | SUFOP01_CHR_01284 hypothetical protein | 1 | 0 | 0 | 1 |
| No family | SUFOP01_CHR_01286 hypothetical protein | 1 | 0 | 0 | 1 |
| No family | SUFOP01_CHR_01287 hypothetical protein | 1 | 0 | 0 | 1 |
| No family | SUFOP01_CHR_01292 DNA-damage-inducible protein I | 1 | 0 | 0 | 1 |
| No family | SUFOP01_CHR_01293 hypothetical protein | 1 | 0 | 0 | 1 |
| No family | SUFOP01_CHR_01296 hypothetical protein | 1 | 0 | 0 | 1 |
| No family | SUFOP01_CHR_01301 hypothetical protein | 1 | 0 | 0 | 1 |
| No family | SUFOP01_CHR_01302 hypothetical protein | 1 | 0 | 0 | 1 |
| No family | SUFOP01_CHR_01303 hypothetical protein | 1 | 0 | 0 | 1 |
| No family | SUFOP01_CHR_01304 hypothetical protein | 1 | 0 | 0 | 1 |
| No family | SUFOP01_CHR_01305 hypothetical protein | 1 | 0 | 0 | 1 |
| No family | SUFOP01_CHR_01306 hypothetical protein | 1 | 0 | 0 | 1 |
| No family | SUFOP01_CHR_01307 hypothetical protein | 1 | 0 | 0 | 1 |
| No family | SUFOP01_CHR_01308 hypothetical protein | 1 | 0 | 0 | 1 |
| No family | SUFOP01_CHR_01309 hypothetical protein | 1 | 0 | 0 | 1 |
| No family | SUFOP01_CHR_01310 hypothetical protein | 1 | 0 | 0 | 1 |
| No family | SUFOP01_CHR_01311 hypothetical protein | 1 | 0 | 0 | 1 |
| No family | SUFOP01_CHR_01312 hypothetical protein | 1 | 0 | 0 | 1 |
| No family | SUFOP01_CHR_01313 hypothetical protein | 1 | 0 | 0 | 1 |
| No family | SUFOP01_CHR_01314 hypothetical protein | 1 | 0 | 0 | 1 |
| No family | SUFOP01_CHR_01316 hypothetical protein | 1 | 0 | 0 | 1 |
| No family | SUFOP01_CHR_01317 hypothetical protein | 1 | 0 | 0 | 1 |
| No family | SUFOP01_CHR_01318 hypothetical protein | 1 | 0 | 0 | 1 |
| No family | SUFOP01_CHR_01319 hypothetical protein | 1 | 0 | 0 | 1 |
| No family | SUFOP01_CHR_01320 hypothetical protein | 1 | 0 | 0 | 1 |
| No family | SUFOP01_CHR_01321 hypothetical protein | 1 | 0 | 0 | 1 |
| No family | SUFOP01_CHR_01323 hypothetical protein | 1 | 0 | 0 | 1 |
| No family | SUFOP01_CHR_01326 hypothetical protein | 1 | 0 | 0 | 1 |
| No family | SUFOP01_CHR_01327 hypothetical protein | 1 | 0 | 0 | 1 |
| No family | SUFOP01_CHR_01328 hypothetical protein | 1 | 0 | 0 | 1 |
| No family | SUFOP01_CHR_01329 hypothetical protein | 1 | 0 | 0 | 1 |
| No family | SUFOP01_CHR_01423 hypothetical protein | 1 | 0 | 0 | 1 |
| No family | SUFOP01_CHR_01552 putative glycosyltransferase EpsJ | 1 | 0 | 0 | 1 |
| No family | SUFOP01_CHR_01571 hypothetical protein | 1 | 0 | 0 | 1 |
| No family | SUFOP01_CHR_01800 hypothetical protein | 1 | 0 | 0 | 1 |
| No family | SUFOP01_CHR_01801 hypothetical protein | 1 | 0 | 0 | 1 |
| No family | SUFOP01_CHR_01962 Formate hydrogenlyase subunit 7 | 1 | 0 | 0 | 1 |
| No family | SUFOP01_CHR_01975 Oxygen-dependent choline dehydrogenase | 1 | 0 | 0 | 1 |
| No family | SUFOP01_CHR_02069 hypothetical protein | 1 | 0 | 0 | 1 |
| No family | SUFOP01_CHR_02165 hypothetical protein | 1 | 0 | 0 | 1 |
| No family | SUFOP01_CHR_02180 Lipase | 1 | 0 | 0 | 1 |
| No family | SUFOP01_CHR_02257 hypothetical protein | 1 | 0 | 0 | 1 |
| No family | SUFOP01_CHR_02258 hypothetical protein | 1 | 0 | 0 | 1 |
| No family | SUFOP01_CHR_02260 hypothetical protein | 1 | 0 | 0 | 1 |
| No family | SUFOP01_CHR_02261 hypothetical protein | 1 | 0 | 0 | 1 |
| No family | SUFOP01_CHR_02262 hypothetical protein | 1 | 0 | 0 | 1 |
| No family | SUFOP01_CHR_02263 hypothetical protein | 1 | 0 | 0 | 1 |
| No family | SUFOP01_CHR_02265 hypothetical protein | 1 | 0 | 0 | 1 |
| No family | SUFOP01_CHR_02266 hypothetical protein | 1 | 0 | 0 | 1 |
| No family | SUFOP01_CHR_02267 hypothetical protein | 1 | 0 | 0 | 1 |
| No family | SUFOP01_CHR_02268 hypothetical protein | 1 | 0 | 0 | 1 |
| No family | SUFOP01_CHR_02269 hypothetical protein | 1 | 0 | 0 | 1 |
| No family | SUFOP01_CHR_02270 hypothetical protein | 1 | 0 | 0 | 1 |
| No family | SUFOP01_CHR_02271 hypothetical protein | 1 | 0 | 0 | 1 |
| No family | SUFOP01_CHR_02272 hypothetical protein | 1 | 0 | 0 | 1 |
| No family | SUFOP01_CHR_02273 hypothetical protein | 1 | 0 | 0 | 1 |
| No family | SUFOP01_CHR_02274 hypothetical protein | 1 | 0 | 0 | 1 |
| No family | SUFOP01_CHR_02275 hypothetical protein | 1 | 0 | 0 | 1 |
| No family | SUFOP01_CHR_02276 hypothetical protein | 1 | 0 | 0 | 1 |
| No family | SUFOP01_CHR_02277 hypothetical protein | 1 | 0 | 0 | 1 |
| No family | SUFOP01_CHR_02279 hypothetical protein | 1 | 0 | 0 | 1 |
| No family | SUFOP01_CHR_02281 hypothetical protein | 1 | 0 | 0 | 1 |
| No family | SUFOP01_CHR_02283 hypothetical protein | 1 | 0 | 0 | 1 |
| No family | SUFOP01_CHR_02285 hypothetical protein | 1 | 0 | 0 | 1 |
| No family | SUFOP01_CHR_02286 hypothetical protein | 1 | 0 | 0 | 1 |
| No family | SUFOP01_CHR_02293 hypothetical protein | 1 | 0 | 0 | 1 |
| No family | SUFOP01_CHR_02294 hypothetical protein | 1 | 0 | 0 | 1 |
| No family | SUFOP01_CHR_02296 hypothetical protein | 1 | 0 | 0 | 1 |
| No family | SUFOP01_CHR_02298 hypothetical protein | 1 | 0 | 0 | 1 |
| No family | SUFOP01_CHR_02299 hypothetical protein | 1 | 0 | 0 | 1 |
| No family | SUFOP01_CHR_02301 hypothetical protein | 1 | 0 | 0 | 1 |
| No family | SUFOP01_CHR_02302 Exodeoxyribonuclease 10 | 1 | 0 | 0 | 1 |
| No family | SUFOP01_CHR_02303 hypothetical protein | 1 | 0 | 0 | 1 |
| No family | SUFOP01_CHR_02304 hypothetical protein | 1 | 0 | 0 | 1 |
| No family | SUFOP01_CHR_02308 hypothetical protein | 1 | 0 | 0 | 1 |
| No family | SUFOP01_CHR_02309 hypothetical protein | 1 | 0 | 0 | 1 |
| No family | SUFOP01_CHR_02315 hypothetical protein | 1 | 0 | 0 | 1 |
| No family | SUFOP01_CHR_02370 O-acetyltransferase WecH | 1 | 0 | 0 | 1 |
| No family | SUFOP01_CHR_02419 hypothetical protein | 1 | 0 | 0 | 1 |
| No family | SUFOP01_CHR_02420 hypothetical protein | 1 | 0 | 0 | 1 |
| No family | SUFOP01_CHR_02475 hypothetical protein | 1 | 0 | 0 | 1 |
| No family | SUFOP01_CHR_02519 hypothetical protein | 1 | 0 | 0 | 1 |
| No family | SUFOP01_CHR_02528 hypothetical protein | 1 | 0 | 0 | 1 |
| No family | SUFOP01_CHR_02532 hypothetical protein | 1 | 0 | 0 | 1 |
| No family | SUFOP01_CHR_02533 hypothetical protein | 1 | 0 | 0 | 1 |
| No family | SUFOP01_CHR_02534 hypothetical protein | 1 | 0 | 0 | 1 |
| No family | SUFOP01_CHR_02535 hypothetical protein | 1 | 0 | 0 | 1 |
| No family | SUFOP01_CHR_02601 hypothetical protein | 1 | 0 | 0 | 1 |
| No family | SUFOP01_CHR_02603 hypothetical protein | 1 | 0 | 0 | 1 |
| No family | SUFOP01_CHR_02605 hypothetical protein | 1 | 0 | 0 | 1 |
| No family | SUFOP01_CHR_02606 hypothetical protein | 1 | 0 | 0 | 1 |
| No family | SUFOP01_CHR_02608 hypothetical protein | 1 | 0 | 0 | 1 |
| No family | SUFOP01_CHR_02609 hypothetical protein | 1 | 0 | 0 | 1 |
| No family | SUFOP01_CHR_02610 hypothetical protein | 1 | 0 | 0 | 1 |
| No family | SUFOP01_CHR_02611 hypothetical protein | 1 | 0 | 0 | 1 |
| No family | SUFOP01_CHR_02612 hypothetical protein | 1 | 0 | 0 | 1 |
| No family | SUFOP01_CHR_02613 hypothetical protein | 1 | 0 | 0 | 1 |
| No family | SUFOP01_CHR_02615 DNA adenine methylase | 1 | 0 | 0 | 1 |
| No family | SUFOP01_CHR_02618 hypothetical protein | 1 | 0 | 0 | 1 |
| No family | SUFOP01_CHR_02619 hypothetical protein | 1 | 0 | 0 | 1 |
| No family | SUFOP01_CHR_02620 hypothetical protein | 1 | 0 | 0 | 1 |
| No family | SUFOP01_CHR_02621 hypothetical protein | 1 | 0 | 0 | 1 |
| No family | SUFOP01_CHR_02622 hypothetical protein | 1 | 0 | 0 | 1 |
| No family | SUFOP01_CHR_02623 hypothetical protein | 1 | 0 | 0 | 1 |
| No family | SUFOP01_CHR_02624 hypothetical protein | 1 | 0 | 0 | 1 |
| No family | SUFOP01_CHR_02625 hypothetical protein | 1 | 0 | 0 | 1 |
| No family | SUFOP01_CHR_02626 hypothetical protein | 1 | 0 | 0 | 1 |
| No family | SUFOP01_CHR_02627 hypothetical protein | 1 | 0 | 0 | 1 |
| No family | SUFOP01_CHR_02628 hypothetical protein | 1 | 0 | 0 | 1 |
| No family | SUFOP01_CHR_02629 hypothetical protein | 1 | 0 | 0 | 1 |
| No family | SUFOP01_CHR_02630 hypothetical protein | 1 | 0 | 0 | 1 |
| No family | SUFOP01_CHR_02631 hypothetical protein | 1 | 0 | 0 | 1 |
| No family | SUFOP01_CHR_02632 hypothetical protein | 1 | 0 | 0 | 1 |
| No family | SUFOP01_CHR_02633 hypothetical protein | 1 | 0 | 0 | 1 |
| No family | SUFOP01_CHR_02634 hypothetical protein | 1 | 0 | 0 | 1 |
| No family | SUFOP01_CHR_02635 hypothetical protein | 1 | 0 | 0 | 1 |
| No family | SUFOP01_CHR_02636 hypothetical protein | 1 | 0 | 0 | 1 |
| No family | SUFOP01_CHR_02637 hypothetical protein | 1 | 0 | 0 | 1 |
| No family | SUFOP01_CHR_02641 hypothetical protein | 1 | 0 | 0 | 1 |
| No family | SUFOP01_CHR_02643 hypothetical protein | 1 | 0 | 0 | 1 |
| No family | SUFOP01_CHR_02644 hypothetical protein | 1 | 0 | 0 | 1 |
| No family | SUFOP01_CHR_02645 Putative prophage major tail sheath protein | 1 | 0 | 0 | 1 |
| No family | SUFOP01_CHR_02646 hypothetical protein | 1 | 0 | 0 | 1 |
| No family | SUFOP01_CHR_02647 hypothetical protein | 1 | 0 | 0 | 1 |
| No family | SUFOP01_CHR_02757 hypothetical protein | 1 | 0 | 0 | 1 |
| No family | SUFOP01_CHR_02893 hypothetical protein | 1 | 0 | 0 | 1 |
| No family | SUFOP01_CHR_02894 hypothetical protein | 1 | 0 | 0 | 1 |
| No family | SUFOP01_CHR_02895 Putative prophage major tail sheath protein | 1 | 0 | 0 | 1 |
| No family | SUFOP01_CHR_02896 hypothetical protein | 1 | 0 | 0 | 1 |
| No family | SUFOP01_CHR_02898 hypothetical protein | 1 | 0 | 0 | 1 |
| No family | SUFOP01_CHR_02899 hypothetical protein | 1 | 0 | 0 | 1 |
| No family | SUFOP01_CHR_02900 hypothetical protein | 1 | 0 | 0 | 1 |
| No family | SUFOP01_CHR_02904 hypothetical protein | 1 | 0 | 0 | 1 |
| No family | SUFOP01_CHR_02907 hypothetical protein | 1 | 0 | 0 | 1 |
| No family | SUFOP01_CHR_02908 Tyrosine recombinase XerD | 1 | 0 | 0 | 1 |
| No family | SUFOP01_CHR_02910 hypothetical protein | 1 | 0 | 0 | 1 |
| No family | SUFOP01_CHR_02947 D-inositol 3-phosphate glycosyltransferase | 1 | 0 | 0 | 1 |
| No family | SUFOP01_CHR_02961 Glycogen synthase | 1 | 0 | 0 | 1 |
| No family | SUFOP01_CHR_02965 hypothetical protein | 1 | 0 | 0 | 1 |
| No family | SUFOP01_CHR_02967 hypothetical protein | 1 | 0 | 0 | 1 |
| No family | SUFOP01_CHR_02973 hypothetical protein | 1 | 0 | 0 | 1 |
| No family | SUFOP01_CHR_02974 Putative pyruvyl transferase EpsO | 1 | 0 | 0 | 1 |
| No family | SUFOP01_CHR_02985 hypothetical protein | 1 | 0 | 0 | 1 |
| No family | SUFOP01_CHR_03129 hypothetical protein | 1 | 0 | 0 | 1 |
| No family | SUFOP01_CHR_03200 D-lactate dehydrogenase | 1 | 0 | 0 | 1 |
| No family | SUFOP01_CHR_03623 hypothetical protein | 1 | 0 | 0 | 1 |
| No family | SUFOP01_CHR_03625 Glycine cleavage system transcriptional activator | 1 | 0 | 0 | 1 |
| No family | SUFOP01_CHR_03626 hypothetical protein | 1 | 0 | 0 | 1 |
| No family | SUFOP01_CHR_03627 putative quorum-quenching lactonase YtnP | 1 | 0 | 0 | 1 |
| No family | SUFOP01_CHR_03656 Uric acid transporter UacT | 1 | 0 | 0 | 1 |
| No family | SUFOP01_CHR_03744 Adenosine monophosphate-protein transferase NmFic | 1 | 0 | 0 | 1 |
| No family | SUFOP01_CHR_03745 hypothetical protein | 1 | 0 | 0 | 1 |
| No family | SUFOP01_CHR_03746 hypothetical protein | 1 | 0 | 0 | 1 |
| No family | SUFOP01_CHR_03747 DNA replication and repair protein RecF | 1 | 0 | 0 | 1 |
| No family | SUFOP01_CHR_03750 hypothetical protein | 1 | 0 | 0 | 1 |
| No family | SUFOP01_CHR_03751 hypothetical protein | 1 | 0 | 0 | 1 |
| No family | SUFOP01_CHR_03752 hypothetical protein | 1 | 0 | 0 | 1 |
| No family | SUFOP01_CHR_03753 hypothetical protein | 1 | 0 | 0 | 1 |
| No family | SUFOP01_CHR_03754 Tyrosine recombinase XerC | 1 | 0 | 0 | 1 |
| No family | SUFOP01_CHR_03755 hypothetical protein | 1 | 0 | 0 | 1 |
| No family | SUFOP01_CHR_03756 hypothetical protein | 1 | 0 | 0 | 1 |
| No family | SUFOP01_CHR_03757 hypothetical protein | 1 | 0 | 0 | 1 |
| No family | SUFOP01_CHR_03758 hypothetical protein | 1 | 0 | 0 | 1 |
| No family | SUFOP01_CHR_03759 hypothetical protein | 1 | 0 | 0 | 1 |
| No family | SUFOP01_CHR_03760 hypothetical protein | 1 | 0 | 0 | 1 |
| No family | SUFOP01_CHR_03761 hypothetical protein | 1 | 0 | 0 | 1 |
| No family | SUFOP01_CHR_03762 hypothetical protein | 1 | 0 | 0 | 1 |
| No family | SUFOP01_CHR_03763 hypothetical protein | 1 | 0 | 0 | 1 |
| No family | SUFOP01_CHR_03764 hypothetical protein | 1 | 0 | 0 | 1 |
| No family | SUFOP01_CHR_03765 hypothetical protein | 1 | 0 | 0 | 1 |
| No family | SUFOP01_CHR_03766 Prophage CP4-57 integrase | 1 | 0 | 0 | 1 |
| No family | SUFOP01_CHR_03820 mRNA interferase YafO | 1 | 0 | 0 | 1 |
| No family | SUFOP01_CHR_03821 hypothetical protein | 1 | 0 | 0 | 1 |
| No family | SUFOP01_CHR_03827 hypothetical protein | 1 | 0 | 0 | 1 |
| No family | SUFOP01_CHR_03847 hypothetical protein | 1 | 0 | 0 | 1 |
| No family | SUFOP01_CHR_03852 hypothetical protein | 1 | 0 | 0 | 1 |
| No family | SUFOP01_CHR_03856 hypothetical protein | 1 | 0 | 0 | 1 |
| No family | SUFOP01_CHR_03974 hypothetical protein | 1 | 0 | 0 | 1 |
| No family | SUFOP01_CHR_03977 hypothetical protein | 1 | 0 | 0 | 1 |
| No family | SUFOP01_CHR_03979 hypothetical protein | 1 | 0 | 0 | 1 |
| No family | SUFOP01_CHR_03980 hypothetical protein | 1 | 0 | 0 | 1 |
| No family | SUFOP01_CHR_03982 hypothetical protein | 1 | 0 | 0 | 1 |
| No family | SUFOP01_CHR_03983 hypothetical protein | 1 | 0 | 0 | 1 |
| No family | SUFOP01_CHR_03987 hypothetical protein | 1 | 0 | 0 | 1 |
| No family | SUFOP01_CHR_03991 hypothetical protein | 1 | 0 | 0 | 1 |
| No family | SUFOP01_CHR_03993 hypothetical protein | 1 | 0 | 0 | 1 |
| No family | SUFOP01_CHR_03999 hypothetical protein | 1 | 0 | 0 | 1 |
| No family | SUFOP01_CHR_04011 hypothetical protein | 1 | 0 | 0 | 1 |
| No family | SUFOP01_CHR_04016 hypothetical protein | 1 | 0 | 0 | 1 |
| No family | SUFOP01_CHR_04017 hypothetical protein | 1 | 0 | 0 | 1 |
| No family | SUFOP01_CHR_04105 Ferredoxin | 1 | 0 | 0 | 1 |
| No family | SUFOP01_CHR_04106 hypothetical protein | 1 | 0 | 0 | 1 |
| No family | SUFOP01_CHR_04107 Linear gramicidin synthase subunit D | 1 | 0 | 0 | 1 |
| No family | SUFOP01_CHR_04108 Kynurenine formamidase | 1 | 0 | 0 | 1 |
| No family | SUFOP01_CHR_04109 Tryptophan 2,3-dioxygenase | 1 | 0 | 0 | 1 |
| No family | SUFOP01_CHR_04118 hypothetical protein | 1 | 0 | 0 | 1 |
| No family | SUFOP01_CHR_04119 hypothetical protein | 1 | 0 | 0 | 1 |
| No family | SUFOP01_CHR_04120 hypothetical protein | 1 | 0 | 0 | 1 |
| No family | SUFOP01_CHR_04122 hypothetical protein | 1 | 0 | 0 | 1 |
| No family | SUFOP01_CHR_04123 Toxin YkfI | 1 | 0 | 0 | 1 |
| No family | SUFOP01_CHR_04124 Cytoskeleton bundling-enhancing protein CbeA | 1 | 0 | 0 | 1 |
| No family | SUFOP01_CHR_04125 hypothetical protein | 1 | 0 | 0 | 1 |
| No family | SUFOP01_CHR_04126 hypothetical protein | 1 | 0 | 0 | 1 |
| No family | SUFOP01_CHR_04128 hypothetical protein | 1 | 0 | 0 | 1 |
| No family | SUFOP01_CHR_04131 hypothetical protein | 1 | 0 | 0 | 1 |
| No family | SUFOP01_CHR_04133 hypothetical protein | 1 | 0 | 0 | 1 |
| No family | SUFOP01_CHR_04137 hypothetical protein | 1 | 0 | 0 | 1 |
| No family | SUFOP01_CHR_04138 hypothetical protein | 1 | 0 | 0 | 1 |
| No family | SUFOP01_CHR_04139 hypothetical protein | 1 | 0 | 0 | 1 |
| No family | SUFOP01_CHR_04140 hypothetical protein | 1 | 0 | 0 | 1 |
| No family | SUFOP01_CHR_04141 hypothetical protein | 1 | 0 | 0 | 1 |
| No family | SUFOP01_CHR_04142 hypothetical protein | 1 | 0 | 0 | 1 |
| No family | SUFOP01_CHR_04143 hypothetical protein | 1 | 0 | 0 | 1 |
| No family | SUFOP01_CHR_04178 Colicin-D immunity protein | 1 | 0 | 0 | 1 |
| No family | SUFOP01_CHR_04500 Porin B | 1 | 0 | 0 | 1 |
| No family | SUFOP01_CHR_04526 hypothetical protein | 1 | 0 | 0 | 1 |
| No family | SUFOP01_CHR_04538 Immunity protein CdiI | 1 | 0 | 0 | 1 |
| No family | SUFOP01_CHR_04539 DNase CdiA | 1 | 0 | 0 | 1 |
| No family | SUFOP01_CHR_04540 hypothetical protein | 1 | 0 | 0 | 1 |
| No family | SUFOP01_CHR_04541 hypothetical protein | 1 | 0 | 0 | 1 |
| No family | SUFOP01_CHR_04542 hypothetical protein | 1 | 0 | 0 | 1 |
| No family | SUFOP01_CHR_04543 hypothetical protein | 1 | 0 | 0 | 1 |
| No family | SUFOP01_CHR_04544 hypothetical protein | 1 | 0 | 0 | 1 |
| No family | SUFOP01_CHR_04689 ATP-dependent Clp protease ATP-binding subunit ClpX | 1 | 0 | 0 | 1 |
| No family | SUFOP01_CHR_04690 hypothetical protein | 1 | 0 | 0 | 1 |
| No family | SUFOP01_CHR_04692 hypothetical protein | 1 | 0 | 0 | 1 |
| No family | SUFOP01_CHR_04693 hypothetical protein | 1 | 0 | 0 | 1 |
| No family | SUFOP01_CHR_04694 hypothetical protein | 1 | 0 | 0 | 1 |
| No family | SUFOP01_CHR_04695 Fertility inhibition protein | 1 | 0 | 0 | 1 |
| No family | SUFOP01_CHR_04696 Single-stranded DNA-binding protein | 1 | 0 | 0 | 1 |
| No family | SUFOP01_CHR_04697 hypothetical protein | 1 | 0 | 0 | 1 |
| No family | SUFOP01_CHR_04699 hypothetical protein | 1 | 0 | 0 | 1 |
| No family | SUFOP01_CHR_04700 hypothetical protein | 1 | 0 | 0 | 1 |
| No family | SUFOP01_CHR_04701 hypothetical protein | 1 | 0 | 0 | 1 |
| No family | SUFOP01_CHR_04702 hypothetical protein | 1 | 0 | 0 | 1 |
| No family | SUFOP01_CHR_04703 hypothetical protein | 1 | 0 | 0 | 1 |
| No family | SUFOP01_CHR_04704 hypothetical protein | 1 | 0 | 0 | 1 |
| No family | SUFOP01_CHR_04705 hypothetical protein | 1 | 0 | 0 | 1 |
| No family | SUFOP01_CHR_04706 hypothetical protein | 1 | 0 | 0 | 1 |
| No family | SUFOP01_CHR_04707 hypothetical protein | 1 | 0 | 0 | 1 |
| No family | SUFOP01_CHR_04708 Prophage CP4-57 integrase | 1 | 0 | 0 | 1 |
| No family | SUFOP01_CHR_05094 Tyrocidine synthase 3 | 1 | 0 | 0 | 1 |
| No family | SUFOP01_CHR_05097 Transcription antitermination protein RfaH | 1 | 0 | 0 | 1 |
| No family | SUFOP01_CHR_05099 Glutamate decarboxylase alpha | 1 | 0 | 0 | 1 |
| No family | SUFOP01_CHR_05100 putative transporter | 1 | 0 | 0 | 1 |
| No family | SUFOP01_CHR_05111 hypothetical protein | 1 | 0 | 0 | 1 |
| No family | SUFOP01_CHR_05124 hypothetical protein | 1 | 0 | 0 | 1 |
| No family | SUFOP01_CHR_05126 hypothetical protein | 1 | 0 | 0 | 1 |
| No family | SUFOP01_CHR_05224 hypothetical protein | 1 | 0 | 0 | 1 |
| No family | SUFOP01_CHR_05233 hypothetical protein | 1 | 0 | 0 | 1 |
| No family | SUFOP01_CHR_05364 hypothetical protein | 1 | 0 | 0 | 1 |
| No family | SUFOP01_CHR_05365 hypothetical protein | 1 | 0 | 0 | 1 |
| No family | SUFOP01_CHR_05366 Transcriptional regulatory protein RcsB | 1 | 0 | 0 | 1 |
| No family | SUFOP01_CHR_05375 hypothetical protein | 1 | 0 | 0 | 1 |
| No family | SUFOP01_P1_00004 hypothetical protein | 0 | 1 | 0 | 1 |
| No family | SUFOP01_P1_00007 hypothetical protein | 0 | 1 | 0 | 1 |
| No family | SUFOP01_P1_00011 hypothetical protein | 0 | 1 | 0 | 1 |
| No family | SUFOP01_P1_00016 hypothetical protein | 0 | 1 | 0 | 1 |
| No family | SUFOP01_P1_00021 hypothetical protein | 0 | 1 | 0 | 1 |
| No family | SUFOP01_P1_00027 hypothetical protein | 0 | 1 | 0 | 1 |
| No family | SUFOP01_P1_00033 7-cyano-7-deazaguanine synthase | 0 | 1 | 0 | 1 |
| No family | SUFOP01_P1_00034 hypothetical protein | 0 | 1 | 0 | 1 |
| No family | SUFOP01_P1_00035 hypothetical protein | 0 | 1 | 0 | 1 |
| No family | SUFOP01_P1_00036 hypothetical protein | 0 | 1 | 0 | 1 |
| No family | SUFOP01_P1_00047 hypothetical protein | 0 | 1 | 0 | 1 |
| No family | SUFOP01_P1_00057 hypothetical protein | 0 | 1 | 0 | 1 |
| No family | SUFOP01_P1_00061 hypothetical protein | 0 | 1 | 0 | 1 |
| No family | SUFOP01_P1_00062 hypothetical protein | 0 | 1 | 0 | 1 |
| No family | SUFOP01_P1_00063 hypothetical protein | 0 | 1 | 0 | 1 |
| No family | SUFOP01_P1_00064 hypothetical protein | 0 | 1 | 0 | 1 |
| No family | SUFOP01_P1_00065 hypothetical protein | 0 | 1 | 0 | 1 |
| No family | SUFOP01_P1_00066 putative chromosome-partitioning protein ParB | 0 | 1 | 0 | 1 |
| No family | SUFOP01_P1_00067 Chromosome-partitioning ATPase Soj | 0 | 1 | 0 | 1 |
| No family | SUFOP01_P1_00068 hypothetical protein | 0 | 1 | 0 | 1 |
| No family | SUFOP01_P1_00069 hypothetical protein | 0 | 1 | 0 | 1 |
| No family | SUFOP01_P1_00070 hypothetical protein | 0 | 1 | 0 | 1 |
| No family | SUFOP01_P1_00071 hypothetical protein | 0 | 1 | 0 | 1 |
| No family | SUFOP01_P1_00073 hypothetical protein | 0 | 1 | 0 | 1 |
| No family | SUFOP01_P1_00074 hypothetical protein | 0 | 1 | 0 | 1 |
| No family | SUFOP01_P1_00075 hypothetical protein | 0 | 1 | 0 | 1 |
| No family | SUFOP01_P1_00077 hypothetical protein | 0 | 1 | 0 | 1 |
| No family | SUFOP01_P1_00078 hypothetical protein | 0 | 1 | 0 | 1 |
| No family | SUFOP01_P1_00080 hypothetical protein | 0 | 1 | 0 | 1 |
| No family | SUFOP01_P1_00081 Transcriptional repressor FrmR | 0 | 1 | 0 | 1 |
| No family | SUFOP01_P1_00083 hypothetical protein | 0 | 1 | 0 | 1 |
| No family | SUFOP01_P1_00086 hypothetical protein | 0 | 1 | 0 | 1 |
| No family | SUFOP01_P1_00089 hypothetical protein | 0 | 1 | 0 | 1 |
| No family | SUFOP01_P1_00092 hypothetical protein | 0 | 1 | 0 | 1 |
| No family | SUFOP01_P1_00093 hypothetical protein | 0 | 1 | 0 | 1 |
| No family | SUFOP01_P1_00096 hypothetical protein | 0 | 1 | 0 | 1 |
| No family | SUFOP01_P1_00098 Toxin ParE1 | 0 | 1 | 0 | 1 |
| No family | SUFOP01_P1_00101 hypothetical protein | 0 | 1 | 0 | 1 |
| No family | SUFOP01_P1_00113 hypothetical protein | 0 | 1 | 0 | 1 |
| No family | SUFOP01_P1_00114 Non-hemolytic phospholipase C | 0 | 1 | 0 | 1 |
| No family | SUFOP01_P1_00115 hypothetical protein | 0 | 1 | 0 | 1 |
| No family | SUFOP01_P1_00116 hypothetical protein | 0 | 1 | 0 | 1 |
| No family | SUFOP01_P1_00118 Antitoxin VapB | 0 | 1 | 0 | 1 |
| No family | SUFOP01_P1_00119 tRNA(fMet)-specific endonuclease VapC | 0 | 1 | 0 | 1 |
| No family | SUFOP01_P1_00120 hypothetical protein | 0 | 1 | 0 | 1 |
| No family | SUFOP01_P1_00121 hypothetical protein | 0 | 1 | 0 | 1 |
| No family | SUFOP01_P1_00123 hypothetical protein | 0 | 1 | 0 | 1 |
| No family | SUFOP01_P1_00125 hypothetical protein | 0 | 1 | 0 | 1 |
| No family | SUFOP01_P1_00132 hypothetical protein | 0 | 1 | 0 | 1 |
| No family | SUFOP01_P1_00134 Mono(ADP-ribosyl)transferase SpvB | 0 | 1 | 0 | 1 |
| No family | SUFOP01_P1_00135 hypothetical protein | 0 | 1 | 0 | 1 |
| No family | SUFOP01_P1_00138 hypothetical protein | 0 | 1 | 0 | 1 |
| No family | SUFOP01_P1_00141 hypothetical protein | 0 | 1 | 0 | 1 |
| No family | SUFOP01_P1_00152 Heat shock protein C | 0 | 1 | 0 | 1 |
| No family | SUFOP01_P1_00153 hypothetical protein | 0 | 1 | 0 | 1 |
| No family | SUFOP01_P1_00157 hypothetical protein | 0 | 1 | 0 | 1 |
| No family | SUFOP01_P1_00159 hypothetical protein | 0 | 1 | 0 | 1 |
| No family | SUFOP01_P1_00168 Group II intron-encoded protein LtrA | 0 | 1 | 0 | 1 |
| No family | SUFOP01_P1_00170 hypothetical protein | 0 | 1 | 0 | 1 |
| No family | SUFOP01_P1_00172 hypothetical protein | 0 | 1 | 0 | 1 |
| No family | SUFOP01_P1_00173 hypothetical protein | 0 | 1 | 0 | 1 |
| No family | SUFOP01_P2_00003 hypothetical protein | 0 | 0 | 1 | 1 |
| No family | SUFOP01_P2_00005 hypothetical protein | 0 | 0 | 1 | 1 |
| No family | SUFOP01_P2_00012 hypothetical protein | 0 | 0 | 1 | 1 |
| No family | SUFOP01_P2_00016 hypothetical protein | 0 | 0 | 1 | 1 |
| No family | SUFOP01_P2_00017 hypothetical protein | 0 | 0 | 1 | 1 |
| No family | SUFOP01_P2_00018 hypothetical protein | 0 | 0 | 1 | 1 |
| No family | SUFOP01_P2_00024 hypothetical protein | 0 | 0 | 1 | 1 |
| No family | SUFOP01_P2_00025 hypothetical protein | 0 | 0 | 1 | 1 |
| No family | SUFOP01_P2_00032 hypothetical protein | 0 | 0 | 1 | 1 |
| No family | SUFOP01_P2_00051 Relaxosome protein TraY | 0 | 0 | 1 | 1 |
| No family | SUFOP01_P2_00052 Relaxosome protein TraY | 0 | 0 | 1 | 1 |
| No family | SUFOP01_P2_00058 hypothetical protein | 0 | 0 | 1 | 1 |
| No family | SUFOP01_P2_00061 hypothetical protein | 0 | 0 | 1 | 1 |
| No family | SUFOP01_P2_00062 hypothetical protein | 0 | 0 | 1 | 1 |
| No family | SUFOP01_P2_00063 hypothetical protein | 0 | 0 | 1 | 1 |
| No family | SUFOP01_P2_00082 hypothetical protein | 0 | 0 | 1 | 1 |
| No family | SUFOP01_P2_00084 hypothetical protein | 0 | 0 | 1 | 1 |
| No family | SUFOP01_P2_00088 Superoxide dismutase [Cu-Zn] | 0 | 0 | 1 | 1 |
| No family | SUFOP01_P2_00090 hypothetical protein | 0 | 0 | 1 | 1 |
| No family | SUFOP01_P2_00091 Inhibitor of vertebrate lysozyme | 0 | 0 | 1 | 1 |
| No family | SUFOP01_P2_00096 S-fimbrial protein subunit SfaG | 0 | 0 | 1 | 1 |
| No family | SUFOP01_P2_00099 Type-1 fimbrial protein, A chain | 0 | 0 | 1 | 1 |
| No family | SUFOP01_P2_00100 Fimbrial subunit type 1 | 0 | 0 | 1 | 1 |
| No family | SUFOP01_P2_00101 hypothetical protein | 0 | 0 | 1 | 1 |
| No family | SUFOP01_P2_00102 HTH-type transcriptional regulator PgrR | 0 | 0 | 1 | 1 |
| No family | SUFOP01_P2_00104 hypothetical protein | 0 | 0 | 1 | 1 |
| No family | SUFOP01_P2_00109 hypothetical protein | 0 | 0 | 1 | 1 |
| No family | SUFOP01_P2_00110 hypothetical protein | 0 | 0 | 1 | 1 |
| No family | SUFOP01_P2_00113 hypothetical protein | 0 | 0 | 1 | 1 |
| No family | SUFOP01_P2_00116 Multiple stress resistance protein BhsA | 0 | 0 | 1 | 1 |
| No family | SUFOP01_P2_00131 hypothetical protein | 0 | 0 | 1 | 1 |
| No family | SUFOP01_P2_00132 hypothetical protein | 0 | 0 | 1 | 1 |
| No family | SUFOP01_P2_00133 hypothetical protein | 0 | 0 | 1 | 1 |
| No family | SUFOP01_P2_00134 hypothetical protein | 0 | 0 | 1 | 1 |
| No family | SUFOP01_P2_00135 hypothetical protein | 0 | 0 | 1 | 1 |
| No family | SUFOP01_P2_00136 hypothetical protein | 0 | 0 | 1 | 1 |
| No family | SUFOP01_P2_00139 LexA repressor | 0 | 0 | 1 | 1 |
| No family | SUFOP01_P2_00140 Protein RecA | 0 | 0 | 1 | 1 |
| No family | SUFOP01_P2_00141 hypothetical protein | 0 | 0 | 1 | 1 |
| No family | SUFOP01_P2_00142 hypothetical protein | 0 | 0 | 1 | 1 |
| No family | SUFOP01_P2_00143 hypothetical protein | 0 | 0 | 1 | 1 |
| No family | SUFOP01_P2_00144 hypothetical protein | 0 | 0 | 1 | 1 |
| No family | SUFOP01_P2_00145 hypothetical protein | 0 | 0 | 1 | 1 |
| No family | SUFOP01_P2_00146 hypothetical protein | 0 | 0 | 1 | 1 |
|  | TOTAL | 411 | 61 | 44 | 516 |
| **Family** | **Description (product)** | **[S_ufop_01_chr]** | **[S_ufop_01_p1]** | **[S_ufop_01_p2]** | **total** |
| 1 | hypothetical protein | 0 | 19 | 0 | 19 |
| 118 | hypothetical protein | 0 | 3 | 2 | 5 |
| 119 | hypothetical protein | 5 | 0 | 0 | 5 |
| 120 | hypothetical protein | 5 | 0 | 0 | 5 |
| 121 | hypothetical protein | 5 | 0 | 0 | 5 |
| 122 | hypothetical protein | 5 | 0 | 0 | 5 |
| 4032 | hypothetical protein | 0 | 3 | 1 | 4 |
| 4033 | hypothetical protein | 0 | 1 | 3 | 4 |
| 4034 | hypothetical protein | 4 | 0 | 0 | 4 |
| 4035 | hypothetical protein | 4 | 0 | 0 | 4 |
| 4036 | Thioredoxin-dependent 5-adenylylsulfate reductase | 4 | 0 | 0 | 4 |
| 4037 | hypothetical protein | 4 | 0 | 0 | 4 |
| 4038 | hypothetical protein | 4 | 0 | 0 | 4 |
| 4039 | hypothetical protein | 4 | 0 | 0 | 4 |
| 4040 | hypothetical protein | 4 | 0 | 0 | 4 |
| 4041 | hypothetical protein | 4 | 0 | 0 | 4 |
| 4042 | hypothetical protein | 4 | 0 | 0 | 4 |
| 4043 | hypothetical protein | 4 | 0 | 0 | 4 |
| 4044 | hypothetical protein | 4 | 0 | 0 | 4 |
| 4045 | hypothetical protein | 4 | 0 | 0 | 4 |
| 4046 | hypothetical protein | 4 | 0 | 0 | 4 |
| 4047 | hypothetical protein | 4 | 0 | 0 | 4 |
| 4048 | hypothetical protein | 4 | 0 | 0 | 4 |
| 4049 | hypothetical protein | 4 | 0 | 0 | 4 |
| 4050 | hypothetical protein | 4 | 0 | 0 | 4 |
| 4051 | hypothetical protein | 4 | 0 | 0 | 4 |
| 4052 | hypothetical protein | 4 | 0 | 0 | 4 |
| 4053 | hypothetical protein | 4 | 0 | 0 | 4 |
| 4054 | hypothetical protein | 4 | 0 | 0 | 4 |
| 4055 | hypothetical protein | 4 | 0 | 0 | 4 |
| 4056 | hypothetical protein | 4 | 0 | 0 | 4 |
| 4057 | hypothetical protein | 4 | 0 | 0 | 4 |
| 4439 | hypothetical protein | 0 | 3 | 0 | 3 |
| 4440 | hypothetical protein | 0 | 1 | 2 | 3 |
| 4441 | hypothetical protein | 0 | 1 | 2 | 3 |
| 4442 | hypothetical protein | 0 | 1 | 2 | 3 |
| 4443 | hypothetical protein | 0 | 1 | 2 | 3 |
| 4444 | hypothetical protein | 1 | 1 | 1 | 3 |
| 4445 | hypothetical protein | 3 | 0 | 0 | 3 |
| 4446 | hypothetical protein | 2 | 1 | 0 | 3 |
| 4447 | hypothetical protein | 3 | 0 | 0 | 3 |
| 4448 | hypothetical protein | 3 | 0 | 0 | 3 |
| 4449 | Tyrosine recombinase XerD | 3 | 0 | 0 | 3 |
| 4694 | hypothetical protein | 0 | 1 | 1 | 2 |
| 4695 | Chromosome-partitioning ATPase Soj | 0 | 1 | 1 | 2 |
| 4696 | hypothetical protein | 0 | 1 | 1 | 2 |
| 4697 | hypothetical protein | 0 | 1 | 1 | 2 |
| 4698 | hypothetical protein | 0 | 1 | 1 | 2 |
| 4699 | Sporulation initiation inhibitor protein Soj | 0 | 1 | 1 | 2 |
| 4700 | hypothetical protein | 0 | 2 | 0 | 2 |
| 4701 | hypothetical protein | 0 | 1 | 1 | 2 |
| 4702 | hypothetical protein | 0 | 1 | 1 | 2 |
| 4703 | Carbonic anhydrase | 0 | 1 | 1 | 2 |
| 4704 | hypothetical protein | 0 | 1 | 1 | 2 |
| 4705 | hypothetical protein | 0 | 1 | 1 | 2 |
| 4706 | hypothetical protein | 0 | 2 | 0 | 2 |
| 4707 | hypothetical protein | 0 | 1 | 1 | 2 |
| 4708 | Antirestriction protein KlcA | 0 | 1 | 1 | 2 |
| 4709 | mRNA interferase RelE | 0 | 1 | 1 | 2 |
| 4710 | hypothetical protein | 0 | 1 | 1 | 2 |
| 4711 | hypothetical protein | 0 | 1 | 1 | 2 |
| 4712 | hypothetical protein | 0 | 1 | 1 | 2 |
| 4713 | hypothetical protein | 0 | 1 | 1 | 2 |
| 4714 | hypothetical protein | 0 | 1 | 1 | 2 |
| 4715 | hypothetical protein | 0 | 1 | 1 | 2 |
| 4716 | Relaxosome protein TraM | 0 | 1 | 1 | 2 |
| 4717 | Pilin | 0 | 1 | 1 | 2 |
| 4718 | hypothetical protein | 0 | 1 | 1 | 2 |
| 4719 | hypothetical protein | 0 | 1 | 1 | 2 |
| 4720 | hypothetical protein | 0 | 1 | 1 | 2 |
| 4721 | hypothetical protein | 0 | 1 | 1 | 2 |
| 4722 | hypothetical protein | 0 | 1 | 1 | 2 |
| 4723 | hypothetical protein | 0 | 1 | 1 | 2 |
| 4724 | hypothetical protein | 0 | 1 | 1 | 2 |
| 4725 | hypothetical protein | 0 | 1 | 1 | 2 |
| 4726 | hypothetical protein | 0 | 1 | 1 | 2 |
| 4727 | hypothetical protein | 0 | 1 | 1 | 2 |
| 4728 | hypothetical protein | 0 | 1 | 1 | 2 |
| 4729 | hypothetical protein | 0 | 1 | 1 | 2 |
| 4730 | hypothetical protein | 0 | 1 | 1 | 2 |
| 4731 | hypothetical protein | 0 | 1 | 1 | 2 |
| 4732 | hypothetical protein | 0 | 1 | 1 | 2 |
| 4733 | hypothetical protein | 0 | 1 | 1 | 2 |
| 4734 | hypothetical protein | 0 | 1 | 1 | 2 |
| 4735 | hypothetical protein | 0 | 1 | 1 | 2 |
| 4736 | Coupling protein TraD | 0 | 1 | 1 | 2 |
| 4737 | Multifunctional conjugation protein TraI | 0 | 1 | 1 | 2 |
| 4738 | Phospholipase D | 0 | 1 | 1 | 2 |
| 4739 | hypothetical protein | 0 | 1 | 1 | 2 |
| 4740 | hypothetical protein | 0 | 1 | 1 | 2 |
| 4741 | hypothetical protein | 2 | 0 | 0 | 2 |
| 4742 | hypothetical protein | 2 | 0 | 0 | 2 |
| 4743 | hypothetical protein | 2 | 0 | 0 | 2 |
| 4744 | hypothetical protein | 2 | 0 | 0 | 2 |
| 4745 | hypothetical protein | 2 | 0 | 0 | 2 |
| 4746 | Lysozyme RrrD | 2 | 0 | 0 | 2 |
| 4747 | hypothetical protein | 2 | 0 | 0 | 2 |
| 4748 | hypothetical protein | 2 | 0 | 0 | 2 |
| 4749 | hypothetical protein | 2 | 0 | 0 | 2 |
| 4750 | hypothetical protein | 2 | 0 | 0 | 2 |
| 4751 | hypothetical protein | 2 | 0 | 0 | 2 |
| 4752 | hypothetical protein | 2 | 0 | 0 | 2 |
| 4753 | hypothetical protein | 2 | 0 | 0 | 2 |
| 4754 | hypothetical protein | 2 | 0 | 0 | 2 |
| 4755 | hypothetical protein | 2 | 0 | 0 | 2 |
| 4756 | hypothetical protein | 2 | 0 | 0 | 2 |
| 4757 | hypothetical protein | 2 | 0 | 0 | 2 |
| 4758 | hypothetical protein | 2 | 0 | 0 | 2 |
| 4759 | hypothetical protein | 2 | 0 | 0 | 2 |
| 4760 | hypothetical protein | 2 | 0 | 0 | 2 |
| 4761 | hypothetical protein | 2 | 0 | 0 | 2 |
| 4762 | hypothetical protein | 2 | 0 | 0 | 2 |
| 4763 | hypothetical protein | 2 | 0 | 0 | 2 |
| 4764 | hypothetical protein | 2 | 0 | 0 | 2 |
| 4765 | hypothetical protein | 2 | 0 | 0 | 2 |
| 4766 | hypothetical protein | 2 | 0 | 0 | 2 |
| 4767 | hypothetical protein | 2 | 0 | 0 | 2 |
| 4768 | hypothetical protein | 2 | 0 | 0 | 2 |
| 4769 | hypothetical protein | 2 | 0 | 0 | 2 |
| 4770 | DNA adenine methylase | 2 | 0 | 0 | 2 |
| 4771 | hypothetical protein | 2 | 0 | 0 | 2 |
| 4772 | hypothetical protein | 2 | 0 | 0 | 2 |
| 4773 | hypothetical protein | 2 | 0 | 0 | 2 |
| 4774 | hypothetical protein | 2 | 0 | 0 | 2 |
| 4775 | hypothetical protein | 2 | 0 | 0 | 2 |
| 4776 | hypothetical protein | 2 | 0 | 0 | 2 |
| 4777 | hypothetical protein | 2 | 0 | 0 | 2 |
|  | **TOTAL** | **205** | **84** | **60** | **349** |

**Supplementary Table 7.** General features of the putative SlFG3 genomic islands.

| **HGT** | **Genome location** | **Start** | **End** | **Genes** | **Unique** | **Phage** | **Hyp** | **tRNA** | **Int.** | **Rec.** | **Toxin / Antitoxin** | **Adaptation and Virulence** |
| --- | --- | --- | --- | --- | --- | --- | --- | --- | --- | --- | --- | --- |
| 1 | 158124-182328 | 146 | 161 | 16 | 6 | 2 | 11 | UP | UP | UP | AntA/AntB | - |
| 2 | 395013-426867 | 345 | 387 | 43 | 27 | 14 | 25 | DOWN | UP | - | - | Methyl-directed Repair DNA adenine methylase |
| 3 | 537838-578350 | 482 | 527 | 46 | 34 | 19 | 18 | - | UP | - | - | RecT |
| 4 | 671907-701030 | 612 | 648 | 37 | 27 | 15 | 19 | - | UP | - | - | Membrane protein related to metalloendopeptidases |
| 5 | 1004064-1047616 | 926 | 967 | 42 | 23 | 24 | 12 | - | UP | - | - | Virulence protein msgA |
| 6 | 1146868-1189430 | 1055 | 1112 | 58 | 46 | 23 | 34 | DOWN | DOWN | - | - | - |
| 7 | 1317790-1362967 | 1235 | 1284 | 50 | 33 | 22 | 28 | - | UP | - |  | RecT |
| 8 | 2349558-2396745 | 2229 | 2282 | 54 | 35 | 16 | 30 | UP | DOWN | - | - | - |
| 9 | 2606518-2642392 | 2484 | 2524 | 41 | 6 | 28 | 11 | - | DOWN | - | - | Methyl-directed Repair DNA adenine methylase; Lead, cadmium, zinc and mercury transporting ATPase; Copper translocating P-type ATPase |
| 10 | 2675865-2711912 | 2561 | 2609 | 47 | 20 | 24 | 21 | - | UP | - | - | Methyl-directed Repair DNA adenine methylase |
| 11 | 2972201-2987936 | 2856 | 2875 | 20 | 7 | 8 | 4 | - | DOWN | UP/DOWN | - | Mobile element protein (4 copies) |
| 12 | 3880691-3901244 | 3719 | 3733 | 15 | 10 | 1 | 9 | DOWN | UP/DOWN | - | - | RadC |
| 13 | 3963850-3995221 | 3788 | 3826 | 39 | 28 | 16 | 21 | - | DOWN | - | - | Membrane protein related to metalloendopeptidases |
| 14 | 4127273-4168305 | 3941 | 3991 | 51 | 31 | 26 | 16 | - | DOWN | - |  | Virulence protein msgA |
| 15 | 4273216-4299766 | 4089 | 4114 | 26 | 17 | 1 | 12 | - | DOWN | - | YpjF / YfjZ | RadC/DndBCDE |
| 16 | 4727001-4760449 | 4500 | 4525 | 26 | 6 | 4 | 12 | - | DOWN | - | YeeU / YeeV and YfjZ / YpjF | RadC |
| 17 | 4929795-4941350 | 4676 | 4692 | 17 | 12 | 3 | 12 | - | DOWN | - | Doc / Phd | - |
| 18 | 5514043-5546006 | 5211 | 5253 | 43 | 30 | 18 | 19 | - | UP | - | - | Methyl-directed Repair DNA adenine methylase |
| Total |  |  |  | 671 | 398 | 264 | 314 |  |  |  |  |  |

Start – initial locus tag; stop – final locus tag; Phage – phage genes; Hyp. – Hypothetical genes; tRNA – tRNA genes; Int. integrase genes; Rec. Recombinase genes; Up – Upstream; Down – Downstream.

**Supplementary Table 8.** General features of the gene cluster of secondary metabolism found in the SlFG3 genome.

| **Cluster** | **Type** | **From** | **To** | **Most similar known cluster**  **(biosynthetic cluster)** | **% genes show similarity** | **MIBiG BGC-ID** |
| --- | --- | --- | --- | --- | --- | --- |
| [1](https://antismash.secondarymetabolites.org/upload/bacteria-2201b9dc-1bbf-4068-9076-40d6da61a07c/index.html#cluster-1) | [Cf_putative](http://antismash.secondarymetabolites.org/help#cf_putative) | 69531 | 80210 | PM100117/PM100118 | 21 | [BGC0001359_c1](http://mibig.secondarymetabolites.org/repository/BGC0001359/index.html) |
| [2](https://antismash.secondarymetabolites.org/upload/bacteria-2201b9dc-1bbf-4068-9076-40d6da61a07c/index.html#cluster-2) | [Nrps](http://antismash.secondarymetabolites.org/help#nrps) | 774158 | 826468 | Turnerbactin | 30 | [BGC0000451_c1](http://mibig.secondarymetabolites.org/repository/BGC0000451/index.html) |
| [3](https://antismash.secondarymetabolites.org/upload/bacteria-2201b9dc-1bbf-4068-9076-40d6da61a07c/index.html#cluster-3) | [Cf_fatty_acid](http://antismash.secondarymetabolites.org/help#cf_fatty_acid%20) | 854967 | 876181 | - |  | - |
| [4](https://antismash.secondarymetabolites.org/upload/bacteria-2201b9dc-1bbf-4068-9076-40d6da61a07c/index.html#cluster-4) | [Cf_putative](http://antismash.secondarymetabolites.org/help#cf_putative) | 946321 | 951799 | - |  | - |
| [5](https://antismash.secondarymetabolites.org/upload/bacteria-2201b9dc-1bbf-4068-9076-40d6da61a07c/index.html#cluster-5) | [Cf_putative](http://antismash.secondarymetabolites.org/help#cf_putative) | 979653 | 985429 | - |  | - |
| [6](https://antismash.secondarymetabolites.org/upload/bacteria-2201b9dc-1bbf-4068-9076-40d6da61a07c/index.html#cluster-6) | [Cf_putative](http://antismash.secondarymetabolites.org/help#cf_putative) | 1260337 | 1272919 | - |  | - |
| [7](https://antismash.secondarymetabolites.org/upload/bacteria-2201b9dc-1bbf-4068-9076-40d6da61a07c/index.html#cluster-7) | [Cf_putative](http://antismash.secondarymetabolites.org/help#cf_putative) | 1295279 | 1303420 | - |  | - |
| [8](https://antismash.secondarymetabolites.org/upload/bacteria-2201b9dc-1bbf-4068-9076-40d6da61a07c/index.html#cluster-8) | [Cf_fatty_acid/](http://antismash.secondarymetabolites.org/help#cf_fatty_acid%20)[Cf_saccharide](http://antismash.secondarymetabolites.org/help#cf_saccharide) | 1506532 | 1543075 | Marinacarboline | 15 | [BGC0001137_c1](http://mibig.secondarymetabolites.org/repository/BGC0001137/index.html) |
| [9](https://antismash.secondarymetabolites.org/upload/bacteria-2201b9dc-1bbf-4068-9076-40d6da61a07c/index.html#cluster-9) | [Cf_putative](http://antismash.secondarymetabolites.org/help#cf_putative) | 1697988 | 1706020 | Polysaccharide_B | 6 | [BGC0001411_c1](http://mibig.secondarymetabolites.org/repository/BGC0001411/index.html) |
| [10](https://antismash.secondarymetabolites.org/upload/bacteria-2201b9dc-1bbf-4068-9076-40d6da61a07c/index.html#cluster-10) | [Cf_putative](http://antismash.secondarymetabolites.org/help#cf_putative) | 1932818 | 1942524 | - |  | - |
| [11](https://antismash.secondarymetabolites.org/upload/bacteria-2201b9dc-1bbf-4068-9076-40d6da61a07c/index.html#cluster-11) | [Cf_putative](http://antismash.secondarymetabolites.org/help#cf_putative) | 2050198 | 2064158 | - |  | - |
| [12](https://antismash.secondarymetabolites.org/upload/bacteria-2201b9dc-1bbf-4068-9076-40d6da61a07c/index.html#cluster-12) | [Cf_putative](http://antismash.secondarymetabolites.org/help#cf_putative) | 2135116 | 2144589 | - |  | - |
| [13](https://antismash.secondarymetabolites.org/upload/bacteria-2201b9dc-1bbf-4068-9076-40d6da61a07c/index.html#cluster-13) | [Cf_putative](http://antismash.secondarymetabolites.org/help#cf_putative) | 2165473 | 2176502 | - |  | - |
| [14](https://antismash.secondarymetabolites.org/upload/bacteria-2201b9dc-1bbf-4068-9076-40d6da61a07c/index.html#cluster-14) | [Cf_fatty_acid](http://antismash.secondarymetabolites.org/help#cf_fatty_acid%20) | 2711704 | 2732657 | - |  | - |
| [15](https://antismash.secondarymetabolites.org/upload/bacteria-2201b9dc-1bbf-4068-9076-40d6da61a07c/index.html#cluster-15) | [Cf_putative](http://antismash.secondarymetabolites.org/help#cf_putative) | 2795605 | 2805225 | - |  | - |
| [16](https://antismash.secondarymetabolites.org/upload/bacteria-2201b9dc-1bbf-4068-9076-40d6da61a07c/index.html#cluster-16) | [Cf_fatty_acid](http://antismash.secondarymetabolites.org/help#cf_fatty_acid%20) | 2836328 | 2857602 | Taxlllaid | 33 | [BGC0001133_c1](http://mibig.secondarymetabolites.org/repository/BGC0001133/index.html) |
| [17](https://antismash.secondarymetabolites.org/upload/bacteria-2201b9dc-1bbf-4068-9076-40d6da61a07c/index.html#cluster-17) | [Thiopeptide](http://antismash.secondarymetabolites.org/help#thiopeptide) | 2894393 | 2920851 | O-antigen | 14 | [BGC0000781_c1](http://mibig.secondarymetabolites.org/repository/BGC0000781/index.html) |
| [18](https://antismash.secondarymetabolites.org/upload/bacteria-2201b9dc-1bbf-4068-9076-40d6da61a07c/index.html#cluster-18) | [Cf_putative](http://antismash.secondarymetabolites.org/help#cf_putative) | 2946724 | 2955330 | - |  | - |
| [19](https://antismash.secondarymetabolites.org/upload/bacteria-2201b9dc-1bbf-4068-9076-40d6da61a07c/index.html#cluster-19) | [Cf_saccharide](http://antismash.secondarymetabolites.org/help#cf_saccharide) | 3012014 | 3039340 | Lipopolysaccharide | 11 | [BGC0000774_c1](http://mibig.secondarymetabolites.org/repository/BGC0000774/index.html) |
| [20](https://antismash.secondarymetabolites.org/upload/bacteria-2201b9dc-1bbf-4068-9076-40d6da61a07c/index.html#cluster-20) | [Cf_saccharide](http://antismash.secondarymetabolites.org/help#cf_saccharide) | 3041888 | 3068192 | Capsular_polysaccharide | 40 | [BGC0000731_c1](http://mibig.secondarymetabolites.org/repository/BGC0000731/index.html) |
| [21](https://antismash.secondarymetabolites.org/upload/bacteria-2201b9dc-1bbf-4068-9076-40d6da61a07c/index.html#cluster-21) | [Cf_putative](http://antismash.secondarymetabolites.org/help#cf_putative) | 3251406 | 3263762 | - |  | - |
| [22](https://antismash.secondarymetabolites.org/upload/bacteria-2201b9dc-1bbf-4068-9076-40d6da61a07c/index.html#cluster-22) | [Cf_putative](http://antismash.secondarymetabolites.org/help#cf_putative) | 3532782 | 3538997 | - |  | - |
| [23](https://antismash.secondarymetabolites.org/upload/bacteria-2201b9dc-1bbf-4068-9076-40d6da61a07c/index.html#cluster-23) | [Cf_putative](http://antismash.secondarymetabolites.org/help#cf_putative) | 3542378 | 3553817 | - |  | - |
| [24](https://antismash.secondarymetabolites.org/upload/bacteria-2201b9dc-1bbf-4068-9076-40d6da61a07c/index.html#cluster-24) | [Nrps](http://antismash.secondarymetabolites.org/help#nrps) | 3717689 | 3769630 | Pseudomonine | 20 | [BGC0000410_c1](http://mibig.secondarymetabolites.org/repository/BGC0000410/index.html) |
| [25](https://antismash.secondarymetabolites.org/upload/bacteria-2201b9dc-1bbf-4068-9076-40d6da61a07c/index.html#cluster-25) | [Arylpolyene](http://antismash.secondarymetabolites.org/help#arylpolyene)-[Siderophore](http://antismash.secondarymetabolites.org/help#siderophore) | 3781438 | 3845994 | APE_Ec | 84 | [BGC0000836_c1](http://mibig.secondarymetabolites.org/repository/BGC0000836/index.html) |
| [26](https://antismash.secondarymetabolites.org/upload/bacteria-2201b9dc-1bbf-4068-9076-40d6da61a07c/index.html#cluster-26) | [Cf_putative](http://antismash.secondarymetabolites.org/help#cf_putative) | 4085879 | 4090846 | Paerucumarin | 40 | [BGC0000923_c1](http://mibig.secondarymetabolites.org/repository/BGC0000923/index.html) |
| [27](https://antismash.secondarymetabolites.org/upload/bacteria-2201b9dc-1bbf-4068-9076-40d6da61a07c/index.html#cluster-27) | [Cf_putative](http://antismash.secondarymetabolites.org/help#cf_putative) | 4223776 | 4231231 | - |  | - |
| [28](https://antismash.secondarymetabolites.org/upload/bacteria-2201b9dc-1bbf-4068-9076-40d6da61a07c/index.html#cluster-28) | [Nrps](http://antismash.secondarymetabolites.org/help#nrps) | 4235918 | 4281650 | Malleobactin | 7 | [BGC0000386_c1](http://mibig.secondarymetabolites.org/repository/BGC0000386/index.html) |
| [29](https://antismash.secondarymetabolites.org/upload/bacteria-2201b9dc-1bbf-4068-9076-40d6da61a07c/index.html#cluster-29) | [Other](http://antismash.secondarymetabolites.org/help#other) | 4305890 | 4348871 | - |  | - |
| [30](https://antismash.secondarymetabolites.org/upload/bacteria-2201b9dc-1bbf-4068-9076-40d6da61a07c/index.html#cluster-30) | [Cf_putative](http://antismash.secondarymetabolites.org/help#cf_putative) | 4616210 | 4625229 | - |  | - |
| [31](https://antismash.secondarymetabolites.org/upload/bacteria-2201b9dc-1bbf-4068-9076-40d6da61a07c/index.html#cluster-31) | [Cf_putative](http://antismash.secondarymetabolites.org/help#cf_putative) | 4652666 | 4662352 | O&K-antigen | 3 | [BGC0000780_c1](http://mibig.secondarymetabolites.org/repository/BGC0000780/index.html) |
| [32](https://antismash.secondarymetabolites.org/upload/bacteria-2201b9dc-1bbf-4068-9076-40d6da61a07c/index.html#cluster-32) | [Cf_putative](http://antismash.secondarymetabolites.org/help#cf_putative) | 4711603 | 4717750 | - |  | - |
| [33](https://antismash.secondarymetabolites.org/upload/bacteria-2201b9dc-1bbf-4068-9076-40d6da61a07c/index.html#cluster-33) | [Cf_saccharide](http://antismash.secondarymetabolites.org/help#cf_saccharide) | 4939461 | 4971677 | Lipopolysaccharide | 27 | [BGC0000776_c1](http://mibig.secondarymetabolites.org/repository/BGC0000776/index.html) |
| [34](https://antismash.secondarymetabolites.org/upload/bacteria-2201b9dc-1bbf-4068-9076-40d6da61a07c/index.html#cluster-34) | [Nrps](http://antismash.secondarymetabolites.org/help#nrps) | 5351208 | 5395152 | - |  | - |
| [35](https://antismash.secondarymetabolites.org/upload/bacteria-2201b9dc-1bbf-4068-9076-40d6da61a07c/index.html#cluster-35) | [Cf_putative](http://antismash.secondarymetabolites.org/help#cf_putative) | 5500683 | 5509483 | - |  | - |
